# Deoxygenative trifluoromethylthiolation of carboxylic acids†
†Electronic supplementary information (ESI) available. See DOI: 10.1039/c9sc03396c


**DOI:** 10.1039/c9sc03396c

**Published:** 2019-08-26

**Authors:** Runze Mao, Srikrishna Bera, Alexis Cheseaux, Xile Hu

**Affiliations:** a Laboratory of Inorganic Synthesis and Catalysis , Institute of Chemical Sciences and Engineering , École Polytechnique Fédérale de Lausanne (EPFL) , ISIC-LSCI , Lausanne 1015 , Switzerland . Email: xile.hu@epfl.ch

## Abstract

Here we describe a deoxygenative trifluoromethylthiolation method that yields trifluoromethyl thioesters from readily available carboxylic acids. The method is built upon an “umpolung” strategy where triphenylphosphine is used to first activate an electrophilic trifluoromethylthiolating reagent and then serves as an oxygen acceptor for the deoxygenation. The method is mild, efficient, broad-scope, and tolerant. It can be applied for the late-stage functionalization of numerous natural products and drug molecules containing a carboxylic acid group. The trifluoromethyl thioesters can be converted into trifluoromethyl thioethers by Pd-catalyzed decarbonylation.

## Abstract 2

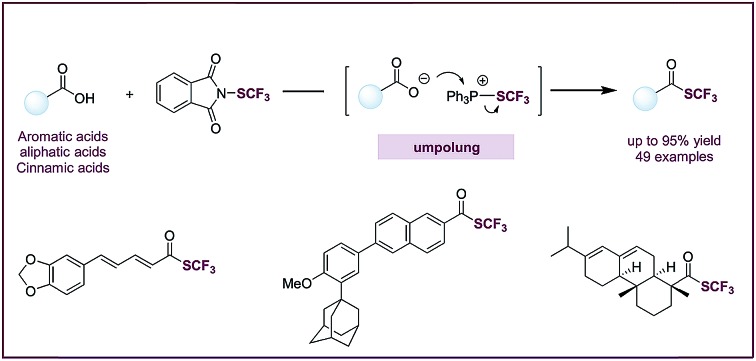
A deoxygenative trifluoromethylthiolation method produces trifluoromethyl thioesters from readily available carboxylic acids.

Organofluorine compounds have widespread applications in medicinal and materials sciences.^1^–^4^ Among fluorine-containing moieties, the trifluoromethylthio group (–SCF_3_) is of considerable interest because of its high lipophilic and electron-withdrawing nature. Significant progress has been made in direct trifluoromethylthiolation of C–H and C–X moieties.^4^–^19^ However, there are still few efficient methods for the synthesis of trifluoromethyl thioesters. Trifluoromethyl thioesters could be prepared by reactions of acid chlorides with Hg(SCF_3_)_2_, NMe_4_SCF_3_ or (bpy)CuSCF_3_ (Fig. 1A).^17^,^20^,^21^ These reactions were limited by the use of reactive or toxic reagents, or the generation of a stoichiometric amount of metallic by-products. The groups of Glorius^18^ and Shen^19^ reported elegant methods of accessing trifluoromethyl thioesters from aldehydes *via* a hydrogen atom transfer (HAT) process (Fig. 1A). Nevertheless, among organic carbonyl compounds aldehydes are relatively instable and less available. Carboxylic acids, on the other hand, are abundant, stable, and non-toxic. The deoxygenative trifluoromethylthiolation of carboxylic acids would represent an efficient and highly desirable approach to the synthesis of trifluoromethyl thioesters.

**Fig. 1:**
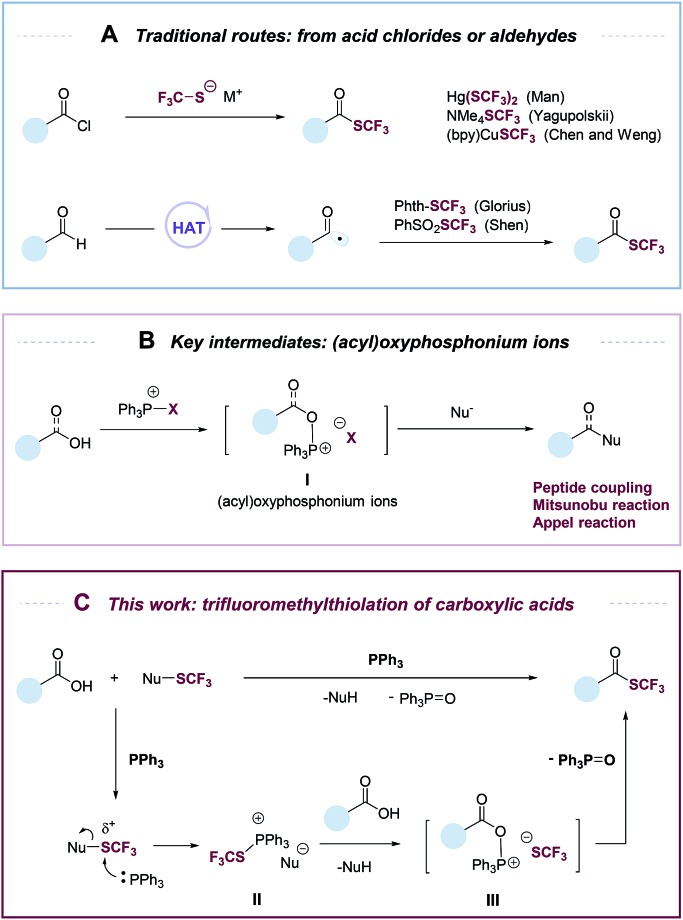
(A) Previous methods to synthesize trifluoromethyl thioesters from acid chlorides or aldehydes; (B) key intermidiates: (acyl)oxyphosphonium ions; (C) this work: deoxygenative trifluoromethylthiolation of carboxylic acids.

Despite its conceptual simplicity, the deoxygenative trifluoromethylthiolation of carboxylic acids is challenging to achieve. It was reported that in the presence of a carboxylic acid, a CF_3_S^–^ anion would lose an F^–^ to form carbonothioic difluoride, which further reacted with carboxylic acids to give eventually an acyl fluoride.^10^,^22^ Inspired by the rich chemistry of phosphorus reagents and (acyl)oxyphosphonium ions I (Fig. 1B) in peptide coupling,^23^ Mitsunobu,^24^,^25^ and Appel^26^ reactions, we hypothesized that such intermediates could be possibly transformed into trifluoromethyl thioesters from carboxylic acids under suitable conditions. Here we describe an “umpolung” strategy that allows the use of electrophilic trifluoromethylthiolating reagents and avoids the decomposition of CF_3_S^–^ anion by carboxylic acids (Fig. 1C). The “umpolung” is achieved with triphenylphosphine (PPh_3_), which first captures a CF_3_S^+^ cation to form a SCF_3_-phosphonium salt (**II**), followed by a metathesis reaction with a carboxylate to give an oxyphosphonium intermediate (**III**), which is probe to deoxygenative trifluoromethylthiolation to give a trifluoromethyl thioester while eliminating triphenylphosphine oxide (PPh_3_=O) (Fig. 1C). This strategy is applicable for the rapid synthesis of a diverse set of trifluoromethyl thioesters from readily available aromatic and aliphatic carboxylic acids, including many natural products and drugs.

We began our investigation by optimizing the reaction of 4-phenylbenzoic acid (**1a**) with *N*-(trifluoromethylthio)phthalimide (**2a**) to give the corresponding trifluoromethyl thioester (**3a**). To our delight, the reaction proceeded in the presence of 1.1 equiv. of Ph_3_P in tetrahydrofuran (THF, 0.2 M) with a yield of 40% (Table 1, entry 1). The reaction was then optimized by varying reaction parameters (Table ESI, S1–S5†). A summary of key observations is shown in Table 1. THF was the best solvent (Table S2†). A small amount of Lewis acid could enhance the reactivity of **2a** (Table 1, entries 2–6; Table S3†). The binding of a Lewis acid by the phthalimide group might polarize *N*-(trifluoromethylthio)phthalimide, facilitating the nucleophilic attack of PPh_3_ to *N*-(trifluoromethylthio)phthalimide and subsequent generation of the key intermediate SCF_3_-phosphonium salt (Fig. 1C, **II**). Anhydrous FeCl_3_ (5 mol%) was the best Lewis acid, giving a yield of 95% (Table 1, entry 2). Among various trifluoromethylthiolating agents,^27^**2a** proved to be superior than other electrophilic trifluoromethylthiolating reagent **2b–2d** (Table 1, entries 7–9). When the nucleophilic NMe_4_SCF_3_ (**2e**) was used, no product was formed (Table 1, entry 10). Addition of an external base slightly lowered the yields (Table 1, entries 11 and 12; Table S4†). Further optimization indicated Ph_3_P was the best mediator (Table 1, entries 2 and 13; Table S5†). It was worthy to note that the reaction completed within 30 minutes at room temperature.

**Table 1:** Summary of the effects of reaction parameters and conditions on the reaction efficiency^*a*^

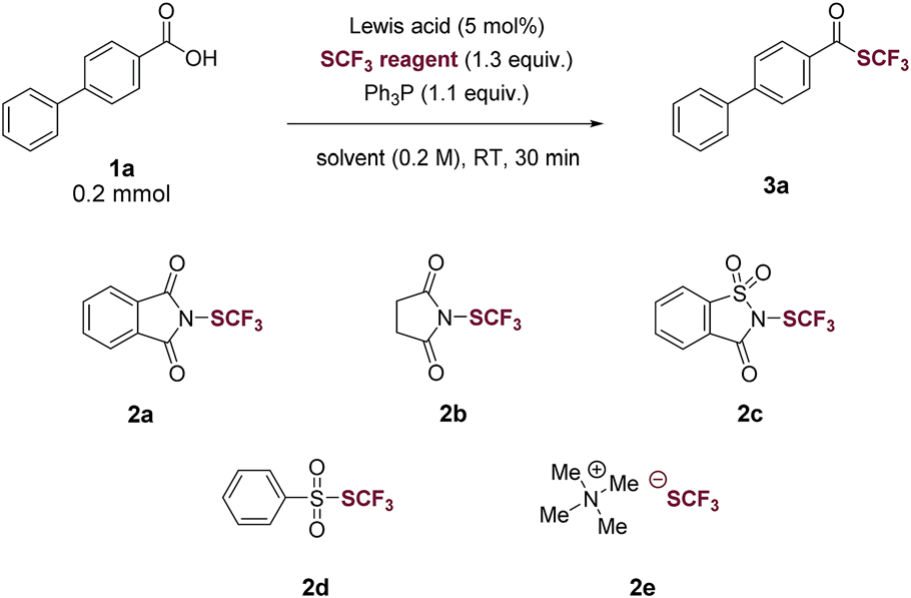			
Entry	SCF_3_ reagent	Lewis acid	Yield
1	**2a**	None	40%
2	**2a**	FeCl_3_	95%
3	**2a**	FeCl_3_·6H_2_O	71%
4^*b*^	**2a**	BF_3_·OEt_2_	64%
5	**2a**	AlCl_3_	39%
6	**2a**	Sc(OTf)_3_	71%
7	**2b**	FeCl_3_	19%
8	**2c**	FeCl_3_	51%
9	**2d**	FeCl_3_	7%
10	**2e**	FeCl_3_	0%
11^*c*^	**2a**	FeCl_3_	69%
12^*d*^	**2a**	FeCl_3_	68%
13^*e*^	**2a**	FeCl_3_	39%

^*a*^Yield determined by ^19^F NMR spectroscopy of the crude reaction mixture using α,α,α-trifluorotoluene as an internal standard.

^*b*^BF_3_·OEt_2_ (10 mol%).

^*c*^NaHCO_3_ (1.0 equiv.) as an external base.

^*d*^2,6-Lutidine (1.0 equiv.) as an external base.

^*e*^Tricyclohexanephosphine (PCy_3_) in place of PPh_3_.

With the optimized conditions in hand (entry 2, Table 1), we probed the generality of this transformations (Table 2). A myriad of aryl carboxylic acids containing electron-donating (**3b–3f**) and electron-withdrawing (**3g–3o**) substituents were coupled to give the corresponding trifluoromethyl thioesters in moderate to excellent yields. Notable, aryl halides (**3g–3m**), including relatively reactive aryl iodides (**3j**, **3m**), were tolerated in the reaction. Functional groups such as trifluoromethyl (**3n**), ester (**3o**), (*tert*-butoxycarbonyl)amino (**3p**), thiomethyl (**3q**), boronic ester (**3r**), alkene (**3s**), alkyne (**3t**), 1,3-benzodioxole (**3u**) and naphthalene (**3v**) were all compatible. The reactions also proceeded smoothly with various heteroaryl carboxylic acids, giving the desired products (**3w–3x**) in satisfying yields. Importantly, the reactions worked with aliphatic carboxylic acids as well. Primary, secondary, tertiary carboxylic acids were all suitable substrates, affording the corresponding trifluoromethyl thioesters (**4a–4e**) in good yields. The trifluoromethylthiolation was also successful for various cinnamic acids containing electron-neutral (**5a**), electron-withdrawing (**5b–5e**, **5h**), and electron-donating (**5f–5g**) substituents. A variety of alcohols and substituted methyl benzoates were also tested as substrates (Table S7†). Reactions with methyl benzoates gave no products under the standard conditions, suggesting a crucial role of the *O*-nucleophilic site of carboxylic acids. Reactions of some alcohols, especially primary alcohols, gave the desired products in low yields, which were difficult to isolate amide various side products. Several amino acids were also used as substrates, however, yields were low (Table S8†), possibly due to the competition of *N*- and *O*-nucleophilic sites.

**Table 2:** Scope of the trifluoromethylthiolation of carboxylic acids^*a*^

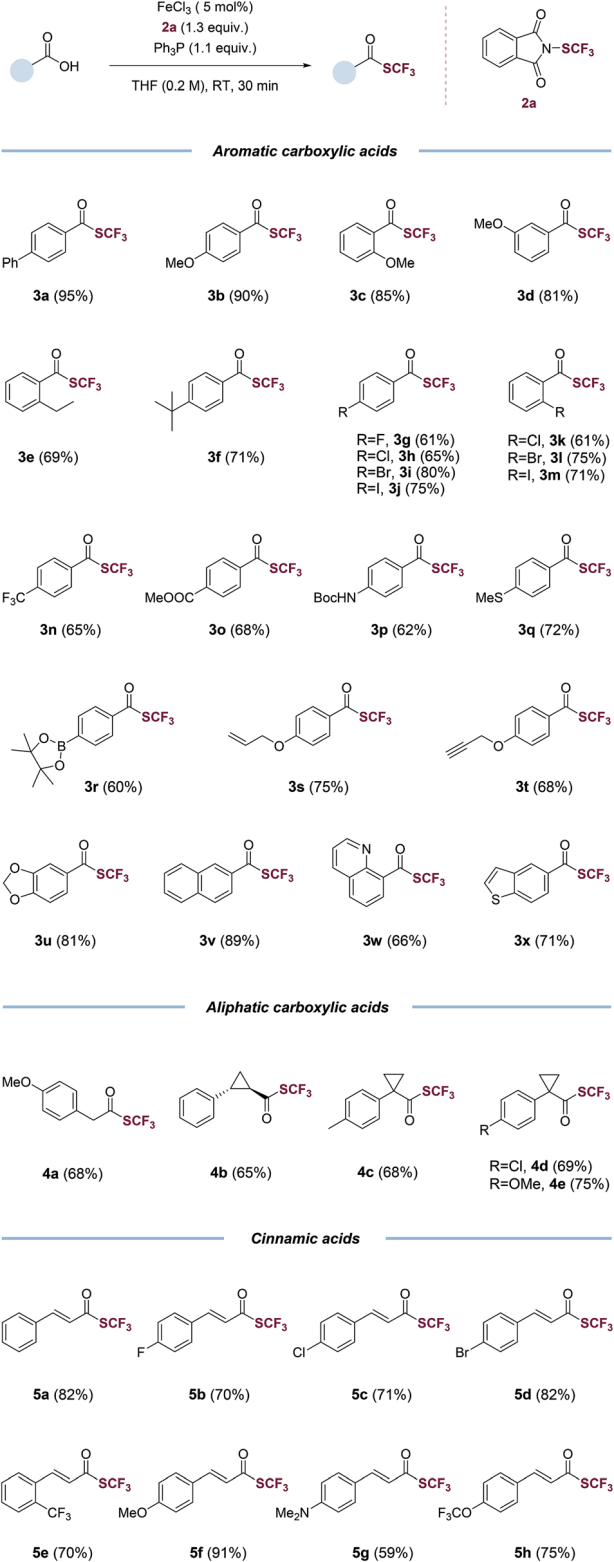

^*a*^Carboxylic acid (1.0 equiv.), triphenylphosphine (Ph_3_P, 1.1 equiv.), *N*-(trifluoromethylthio)phthalimide (1.3 equiv.), FeCl_3_ (5 mol%) in THF (0.2 M), room temperature, 30 min, isolated yield.

The direct use of carboxylic acids as substrates makes the current trifluoromethylthiolation method applicable for the rapid, late-stage modification of carboxylic acid-containing natural products and drug molecules (Table 3). Indeed, aromatic carboxylic acids such as adapalene (**6a**), probenecid (**6b**), lanosterol (**6c**), l-menthol derivative (**6d**) were trifluoromethylthiolated with ease. Moreover, natural-occurring cinnamic acids such as piperic acid (**6e**) and caffeic acid isomer (**6f**) underwent smooth transformations as well. Drug molecules and natural products containing an aliphatic carboxylic acid group such as zaltoprofen (**6g**), ibuprofen (**6h**), ketoprofen (**6i**), naproxen (**6j**), gemfibrozil (**6k**) and abietic acid (**6l**) were all easily converted to their corresponding trifluoromethyl thioesters. The successful late-stage functionalization of these natural products and drugs, many of which contain sensitive sulfonamide, alkene, carbonyl and heterocyclic groups, underscores the high chemoselectivity and functional group tolerance of the current method (Table 3).

**Table 3:** Late-stage trifluoromethylthiolation of natural products and drugs containing a carboxylic group^*a*^

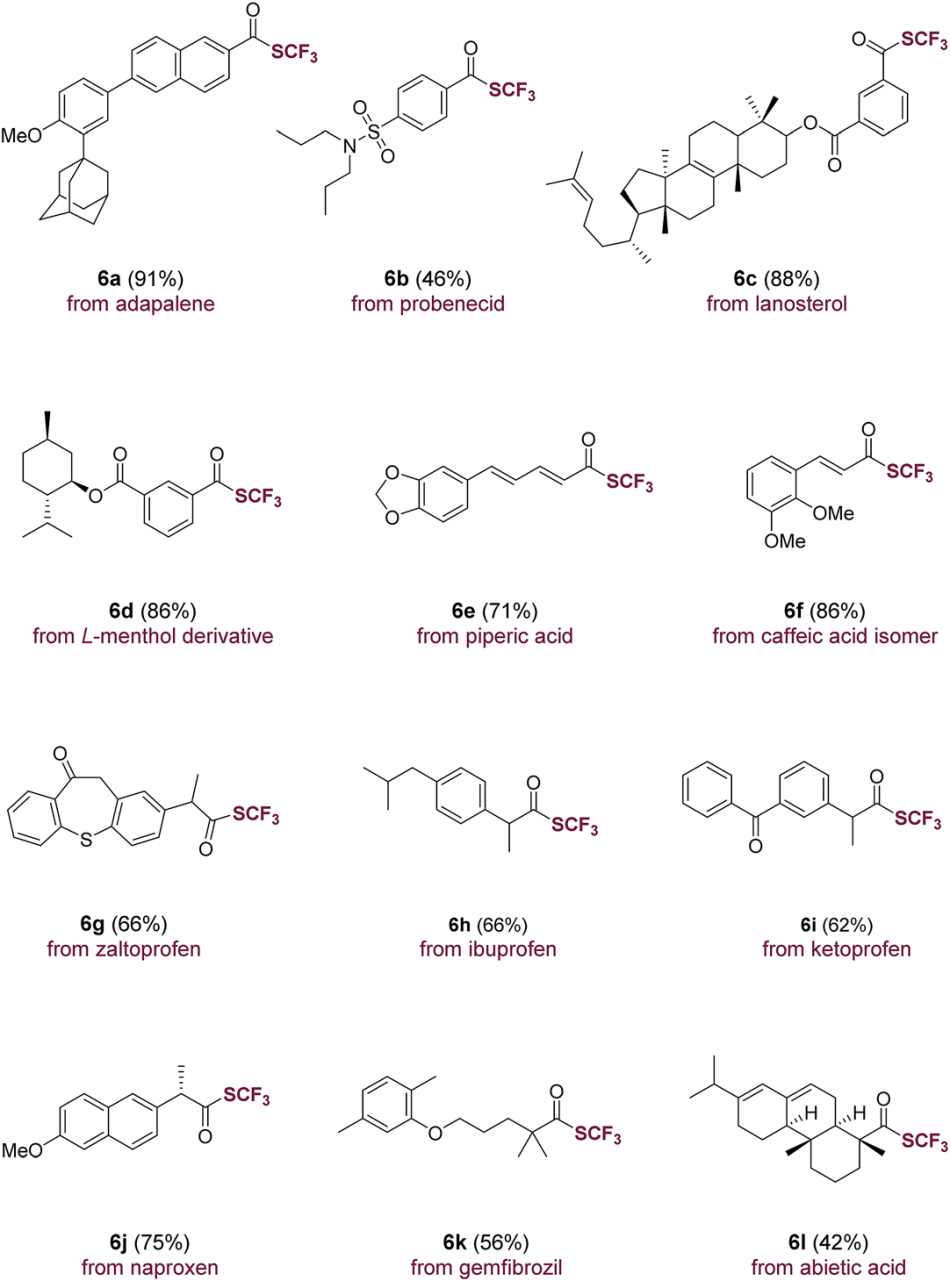

^*a*^Carboxylic acid (1.0 equiv.), triphenylphosphine (Ph_3_P, 1.1 equiv.), *N*-(trifluoromethylthio)phthalimide (1.3 equiv.), FeCl_3_ (5 mol%) in THF (0.2 M), room temperature, 30 min, isolated yield.

To demonstrate the synthetic utility of trifluoromethyl thioesters, compound **3a** was subjected to a Pd-catalyzed decarbonylation^28^,^29^ to give the corresponding trifluoromethyl thioether **7a** in 91% yield (Fig. 2). Trifluoromethyl thioethers are ubiquitous in pharmaceutical and agrochemical compounds.^4^ Thus, our method enables the synthesis of trifluoromethyl thioethers from readily available carboxylic acids.

**Fig. 2:**
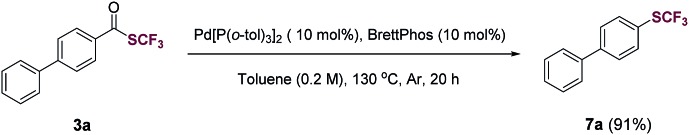
Conversion of a trifluoromethyl thioester (**3a**) to the corresponding trifluoromethyl thioether (**7a**) *via* a Pd-catalyzed decarbonylation.

Based on results from the control experiments (Table 1 and S1–S5†),^30^^31^P NMR study (Fig. S51†), and previous reports,^31^,^32^ we propose a tentative mechanism for the deoxygenative trifluoromethylthiolation (Fig. 3). *N*-(Trifluoromethylthio)phthalimide (**2a**) coordinates to FeCl_3_*via* the phthalimide group. This coordination increases the electrophilicity of the SCF_3_ group, promoting the nucleophilic attack of PPh_3_. The latter generates a trifluoromethylthiophosphonium ion **II**, which reacts with a carboxylic acid to generate an acyloxyphosphonium CF_3_S^–^ intermediate **III**. Intramolecular attack of the CF_3_S^–^ anion on the acyl carbon of **III** then gives the thioester product as well as the Ph_3_PO byproduct.

**Fig. 3:**
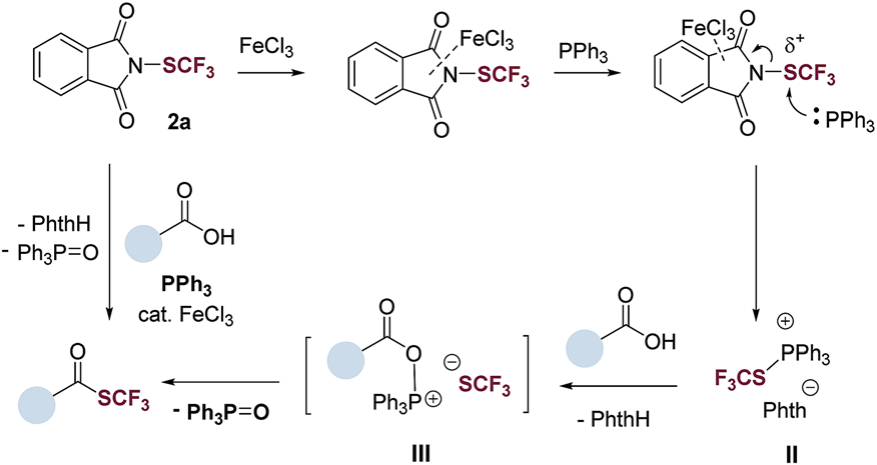
A tentative reaction pathway.

## Conclusions

In summary, by using PPh_3_ as a mediator to “umpolung” the electrophilic trifluoromethylthiolating agent **2a**, we have achieved, for the first time, deoxygenative trifluoromethylthiolation of carboxylic acids. The reactions are rapid and occur at room temperature. They allow the access of a wide range of trifluoromethyl thioesters from readily available carboxylic acids. The method can be applied for the late-stage functionalization of many natural products and drug molecules. The trifluoromethyl thioesters can be converted into trifluoromethyl thioethers in one step by Pd-catalyzed decarbonylation.

## Conflicts of interest

The authors declare no conflict of interest.

## Supplementary Material

Supplementary information
