# Supplementary material for: Deoxygenative trifluoromethylthiolation of carboxylic acids
Source: Chem Sci. 2019 Aug 26;10(41):9555–9. doi: 10.1039/c9sc03396c (PMC6979494; doi:10.1039/c9sc03396c)
Supplement: Supplementary file 1 [file SC-010-C9SC03396C-s001.pdf]

Electronic Supplementary Material (ESI) for Chemical Science.
This journal is © The Royal Society of Chemistry 2019

Supporting Information

Deoxygenative Trifluoromethylthiolation of Carboxylic Acids

Runze Mao, Srikrishna Bera, Alexis Cheseaux, and Xile Hu*

Laboratory of Inorganic Synthesis and Catalysis, Institute of Chemical Sciences and Engineering, École Polytechnique Fédérale de Lausanne (EPFL), ISIC-LSCI, BCH 3305, Lausanne 1015, Switzerland

Table of contents

1.**Methods** S2
2.**Tables** S30
3.**Figures** S35
4.**References** S136

Methods

General Analytical Information

Nuclear Magnetic Resonance spectra were recorded on a Bruker Avance 400 MHz instruments at ambient temperature. All ^{1}H NMR spectra were measured in part per million (ppm) relative to the signals of tetramethylsilane (TMS, 0.00 ppm) added into the deuterated chloroform ($CDCl_3$, 7.26 ppm), deuterated dichloromethane (CD_2Cl_2, 5.32 ppm), unless otherwise stated.[1] Data for ^{1}H NMR were reported as follows: chemical shift, multiplicity (s = singlet, d = doublet, t = triplet, q = quartet, quint = quintet, m = multiplet, dd = doublet of doublets, dt = doublet of triplets, td = triplet of doublets, and br = broad signal), coupling constants, and integration. All ^{13}C NMR spectra were reported in ppm relative to $CDCl_3$ (77.16 ppm), CD_2Cl_2 (53.84 ppm), unless otherwise stated,[1] and were obtained with complete ^{1}H decoupling. All ^{19}F NMR spectra were reported in ppm unless otherwise stated, and were obtained with complete ^{1}H decoupling. All GC analyses were performed on a Perkin-Elmer Clarus 400 GC system with a FID detector. All GC-MS analyses were performed on an Agilent Technologies 7890A GC system equipped with a 5975C MS detector. High-resolution mass spectra (HRMS) by electrospray ionization (ESI), atmospheric pressure chemical ionization (APCI) and atmospheric pressure photoionization (APPI) method were performed at the EPFL ISIC Mass Spectroscopy Service.

General Manipulation Considerations

Flash column chromatography was performed using silica gel (Silicycle, ultra-pure grade). The eluents for column chromatography were presented as ratios of solvent volumes. Yields reported in the publication are of isolated materials unless otherwise noted. All new products were characterized by ^{1}H, ^{13}C and ^{19}F NMR spectroscopy as well as high-resolution mass spectrometry (HRMS), and reported with physical state and the eluents for column chromatography were presented as ratios of solvent volumes. All known products were characterized by ^{1}H, ^{13}C and ^{19}F NMR spectroscopies and the spectra were compared with the reported data.

General procedure for deoxygenative trifluoromethylthiolation of carboxylic acids

Two oven-dried 15 mL re-sealable screw-cap vials (oven-dried) were both equipped with a Teflon-coated magnetic stirring bar (oven-dried), and were transferred into glovebox. One vial was sequentially charged with carboxylic acid (1.0 equiv., 0.30 mmol), triphenylphosphine (Ph_3P, 1.1 equiv., 0.33 mmol, 87 mg) and anhydrous Tetrahydrofuran (THF, 0.5 mL) in glovebox and the mixture was stirred vigorously. Another vial was sequentially charged with *N*-(trifluoromethylthio)phthalimide (1.3 equiv., 0.39 mmol, 96 mg), anhydrous $FeCl_3$ (5 mol%, 2.4 mg), and anhydrous THF (1 mL) in the glovebox. Then the 2 vials were transferred out of the glovebox. The solution of the second vial was added into the first one dropwise under nitrogen at room temperature. The resulting mixture was stirred at room temperature for 30 minutes during which time the color of the mixture change from red to bright yellow. After the reaction, the solvent was blown away by dry air and the reaction mixture was diluted with dichloromethane and was purified by flash column chromatography using a solvent mixture (ethyl acetate, hexanes or diethyl ether, *n*-pentane) as an eluent to afford the purified product.

Notice: Because of the high volatility of certain products, their isolated yields were low.

***S*-(trifluoromethyl) [1,1'-biphenyl]-4-carbothioate (3a)**

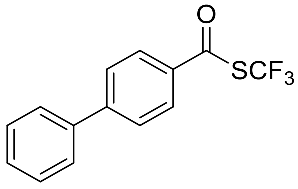

Following the General Procedure with the corresponding carboxylic acid (0.3 mmol). The crude product purified by flash column chromatography, using hexanes/EA = 30/1 (v/v) as an eluent, to yield the title compound **3a** (80 mg) in 95%.

^{1}H NMR (400 MHz, Chloroform-d): δ 7.98 – 7.88 (m, 2H), 7.75 – 7.70 (m, 2H), 7.65 – 7.60 (m, 2H), 7.52 – 7.42 (m, 3H).

^{13}C NMR (101 MHz, Chloroform-d): δ 183.25, 148.40, 139.64, 134.24 (q, *J* = 2.7 Hz), 129.64, 129.32, 128.77, 128.61 (q, *J* = 309.6 Hz), 128.26, 127.83.

^{19}F NMR (376 MHz, Chloroform-d): δ -39.50.

Physical State: white solid.

HRMS (EI) m/z: $[M]^+$ Calcd for $C_{14}H_9F_3OS^+$ 282.0321, Found 282.0321.

The spectra were consistent with the spectrum reported in the literature.[2]

***S*-(trifluoromethyl) 4-methoxybenzothioate (3b)**

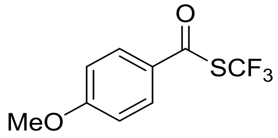

Following the General Procedure with the corresponding carboxylic acid (0.3 mmol). The crude product purified by flash column chromatography, using hexanes/EA = 30/1 (v/v) as an eluent, to yield the title compound **3b** (64 mg) in 90%.

^{1}H NMR (400 MHz, Chloroform-d): δ 7.82 (d, *J* = 8.9 Hz, 2H), 6.96 (d, *J* = 8.9 Hz, 2H), 3.89 (s, 3H).

^{13}C NMR (101 MHz, Chloroform-d): δ 181.48, 165.07, 130.09, 128.20 (q, *J* = 309.2 Hz), 127.82 (q, *J* = 2.7 Hz), 114.41, 55.70.

^{19}F NMR (376 MHz, Chloroform-d): δ -39.43.

Physical State: pale brown oil.

HRMS (ESI/QTOF) m/z: $[M + H]^+$ Calcd for $C_{10}H_7F_3O_2S^+$ 237.0192, Found 237.0193.

The spectra were consistent with the spectrum reported in the literature.[2]

***S*-(trifluoromethyl) 2-methoxybenzothioate (3c)**

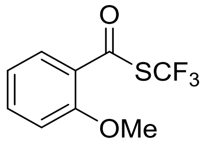

Following the General Procedure with the corresponding carboxylic acid (0.3 mmol). The crude product purified by flash column chromatography, using *n*-pentane/diethyl ether = 30/1 (v/v) as an eluent, to yield the title compound **3c** (60 mg) in 85%.

^{1}H NMR (400 MHz, Chloroform-d): δ 7.89 (dd, *J* = 7.9, 1.8 Hz, 1H), 7.58 (ddd, *J* = 8.2, 7.3, 1.8 Hz, 1H), 7.10 – 7.05 (m, 1H), 7.03 (d, *J* = 8.4 Hz, 1H), 3.99 (s, 3H).

^{13}C NMR (101 MHz, Chloroform-d): δ 182.01, 159.32, 136.03, 130.37, 128.31 (q, *J* = 310.1 Hz), 124.09 (q, *J* = 2.6 Hz), 121.29, 112.30, 56.01.

^{19}F NMR (376 MHz, Chloroform-d): δ -42.13.

Physical State: colorless oil.

HRMS (ESI/QTOF) m/z: $[M + H]^+$ Calcd for $C_{10}H_7F_3O_2S^+$ 237.0192, Found 237.0192.

The spectra were consistent with the spectrum reported in the literature.[3]

***S*-(trifluoromethyl) 2-methoxybenzothioate (3d)**

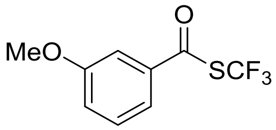

Following the General Procedure with the corresponding carboxylic acid (0.3 mmol). The crude product purified by flash column chromatography, using *n*-pentane/diethyl ether = 30/1 (v/v) as an eluent, to yield the title compound **3d** (57 mg) in 81%.

^{1}H NMR (400 MHz, Chloroform-d): δ 7.46 – 7.39 (m, 2H), 7.36 (s, 1H), 7.22 – 7.18 (m, 1H), 3.86 (s, 3H).

^{13}C NMR (101 MHz, Chloroform-d): δ 183.36, 160.27, 136.54, 130.36, 128.14 (q, *J* = 309.6 Hz), 121.67, 120.28, 111.93, 55.74.

^{19}F NMR (376 MHz, Chloroform-d): δ -39.77.

Physical State: colorless oil.

HRMS (ESI/QTOF) m/z: $[M + Na]^+$ Calcd for $C_9H_7F_3O_2SNa^+$ 259.0017, Found 259.0018.

The spectra were consistent with the spectrum reported in the literature.[3]

***S*-(trifluoromethyl) 2-ethylbenzothioate (3e)**

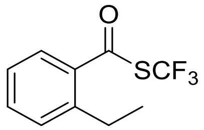

Following the General Procedure with the corresponding carboxylic acid (0.3 mmol). The crude product purified by flash column chromatography, using *n*-pentane/diethyl ether = 30/1 (v/v) as an eluent, to yield the title compound **3e** (48 mg) in 69%.

^{1}H NMR (400 MHz, Chloroform-d): δ 7.67 (dd, *J* = 7.8, 1.4 Hz, 1H), 7.52 (td, *J* = 7.6 Hz, 1.4 Hz, 1H), 7.32 (ddd, *J* = 15.2, 7.7, 1.2 Hz, 2H), 2.88 (q, *J* = 7.5 Hz, 2H), 1.24 (td, *J* = 7.5, 1.1 Hz, 4H).

^{13}C NMR (101 MHz, Chloroform-d): δ 184.84, 144.67, 134.65 (q, *J* = 2.7 Hz), 133.67, 130.94, 128.74, 128.05 (q, *J* = 309.9 Hz), 126.36, 26.97, 15.81.

^{19}F NMR (376 MHz, Chloroform-d): δ -40.61.

Physical State: yellowish oil.

HRMS (APPI/LTQ-Orbitrap) m/z: $[M + H]^+$ Calcd for $C_{10}H_{10}F_3OS^+$ 235.0399, Found 235.0399.

The spectra were consistent with the spectrum reported in the literature.[2]

***S*-(trifluoromethyl) 4-(*tert*-butyl)benzothioate (3f)**

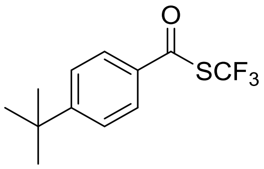

Following the General Procedure with the corresponding carboxylic acid (0.3 mmol). The crude product purified by flash column chromatography, using *n*-pentane/diethyl ether = 30/1 (v/v) as an eluent, to yield the title compound **3f** (56 mg) in 71%.

^{1}H NMR (400 MHz, Chloroform-d): δ 7.87 – 7.72 (m, 2H), 7.59 – 7.45 (m, 2H), 1.35 (s, 9H).

^{13}C NMR (101 MHz, Chloroform-d): δ 182.73, 159.30, 132.51 (q, *J* = 2.8 Hz), 128.15 (q, *J* = 309.3 Hz), 127.65, 126.19, 35.42, 30.96.

^{19}F NMR (376 MHz, Chloroform-d): δ -39.57.

Physical State: yellowish oil.

HRMS (ESI/QTOF) m/z: $[M + Na]^+$ Calcd for $C_{12}H_{13}F_3OSNa^+$ 285.0537, Found 285.0534.

The spectra were consistent with the spectrum reported in the literature.[2]

***S*-(trifluoromethyl) 4-fluorobenzothioate (3g)**

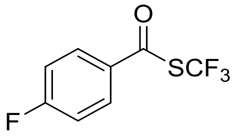

Following the General Procedure with the corresponding carboxylic acid (0.3 mmol). The crude product purified by flash column chromatography, using *n*-pentane/diethyl ether = 30/1 (v/v) as an eluent, to yield the title compound **3g** (41 mg) in 61%.

^{1}H NMR (400 MHz, Chloroform-d): δ 7.90 (dd, *J* = 8.5, 5.1 Hz, 2H), 7.20 (t, *J* = 8.3 Hz, 2H).

^{13}C NMR (101 MHz, Chloroform-d): δ 181.91, 168.23, 165.66, 131.62, 130.61, 130.51, 128.00 (q, *J* = 309.8 Hz), 116.84, 116.62.

^{19}F NMR (376 MHz, Chloroform-d): δ -39.56, -101.01.

Physical State: yellowish oil.

HRMS (APPI/LTQ-Orbitrap) m/z: $[M + H]^+$ Calcd for $C_8H_5F_4OS^+$ 224.9992; Found 224.9994.

The spectra were consistent with the spectrum reported in the literature.[2]

***S*-(trifluoromethyl) 4-chlorobenzothioate (3h)**

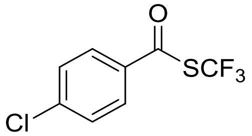

Following the General Procedure with the corresponding carboxylic acid (0.3 mmol). The crude product purified by flash column chromatography, using *n*-pentane/diethyl ether = 30/1 (v/v) as an eluent, to yield the title compound **3h** (47 mg) in 65%.

^{1}H NMR (400 MHz, Chloroform-d): δ 7.80 (d, *J* = 8.2 Hz, 2H), 7.50 (d, *J* = 8.2 Hz, 2H).

^{13}C NMR (101 MHz, Chloroform-d): δ 182.32, 141.90, 133.56 (q, *J* = 2.7 Hz), 129.75, 129.09, 127.95 (q, *J* = 310.0 Hz).

^{19}F NMR (376 MHz, Chloroform-d): δ -39.53.

Physical State: yellowish oil.

HRMS (APPI/LTQ-Orbitrap) m/z: $[M + H]^+$ Calcd for $C_8H_5F_3ClOS^+$ 240.9696; Found 240.9698.

The spectra were consistent with the spectrum reported in the literature.[2]

***S*-(trifluoromethyl) 4-bromobenzothioate (3i)**

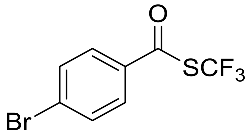

Following the General Procedure with the corresponding carboxylic acid (0.3 mmol). The crude product purified by flash column chromatography, using *n*-pentane/diethyl ether = 30/1 (v/v) as an eluent, to yield the title compound **3i** (68 mg) in 80%.

^{1}H NMR (400 MHz, Chloroform-d): δ 7.75 – 7.62 (m, 4H).

^{13}C NMR (101 MHz, Chloroform-d): δ 182.54, 133.98 (q, *J* = 2.7 Hz), 132.73, 130.63, 129.09, 127.91 (q, *J* = 309.9 Hz).

^{19}F NMR (376 MHz, Chloroform-d): δ -39.54.

Physical State: yellowish solid.

HRMS (APPI/LTQ-Orbitrap) m/z: $[M + H]^+$ Calcd for $C_8H_5BrF_3OS^+$ 284.9191; Found 284.9192.

The spectra were consistent with the spectrum reported in the literature.[2]

***S*-(trifluoromethyl) 4-iodobenzothioate (3j)**

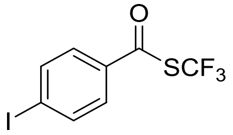

Following the General Procedure with the corresponding carboxylic acid (0.3 mmol). The crude product purified by flash column chromatography, using *n*-pentane/diethyl ether = 30/1 (v/v) as an eluent, to yield the title compound **3j** (75 mg) in 75%.

^{1}H NMR (400 MHz, Chloroform-d): δ 7.95 – 7.87 (m, 2H), 7.63 – 7.55 (m, 2H).

^{13}C NMR (101 MHz, Chloroform-d): δ 182.77, 138.57, 134.42, 134.39, 129.27, 128.71, 126.19, 103.38.

^{19}F NMR (376 MHz, Chloroform-d): δ -39.53.

Physical State: white solid.

HRMS (APPI/LTQ-Orbitrap) m/z: $[M + H]^+$ Calcd for $C_8H_5F_3IOS^+$ 332.9052; Found 332.9059.

***S*-(trifluoromethyl) 2-chlorobenzothioate (3k)**

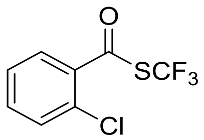

Following the General Procedure with the corresponding carboxylic acid (0.3 mmol). The crude product purified by flash column chromatography, using *n*-pentane/diethyl ether = 30/1 (v/v) as an eluent, to yield the title compound **3k** (44 mg) in 61%.

^{1}H NMR (400 MHz, Chloroform-d): δ 7.73 – 7.64 (m, 1H), 7.56 – 7.48 (m, 2H), 7.45 – 7.35 (m, 1H).

^{13}C NMR (101 MHz, Chloroform-d): δ 182.67, 134.78 (q, *J* = 2.6 Hz), 134.12, 131.91, 131.68, 129.67, 127.60 (q, *J* = 310.9 Hz), 127.28.

^{19}F NMR (376 MHz, Chloroform-d): δ -40.55.

Physical State: yellowish oil.

HRMS (APPI/LTQ-Orbitrap) m/z: $[M + H]^+$ Calcd for $C_8H_5F_3ClOS^+$ 240.9696; Found 240.9697.

The spectra were consistent with the spectrum reported in the literature.[3]

***S*-(trifluoromethyl) 2-bromobenzothioate (3l)**

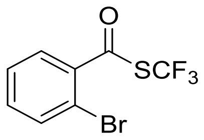

Following the General Procedure with the corresponding carboxylic acid (0.3 mmol). The crude product purified by flash column chromatography, using *n*-pentane/diethyl ether = 30/1 (v/v) as an eluent, to yield the title compound **3l** (64 mg) in 75%.

^{1}H NMR (400 MHz, Chloroform-d): δ 7.79 – 7.67 (m, 1H), 7.67 – 7.58 (m, 1H), 7.48 – 7.40 (m, 2H).

^{13}C NMR (101 MHz, Chloroform-d): δ 183.55 (q, *J* = 1.3 Hz), 136.84 (q, *J* = 2.8 Hz), 134.95, 134.02, 129.53, 127.80, 127.54 (q, *J* = 311.1 Hz), 119.62.

^{19}F NMR (376 MHz, Chloroform-d): δ -40.51.

Physical State: yellowish oil.

HRMS (APPI/LTQ-Orbitrap) m/z: $[M + H]^+$ Calcd for $C_8H_5BrF_3OS^+$ 284.9191; Found 284.9193.

***S*-(trifluoromethyl) 2-iodobenzothioate (3m)**

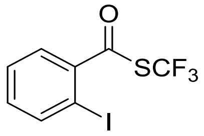

Following the General Procedure with the corresponding carboxylic acid (0.3 mmol). The crude product purified by flash column chromatography, using *n*-pentane/diethyl ether = 30/1 (v/v) as an eluent, to yield the title compound **3m** (71 mg) in 71%.

^{1}H NMR (400 MHz, Chloroform-d): δ 8.01 (d, *J* = 8.0 Hz, 1H), 7.59 (dd, *J* = 7.9, 1.6 Hz, 1H), 7.51 – 7.41 (m, 1H), 7.27 – 7.23 (m, 1H).

^{13}C NMR (101 MHz, Chloroform-d): δ 184.66, 141.86, 139.85 (q, *J* = 2.7 Hz), 134.00, 129.22, 128.48, 127.55 (q, *J* = 311.0 Hz), 91.58.

^{19}F NMR (376 MHz, Chloroform-d): δ -40.46.

Physical State: yellowish oil.

HRMS (APPI/LTQ-Orbitrap) m/z: $[M + H]^+$ Calcd for $C_8H_5F_3IOS^+$ 332.9052; Found 332.9059.

***S*-(trifluoromethyl) 4-(trifluoromethyl)benzothioate (3n)**

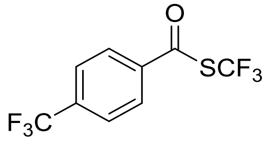

Following the General Procedure with the corresponding carboxylic acid (0.3 mmol). The crude product purified by flash column chromatography, using hexanes/EA = 30/1 (v/v) as an eluent, to yield the title compound **3n** (53 mg) in 65%.

^{1}H NMR (400 MHz, Chloroform-d): δ 8.12 – 7.92 (m, 2H), 7.92 – 7.73 (m, 2H).

^{13}C NMR (101 MHz, Chloroform-d): δ 182.77, 137.95, 136.42 (q, *J* = 33.2 Hz), 128.16, 127.79 (q, *J* = 310.2 Hz), 126.50 (q, *J* = 3.7 Hz), 123.29 (q, *J* = 273.1 Hz).

^{19}F NMR (376 MHz, Chloroform-d): δ -39.60, -63.42.

Physical State: white solid.

HRMS (EI) m/z: $[M - F]^+$ Calcd for 254.9903; Found: 254.9904.

The spectra were consistent with the spectrum reported in the literature.[3]

4-((trifluoromethylthio)carbonyl)benzoate (3o)

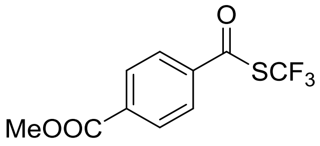

Following the General Procedure with the corresponding carboxylic acid (0.3 mmol). The crude product purified by flash column chromatography, using hexanes/EA = 20/1 (v/v) as an eluent, to yield the title compound **3o** (54 mg) in 68%.

^{1}H NMR (400 MHz, Chloroform-d): δ 8.23 – 8.07 (m, 2H), 7.99 – 7.84 (m, 2H), 3.96 (s, 3H).

^{13}C NMR (101 MHz, Chloroform-d): δ 183.06, 165.69, 138.32 (q, *J* = 2.7 Hz), 135.83, 130.47, 127.89 (d, *J* = 310.1 Hz), 127.70, 52.85.

^{19}F NMR (376 MHz, Chloroform-d): δ -39.65.

Physical State: off-white solid.

HRMS (APPI/LTQ-Orbitrap) m/z: $[M + H]^+$ Calcd for $C_{10}H_8F_3O_3S^+$ 265.0141; Found 265.0140.

The spectra were consistent with the spectrum reported in the literature.[2]

***S*-(trifluoromethyl) 4-((*tert*-butoxycarbonyl)amino)benzothioate (3p)**

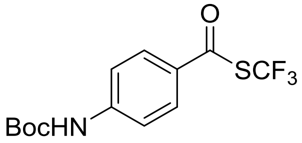

Following the General Procedure with the corresponding carboxylic acid (0.3 mmol). The crude product purified by flash column chromatography, using hexanes/EA = 30/1 (v/v) as an eluent, to yield the title compound **3p** (60 mg) in 62%.

^{1}H NMR (400 MHz, Chloroform-d): δ 7.86 – 7.76 (m, 2H), 7.58 – 7.49 (m, 2H), 6.88 (s, 1H), 1.55 (s, 9H).

^{13}C NMR (101 MHz, Chloroform-d): δ 151.88, 144.83, 129.31, 128.16 (q, *J* = 309.3 Hz), 117.71, 81.82, 28.21.

^{19}F NMR (376 MHz, Chloroform-d): δ -39.43.

Physical State: pale grey solid.

HRMS (APPI/LTQ-Orbitrap) m/z: $[M + H]^+$ Calcd for $C_{13}H_{15}F_3NO_3S^+$ 322.0719; Found 322.0710.

***S*-(trifluoromethyl) 4-(methylthio)benzothioate (3q)**

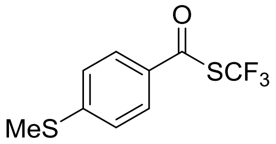

Following the General Procedure with the corresponding carboxylic acid (0.3 mmol). The crude product purified by flash column chromatography, using hexanes/EA = 30/1 (v/v) as an eluent, to yield the title compound **3q** (54 mg) in 72%.

^{1}H NMR (400 MHz, Chloroform-d): δ 7.88 – 7.69 (m, 2H), 7.40 – 7.24 (m, 2H), 2.55 (s, 3H).

^{13}C NMR (101 MHz, Chloroform-d): δ 182.14, 149.30, 131.17 (q, *J* = 2.9 Hz), 128.24 (q, *J* = 309.5 Hz), 128.05, 125.25, 14.73.

^{19}F NMR (376 MHz, Chloroform-d): δ -39.43.

Physical State: white solid.

HRMS (APPI/LTQ-Orbitrap) m/z: $[M + H]^+$ Calcd for $C_9H_8F_3OS_2^+$ 252.9963; Found 252.9958.

***S*-(trifluoromethyl) 4-(4,4,5,5-tetramethyl-1,3,2-dioxaborolan-2-yl)benzothioate (3r)**

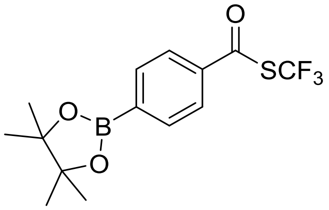

Following the General Procedure with the corresponding carboxylic acid (0.2 mmol). The crude product purified by flash column chromatography, using *n*-pentane/diethyl ether = 30/1 (v/v) as an eluent, to yield the title compound **3r** (40 mg) in 60%.

^{1}H NMR (400 MHz, Chloroform-d): δ 8.04 – 7.90 (m, 2H), 7.90 – 7.79 (m, 2H), 1.38 (s, 12H).

^{13}C NMR (101 MHz, Chloroform-d): δ 183.69, 137.06 (q, *J* = 2.6 Hz), 135.52, 128.16 (q, *J* = 309.6 Hz), 126.69, 84.65, 25.03.

^{19}F NMR (376 MHz, Chloroform-d): δ -39.68.

Physical State: yellow oil.

HRMS (APPI/LTQ-Orbitrap) m/z: $[M + H]^+$ Calcd for $C_{14}H_{17}BF_3O_3S^+$ 333.0938; Found 333.0939.

***S*-(trifluoromethyl) 4-(allyloxy)benzothioate (3s)**

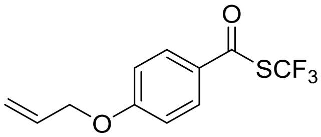

Following the General Procedure with the corresponding carboxylic acid (0.3 mmol). The crude product purified by flash column chromatography, using hexanes/EA = 30/1 (v/v) as an eluent, to yield the title compound **3s** (59 mg) in 75%.

^{1}H NMR (400 MHz, Chloroform-d): δ 7.91 – 7.71 (m, 2H), 7.04 – 6.91 (m, 2H), 6.14 – 5.92 (m, 1H), 5.47 – 5.27 (m, 2H), 4.62 (dt, *J* = 5.3, 1.6 Hz, 2H).

^{13}C NMR (101 MHz, Chloroform-d): δ 181.57, 164.20, 132.12, 130.19, 128.34 (q, *J* = 309.2 Hz), 128.01 (q, *J* = 2.9 Hz), 118.69, 115.23, 69.27.

^{19}F NMR (376 MHz, Chloroform-d): δ -39.43.

Physical State: white solid.

HRMS (APPI/LTQ-Orbitrap) m/z: $[M + H]^+$ Calcd for $C_{11}H_{10}F_3O_2S^+$ 263.0348; Found 263.0347.

***S*-(trifluoromethyl) 4-(allyloxy)benzothioate (3t)**

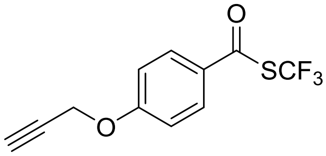

Following the General Procedure with the corresponding carboxylic acid (0.3 mmol). The crude product purified by flash column chromatography, using hexanes/EA = 30/1 (v/v) as an eluent, to yield the title compound **3t** (53 mg) in 68%.

^{1}H NMR (400 MHz, Chloroform-d): δ 7.89 – 7.80 (m, 2H), 7.10 – 7.01 (m, 2H), 4.78 (d, *J* = 2.4 Hz, 2H), 2.57 (t, *J* = 2.4 Hz, 1H).

^{13}C NMR (101 MHz, Chloroform-d): δ 181.69, 162.94, 130.66, 130.13, 128.71 (q, *J* = 2.8 Hz), 128.27 (q, *J* = 309.3 Hz), 115.44, 56.20.

^{19}F NMR (376 MHz, Chloroform-d): δ -39.44.

Physical State: white solid.

HRMS (APPI/LTQ-Orbitrap) m/z: $[M + H]^+$ Calcd for $C_{11}H_8F_3O_2S^+$ 261.0192; Found 261.0190.

***S*-(trifluoromethyl) benzo[1,3]dioxole-5-carbothioate (3u)**

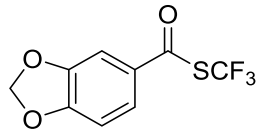

Following the General Procedure with the corresponding carboxylic acid (0.3 mmol). The crude product purified by flash column chromatography, using *n*-pentane/diethyl ether = 20/1 (v/v) as an eluent, to yield the title compound **3u** (61 mg) in 81%.

^{1}H NMR (400 MHz, Chloroform-d): δ 7.46 (ddd, *J* = 8.2, 1.9, 0.7 Hz, 1H), 7.28 (dd, *J* = 12.7, 1.2 Hz, 1H), 6.88 (d, *J* = 8.2 Hz, 1H), 6.09 (s, 2H).

^{13}C NMR (101 MHz, Chloroform-d): δ 181.40, 153.66, 148.81, 129.64 (q, *J* = 2.8 Hz), 128.20 (q, *J* = 309.3 Hz), 124.60, 108.58, 107.39, 102.61.

^{19}F NMR (376 MHz, Chloroform-d): δ -39.58.

Physical State: off-white solid.

HRMS (APPI/LTQ-Orbitrap) m/z: $[M + H]^+$ Calcd for $C_9H_6F_3O_3S^+$ 250.9984; Found 250.9983.

***S*-(trifluoromethyl) naphthalene-2-carbothioate (3v)**

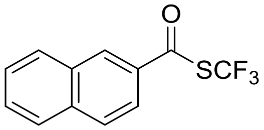

Following the General Procedure with the corresponding carboxylic acid (0.3 mmol). The crude product purified by flash column chromatography, using *n*-pentane/diethyl ether = 20/1 (v/v) as an eluent, to yield the title compound **3v** (68 mg) in 89%.

^{1}H NMR (400 MHz, Chloroform-d): δ 8.39 (s, 1H), 8.05 – 7.76 (m, 4H), 7.64 (m, 2H).

^{13}C NMR (101 MHz, Chloroform-d): δ 183.14, 136.37, 132.40 (q, *J* = 2.8 Hz), 132.25, 129.86, 129.72, 129.59, 129.28, 128.13 (q, *J* = 309.6 Hz), 127.97, 127.56, 122.57.

^{19}F NMR (376 MHz, Chloroform-d): δ -39.50.

Physical State: white solid.

HRMS (APPI/LTQ-Orbitrap) m/z: $[M + H]^+$ Calcd for $C_{12}H_8F_3OS^+$ 257.0243; Found 257.0244.

The spectra were consistent with the spectrum reported in the literature.[2]

***S*-(trifluoromethyl) quinoline-8-carbothioate (3w)**

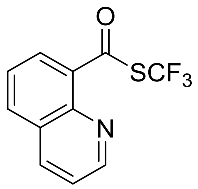

Following the General Procedure with the corresponding carboxylic acid (0.3 mmol). The crude product purified by flash column chromatography, using hexanes/EA = 20/1 (v/v) as an eluent, to yield the title compound **3w** (51 mg) in 66%.

^{1}H NMR (400 MHz, Chloroform-d): δ 9.01 (dd, *J* = 4.2, 1.8 Hz, 1H), 8.44 (dd, *J* = 7.4, 1.5 Hz, 1H), 8.28 (dd, *J* = 8.3, 1.8 Hz, 1H), 8.10 (dd, *J* = 8.2, 1.5 Hz, 1H), 7.68 (t, *J* = 7.7 Hz, 1H), 7.56 (dd, *J* = 8.4, 4.2 Hz, 1H).

^{13}C NMR (101 MHz, Chloroform-d): δ 149.95, 145.01, 136.90, 134.32, 132.10 (q, *J* = 9.9 Hz), 131.92, 128.52 (q, *J* = 12.3 Hz), 128.29 (q, *J* = 313.1 Hz), 128.06, 126.34, 122.14.

^{19}F NMR (376 MHz, Chloroform-d): δ -43.85.

Physical State: off-white solid.

HRMS (APPI/LTQ-Orbitrap) m/z: $[M + H]^+$ Calcd for $C_{11}H_7F_3NOS^+$ 258.0195; Found 258.019.

***S*-(trifluoromethyl) benzothiophene-5-carbothioate (3x)**

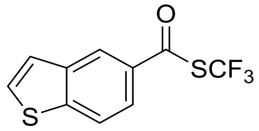

Following the General Procedure with the corresponding carboxylic acid (0.3 mmol). The crude product purified by flash column chromatography, using hexanes/EA = 20/1 (v/v) as an eluent, to yield the title compound **3x** (56 mg) in 71%.

^{1}H NMR (400 MHz, Chloroform-d): δ 8.33 (d, *J* = 1.8 Hz, 1H), 7.97 (dd, *J* = 8.4, 0.9 Hz, 1H), 7.79 (dd, *J* = 8.5, 1.8 Hz, 1H), 7.60 (d, *J* = 5.5 Hz, 1H), 7.46 (dd, *J* = 5.5, 0.8 Hz, 1H).

^{13}C NMR (101 MHz, Chloroform-d): δ 183.07, 146.08, 139.44, 131.61 (q, *J* = 2.8 Hz), 129.03, 128.11 (q, *J* = 309.5 Hz), 124.52, 123.71, 123.28, 122.25.

^{19}F NMR (376 MHz, Chloroform-d): δ -39.51.

Physical State: yellowish solid.

HRMS (APPI/LTQ-Orbitrap) m/z: $[M + H]^+$ Calcd for $C_{10}H_6F_3OS_2^+$ 262.9807; Found 262.9806.

***S*-(trifluoromethyl) 2-(4-methoxyphenyl)ethanethioate (4a)**

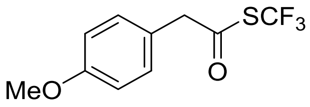

Following the General Procedure with the corresponding carboxylic acid (0.3 mmol). The crude product purified by flash column chromatography, using hexanes/EA = 30/1 (v/v) as an eluent, to yield the title compound **4a** (51 mg) in 68%.

^{1}H NMR (400 MHz, Chloroform-d): δ 7.23 – 7.16 (m, 2H), 6.97 – 6.85 (m, 2H), 3.82 (s, 3H), 3.81 (s, 2H).

^{13}C NMR (101 MHz, Chloroform-d): δ 190.03, 159.99, 131.52, 127.93 (q, *J* = 310.2 Hz), 122.62, 114.68, 55.43, 50.12 (q, *J* = 2.8 Hz).

^{19}F NMR (376 MHz, Chloroform-d): δ -40.69.

Physical State: colorless oil.

HRMS (nanochip-ESI/LTQ-Orbitrap) m/z: $[M]^+$ Calcd for $C_{10}H_9F_3O_2S^+$ 250.0270; Found 250.0267.

***S*-(trifluoromethyl) (1*R*,2*R*)-2-phenylcyclopropane-1-carbothioate (4b)**

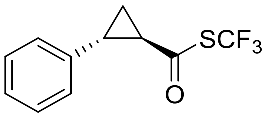

Following the General Procedure with the corresponding carboxylic acid (0.3 mmol). The crude product purified by flash column chromatography, using *n*-pentane/diethyl ether = 30/1 (v/v) as an eluent, to yield the title compound **4b** (48 mg) in 65%.

^{1}H NMR (400 MHz, Chloroform-*d*): δ 7.41 – 7.23 (m, 3H), 7.21 – 7.09 (m, 2H), 2.86 (ddd, *J* = 9.5, 7.1, 4.0 Hz, 1H), 2.17 (dt, *J* = 8.6, 4.6 Hz, 1H), 1.94 (dt, *J* = 9.6, 5.0 Hz, 1H), 1.65 (td, *J* = 7.6, 4.8 Hz, 1H).

^{13}C NMR (101 MHz, Chloroform-*d*): δ 188.73, 138.41, 128.86, 127.72 (q, *J* = 310.0 Hz), 127.41, 126.42, 33.92 (q, *J* = 3.7 Hz), 30.30, 19.79.

^{19}F NMR (376 MHz, Chloroform-*d*): δ -40.02.

Physical State: pale yellow oil.

HRMS (APPI/LTQ-Orbitrap) m/z: $[M]^+$ Calcd for $C_{11}H_9F_3OS^+$ 246.0321; Found 246.0317.

***S*-(trifluoromethyl) 1-(4-methylphenyl)cyclopropane-1-carbothioate (4c)**

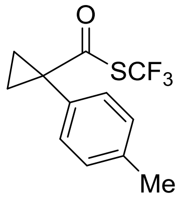

Following the General Procedure with the corresponding carboxylic acid (0.3 mmol). The crude product purified by flash column chromatography, using *n*-pentane/diethyl ether =30/1 (v/v) as an eluent, to yield the title compound **4c** (53 mg) in 68%.

^{1}H NMR (400 MHz, Chloroform-*d*): δ 7.34 (d, *J* = 7.7 Hz, 2H), 7.21 (d, *J* = 7.7 Hz, 2H), 2.40 (s, 3H), 1.83 (q, *J* = 3.6 Hz, 2H), 1.37 (q, *J* = 4.1 Hz, 2H).

^{13}C NMR (101 MHz, Chloroform-*d*): δ 193.73, 139.75, 132.97, 132.42, 129.68, 128.07 (q, *J* = 310.1 Hz), 38.23 (q, *J* = 3.2 Hz), 21.45, 20.48.

^{19}F NMR (376 MHz, Chloroform-*d*): δ -41.84.

Physical State: yellowish oil.

HRMS (APPI/LTQ-Orbitrap) m/z: $[M]^+$ Calcd for $C_{12}H_{11}F_3OS^+$ 260.0477; Found 260.0478.

***S*-(trifluoromethyl) 1-(4-chlorophenyl)cyclopropane-1-carbothioate (4d)**

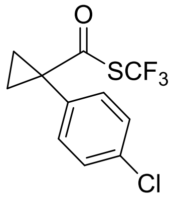

Following the General Procedure with the corresponding carboxylic acid (0.3 mmol). The crude product purified by flash column chromatography, using *n*-pentane/diethyl ether =30/1 (v/v) as an eluent, to yield the title compound **4d** (58 mg) in 69%.

^{1}H NMR (400 MHz, Chloroform-*d*): δ 7.38 (d, *J* = 2.4 Hz, 4H), 1.85 (q, *J* = 4.1 Hz, 2H), 1.36 (q, *J* = 4.1 Hz, 2H).

^{13}C NMR (101 MHz, Chloroform-*d*): δ 192.73, 135.79, 134.53, 133.80, 129.27, 127.86 (q, *J* = 310.2 Hz), 38.02 (q, *J* = 3.3 Hz), 20.48.

^{19}F NMR (376 MHz, Chloroform-*d*): δ -41.61.

Physical State: yellowish oil.

HRMS (APPI/LTQ-Orbitrap) m/z: $[M]^+$ Calcd for $C_{11}H_8ClF_3OS^+$ 279.9931; Found 279.9932.

S-(trifluoromethyl) 1-(4-methoxyphenyl)cyclopropane-1-carbothioate (4e)

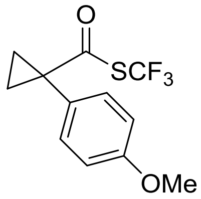

Following the General Procedure with the corresponding carboxylic acid (0.3 mmol). The crude product purified by flash column chromatography, using *n*-pentane/diethyl ether =30/1 (v/v) as an eluent, to yield the title compound **4e** (62 mg) in 75%.

^{1}H NMR (400 MHz, Chloroform-*d*): δ 7.36 (d, *J* = 8.4 Hz, 2H), 6.91 (d, *J* = 8.4 Hz, 2H), 3.84 (s, 3H), 1.81 (q, *J* = 3.9 Hz, 2H), 1.35 (q, *J* = 4.0 Hz, 2H).

^{13}C NMR (101 MHz, Chloroform-*d*): δ 194.05, 160.60, 133.82, 128.10 (q, *J* = 310.1 Hz), 127.80, 114.30, 55.46, 37.80 (q, *J* = 3.3 Hz), 20.64.

^{19}F NMR (376 MHz, Chloroform-*d*): δ -41.95.

Physical State: reddish oil.

HRMS (APPI/LTQ-Orbitrap) m/z: $[M]^+$ Calcd for $C_{12}H_{11}F_3O_2S^+$ 276.0426; Found 276.0429.

S-(trifluoromethyl) (*E*)-3-phenylprop-2-enethioate (5a)

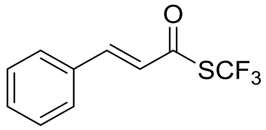

Following the General Procedure with the corresponding carboxylic acid (0.3 mmol). The crude product purified by flash column chromatography, using *n*-pentane/diethyl ether =30/1 (v/v) as an eluent, to yield the title compound **5a** (57 mg) in 82%.

^{1}H NMR (400 MHz, Chloroform-d): δ 7.65 (d, *J* = 15.8 Hz, 1H), 7.60 – 7.53 (m, 2H), 7.51 – 7.38 (m, 3H), 6.61 (d, *J* = 15.8 Hz, 1H).

^{13}C NMR (101 MHz, Chloroform-d): δ 180.95, 145.18, 133.14, 131.95, 129.36, 129.01, 128.07 (q, *J* = 309.5 Hz), 122.86 (q, *J* = 3.1 Hz).

^{19}F NMR (376 MHz, Chloroform-d): δ -39.52.

Physical State: colorless oil.

HRMS (APPI/LTQ-Orbitrap) m/z: $[M + H]^+$ Calcd for $C_{10}H_8F_3OS^+$ 233.0242; Found 233.0241.

***S*-(trifluoromethyl) (*E*)-3-(4-fluorophenyl)prop-2-enethioate (5b)**

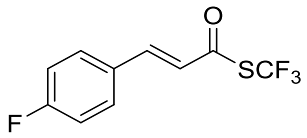

Following the General Procedure with the corresponding carboxylic acid (0.3 mmol). The crude product purified by flash column chromatography, using *n*-pentane/diethyl ether =30/1 (v/v) as an eluent, to yield the title compound **5b** (53 mg) in 70%.

^{1}H NMR (400 MHz, Chloroform-d): δ 7.71 – 7.44 (m, 3H), 7.12 (t, *J* = 8.4 Hz, 2H), 6.53 (d, *J* = 15.8 Hz, 1H).

^{13}C NMR (101 MHz, Chloroform-d): δ 180.77, 164.90 (d, *J* = 254.3 Hz), 143.75, 131.08 (d, *J* = 8.8 Hz), 129.42 (d, *J* = 3.4 Hz), 128.02 (q, *J* = 309.5 Hz), 122.86 – 122.11 (m), 116.64 (d, *J* = 22.2 Hz).

^{19}F NMR (376 MHz, Chloroform-d): δ -39.53, -106.55.

Physical State: yellowish solid.

HRMS (APPI/LTQ-Orbitrap) m/z: $[M + H]^+$ Calcd for $C_{10}H_7F_4OS^+$ 251.0148; Found 251.0149.

***S*-(trifluoromethyl) (*E*)-3-(4-chlorophenyl)prop-2-enethioate (5c)**

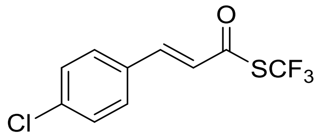

Following the General Procedure with the corresponding carboxylic acid (0.3 mmol). The crude product purified by flash column chromatography, using *n*-pentane/diethyl ether =30/1 (v/v) as an eluent, to yield the title compound **5c** (57 mg) in 71%.

^{1}H NMR (400 MHz, Chloroform-d): δ 7.59 (d, *J* = 15.8 Hz, 1H), 7.52 – 7.46 (m, 2H), 7.44 – 7.37 (m, 2H), 6.57 (d, *J* = 15.8 Hz, 1H).

^{13}C NMR (101 MHz, Chloroform-d): δ 180.77 (q, *J* = 1.6 Hz), 143.55, 138.04, 131.60, 130.11, 129.68, 127.97 (q, *J* = 309.6 Hz), 123.19 (q, *J* = 3.1 Hz).

^{19}F NMR (376 MHz, Chloroform-d): δ -39.52.

Physical State: white solid.

HRMS (APPI/LTQ-Orbitrap) m/z: $[M + H]^+$ Calcd for $C_{10}H_7ClF_3OS^+$ 266.9853; Found 266.9853.

***S*-(trifluoromethyl) (*E*)-3-(4-bromophenyl)prop-2-enethioate (5d)**

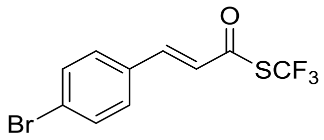

Following the General Procedure with the corresponding carboxylic acid (0.2 mmol). The crude product purified by flash column chromatography, using hexanes/EA = 30/1 (v/v) as an eluent, to yield the title compound **5d** (51 mg) in 82%.

^{1}H NMR (400 MHz, Chloroform-d): δ 7.64 – 7.53 (m, 3H), 7.42 (d, *J* = 8.5 Hz, 2H), 6.59 (d, *J* = 15.8 Hz, 1H).

^{13}C NMR (101 MHz, Chloroform-d): δ 180.80, 143.64, 132.68, 132.04, 130.26, 127.95 (q, *J* = 309.7 Hz), 126.51, 123.30 (q, *J* = 3.2 Hz).

^{19}F NMR (376 MHz, Chloroform-d): δ -39.52.

Physical State: white solid.

HRMS (APPI/LTQ-Orbitrap) m/z: $[M + H]^+$ Calcd for $C_{10}H_7BrF_3OS^+$ 310.9348; Found 310.9350.

***S*-(trifluoromethyl) (*E*)-3-(2-(trifluoromethyl)phenyl)prop-2-enethioate (5e)**

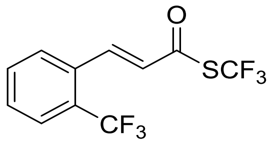

Following the General Procedure with the corresponding carboxylic acid (0.3 mmol). The crude product purified by flash column chromatography, using *n*-pentane/diethyl ether =30/1 (v/v) as an eluent, to yield the title compound **5e** (63 mg) in 70%.

^{1}H NMR (400 MHz, Chloroform-d): δ 8.03 (dd, *J* = 15.7, 2.1 Hz, 1H), 7.73 (dd, *J* = 14.3, 7.7 Hz, 2H), 7.59 (dt, *J* = 22.1, 7.5 Hz, 2H), 6.58 (d, *J* = 15.6 Hz, 1H).

^{13}C NMR (101 MHz, Chloroform-d): δ 180.67, 140.32 (q, *J* = 2.2 Hz), 132.33 (q, *J* = 1.1 Hz), 131.78 (q, *J* = 1.6 Hz), 130.91, 129.64 (q, *J* = 30.6 Hz), 128.00, 127.73 (q, *J* = 309.8 Hz), 126.82 – 126.32 (m), 123.70 (q, *J* = 274.1 Hz).

^{19}F NMR (376 MHz, Chloroform-d): δ -39.62, -58.73.

Physical State: yellowish oil.

HRMS (APPI/LTQ-Orbitrap) m/z: $[M + H]^+$ Calcd for $C_{11}H_7F_6OS^+$ 301.0116; Found 301.0116.

***S*-(trifluoromethyl) (*E*)-3-(4-methoxyphenyl)prop-2-enethioate (5f)**

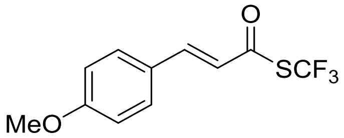

Following the General Procedure with the corresponding carboxylic acid (0.3 mmol). The crude product purified by flash column chromatography, using *n*-pentane/diethyl ether =20/1 (v/v) as an eluent, to yield the title compound **5f** (72 mg) in 91%.

^{1}H NMR (400 MHz, Chloroform-d): δ 7.60 (d, *J* = 15.7 Hz, 1H), 7.55 – 7.48 (m, 2H), 6.96 – 6.88 (m, 2H), 6.46 (d, *J* = 15.7 Hz, 1H), 3.86 (s, 3H).

^{13}C NMR (101 MHz, Chloroform-d): δ 180.68, 162.84, 145.00, 130.99, 128.22 (q, *J* = 309.2 Hz), 125.81, 120.31 (q, *J* = 3.1 Hz), 114.84, 55.62.

^{19}F NMR (376 MHz, Chloroform-d): δ -39.45.

Physical State: off-white solid.

HRMS (APPI/LTQ-Orbitrap) m/z: $[M + H]^+$ Calcd for $C_{11}H_{10}F_3O_2S^+$ 263.0348; Found 263.0352.

***S*-(trifluoromethyl) (*E*)-3-(4-(dimethylamino)phenyl)prop-2-enethioate (5g)**

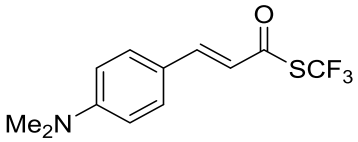

Following the General Procedure with the corresponding carboxylic acid (0.3 mmol). The crude product purified by flash column chromatography, using *n*-pentane/diethyl ether =20/1 (v/v) as an eluent, to yield the title compound **5g** (49 mg) in 59%.

^{1}H NMR (400 MHz, Chloroform-d): δ 7.58 (d, *J* = 15.4 Hz, 1H), 7.51 – 7.39 (m, 2H), 6.73 – 6.62 (m, 2H), 6.36 (d, *J* = 15.4 Hz, 1H), 3.06 (s, 6H).

^{13}C NMR (101 MHz, Chloroform-d): δ 180.20, 152.91, 146.10, 131.27, 128.52 (q, *J* = 308.8 Hz), 120.69, 116.90 (q, *J* = 3.3 Hz), 111.92, 40.21.

^{19}F NMR (376 MHz, Chloroform-d): δ -39.29.

Physical State: yellowish solid.

HRMS (APPI/LTQ-Orbitrap) m/z: $[M + H]^+$ Calcd for $C_{12}H_{13}F_3NOS^+$ 276.0664; Found 276.0665.

***S*-(trifluoromethyl) (E)-3-(4-(trifluoromethoxy)phenyl)prop-2-enethioate (5h)**

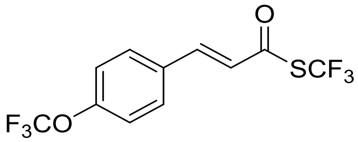

Following the General Procedure with the corresponding carboxylic acid (0.3 mmol). The crude product purified by flash column chromatography, using *n*-pentane/diethyl ether =20/1 (v/v) as an eluent, to yield the title compound **5h** (71 mg) in 75%.

^{1}H NMR (400 MHz, Chloroform-d): δ 7.64 (d, *J* = 15.8 Hz, 1H), 7.55 – 7.45 (m, 2H), 7.42 (s, 1H), 7.38 – 7.30 (m, 1H), 6.64 (d, *J* = 15.8 Hz, 1H).

^{13}C NMR (101 MHz, Chloroform-d): δ 180.61, 149.80 (d, *J* = 2.1 Hz), 142.94, 135.05, 130.69, 127.75 (q, *J* = 309.8 Hz), 127.16, 124.20 (q, *J* = 3.1 Hz), 123.82, 120.71, 120.37 (q, *J* = 258.2 Hz).

^{19}F NMR (376 MHz, Chloroform-d): δ -39.60, -57.89.

Physical State: colorless oil.

HRMS (APPI/LTQ-Orbitrap) m/z: $[M + H]^+$ Calcd for $C_{11}H_7F_6O_2S^+$ 317.0066; Found 317.0069.

***S*-(trifluoromethyl) 6-(3-(adamantan-1-yl)-4-methoxyphenyl)naphthalene-2-carbothioate (6a)**

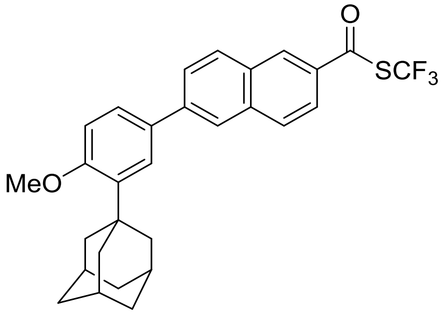

Following the General Procedure with the corresponding carboxylic acid (0.2 mmol). The crude product purified by flash column chromatography, using hexanes/EA = 30/1 (v/v) as an eluent, to yield the title compound **6a** (90 mg) in 91%.

^{1}H NMR (400 MHz, Chloroform-*d*): δ 8.41 (s, 1H), 8.07 – 7.99 (m, 2H), 7.97 (d, *J* = 8.7 Hz, 1H), 7.87 (ddd, *J* = 8.6, 4.5, 1.8 Hz, 2H), 7.61 (d, *J* = 2.4 Hz, 1H), 7.56 (dd, *J* = 8.4, 2.3 Hz, 1H), 7.01 (d, *J* = 8.5 Hz, 1H), 2.23 – 2.07 (m, 9H), 1.81 (d, *J* = 3.0 Hz, 6H).

^{13}C NMR (101 MHz, Chloroform-*d*): 182.97, 159.23, 142.80, 139.17, 136.89, 132.00, 131.90 (q, *J* = 2.6 Hz), 130.96, 130.10, 129.72, 129.28, 128.18 (q, *J* = 310.6 Hz), 127.34, 126.01, 125.83, 124.77, 122.97, 112.16, 55.19, 40.61, 37.25, 37.12, 29.11.

^{19}F NMR (376 MHz, Chloroform-*d*): δ -39.46.

Physical State: white solid.

HRMS (APPI/LTQ-Orbitrap) $[M]^+$ Calcd for $C_{29}H_{27}F_3O_2S^+$ 496.1678; Found 496.1672.

***S*-(trifluoromethyl) 4-(*N,N*-dipropylsulfamoyl)benzothioate (6b)**

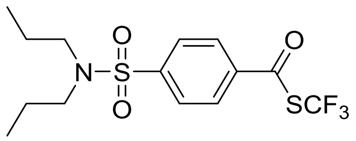

Following the General Procedure with the corresponding carboxylic acid (0.3 mmol). The crude product purified by flash column chromatography, using hexanes/EA = 20/1 (v/v) as an eluent, to yield the title compound **6b** (51 mg) in 46%.

^{1}H NMR (400 MHz, Methylene Chloride-d_2): δ 8.12 – 7.91 (m, 4H), 3.19 – 3.05 (t, *J* = 7.4 Hz, 4H), 1.57 (h, *J* = 7.4 Hz, 4H), 0.90 (t, *J* = 7.4 Hz, 6H).

^{13}C NMR (101 MHz, Methylene Chloride-d_2): δ 146.29, 137.51 (q, *J* = 2.7 Hz), 132.15 (q, *J* = 10.8 Hz), 128.48 (q, *J* = 12.5 Hz), 128.28, 127.83 (q, *J* = 309.9 Hz), 127.73, 49.90, 21.88, 10.86.

^{19}F NMR (376 MHz, Methylene Chloride-d_2): δ -40.18.

Physical State: white solid.

HRMS (APPI/LTQ-Orbitrap) m/z: $[M + H]^+$ Calcd for $C_{14}H_{19}F_3NO_3S_2^+$ 370.0753; Found 370.0752.

(10S,13R,14R,17R)-4,4,10,13,14-pentamethyl-17-((R)-6-methylhept-5-en-2-yl)-2,3,4,5,6,7,10,11,12,13,14,15,16,17-tetradecahydro-1H-cyclopenta[a]phenanthren-3-yl 3-(((trifluoromethyl)thio)carbonyl)benzoate (6c)

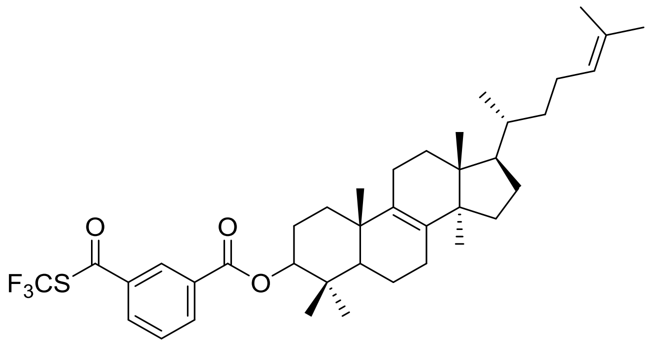

Following the General Procedure with the corresponding carboxylic acid (0.2 mmol). The crude product purified by flash column chromatography, using hexanes/EA = 20/1 (v/v) as an eluent, to yield the title compound **6c** (116 mg) in 88% (mixture of 2 diastereoisomers).

^{1}H NMR (400 MHz, Chloroform-*d*): δ 146.29, 137.51 (q, *J* = 2.7 Hz), 132.15 (q, *J* = 10.8 Hz), 128.48 (q, *J* = 12.5 Hz), 128.28, 127.83 (q, *J* = 309.9 Hz), 127.73, 49.90, 21.88, 10.86.

^{13}C NMR (101 MHz, Chloroform-*d*): δ 182.74, 164.57, 135.62, 134.68, 134.66, 134.13, 132.31, 131.25, 130.90, 129.47, 128.67, 125.25, 82.66, 50.59, 50.52, 50.41, 49.84, 44.51, 44.49, 39.54, 38.24, 36.96, 36.50, 36.37, 36.28, 35.28, 30.99, 30.85, 29.72, 28.23, 28.22, 28.14, 28.02, 26.39, 25.74, 24.94, 24.31, 24.29, 24.25, 24.13, 22.85, 22.56, 21.07, 19.22, 18.74, 18.66, 18.17, 17.65, 16.86, 15.78.

^{19}F NMR (376 MHz, Chloroform-*d*): δ -39.55.

Physical State: white solid.

HRMS (APPI/LTQ-Orbitrap) m/z: $[M]^+$ Calcd for $C_{39}H_{53}F_3O_3S^+$ 658.3662; Found 658.3661.

***S*-(trifluoromethyl) 4-(((1*R*,2*S*,5*R*)-2-isopropyl-5-methylcyclohexyl)oxy)benzothioate (6d)**

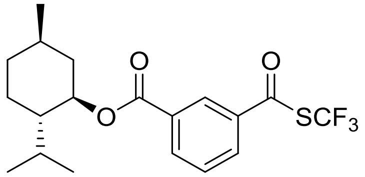

Following the General Procedure with the corresponding carboxylic acid (0.2 mmol). The crude product purified by flash column chromatography, using hexanes/EA = 30/1 (v/v) as an eluent, to yield the title compound **6d** (67 mg) in 86%.

^{1}H NMR (400 MHz, Chloroform-d): δ 8.49 (t, *J* = 1.8 Hz, 1H), 8.33 (dt, *J* = 7.8, 1.4 Hz, 1H), 8.03 (ddd, *J* = 7.9, 2.0, 1.2 Hz, 1H), 7.61 (t, *J* = 7.8 Hz, 1H), 4.98 (td, *J* = 10.9, 4.4 Hz, 1H), 2.16 – 2.08 (m, 1H), 1.92 (pd, *J* = 7.0, 2.8 Hz, 1H), 1.78 – 1.71 (m, 2H), 1.62 – 1.55 (m, 2H), 1.18 – 1.09 (m, 2H), 0.93 (dd, *J* = 6.8, 5.3 Hz, 6H), 0.80 (d, *J* = 6.9 Hz, 3H).

^{13}C NMR (101 MHz, Chloroform-d): δ 182.98, 164.59, 135.82, 135.52 (q, *J* = 2.8 Hz), 132.32, 131.41, 129.58, 128.80, 127.98 (q, *J* = 309.9 Hz), 76.03, 47.33, 41.03, 34.36, 31.62, 26.76, 23.80, 22.15, 20.87, 16.70.

^{19}F NMR (376 MHz, Chloroform-d): δ -39.58.

Physical State: white solid.

HRMS (APPI/LTQ-Orbitrap) m/z: $[M+H]^+$ Calcd for $C_{19}H_{24}F_3O_3S^+$ 389.1393; Found 389.1394.

***S*-(trifluoromethyl) (2*E*,4*E*)-5-(benzo[1,3]dioxol-5-yl)penta-2,4-dienethioate (6e)**

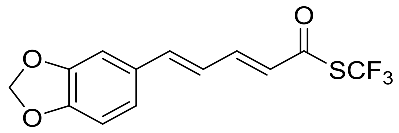

Following the General Procedure with the corresponding carboxylic acid (0.2 mmol). The crude product purified by flash column chromatography, using *n*-pentane/diethyl ether =20/1 (v/v) as an eluent, to yield the title compound **6e** (43 mg) in 71%.

^{1}H NMR (400 MHz, Chloroform-d): δ 7.38 (dd, *J* = 15.0, 11.1 Hz, 1H), 7.02 – 6.94 (m, 3H), 6.81 (d, *J* = 8.0 Hz, 1H), 6.67 (dd, *J* = 15.4, 11.1 Hz, 1H), 6.10 (d, *J* = 15.0 Hz, 1H), 6.01 (s, 2H).

^{13}C NMR (101 MHz, Chloroform-d): δ 180.46, 149.63, 148.62, 145.39, 144.81, 130.06, 128.21 (q, *J* = 309.2 Hz), 124.57 (q, *J* = 3.1 Hz), 124.19, 123.43, 108.83, 106.17, 101.76.

^{19}F NMR (376 MHz, Chloroform-d): δ -39.42.

Physical State: yellowish solid.

HRMS (APPI/LTQ-Orbitrap) m/z: $[M + H]^+$ Calcd for $C_{13}H_{10}F_3O_3S^+$ 303.0297; Found 303.0296.

***S*-(trifluoromethyl) (*E*)-3-(2,3-dimethoxyphenyl)prop-2-enethioate (6f)**

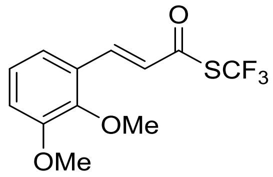

Following the General Procedure with the corresponding carboxylic acid (0.3 mmol). The crude product purified by flash column chromatography, using *n*-pentane/diethyl ether =20/1 (v/v) as an eluent, to yield the title compound **6f** (75 mg) in 86%.

^{1}H NMR (400 MHz, Chloroform-d): δ 7.96 (d, *J* = 16.0 Hz, 1H), 7.14 – 6.98 (m, 3H), 6.67 (d, *J* = 16.0 Hz, 1H), 3.89 (s, 6H).

^{13}C NMR (101 MHz, Chloroform-d): δ 181.31, 153.31, 149.48, 140.29, 128.16 (q, *J* = 309.4 Hz), 127.19, 124.49, 124.07 (q, *J* = 3.1 Hz), 119.78, 115.59, 61.56, 56.04.

^{19}F NMR (376 MHz, Chloroform-d): δ -39.56.

Physical State: yellowish oil.

HRMS (APPI/LTQ-Orbitrap) m/z: $[M + H]^+$ Calcd for $C_{12}H_{12}F_3O_3S^+$ 293.0454; Found 293.0447.

***S*-(trifluoromethyl) 2-(10-oxo-10,11-dihydrodibenzo[*b*,*f*]thiepin-2-yl)propanethioate (6g)**

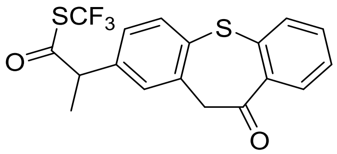

Following the General Procedure with the corresponding carboxylic acid (0.2 mmol). The crude product purified by flash column chromatography, using *n*-pentane/diethyl ether =20/1 (v/v) as an eluent, to yield the title compound **6g** (50 mg) in 66%.

^{1}H NMR (400 MHz, Chloroform-d): δ 8.21 (dd, *J* = 8.0, 1.7 Hz, 1H), 7.66 (d, *J* = 8.0 Hz, 1H), 7.60 (dd, *J* = 7.9, 1.3 Hz, 1H), 7.44 (ddd, *J* = 7.9, 7.2, 1.7 Hz, 1H), 7.39 – 7.30 (m, 2H), 7.13 (dd, *J* = 8.0, 2.0 Hz, 1H), 4.38 (d, *J* = 1.9 Hz, 2H), 3.86 (q, *J* = 7.1 Hz, 1H), 1.55 (d, *J* = 7.1 Hz, 3H).

^{13}C NMR (101 MHz, Chloroform-*d*) δ 191.83, 191.06, 139.88, 139.38, 138.76, 136.19, 135.11, 132.80, 132.13, 131.70, 131.01, 129.37, 127.70 (q, *J* = 310.5 Hz), 127.14, 127.10, 54.80 (q, *J* = 2.7 Hz), 51.16, 17.91.

^{19}F NMR (376 MHz, Chloroform-d): δ -40.31.

Physical State: yellowish oil.

HRMS (APPI/LTQ-Orbitrap) m/z: $[M]^+$ Calcd for $C_{18}H_{14}F_3O_2S_2^+$ 383.0382; Found 383.0380.

***S*-(trifluoromethyl) 2-(4-isobutylphenyl)propanethioate (6h)**

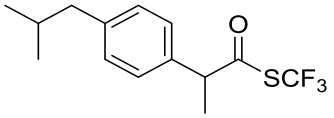

Following the General Procedure with the corresponding carboxylic acid (0.2 mmol). The crude product purified by flash column chromatography, using *n*-pentane/diethyl ether =20/1 (v/v) as an eluent, to yield the title compound **6h** (38 mg) in 66%.

^{1}H NMR (400 MHz, Chloroform-d): δ 7.17 (d, *J* = 2.3 Hz, 4H), 3.84 (q, *J* = 7.1 Hz, 1H), 2.49 (d, *J* = 7.2 Hz, 2H), 1.87 (dp, *J* = 13.6, 7.0 Hz, 1H), 1.57 (d, *J* = 7.1 Hz, 3H), 0.92 (d, *J* = 6.6 Hz, 6H).

^{13}C NMR (101 MHz, Chloroform-d): δ 192.87, 142.32, 134.23, 129.90, 128.24, 127.90 (q, *J* = 310.1 Hz), 54.87 (q, *J* = 2.5 Hz), 45.07, 30.16, 22.35, 17.57.

^{19}F NMR (376 MHz, Chloroform-d): δ -40.59.

Physical State: yellowish oil.

HRMS (APPI/LTQ-Orbitrap) m/z: $[M]^+$ Calcd for $C_{14}H_{17}F_3OS^+$ 290.0947; Found 290.0942.

***S*-(trifluoromethyl) 2-(3-benzoylphenyl)propanethioate (6i)**

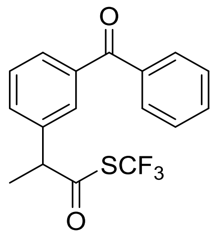

Following the General Procedure with the corresponding carboxylic acid (0.2 mmol). The crude product purified by flash column chromatography, using *n*-pentane/diethyl ether =20/1 (v/v) as an eluent, to yield the title compound **6i** (42 mg) in 62%.

^{1}H NMR (400 MHz, Chloroform-d): δ 7.88 – 7.68 (m, 4H), 7.63 (d, *J* = 7.5 Hz, 1H), 7.57 – 7.45 (m, 4H), 3.95 (q, *J* = 7.1 Hz, 1H), 1.61 (d, *J* = 7.1 Hz, 3H).

^{13}C NMR (101 MHz, Chloroform-d): δ 195.99, 191.93, 138.48, 137.60, 137.17, 132.77, 132.05, 130.21, 130.07, 129.88, 129.22, 128.43, 127.64 (q, *J* = 310.4 Hz), 54.94 (q, *J* = 2.6 Hz), 17.76.

^{19}F NMR (376 MHz, Chloroform-d): δ -40.33.

Physical State: yellowish oil.

HRMS (APPI/LTQ-Orbitrap) m/z: $[M + H]^+$ Calcd for $C_{17}H_{14}F_3O_2S^+$ 339.0661; Found 339.0659.

***S*-(trifluoromethyl) (*S*)-2-(6-methoxynaphthalen-2-yl)propanethioate (6j)**

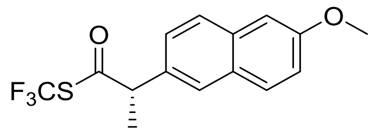

Following the General Procedure with the corresponding carboxylic acid (0.2 mmol). The crude product purified by flash column chromatography, using *n*-pentane/diethyl ether =20/1 (v/v) as an eluent, to yield the title compound **6j** (47 mg) in 75%.

^{1}H NMR (400 MHz, Chloroform-d): δ 7.76 (t, *J* = 9.4 Hz, 2H), 7.68 (d, *J* = 1.9 Hz, 1H), 7.33 (dd, *J* = 8.4, 1.8 Hz, 1H), 7.20 (dd, *J* = 8.9, 2.6 Hz, 1H), 7.15 (d, *J* = 2.5 Hz, 1H), 4.00 (q, *J* = 7.1 Hz, 1H), 3.93 (s, 3H), 1.65 (d, *J* = 7.0 Hz, 3H).

^{13}C NMR (101 MHz, Chloroform-d): δ 192.84, 158.29, 134.43, 132.04, 129.43, 128.89, 127.94, 127.81, 127.62 (q, *J* = 255.3 Hz), 126.19, 119.61, 105.70, 55.38, 55.19 (q, *J* = 2.5 Hz), 17.59.

^{19}F NMR (376 MHz, Chloroform-d): δ -40.55.

Physical State: white solide.

HRMS (APPI/LTQ-Orbitrap) m/z: $[M]^+$ Calcd for $C_{15}H_{13}F_3O_2S^+$ 314.0583; Found 314.0581.

***S*-(trifluoromethyl) 5-(2,5-dimethylphenoxy)-2,2-dimethylpentanethioate (6k)**

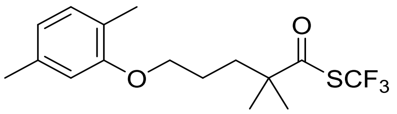

Following the General Procedure with the corresponding carboxylic acid (0.2 mmol). The crude product purified by flash column chromatography, using hexanes/EA = 30/1 (v/v) as an eluent, to yield the title compound **6k** (37 mg) in 56%.

^{1}H NMR (400 MHz, Chloroform-*d*): δ 7.02 (d, *J* = 7.4 Hz, 1H), 6.68 (d, *J* = 7.0 Hz, 1H), 6.61 (s, 1H), 3.95 (p, *J* = 2.8 Hz, 2H), 2.32 (s, 3H), 2.19 (s, 3H), 1.85 – 1.74 (m, 4H), 1.30 (s, 6H).

^{13}C NMR (101 MHz, Chloroform-*d*): δ 197.30, 156.89, 136.67, 130.51, 128.43 (q, *J* = 309.4 Hz), 123.68, 121.02, 112.01, 67.46, 51.38 (q, *J* = 2.2 Hz), 37.20, 24.83, 24.72, 21.52, 15.90.

^{19}F NMR (376 MHz, Chloroform-*d*): δ -40.08.

Physical State: white solid.

HRMS (APPI/LTQ-Orbitrap) m/z: $[M]^+$ Calcd for $C_{16}H_{21}F_3O_2S^+$ 334.1209; Found 334.1209.

***S*-(trifluoromethyl) (1R,4aR,4bR,10aR)-7-isopropyl-1,4a-dimethyl-1,2,3,4,4a,4b,5,6,10,10a-decahydrophenanthrene-1-carbothioate (6l)**

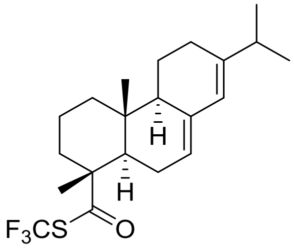

Following the General Procedure with the corresponding carboxylic acid (0.2 mmol). The crude product purified by flash column chromatography, using hexanes/EA = 30/1 (v/v) as an eluent, to yield the title compound **6l** (32 mg) in 42%.

^{1}H NMR (400 MHz, Chloroform-*d*): δ 5.78 (s, 1H), 5.39 – 5.32 (m, 1H), 2.23 (octet, *J* = 8.0, Hz, 1H), 2.09 – 1.62 (m, 12H), 1.32 (s, 3H), 1.23 – 1.10 (m, 3H), 1.03 (d, *J* = 3.4 Hz, 3H), 1.01 (d, *J* = 3.4 Hz, 3H), 0.84 (s, 3H).

^{13}C NMR (101 MHz, Chloroform-*d*): δ 198.61, 145.72, 128.50 (q, *J* = 309.5 Hz), 119.78, 55.86, 50.92, 45.81, 38.00, 37.51, 34.91, 27.42, 25.28, 22.53, 21.42, 20.86, 18.00, 16.87, 14.29.

^{19}F NMR (376 MHz, Chloroform-*d*): δ -40.41.

Physical State: colorless oil.

HRMS (APPI/LTQ-Orbitrap) $[M]^+$ Calcd for $C_{21}H_{29}F_3OS^+$ 386.1886; Found 386.1880.

[1,1'-biphenyl]-4-yl(trifluoromethyl)sulfane (7a)

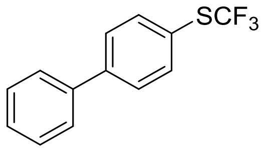

In a nitrogen-filled glovebox, **3a** (1 equiv, 0.1 mmol) was weighed into a 4 mL vial equipped with a 10 µm magnetic stir bar. A pre-mixed solution of Pd catalyst and ligand in toluene (0.3 mL) was added. The vial was connected to a reflux condenser, capped with a rubber septum, and this assembly was removed from the glovebox. An argon balloon was placed on top of the condenser and the reaction mixture was stirred at 130 ºC. After 20 h, the reaction mixture was cooled to room temperature and diluted with diethyl ether. The resulting solution was filtered through a silica plug and concentrated under reduced pressure. The products were purified via flash column chromatography on silica gel (hexanes/Et_2O=30/1, v/v).

^{1}H NMR (400 MHz, Chloroform-*d*): δ 7.79 (dd, J = 16.6, 8.4 Hz, 2H), 7.66 (dd, J = 15.0, 7.9 Hz, 4H), 7.51 (t, J = 7.6 Hz, 2H), 7.47 – 7.41 (m, 1H).

^{13}C NMR (101 MHz, Chloroform-*d*): δ 143.9 (s), 139.7 (s), 136.7 (s), 132.7 (q, J = 308.1 Hz), 129.0 (s), 128.2 (s), 127.2 (s), 123.1 (q, J = 1.8 Hz).

^{19}F NMR (376 MHz, Chloroform-*d*): δ -42.68 (s, 3F).

Physical State: White solid.

HRMS (APPI/LTQ-Orbitrap) $[M+H]^+$ Calcd for $C_{13}H_{10}F_3S^+$ 255.0450; Found 255.0451.

Tables

Table S1. Optimization of trifluoromethylthiolating reagent

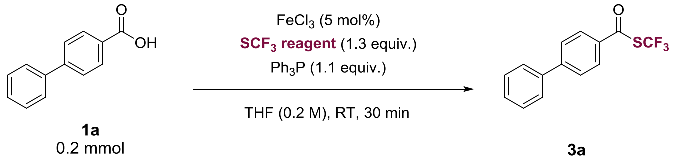

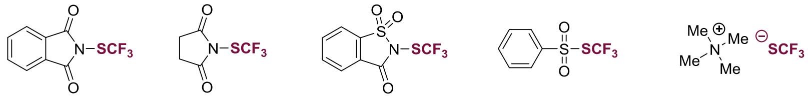

2a (95%) **2b** (19%) **2c** (51%) **2d** (7%) **2e** (0%)

Table S2. Optimization of solvent

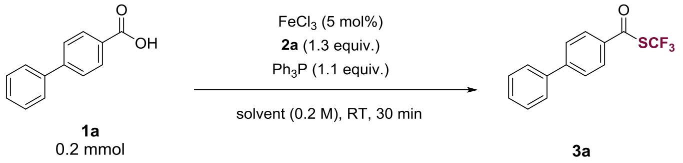

Entry	Slovent	GC yield[a]
1	THF	95%
2	MeCN	36%
3	DCM	29%
4	DMF	18%
5	PhMe	70%
6	$PhCF_3$	81%
7	Et_2O	55%

Table S3. Optimization of Lewis acid

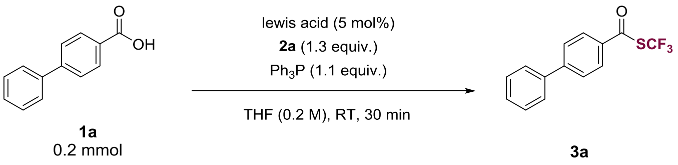

Entry	Lewis acid	GC yield[a]
1	none	40%
2	$Sc(OTf)_4$	71%
3	$SnCl_4$	69%
4	BF_3•OEt (10 mol%)	64%
5	$AlCl_3$	39%
6	$ZnCl_2$	66%
7	$CeCl_3$	41%
8	$Ce(OTf)_4$	45%
9	$Fe(acac)_3$	39%
10	$FeCl_3$•$6H_2O$	71%
11	$FeCl_3$	95%
12	$Fe(OTf)_3$	62%

Table S4. Optimization of external base

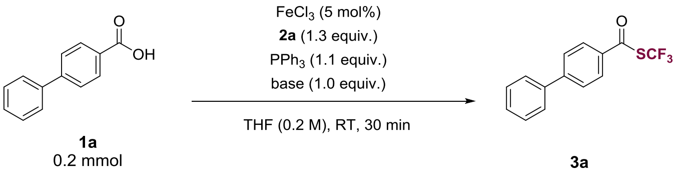

Entry	Base	GC yield[a]
1	$NaHCO_3$	69%
2	$NaHCO_3$ (10 mol%)	78%
3	Cs_2CO_3	40%
4	2,6-lutidine	68%
5	NaH_2PO_4	42%
6	none	95%

Table S5. Optimization of R_3P

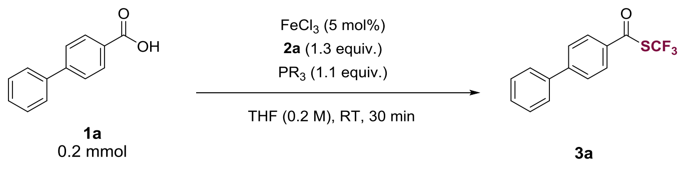

Entry	R_3P	GC yield[a]
1	PPh_3	95%
2	PCy_3	39%
3	$P(OEt)_3$	12%
4	$P(OEt)Ph_2$	35%
5	none	ND

Table S6. Optimization of concentration of reagents

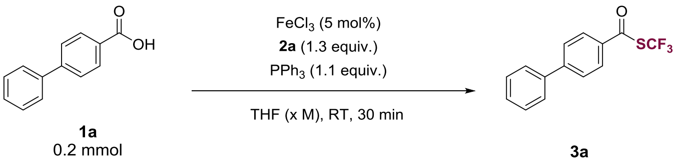

Entry	x	GC yield[a]
1	0.2 M	95%
2	0.1 M	81%
3	0.05 M	69%
4	0.4 M	89%

Table S7. Optimization of SCF_3 reagent/PPh_3 ratio

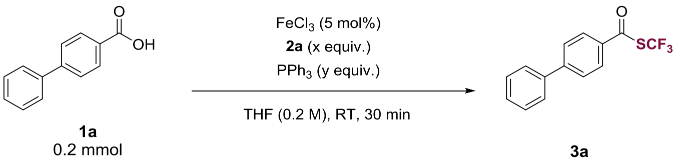

Entry	x/y ratio	GC yield[a]
1	1.3/1.1	95%
2	1.1/1.3	76%
3	1.5/1.3	89%
4	1.8/1.5	80%

Table S8. Unsuccessful examples

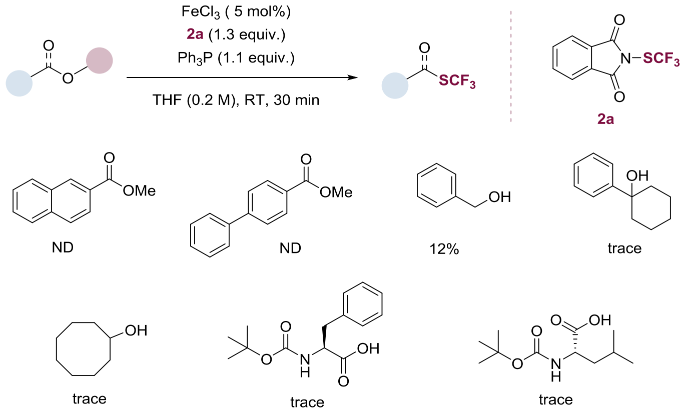

Following the General Procedure with the corresponding substrates (0.2 mmol). Reactions were monitored using GC-MS after 30 minutes, yields mentioned here are GC yields, *n*-dodecane was used as internal standard. ND (not detected).

Figures

Figure S1. NMR spectra of 3a

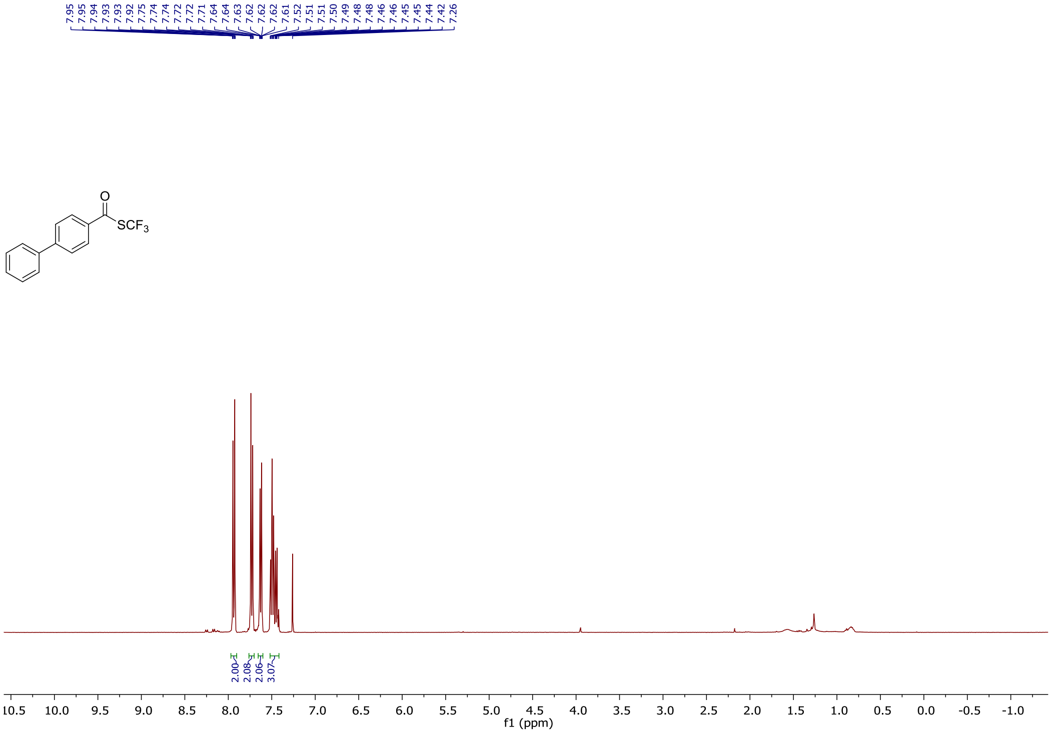

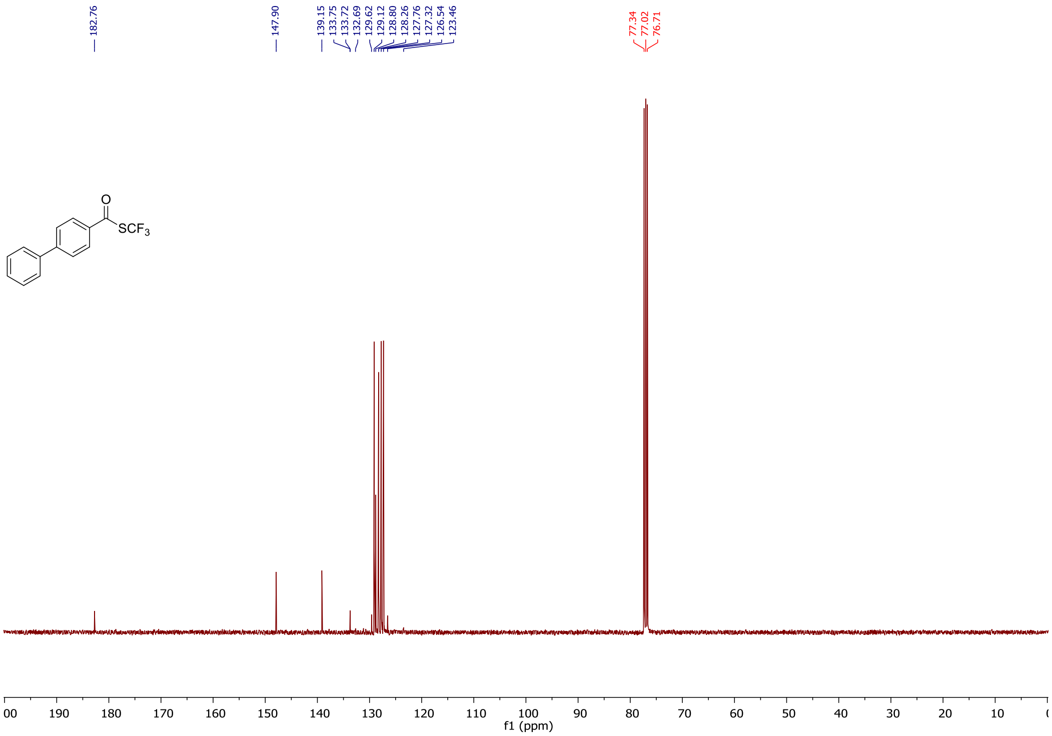

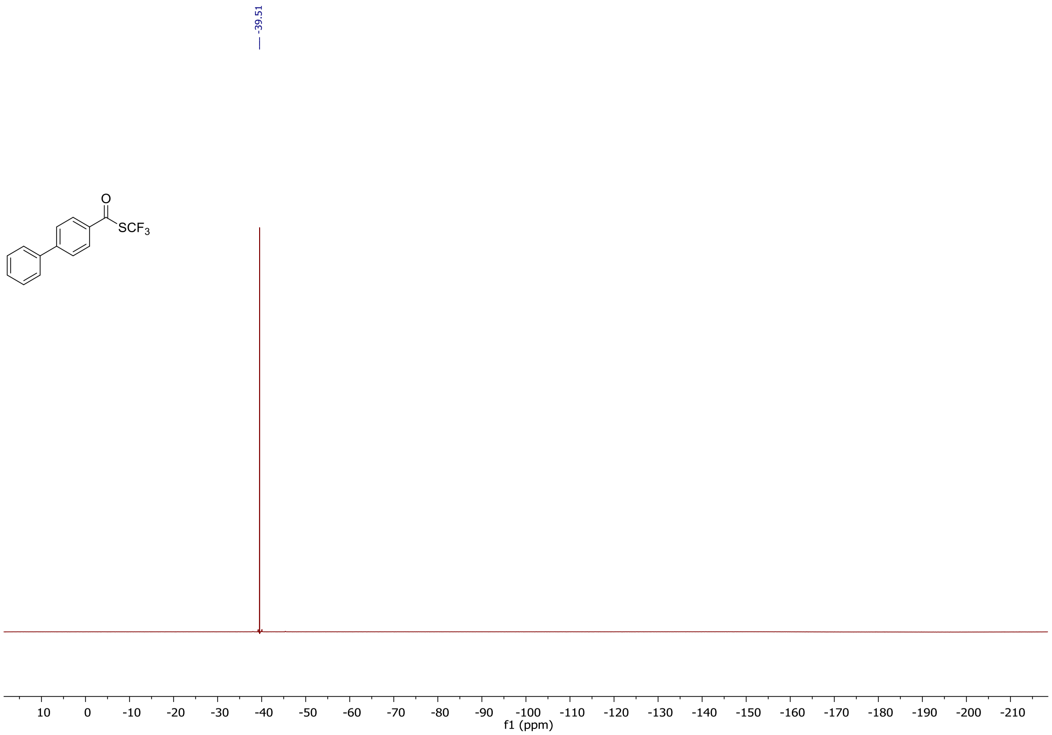

-39.51
O
SCF3
10 0 -10 -20 -30 -40 -50 -60 -70 -80 -90 -100 -110 -120 -130 -140 -150 -160 -170 -180 -190 -200 -210
f1 (ppm)

Figure S2. NMR spectra of 3b

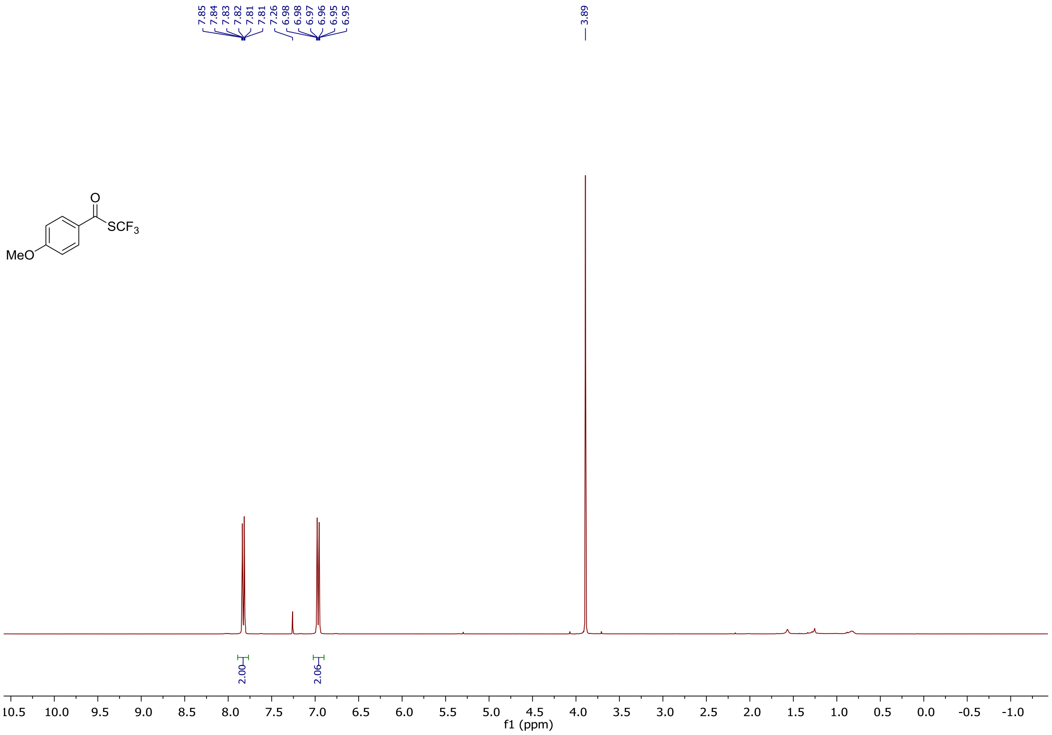

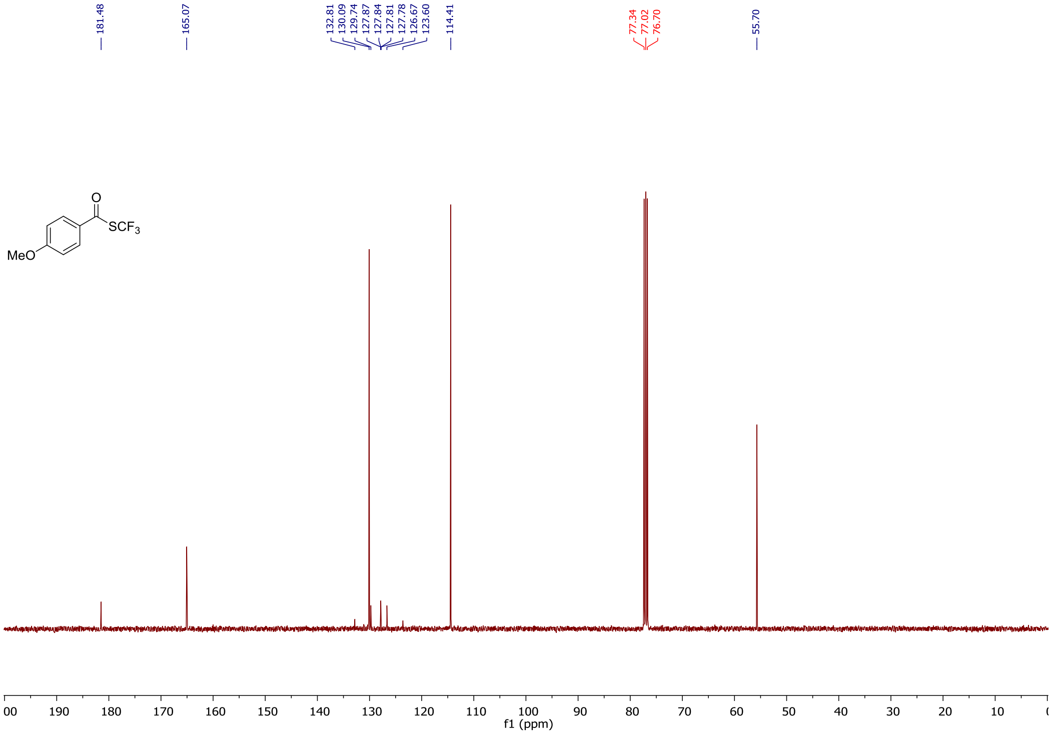

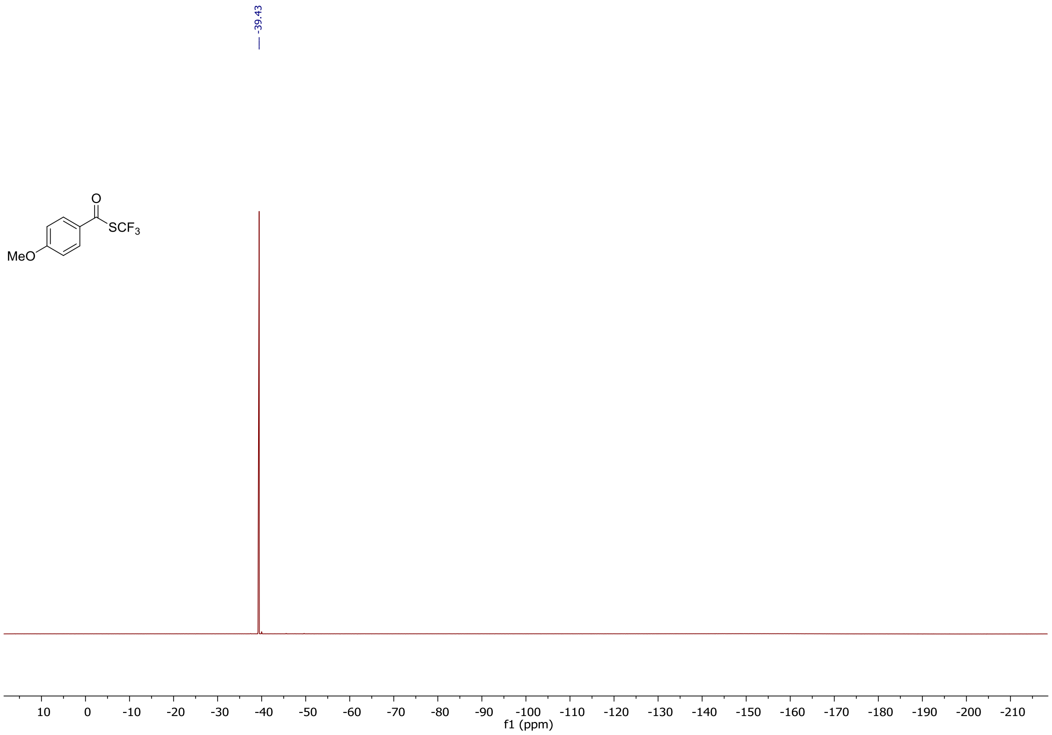

-39.43
O
SCF3
MeO
10 0 -10 -20 -30 -40 -50 -60 -70 -80 -90 -100 -110 -120 -130 -140 -150 -160 -170 -180 -190 -200 -210
f1 (ppm)

Figure S3. NMR spectra of 3c

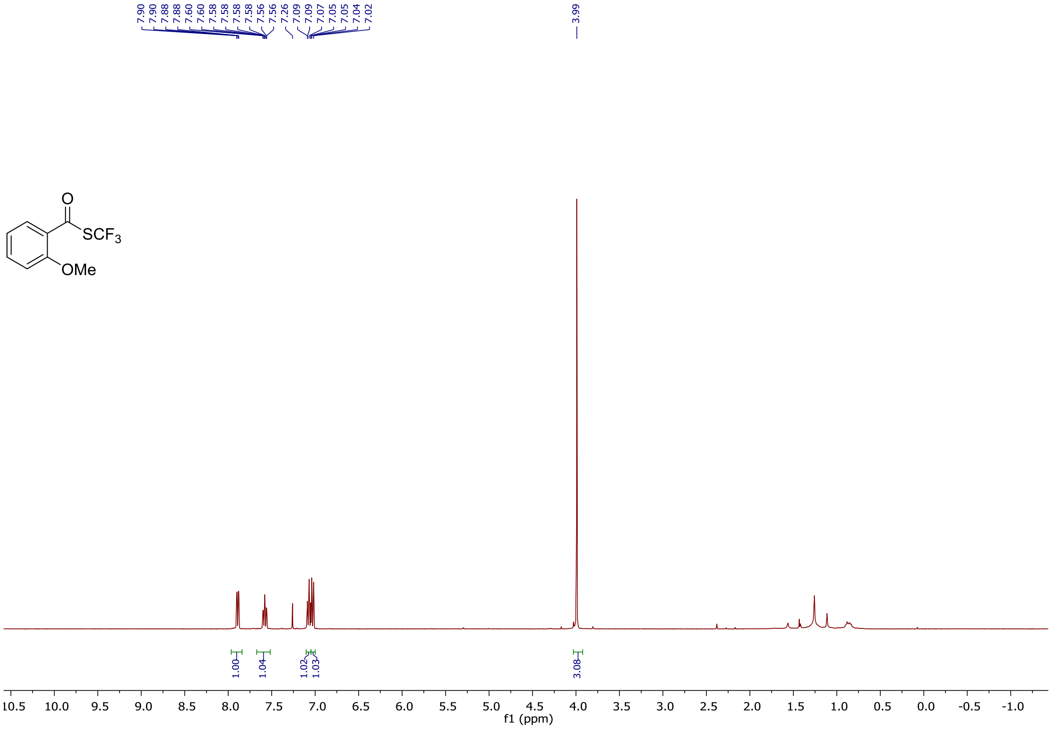

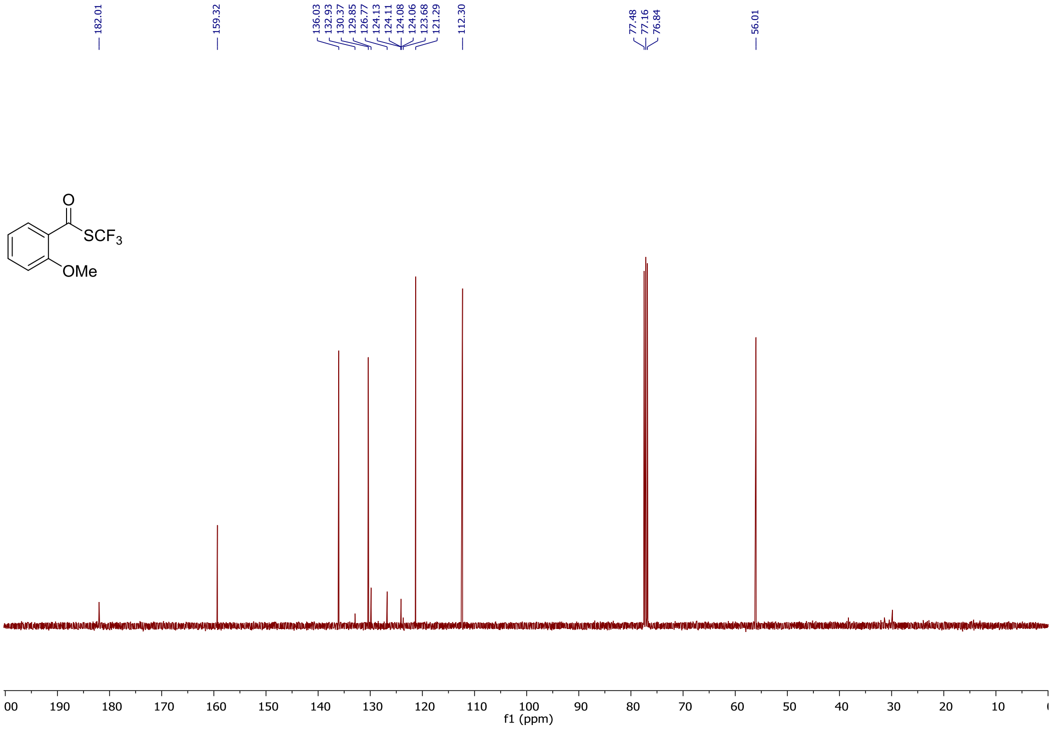

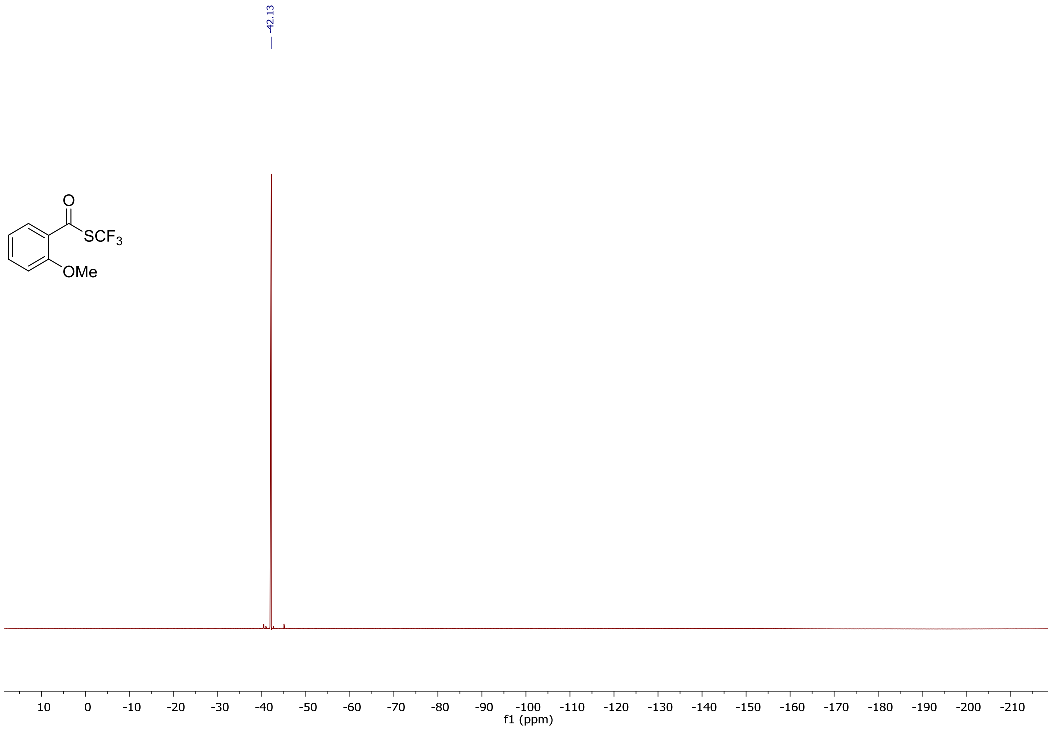
-42.13
SCF3
OMe
O
10 0 -10 -20 -30 -40 -50 -60 -70 -80 -90 -100 -110 -120 -130 -140 -150 -160 -170 -180 -190 -200 -210
f1 (ppm)

Figure S4. NMR spectra of 3d

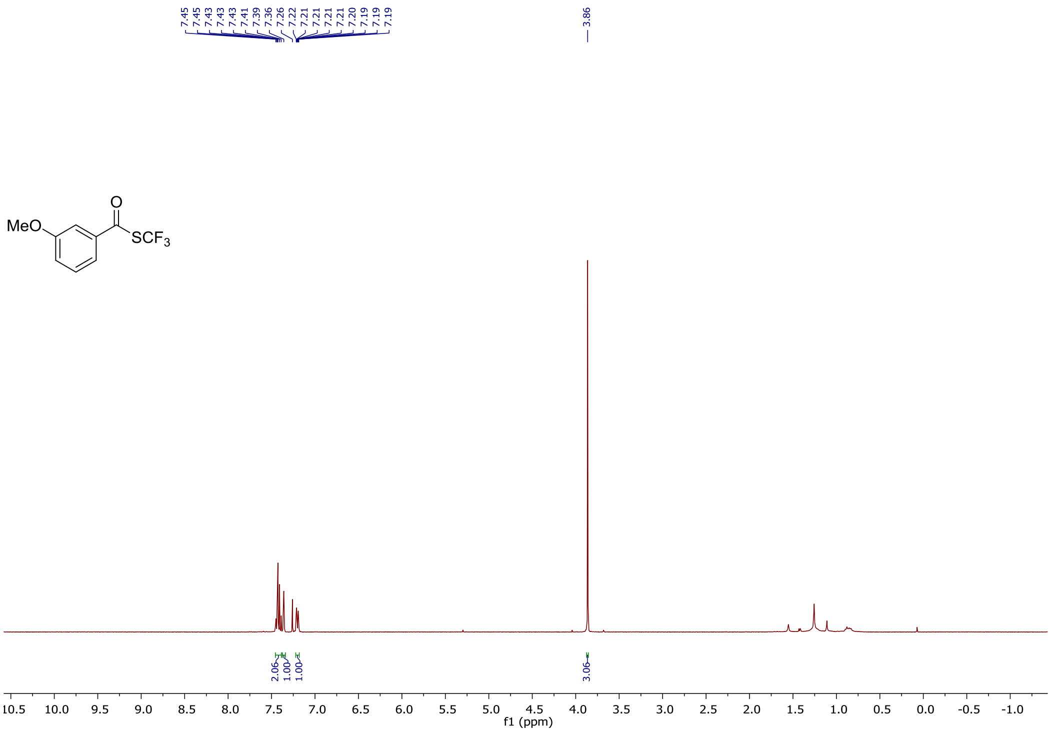

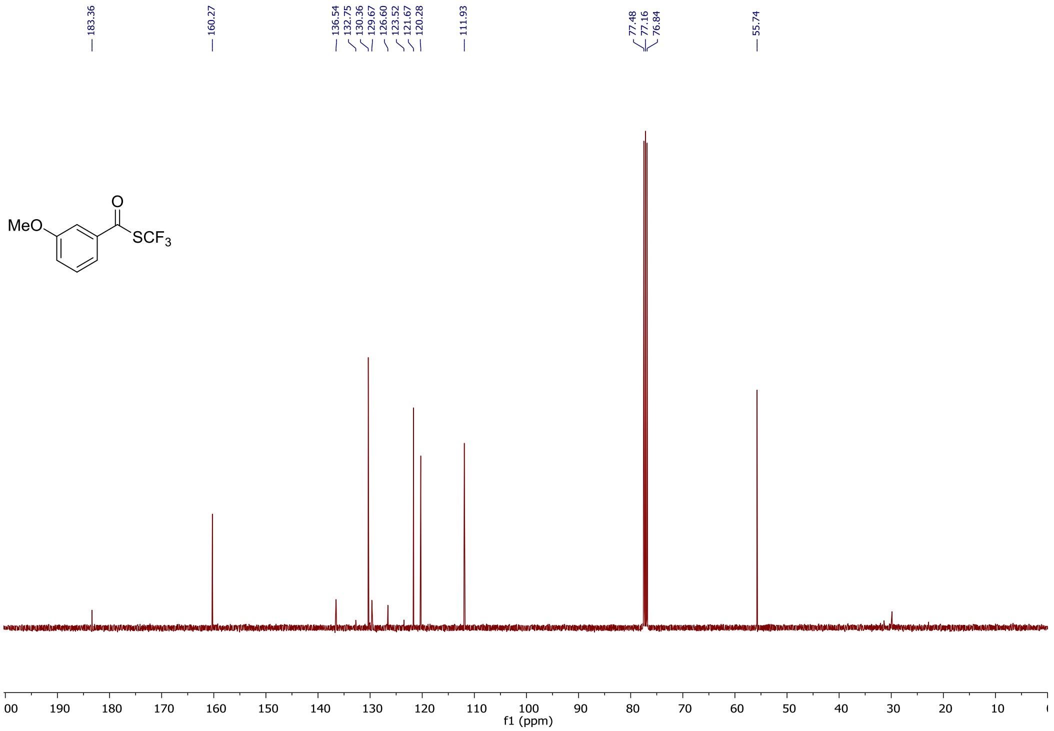

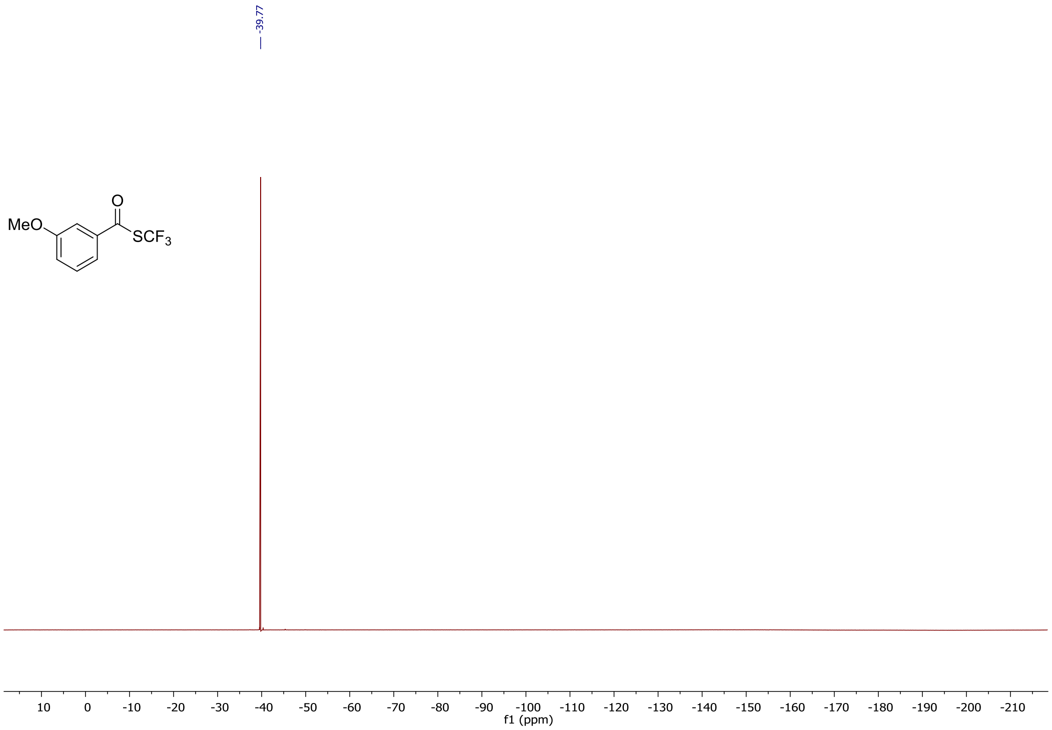

-39.77
MeO
O
SCF3
10 0 -10 -20 -30 -40 -50 -60 -70 -80 -90 -100 -110 -120 -130 -140 -150 -160 -170 -180 -190 -200 -210
f1 (ppm)

Figure S5. NMR spectra of 3e

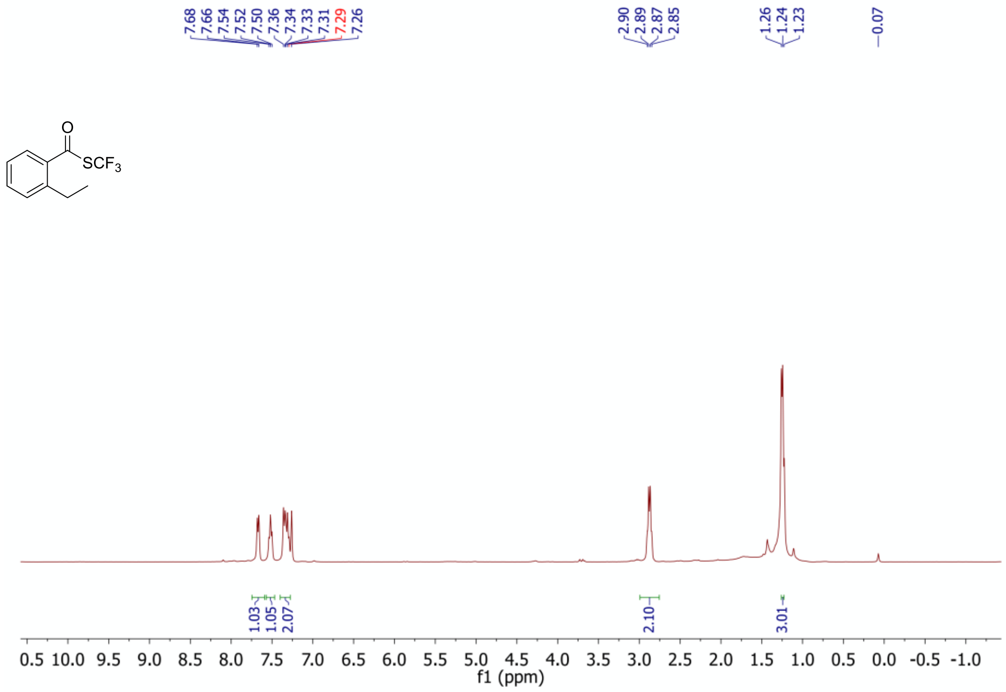

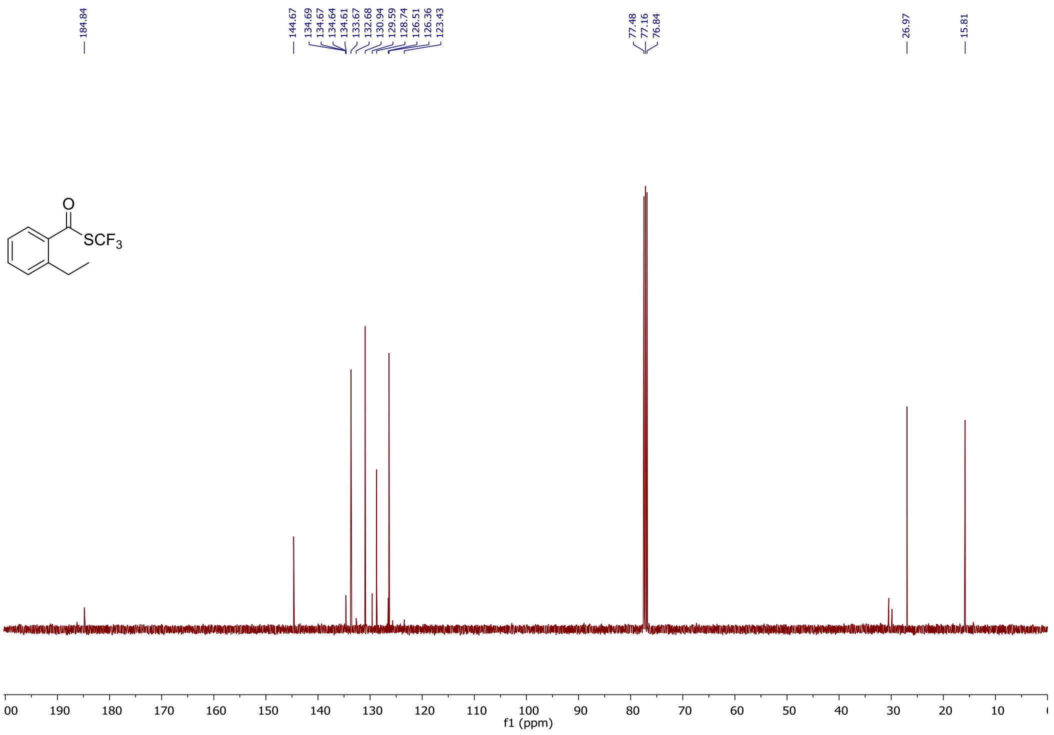

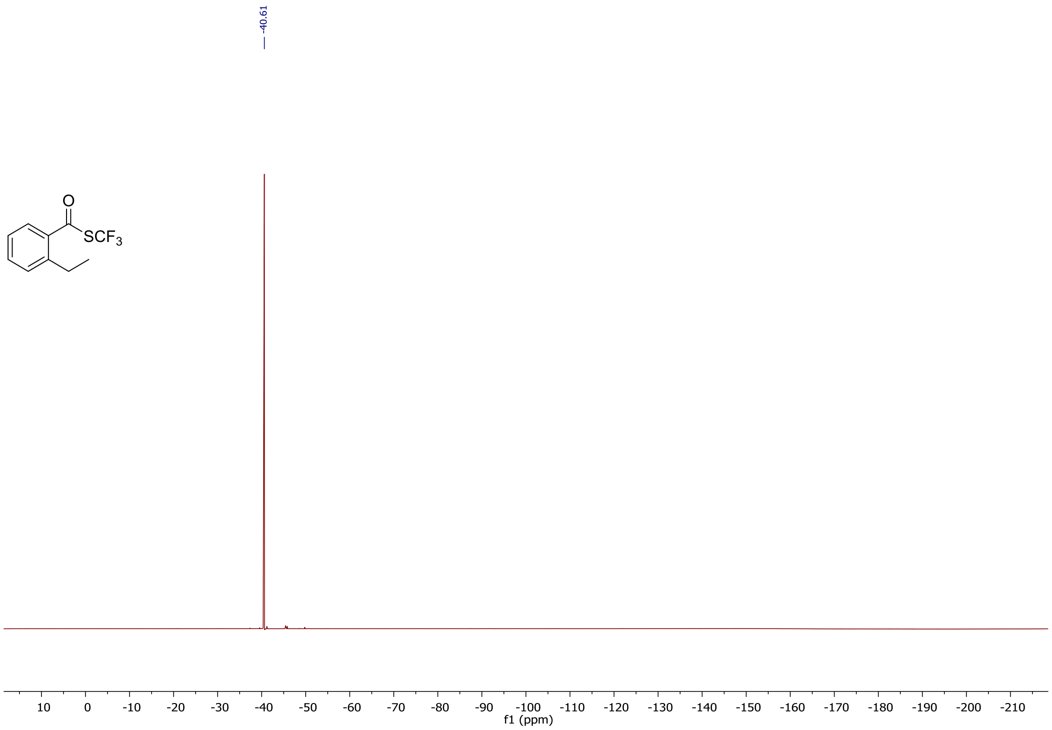
-40.61
O
SCF3
10 0 -10 -20 -30 -40 -50 -60 -70 -80 -90 -100 -110 -120 -130 -140 -150 -160 -170 -180 -190 -200 -210
f1 (ppm)

Figure S6. NMR spectra of 3f

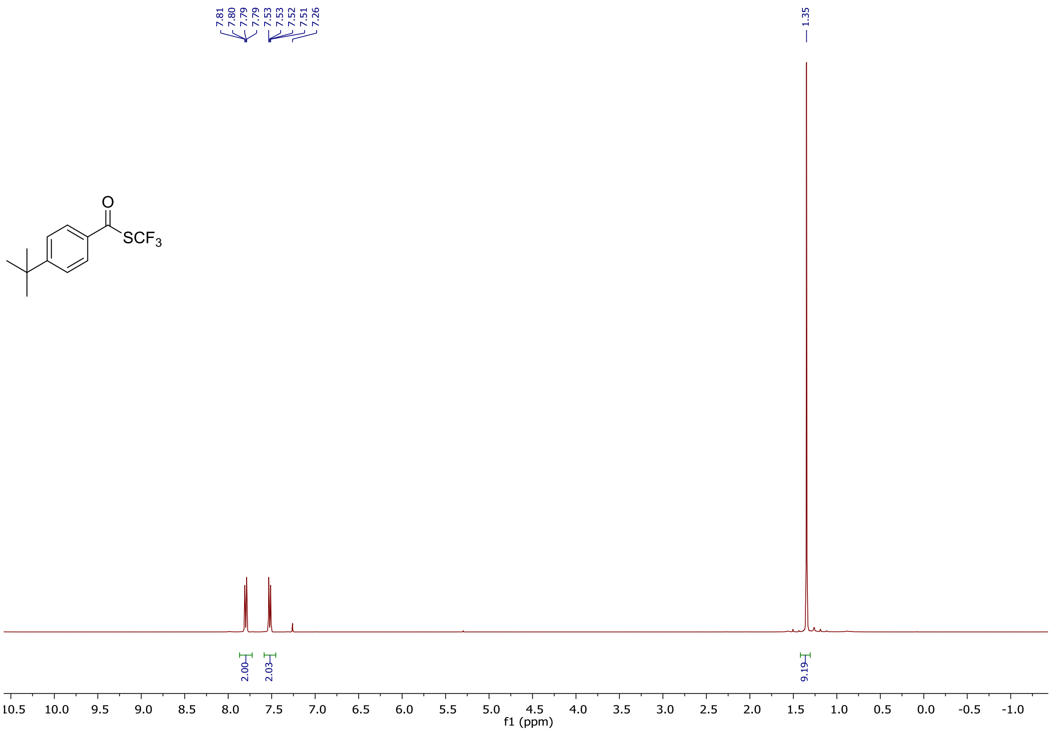

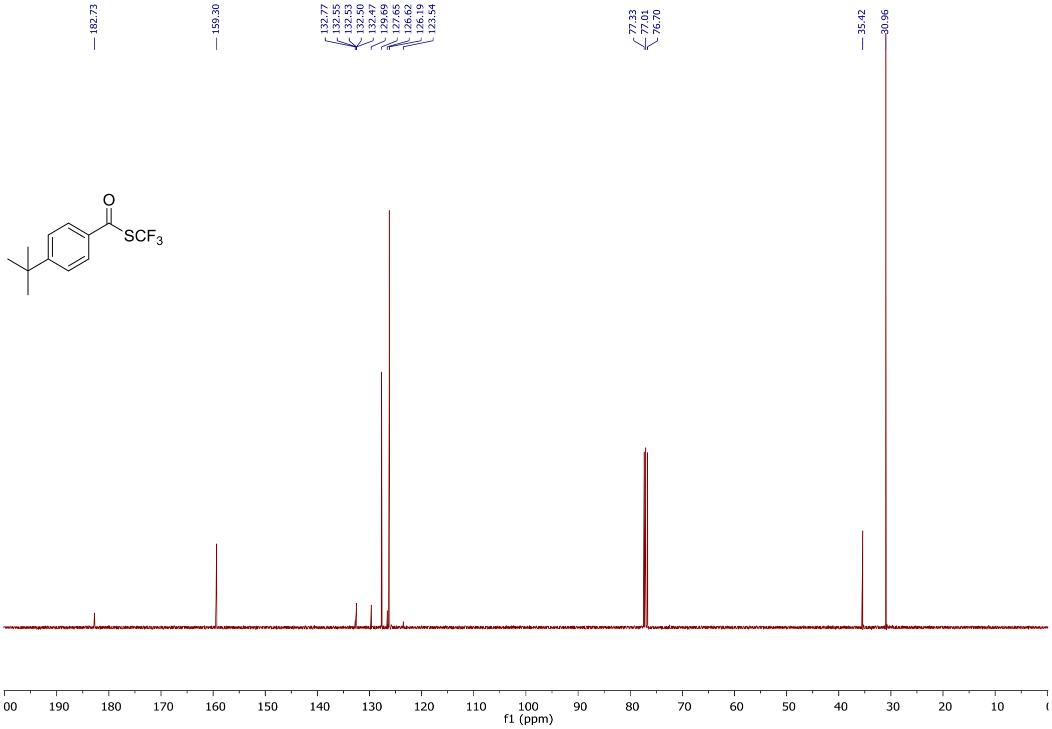

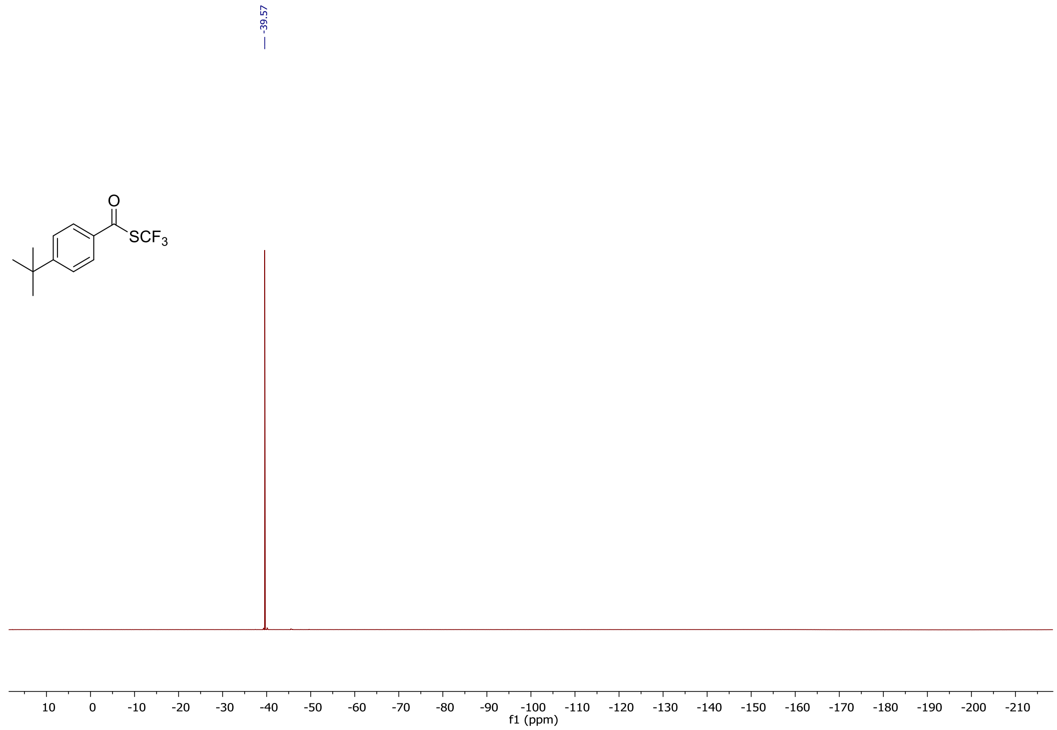

-39.57
O
SCF3
10 0 -10 -20 -30 -40 -50 -60 -70 -80 -90 -100 -110 -120 -130 -140 -150 -160 -170 -180 -190 -200 -210
f1 (ppm)

Figure S7. **NMR spectra of 3g**

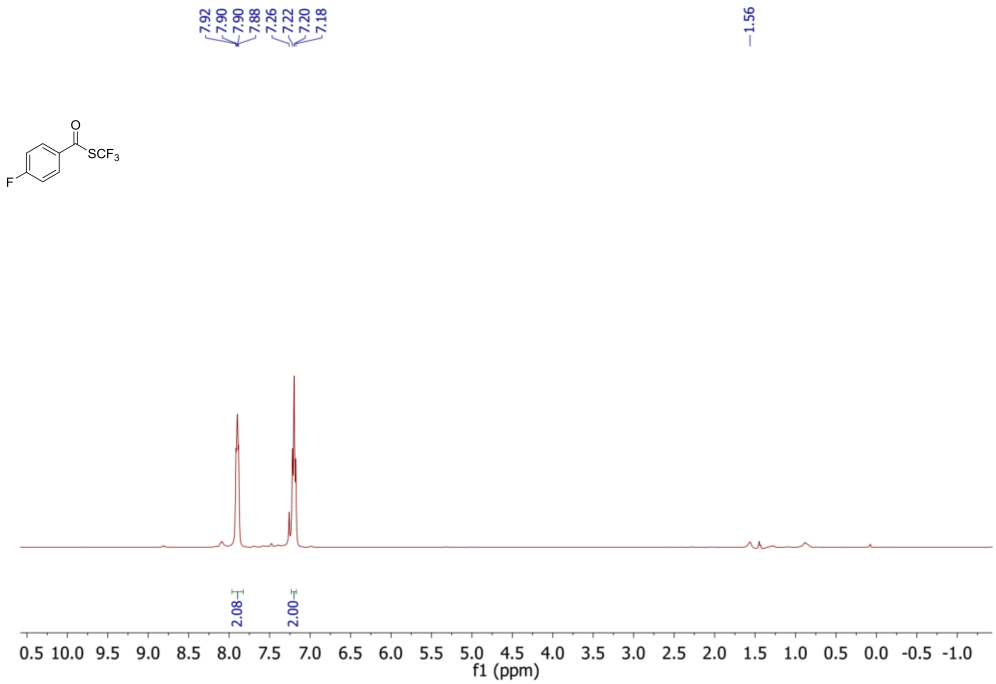

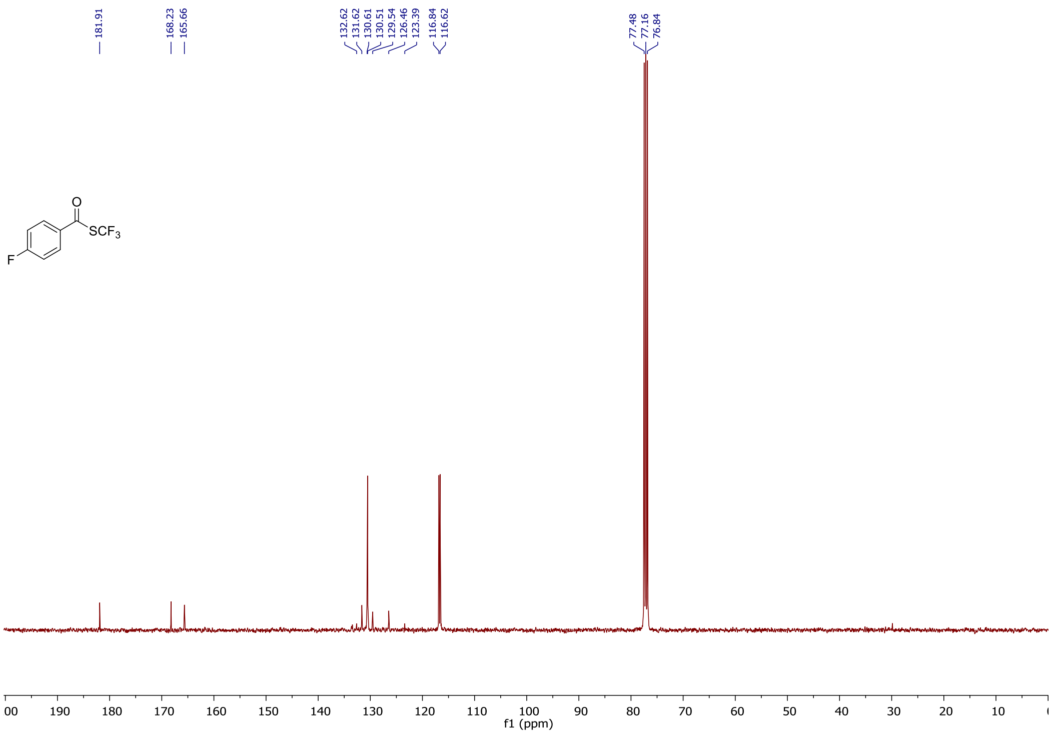

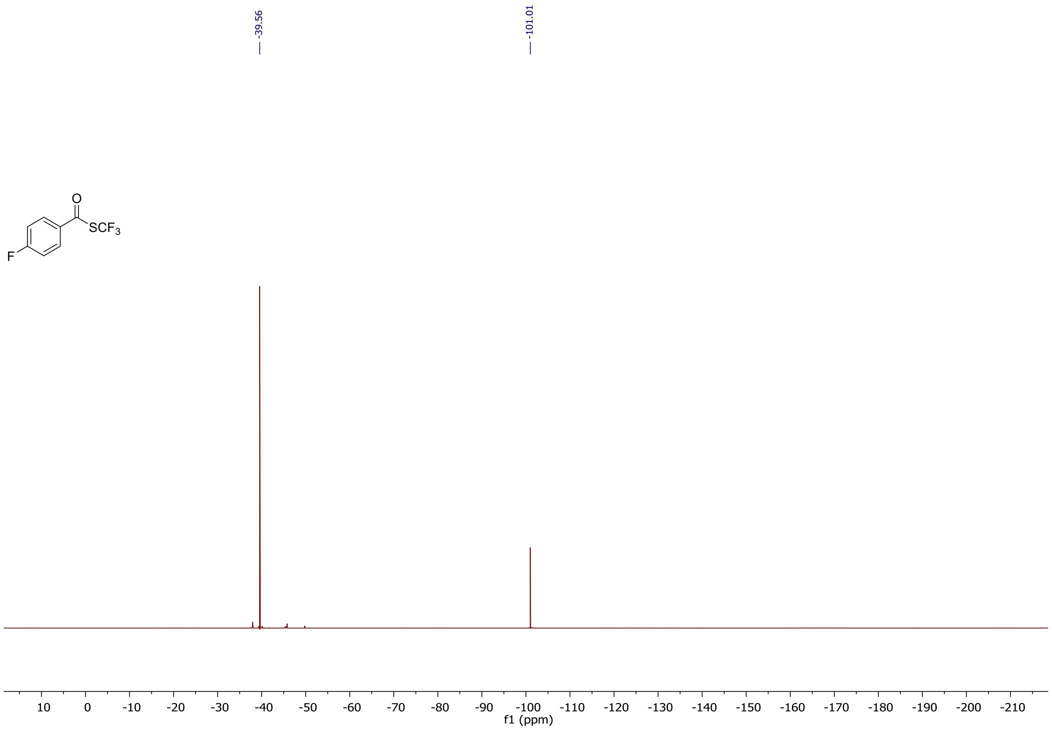
-39.56
-101.01
O
SCF3
F
10
0
-10
-20
-30
-40
-50
-60
-70
-80
-90
-100
-110
-120
-130
-140
-150
-160
-170
-180
-190
-200
-210
f1 (ppm)

Figure S8. NMR spectra of 3h

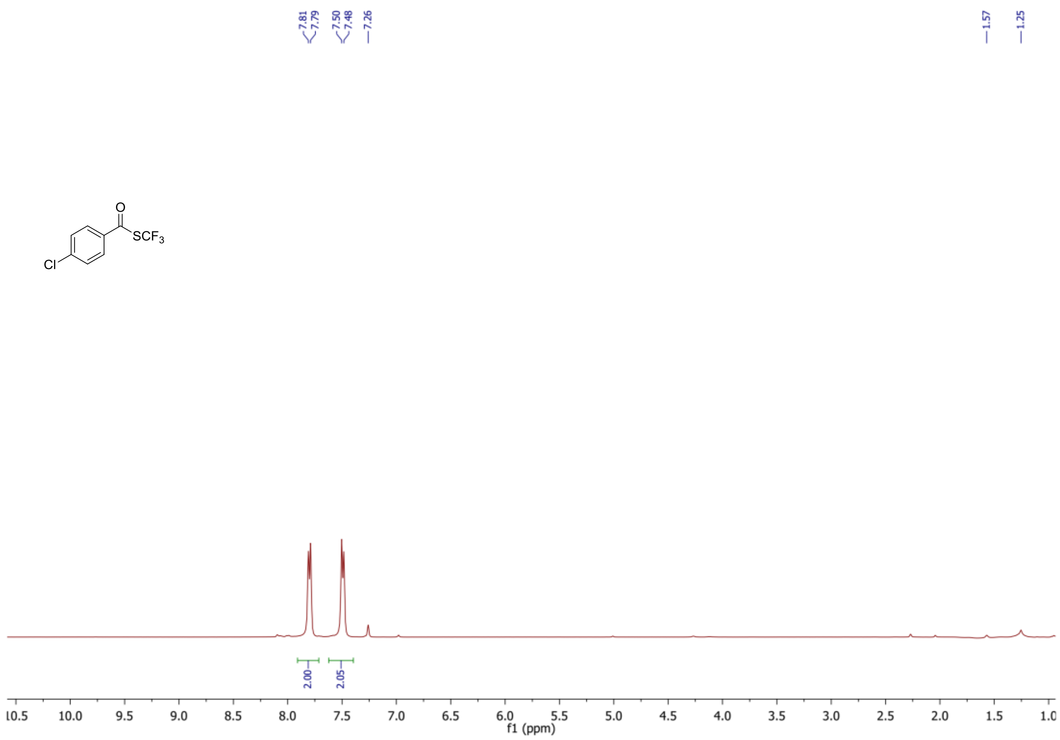

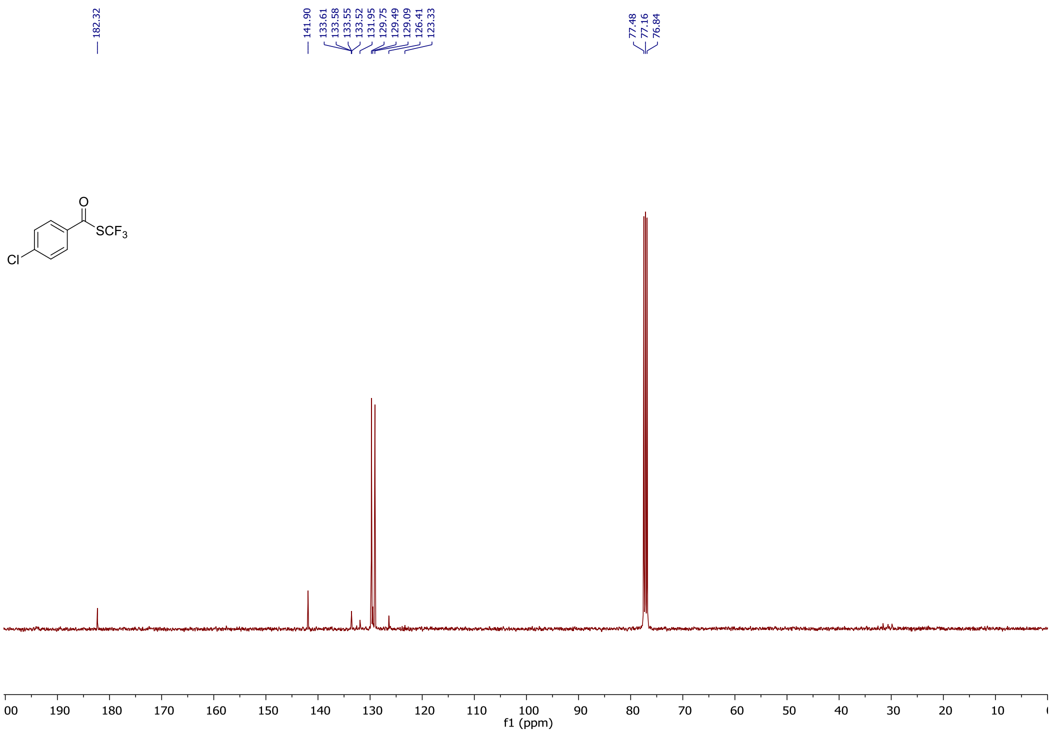

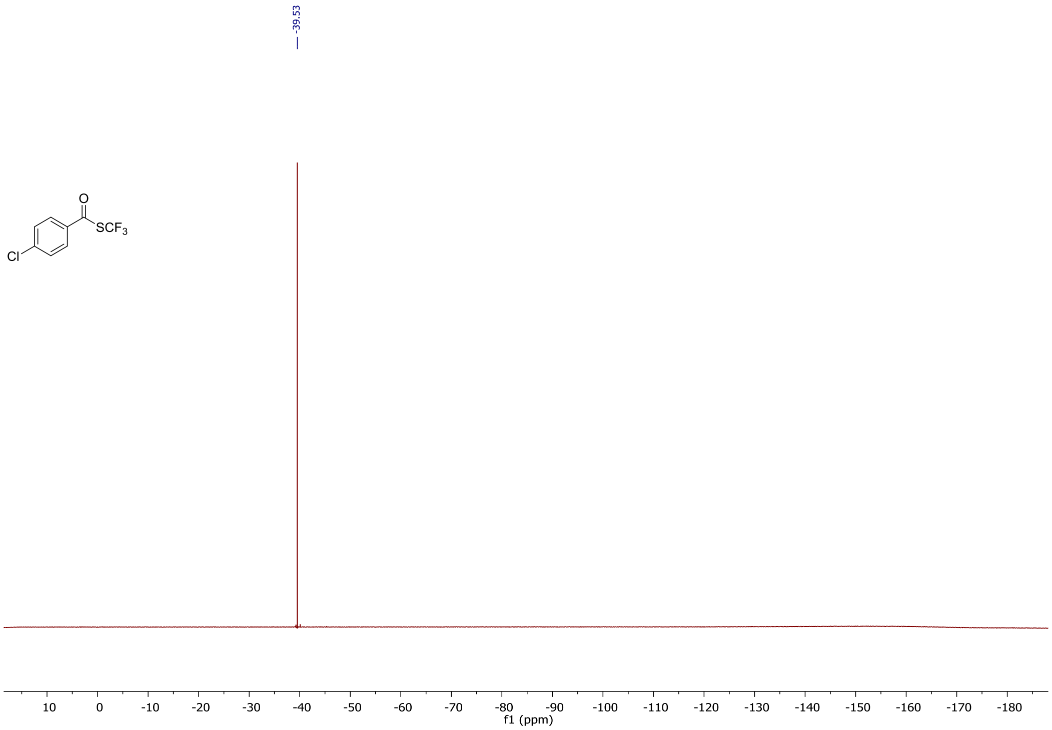

-39.53
SCF3
Cl
O
10 0 -10 -20 -30 -40 -50 -60 -70 -80 -90 -100 -110 -120 -130 -140 -150 -160 -170 -180
f1 (ppm)

Figure S9. NMR spectra of 3i

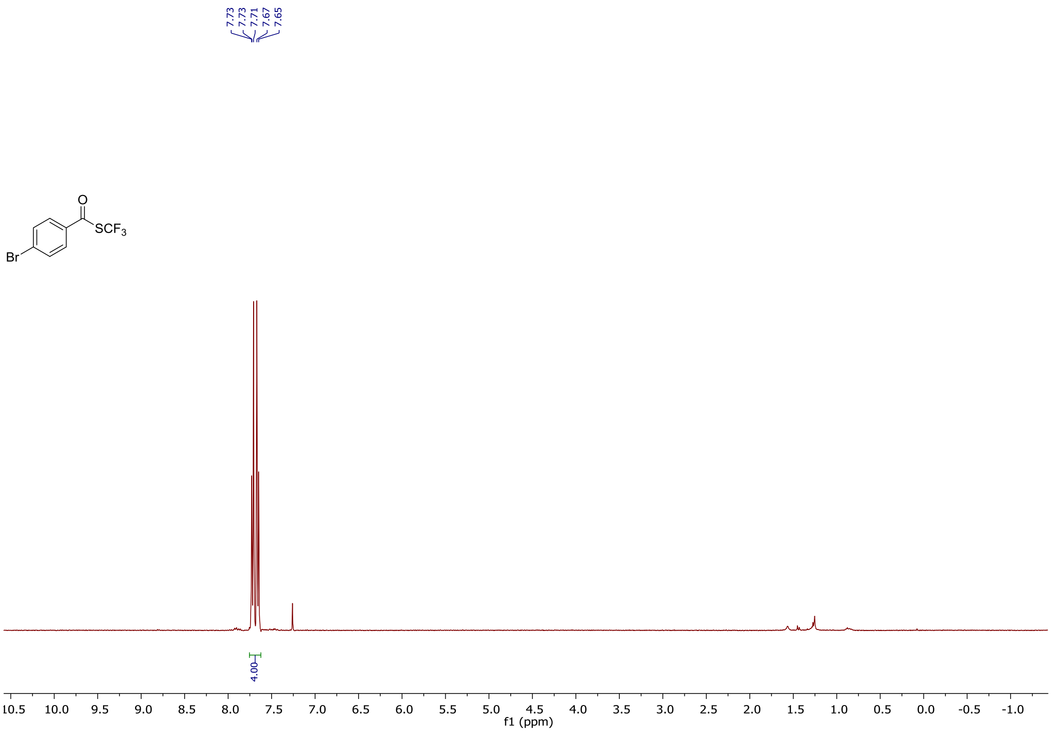

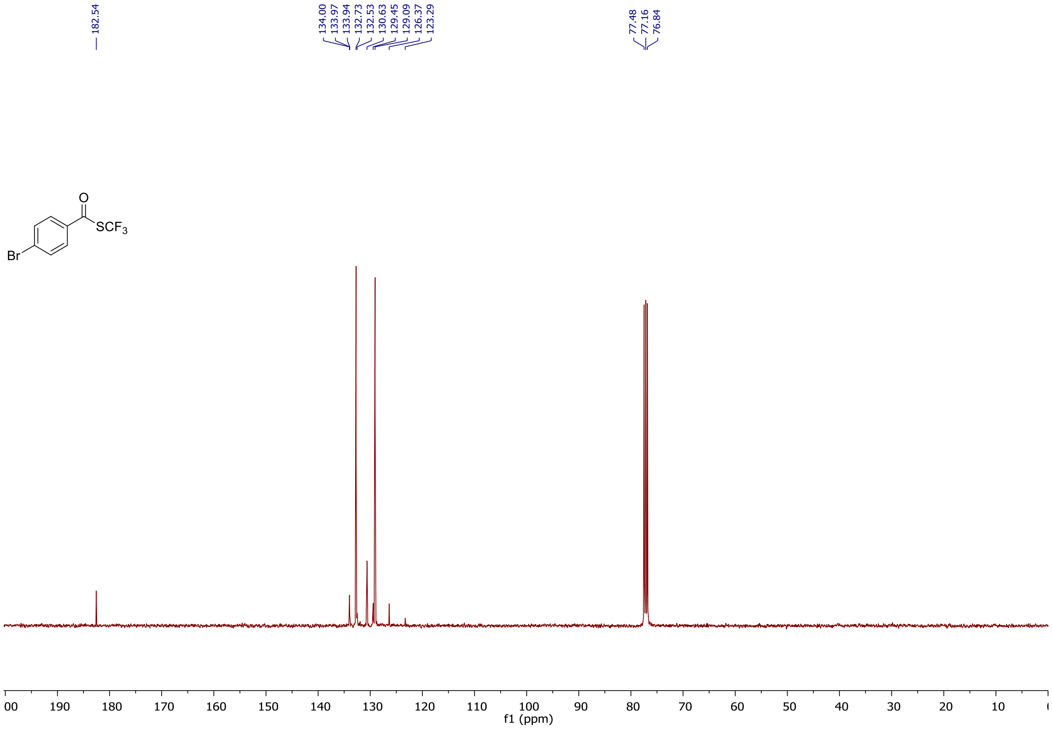

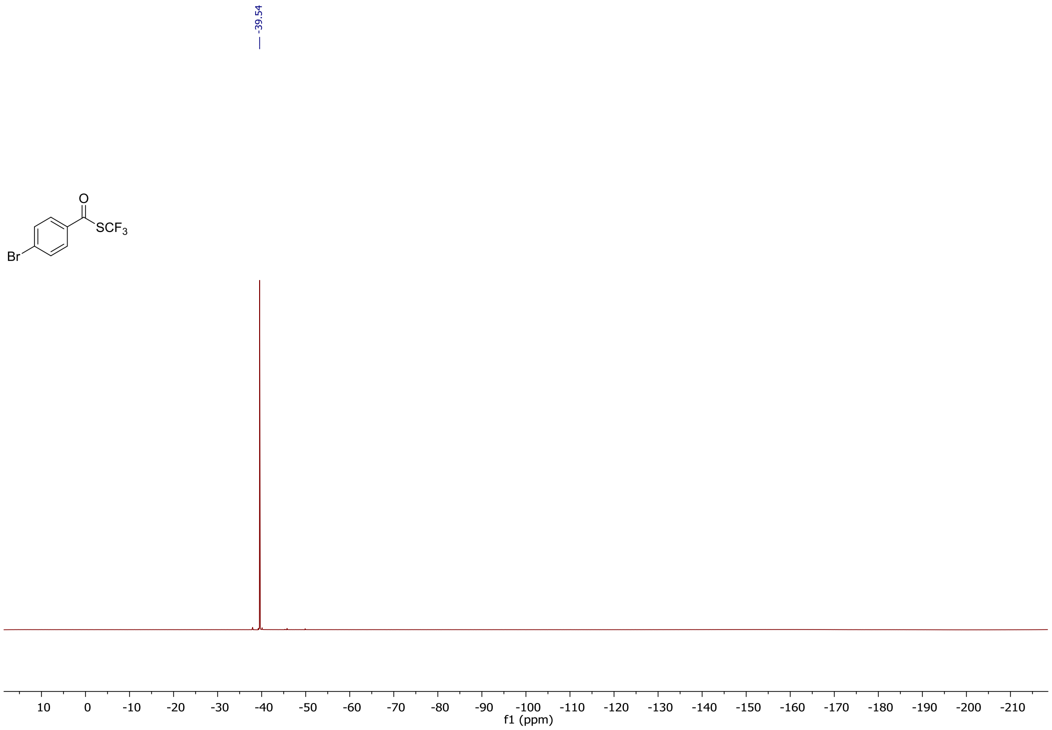
-39.54
O
SCF3
Br
10 0 -10 -20 -30 -40 -50 -60 -70 -80 -90 -100 -110 -120 -130 -140 -150 -160 -170 -180 -190 -200 -210
f1 (ppm)

Figure S10. NMR spectra of 3j

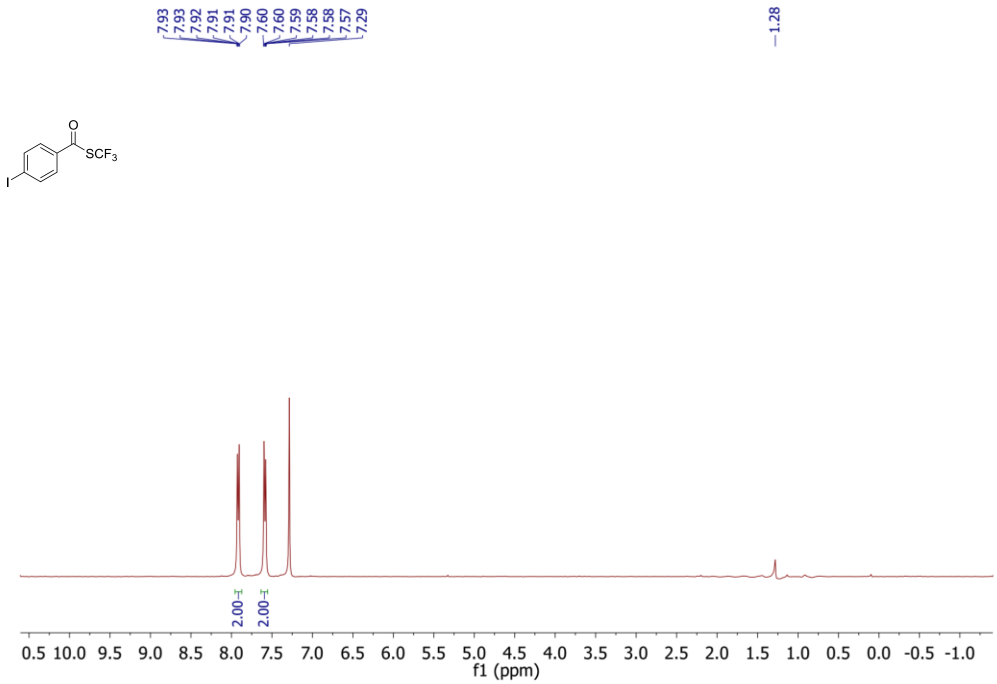

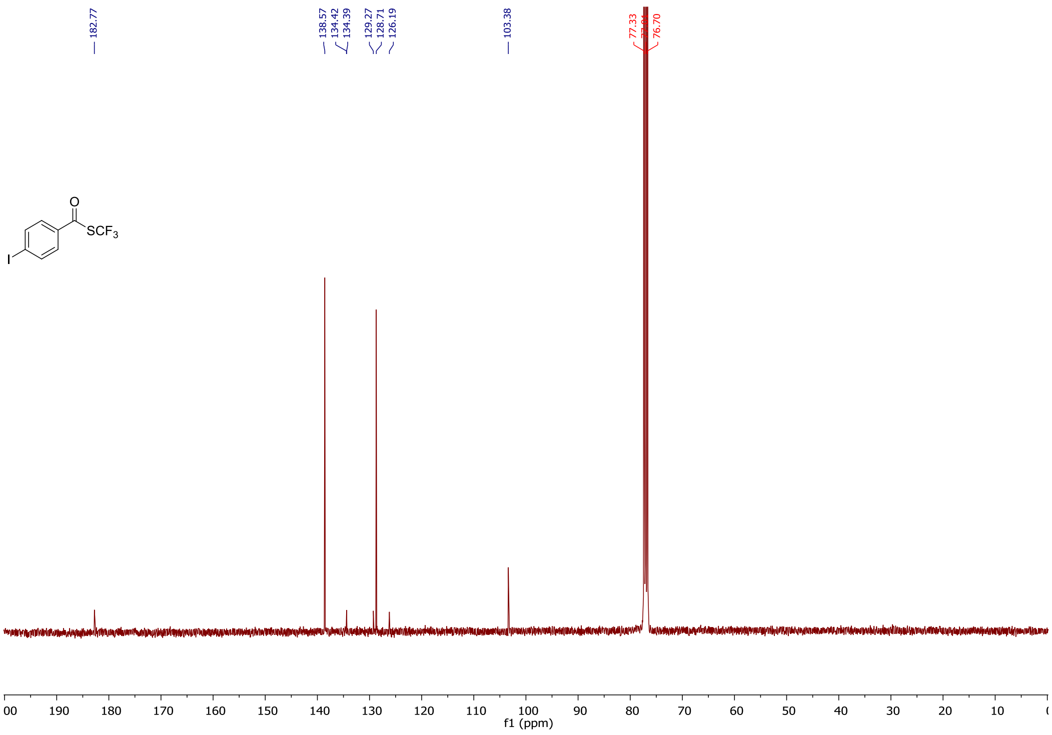

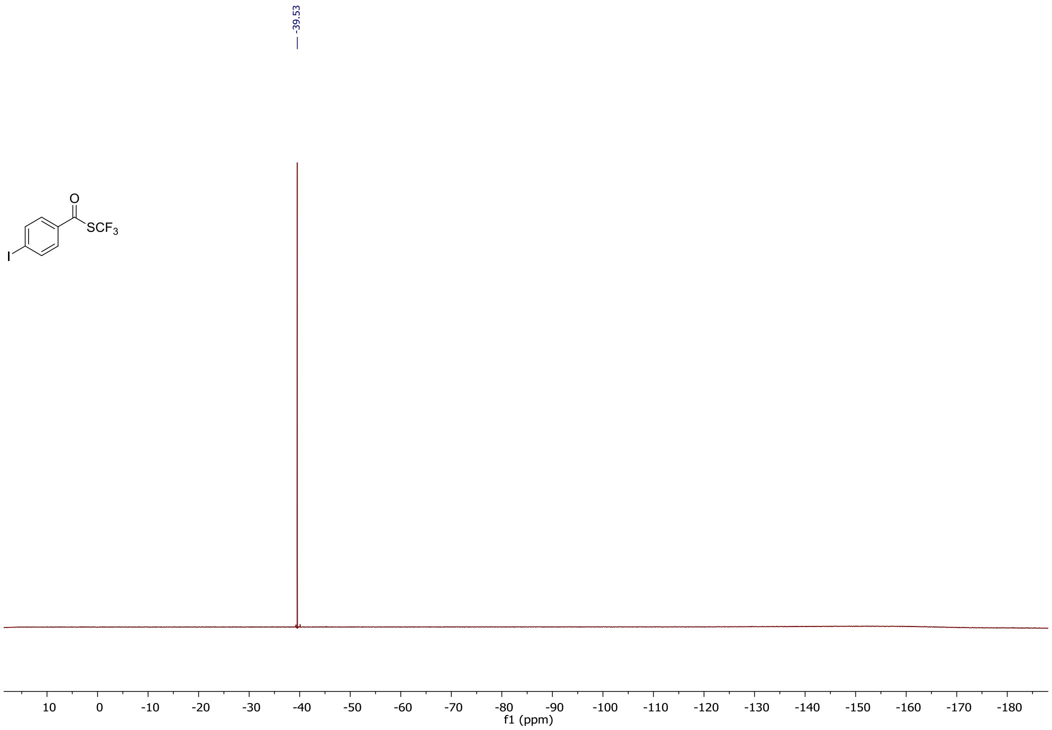

-39.53
O
SCF3
I
10 0 -10 -20 -30 -40 -50 -60 -70 -80 -90 -100 -110 -120 -130 -140 -150 -160 -170 -180
f1 (ppm)

Figure S11. NMR spectra of 3k

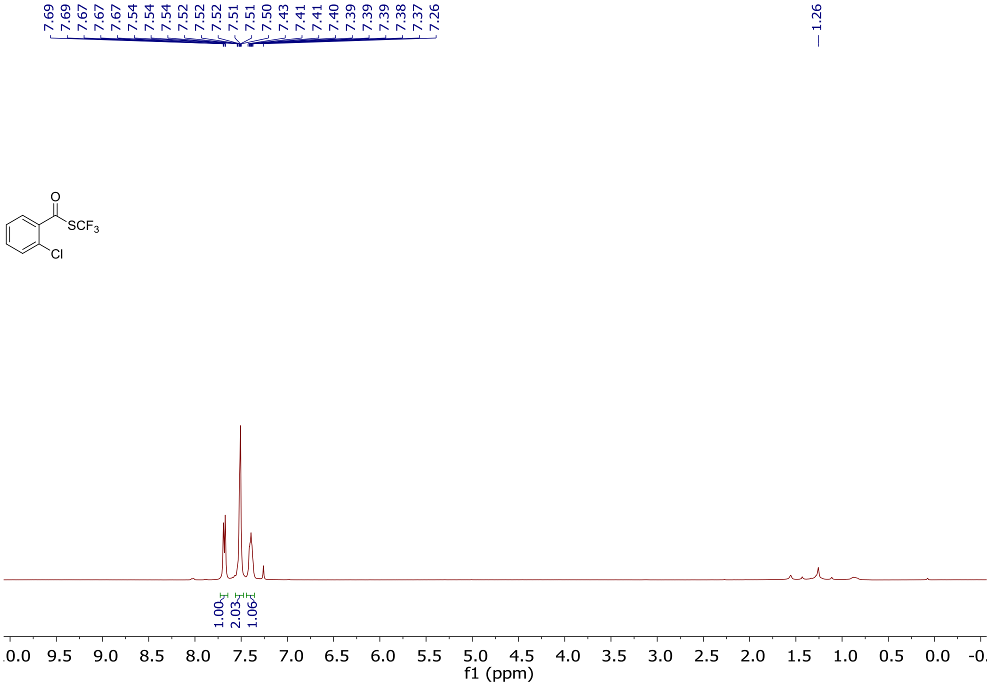

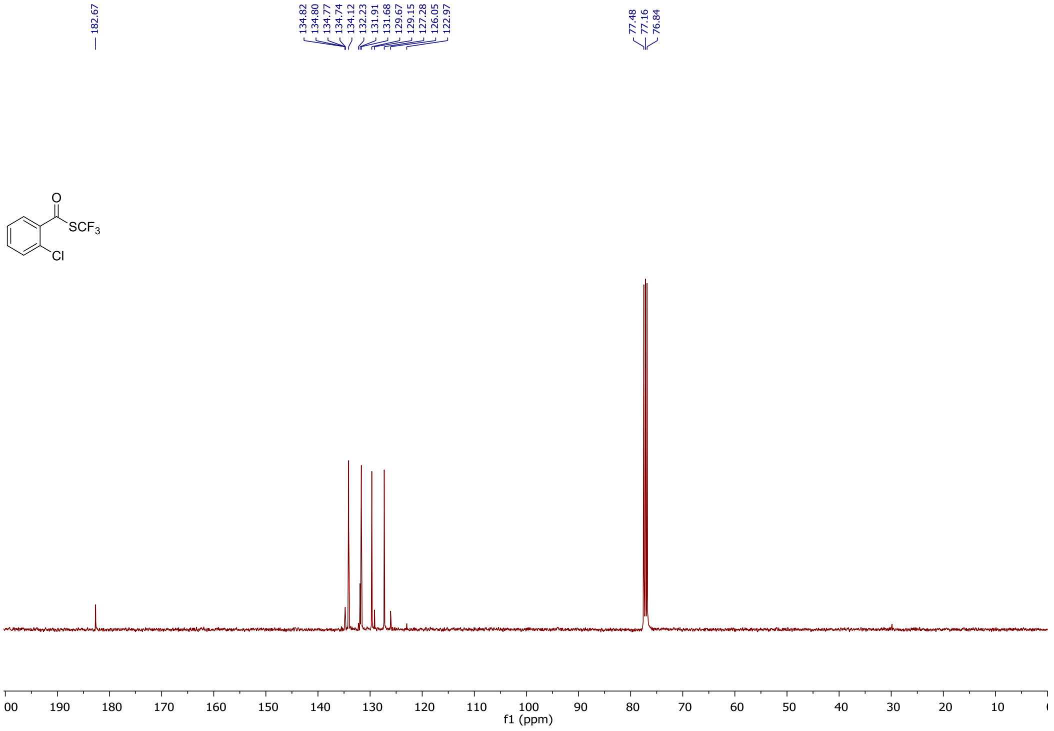

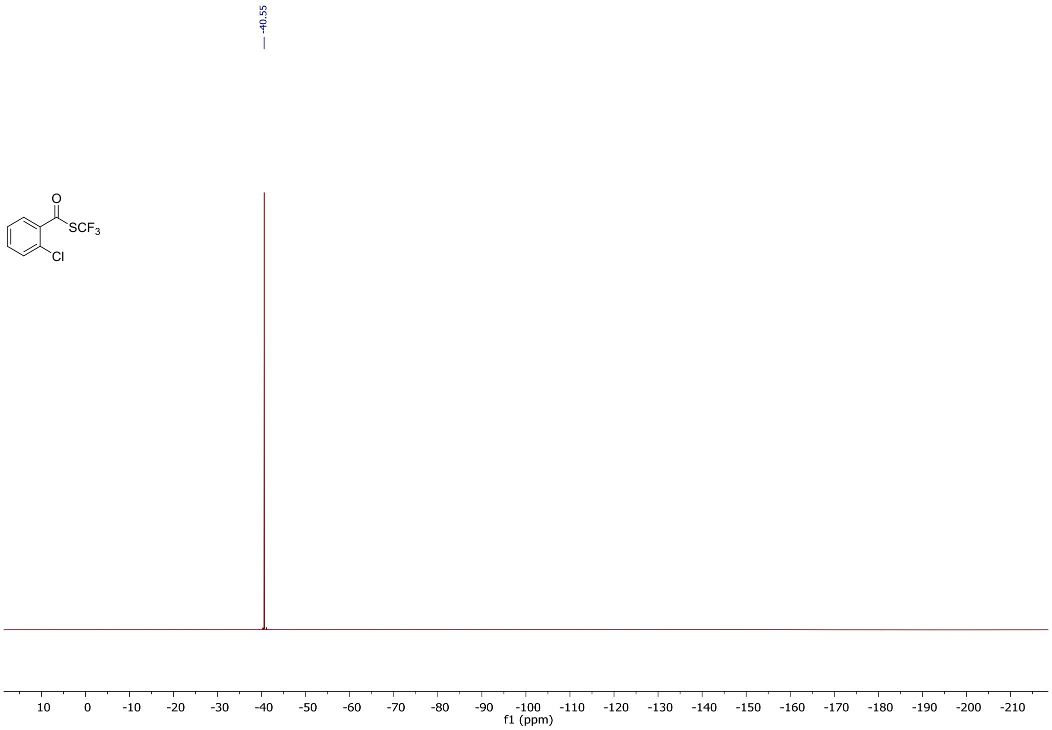
-40.55
SCF3
Cl
O
10
0
-10
-20
-30
-40
-50
-60
-70
-80
-90
-100
-110
-120
-130
-140
-150
-160
-170
-180
-190
-200
-210
f1 (ppm)

Figure S12. NMR spectra of 3l

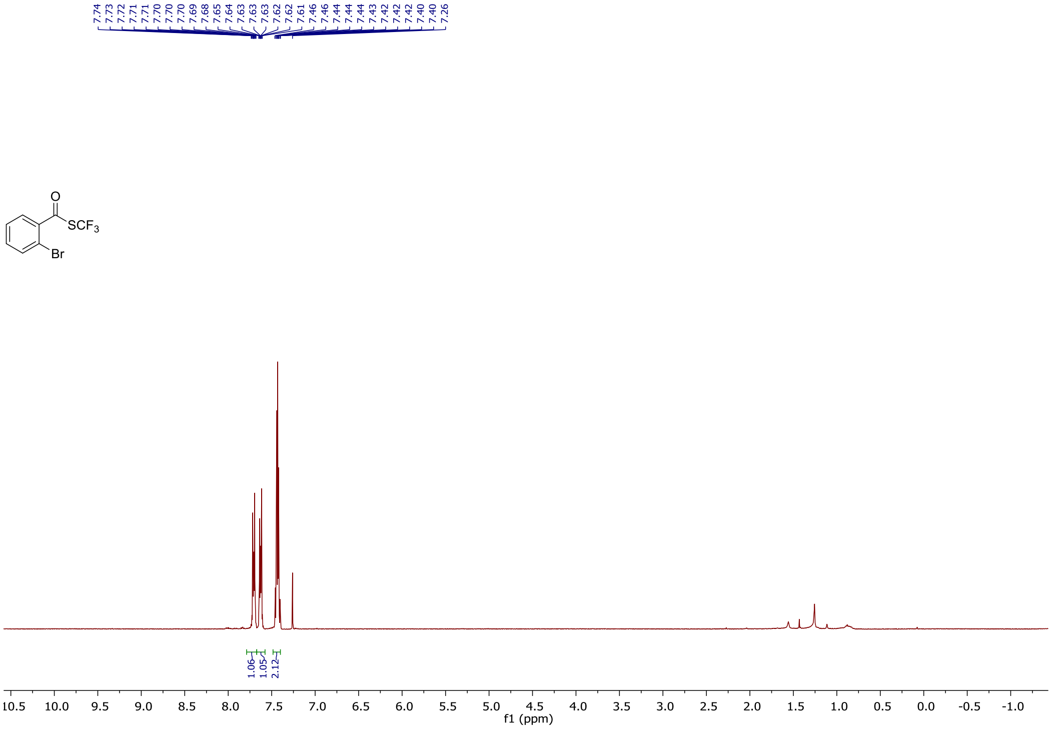

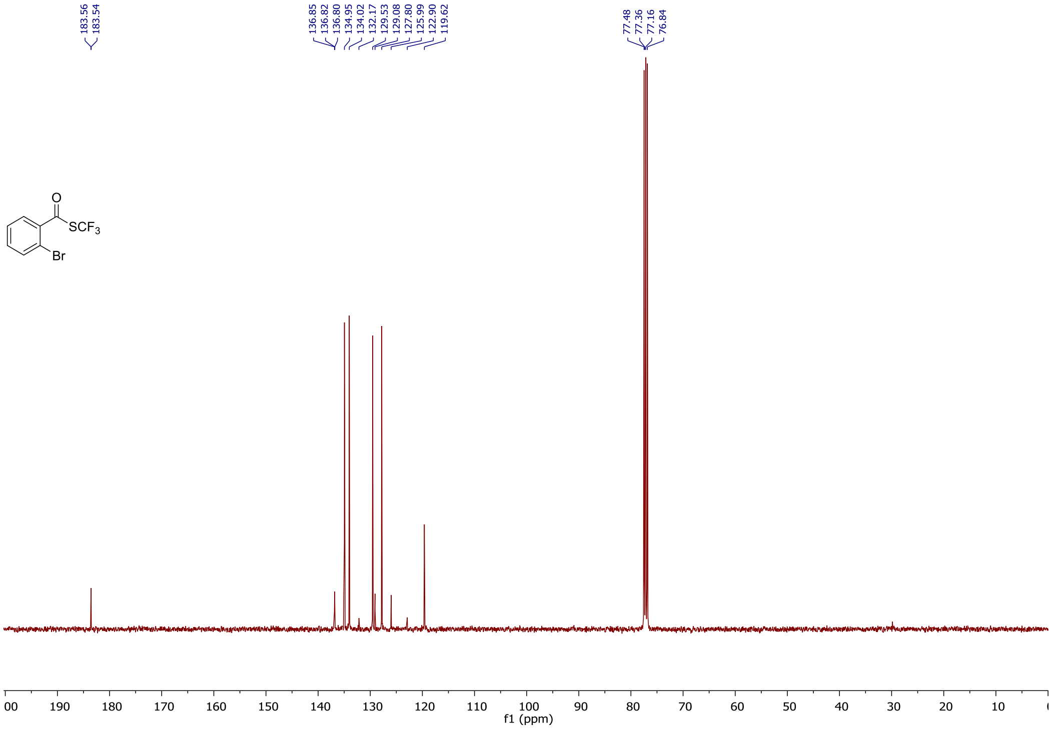

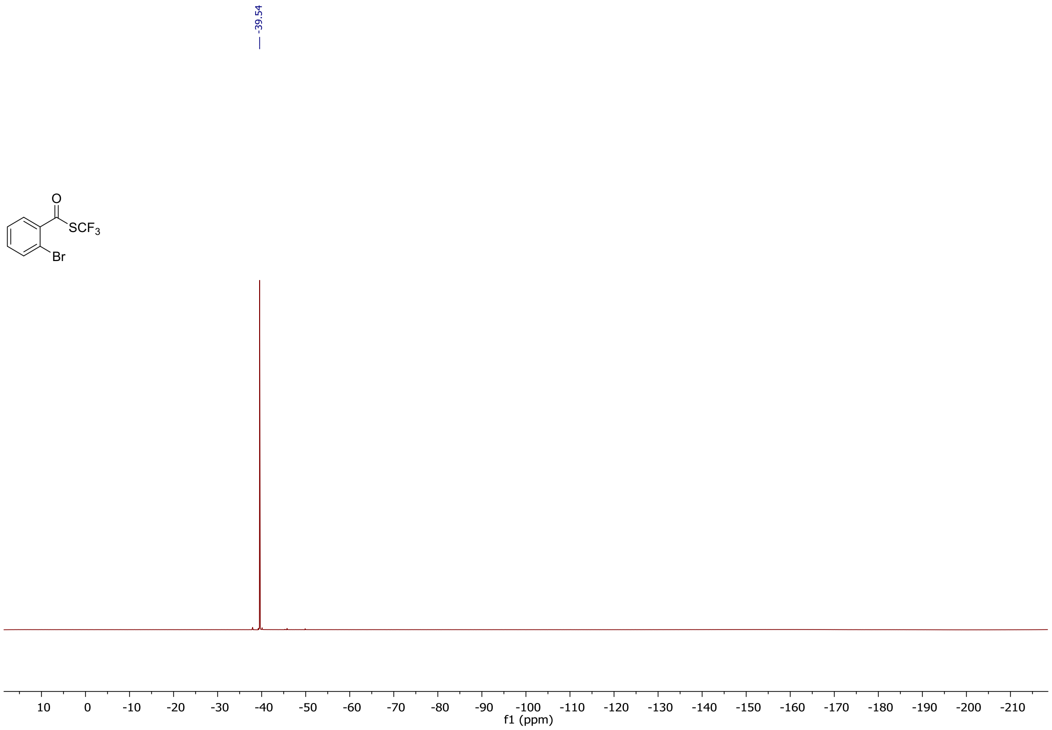

-39.54
SCF3
Br
10
0
-10
-20
-30
-40
-50
-60
-70
-80
-90
-100
-110
-120
-130
-140
-150
-160
-170
-180
-190
-200
-210
f1 (ppm)

Figure S13. NMR spectra of 3m

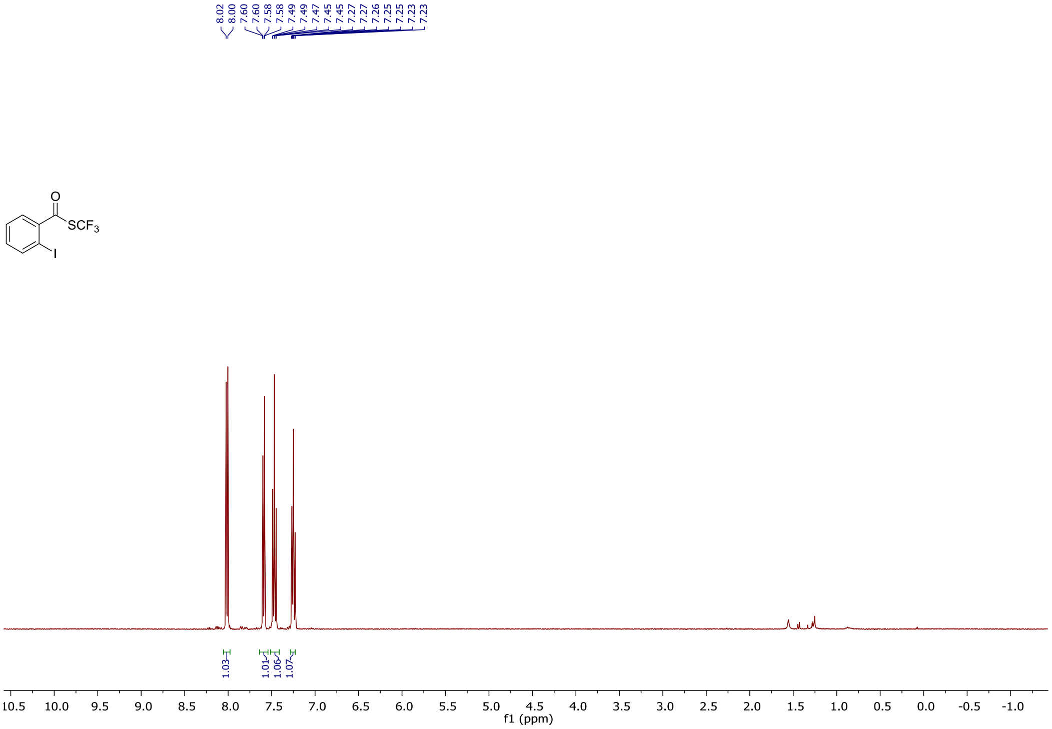

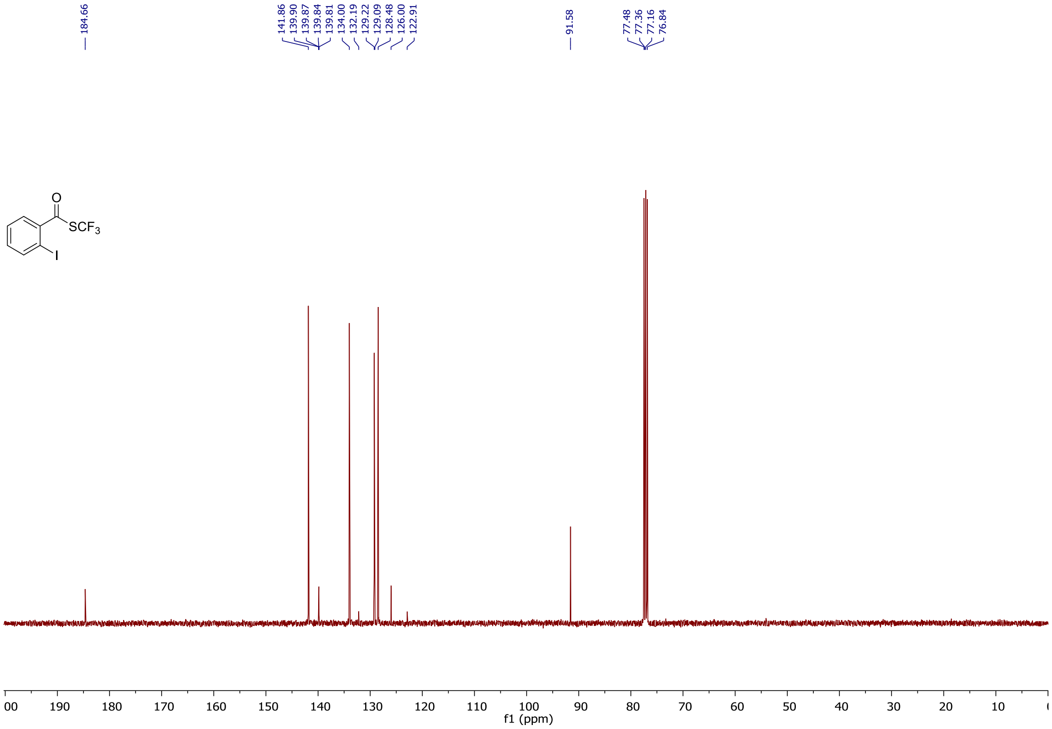

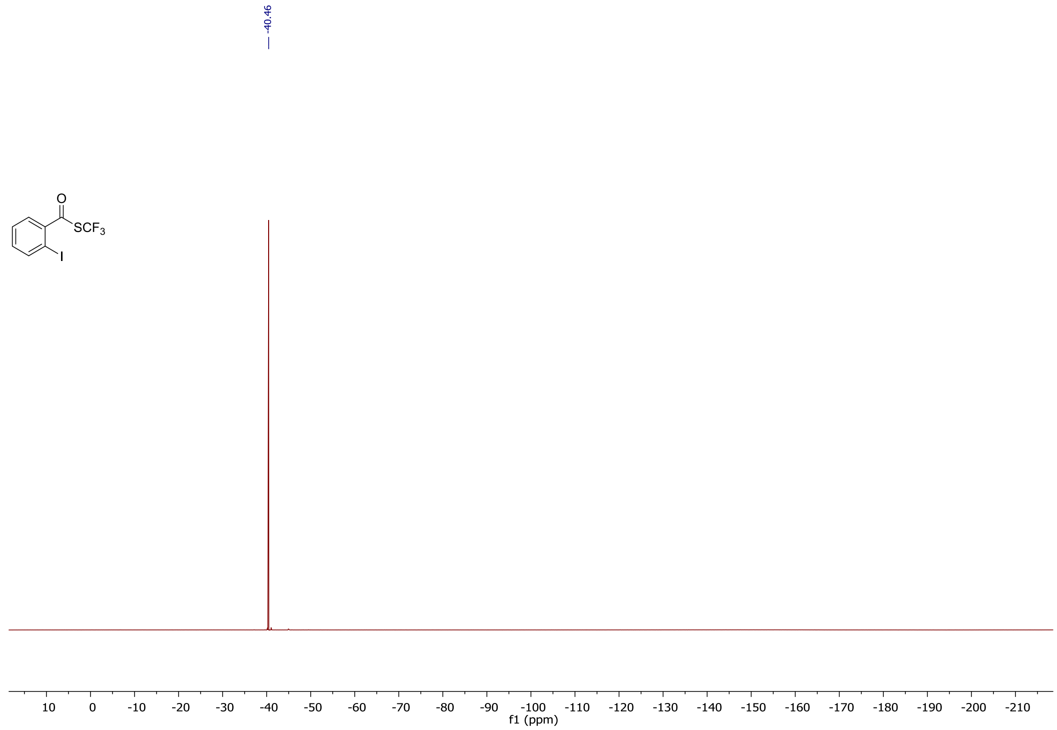

-40.46
SCF3
O
I
f1 (ppm)

Figure S14. NMR spectra of 3n

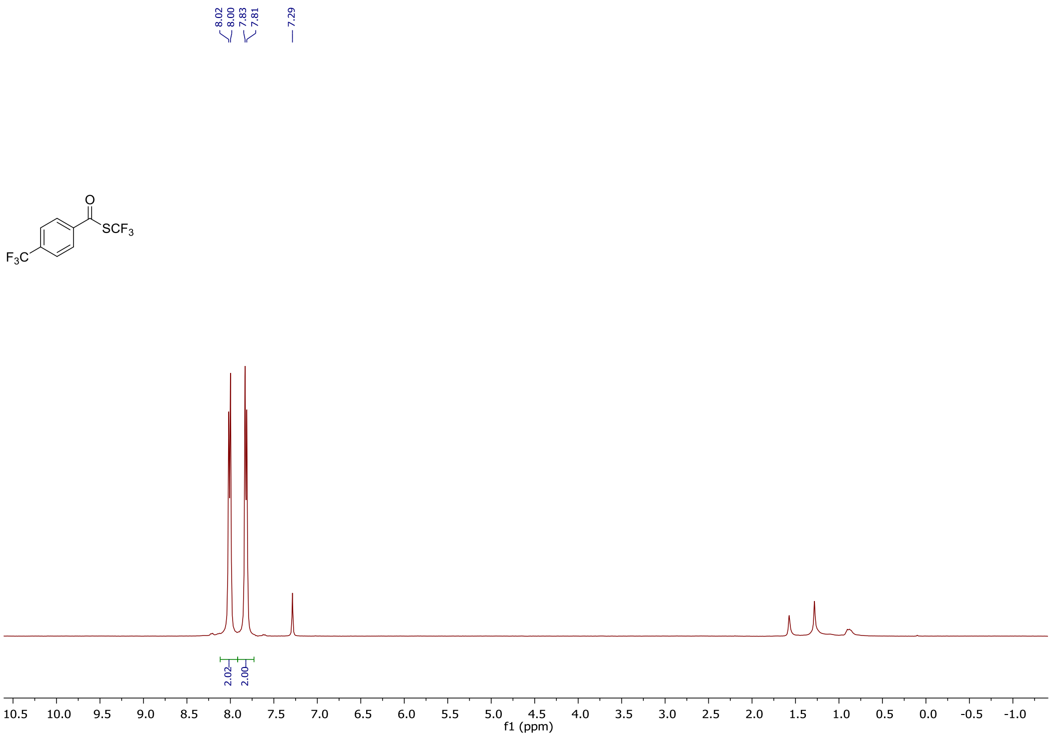

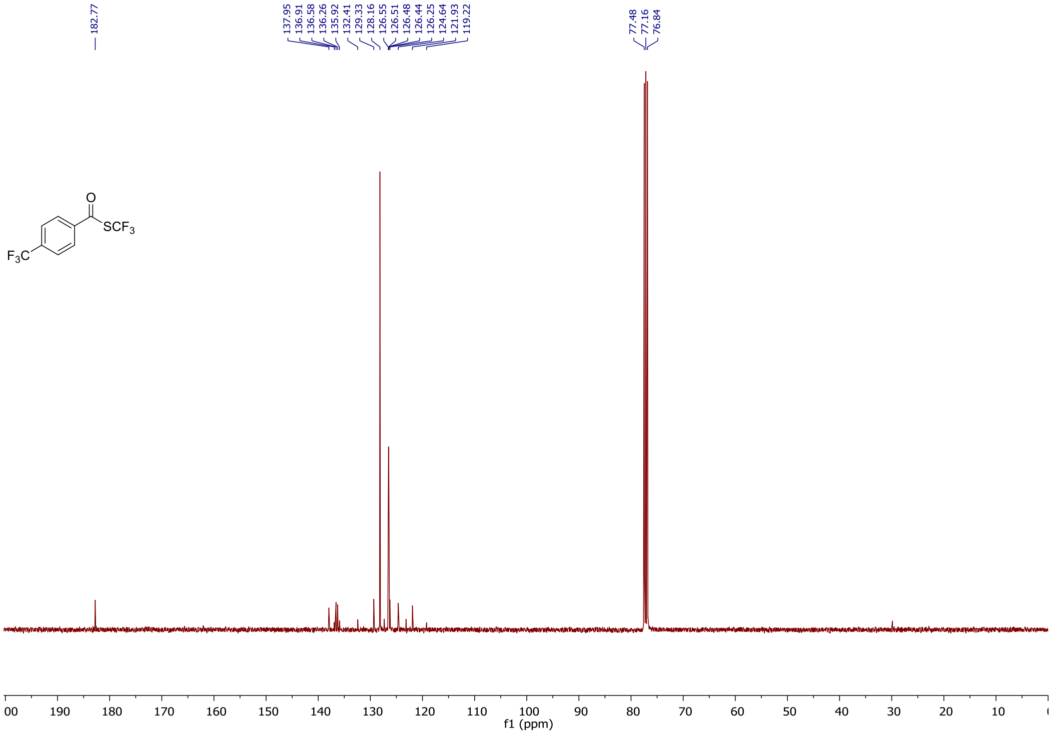

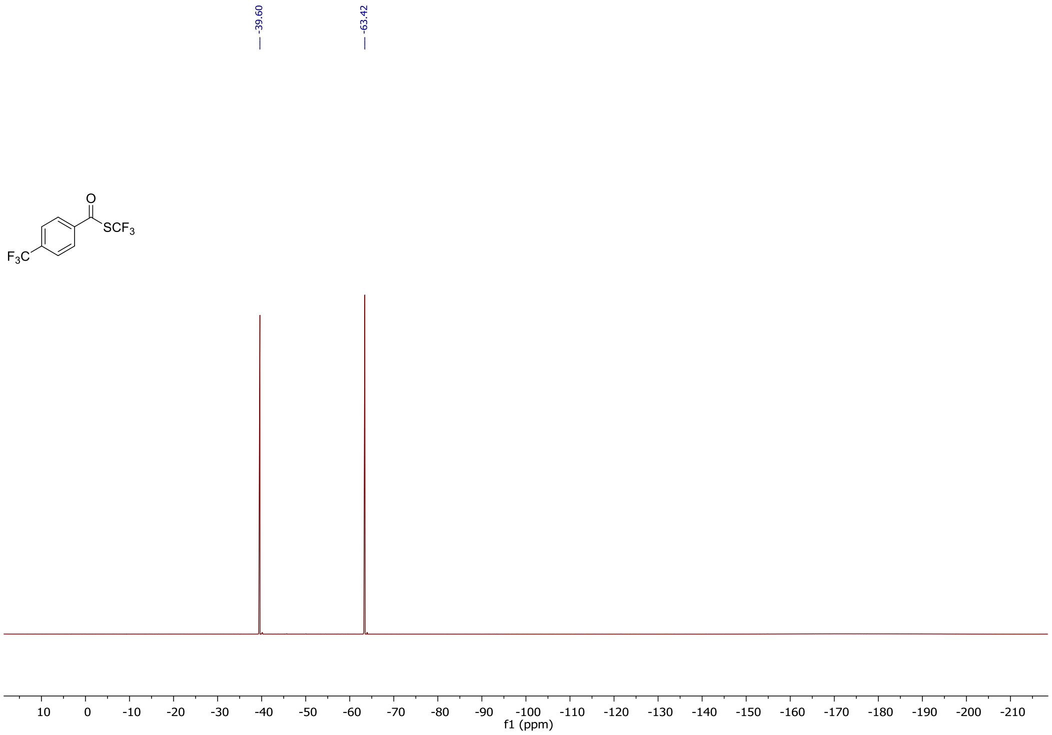
-39.60
-63.42
O
SCF3
F3C
10
0
-10
-20
-30
-40
-50
-60
-70
-80
-90
-100
-110
-120
-130
-140
-150
-160
-170
-180
-190
-200
-210
f1 (ppm)

Figure S15. NMR spectra of 3o

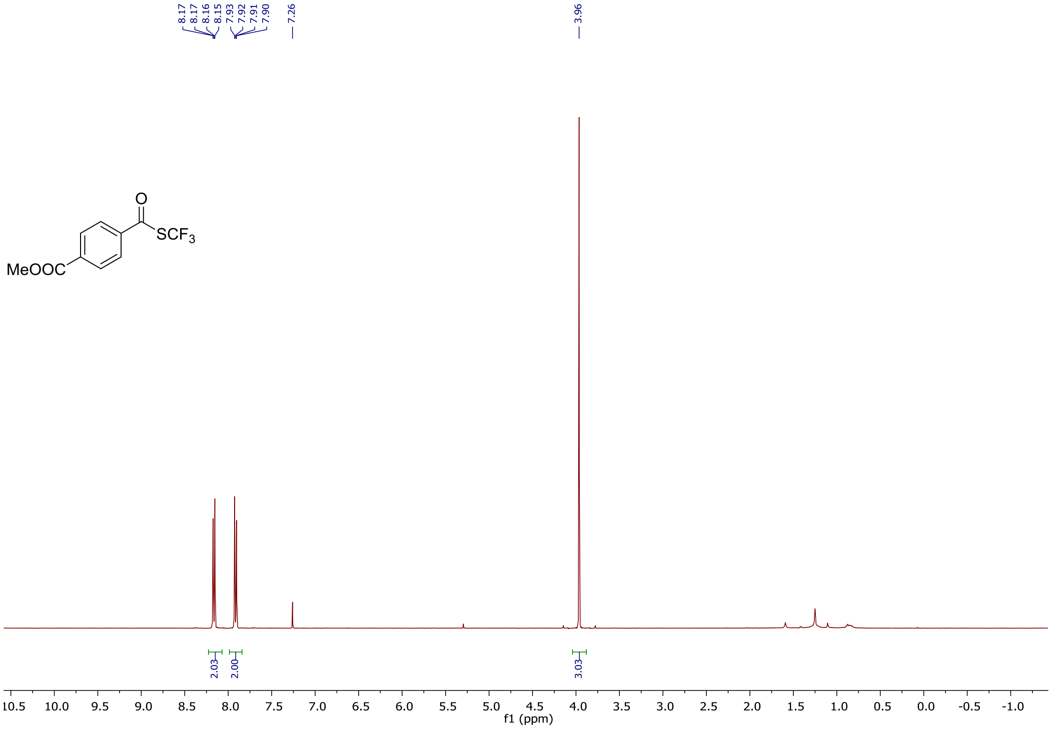

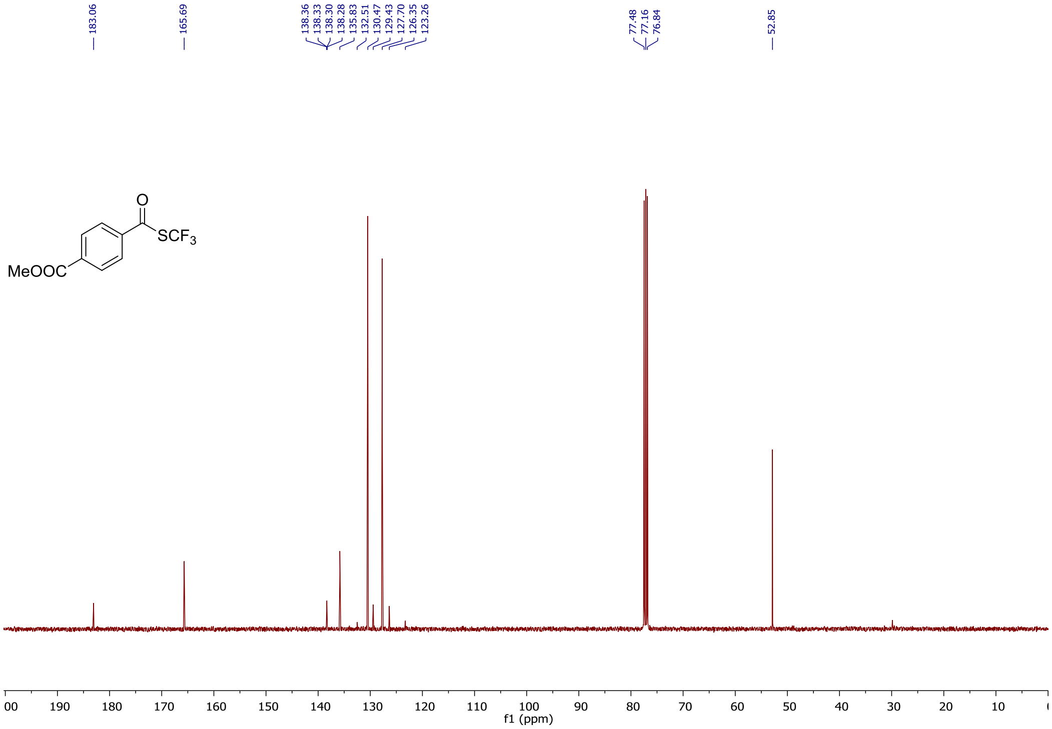

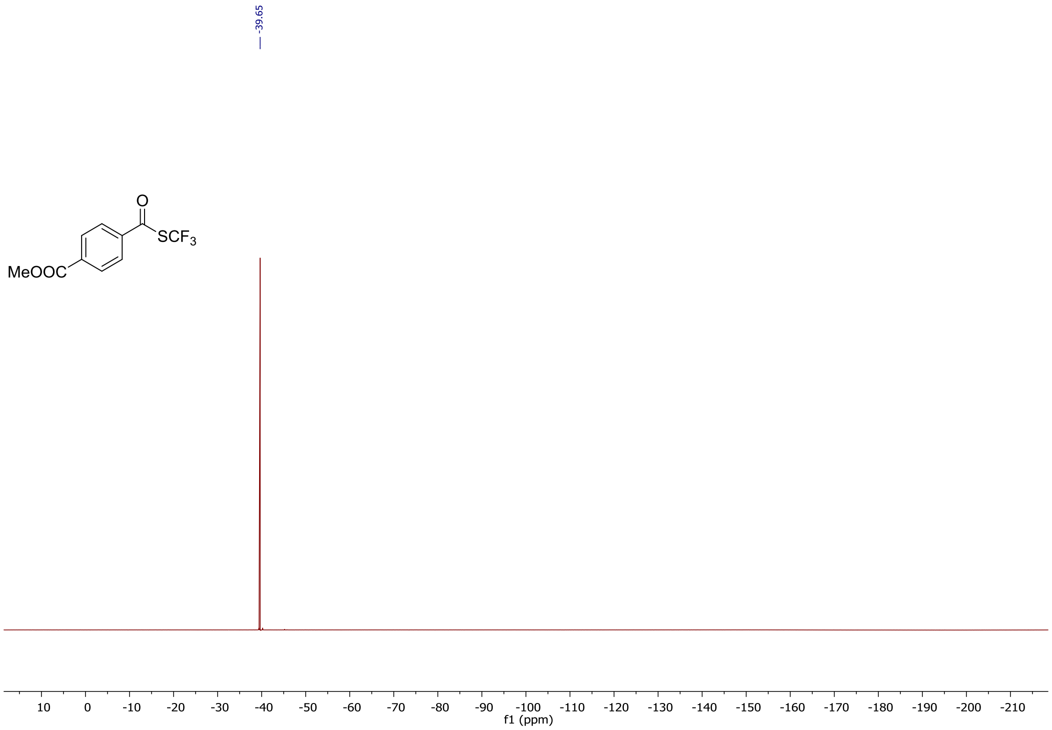

-39.65
MeOOC
O
SCF3
10
0
-10
-20
-30
-40
-50
-60
-70
-80
-90
-100
-110
-120
-130
-140
-150
-160
-170
-180
-190
-200
-210
f1 (ppm)

Figure S16. NMR spectra of 3p

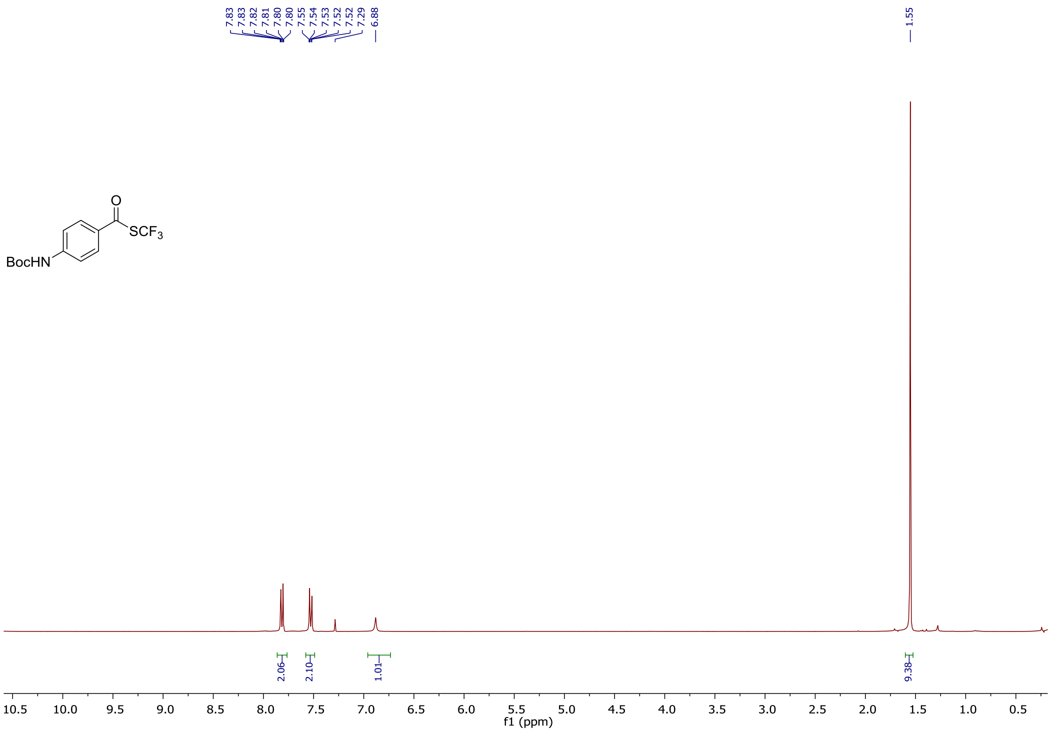

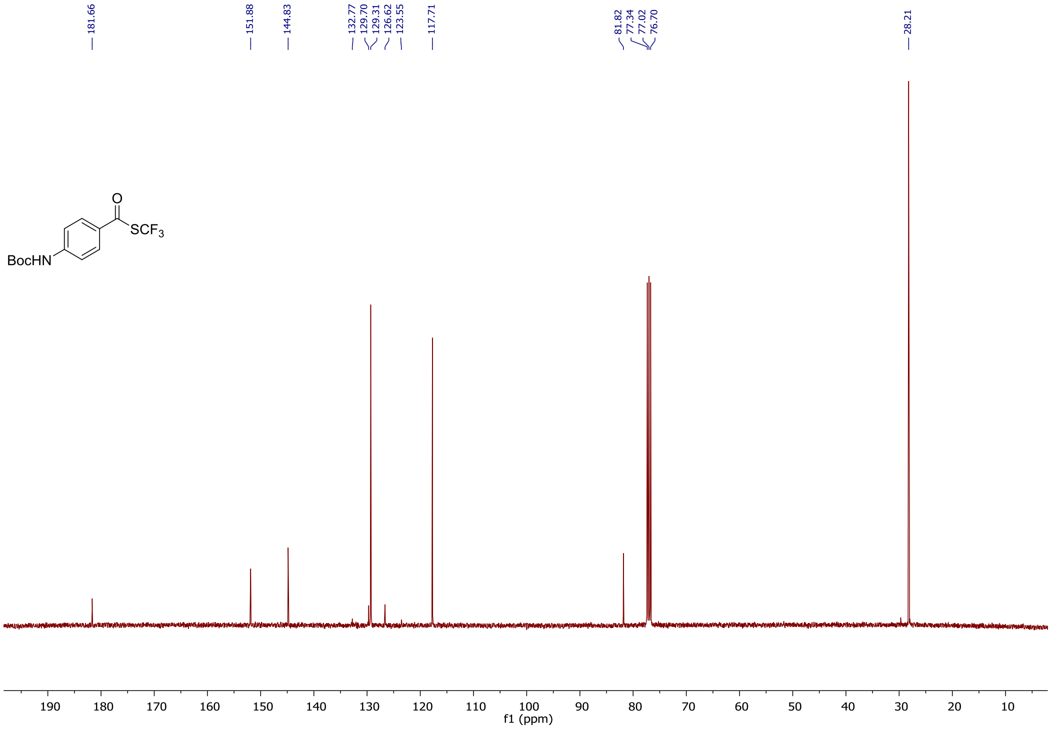

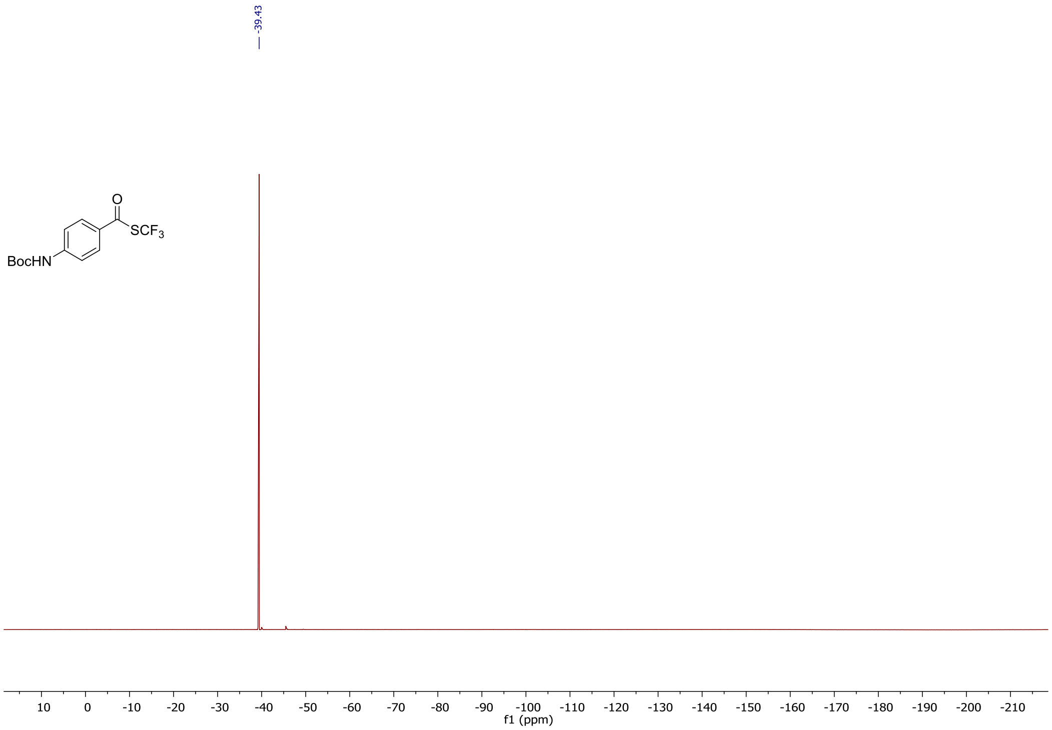

-39.43
O
SCF3
BocHN
10 0 -10 -20 -30 -40 -50 -60 -70 -80 -90 -100 -110 -120 -130 -140 -150 -160 -170 -180 -190 -200 -210
f1 (ppm)

Figure S17. NMR spectra of 3q

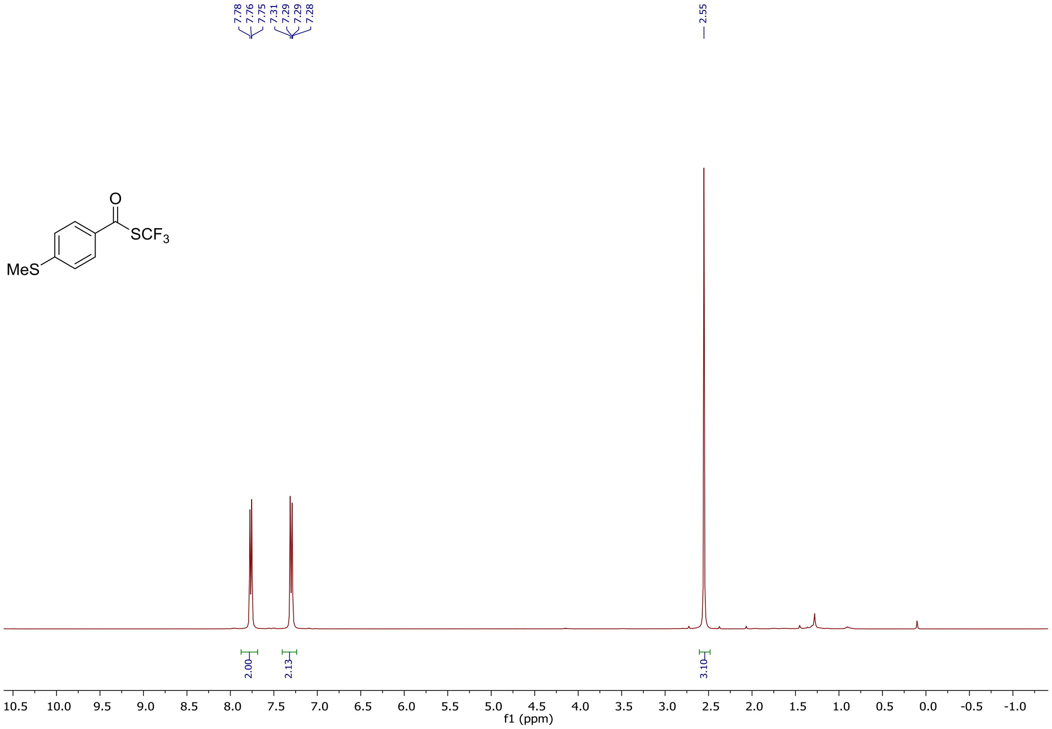

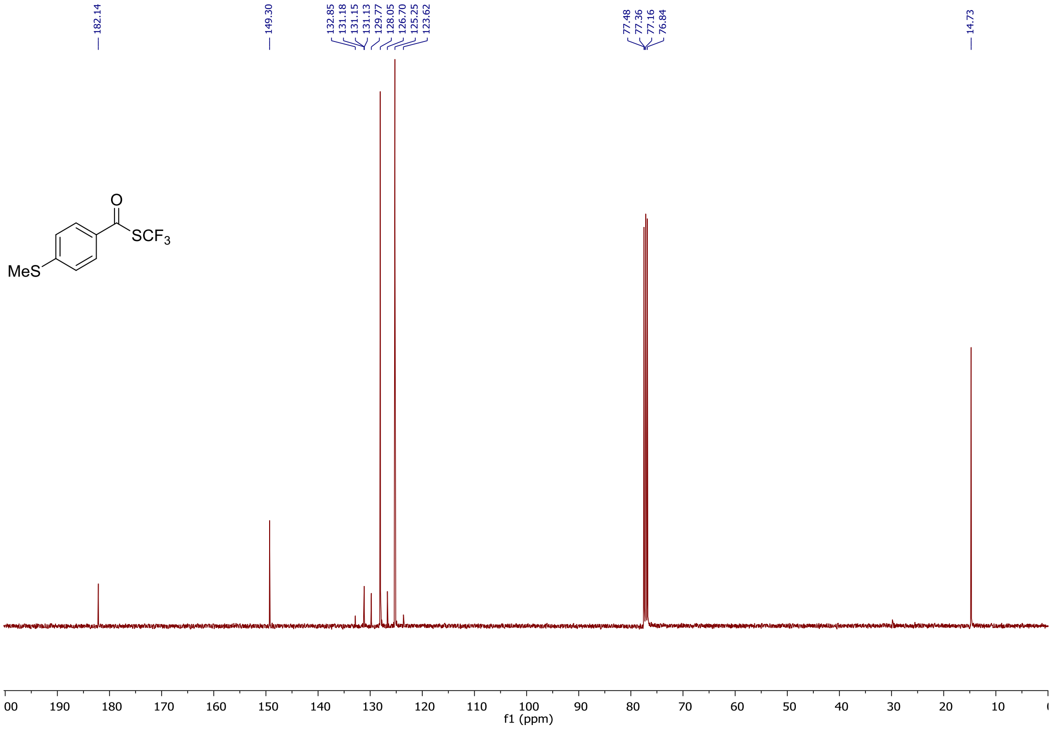

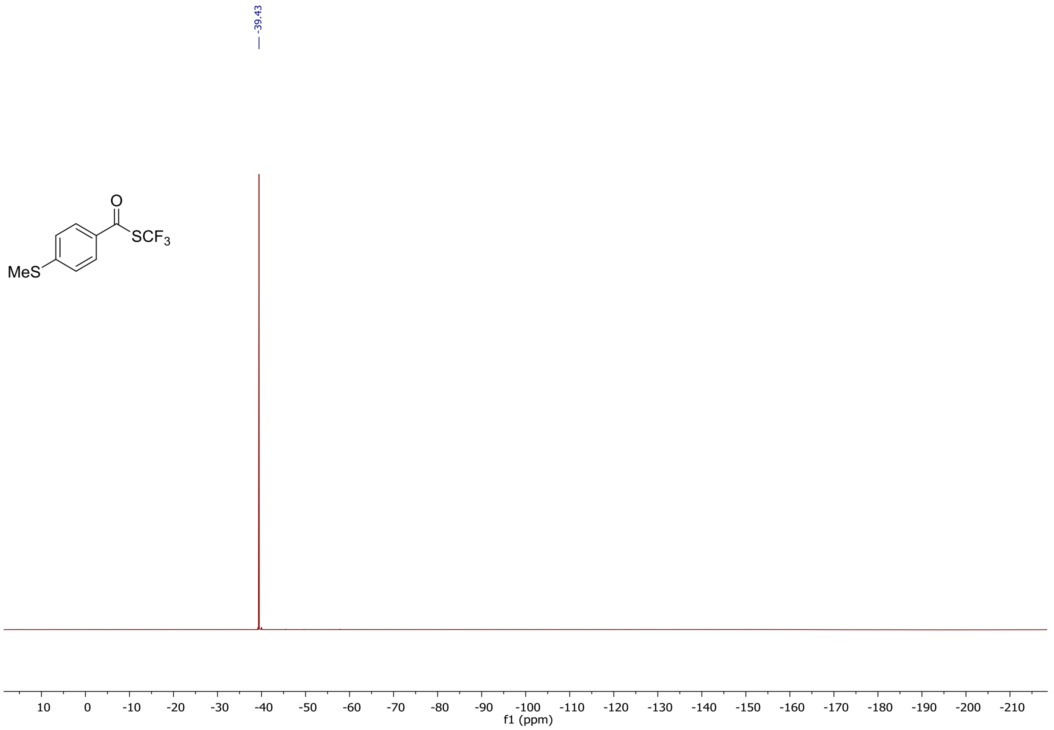

-39.43
O
SCF3
MeS
10 0 -10 -20 -30 -40 -50 -60 -70 -80 -90 -100 -110 -120 -130 -140 -150 -160 -170 -180 -190 -200 -210
f1 (ppm)

Figure S18. NMR spectra of 3r

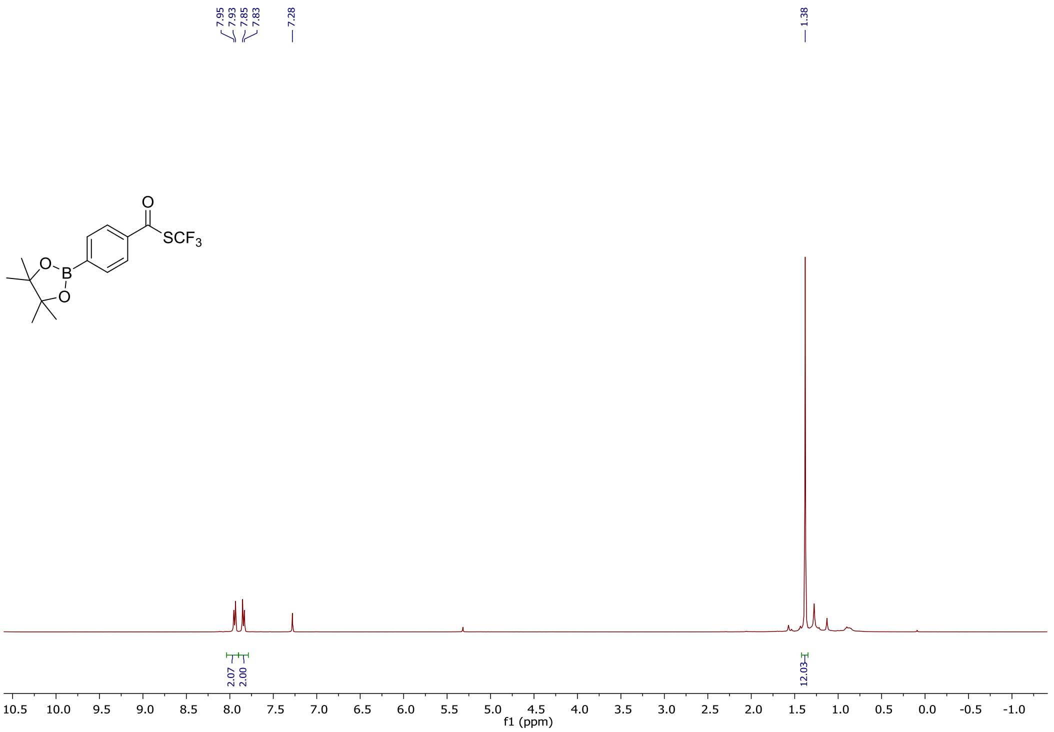

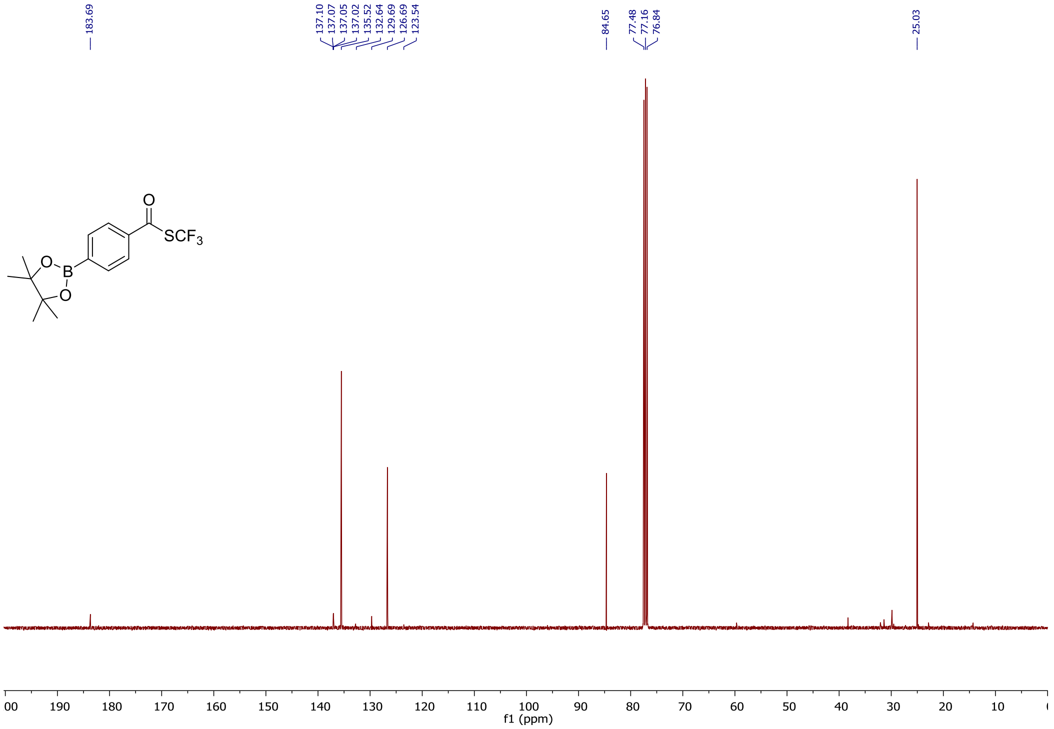

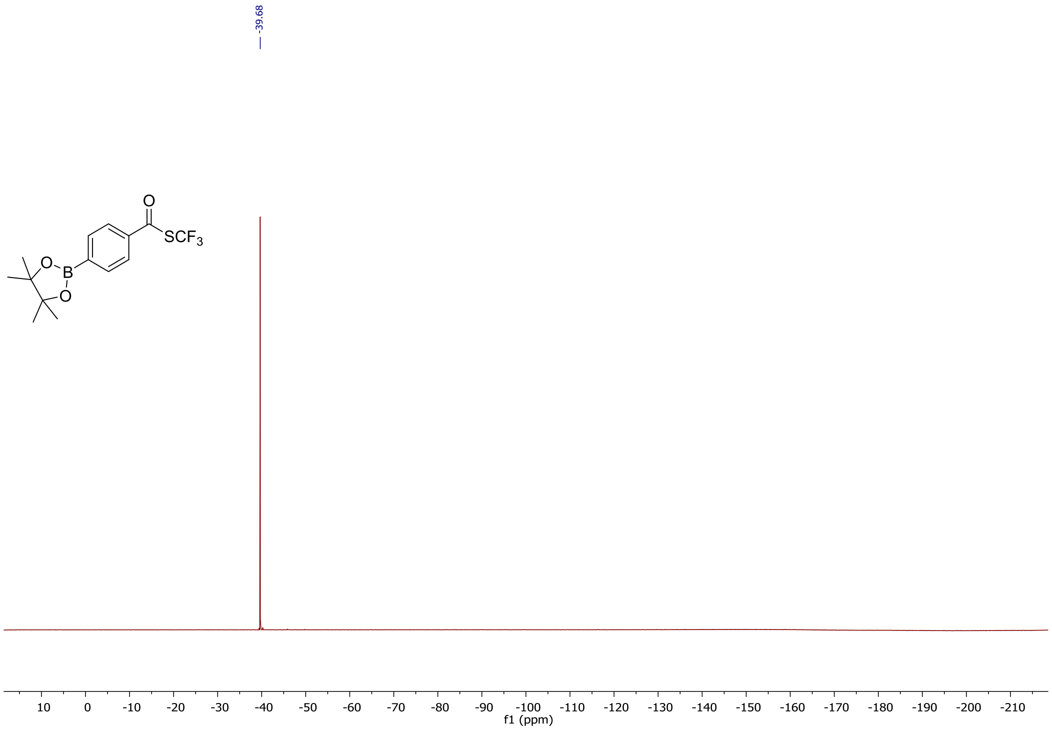

-39.68
SCF3
O
B
10
0
-10
-20
-30
-40
-50
-60
-70
-80
-90
-100
-110
-120
-130
-140
-150
-160
-170
-180
-190
-200
-210
f1 (ppm)

Figure S19. NMR spectra of 3s

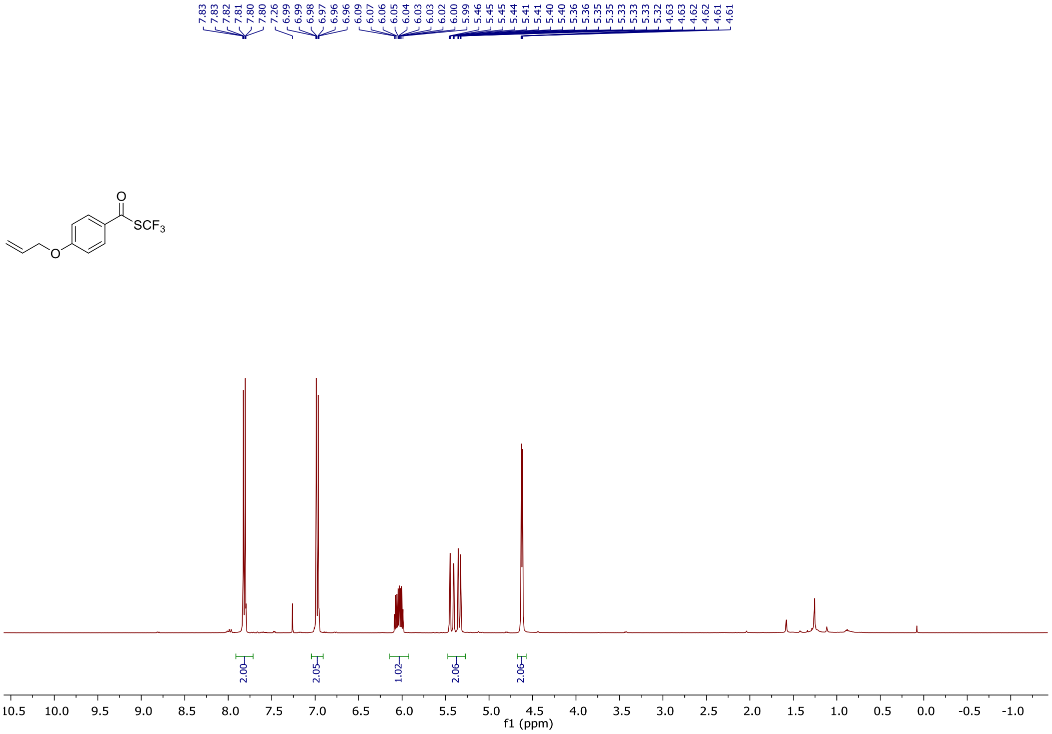

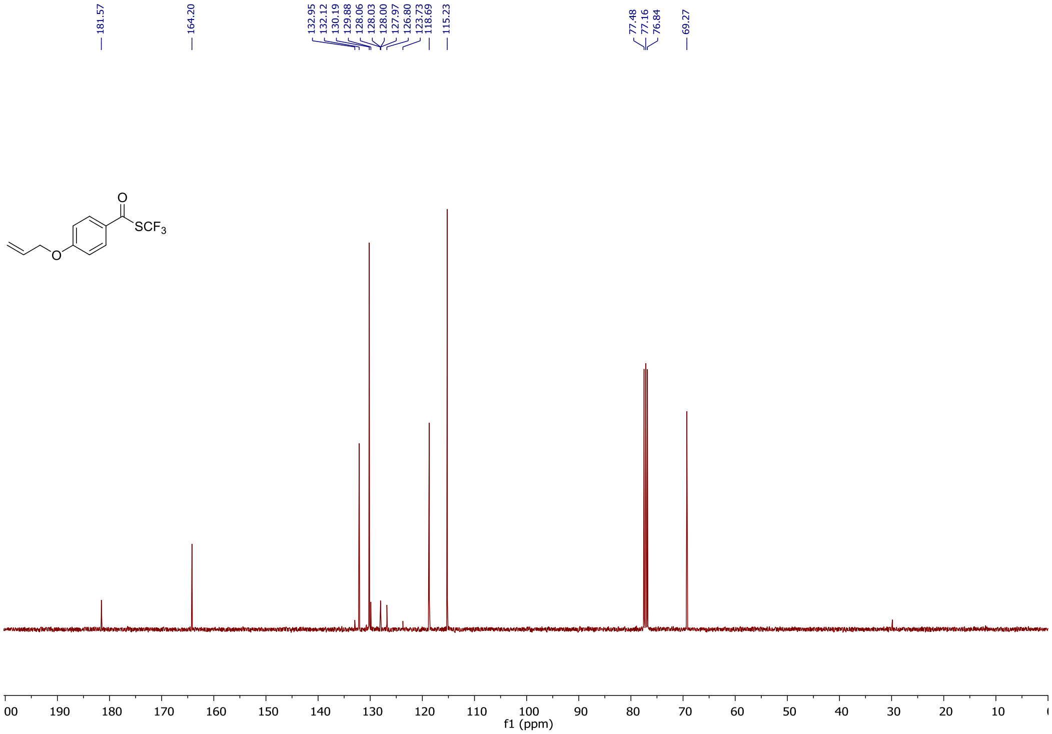

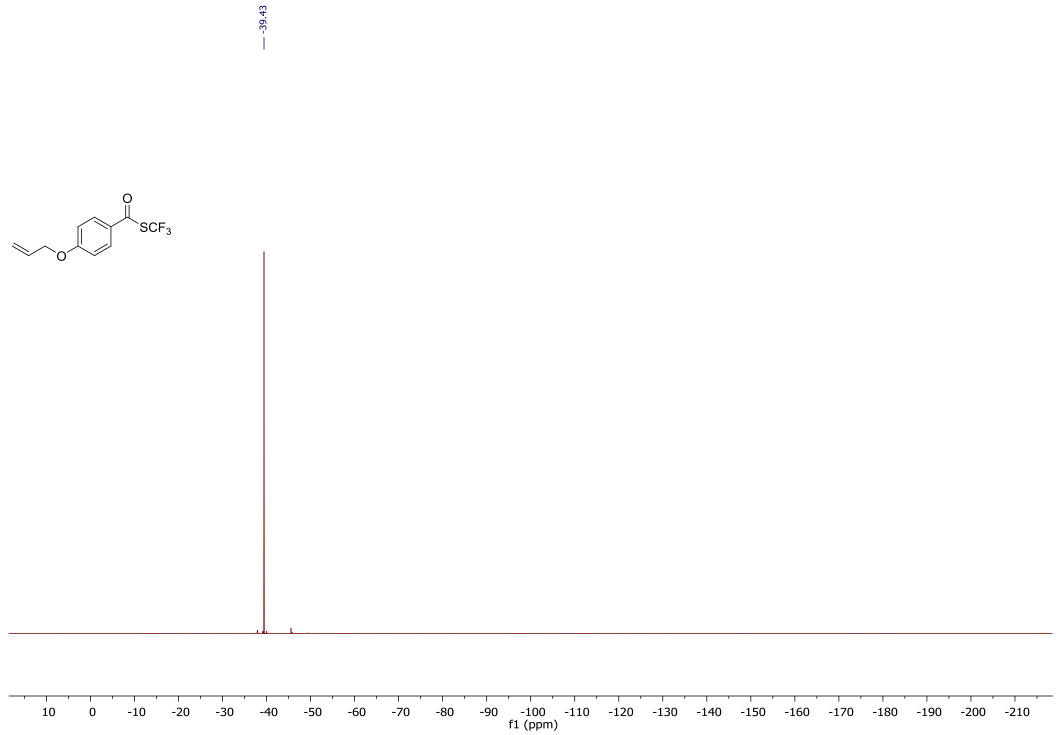

-39.43
O
SCF3
O
10 0 -10 -20 -30 -40 -50 -60 -70 -80 -90 -100 -110 -120 -130 -140 -150 -160 -170 -180 -190 -200 -210
f1 (ppm)

Figure S20. NMR spectra of 3t

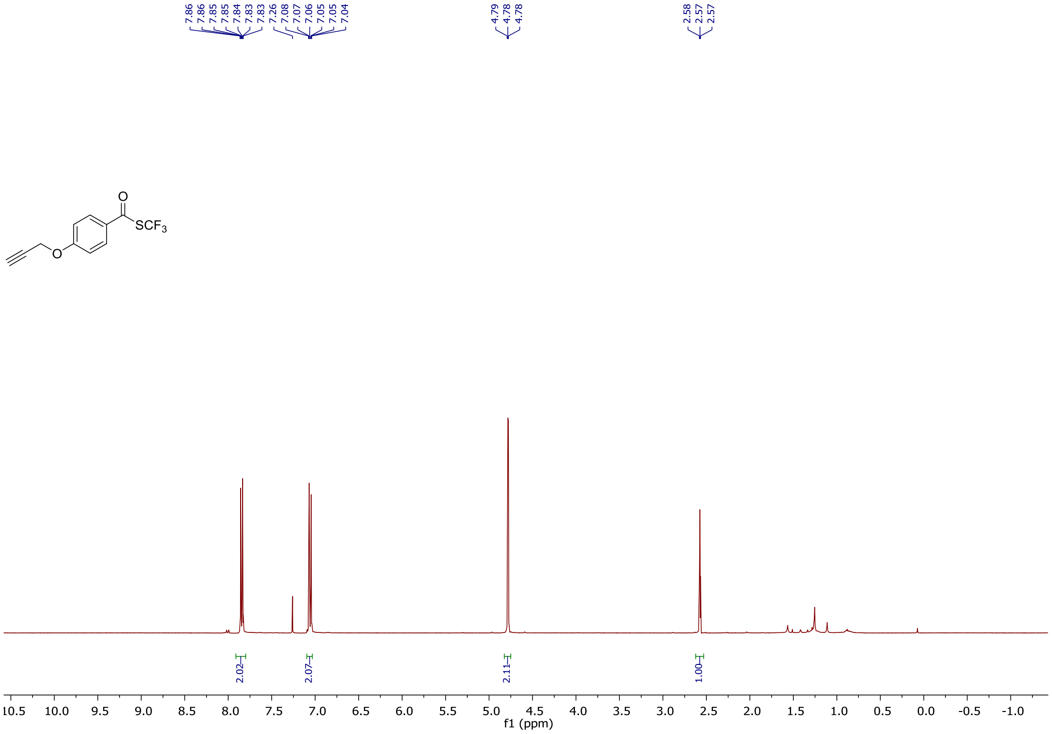

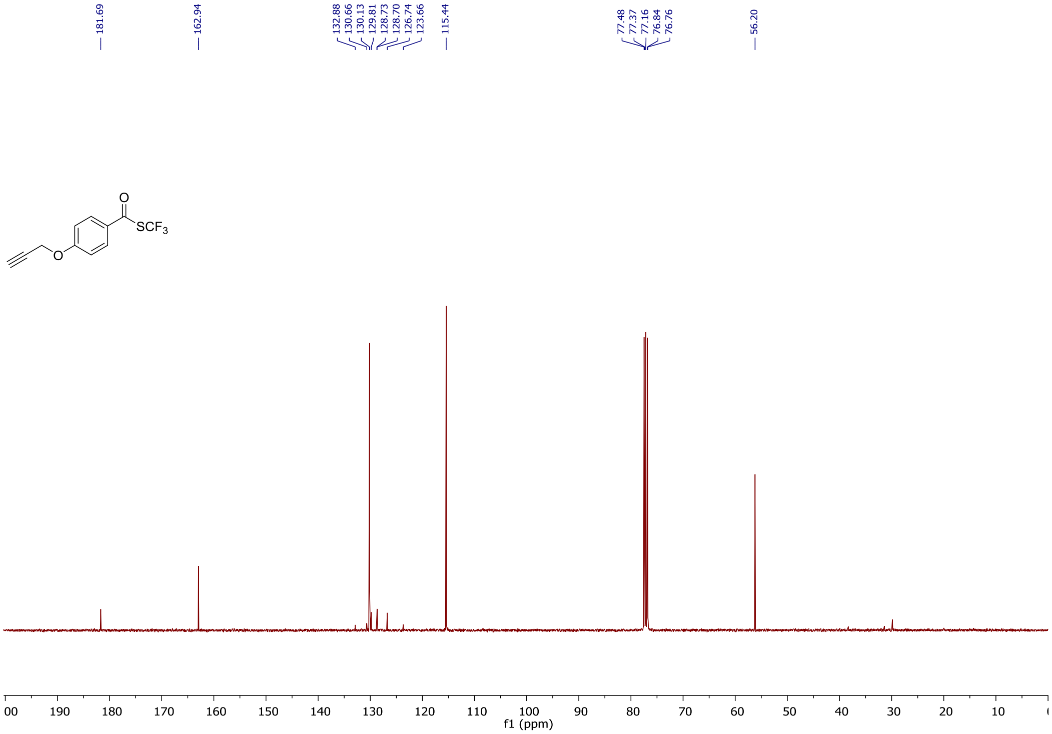

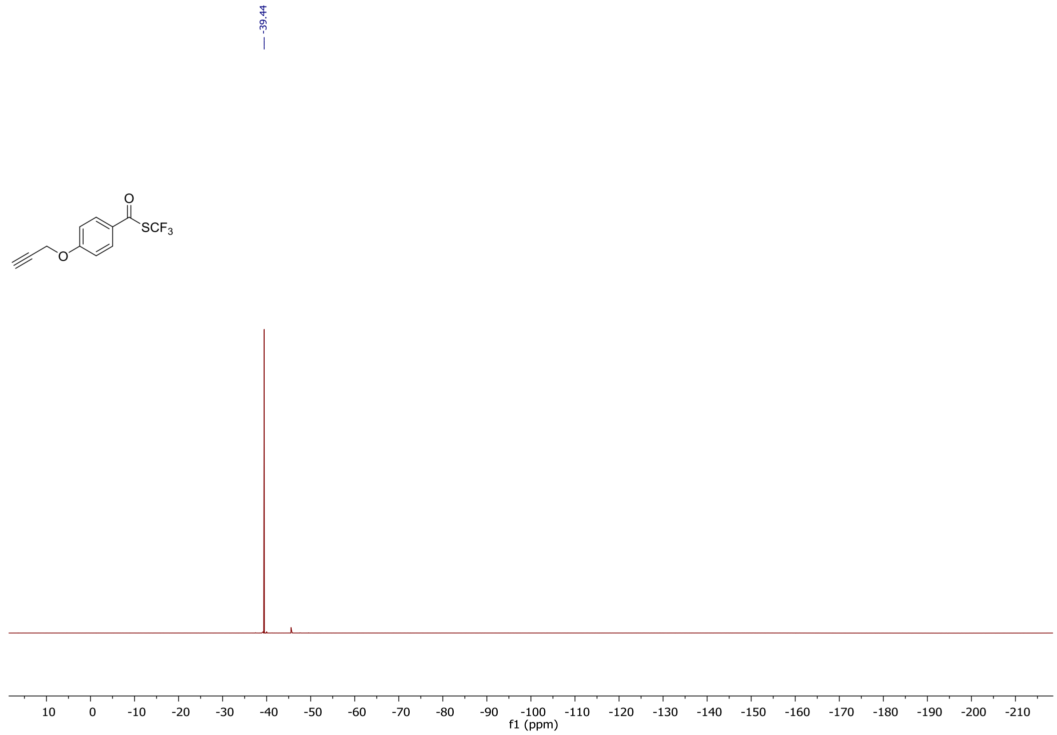
-39.44
O
SCF3
O
10
0
-10
-20
-30
-40
-50
-60
-70
-80
-90
-100
-110
-120
-130
-140
-150
-160
-170
-180
-190
-200
-210
f1 (ppm)

Figure S21. NMR spectra of 3u

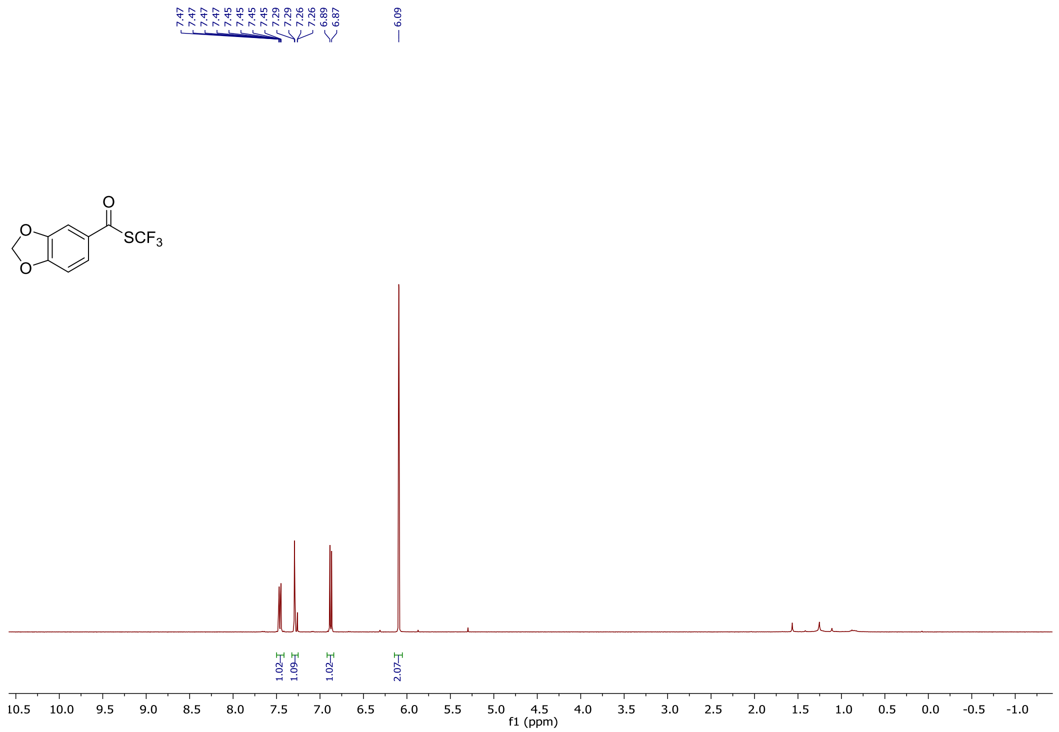

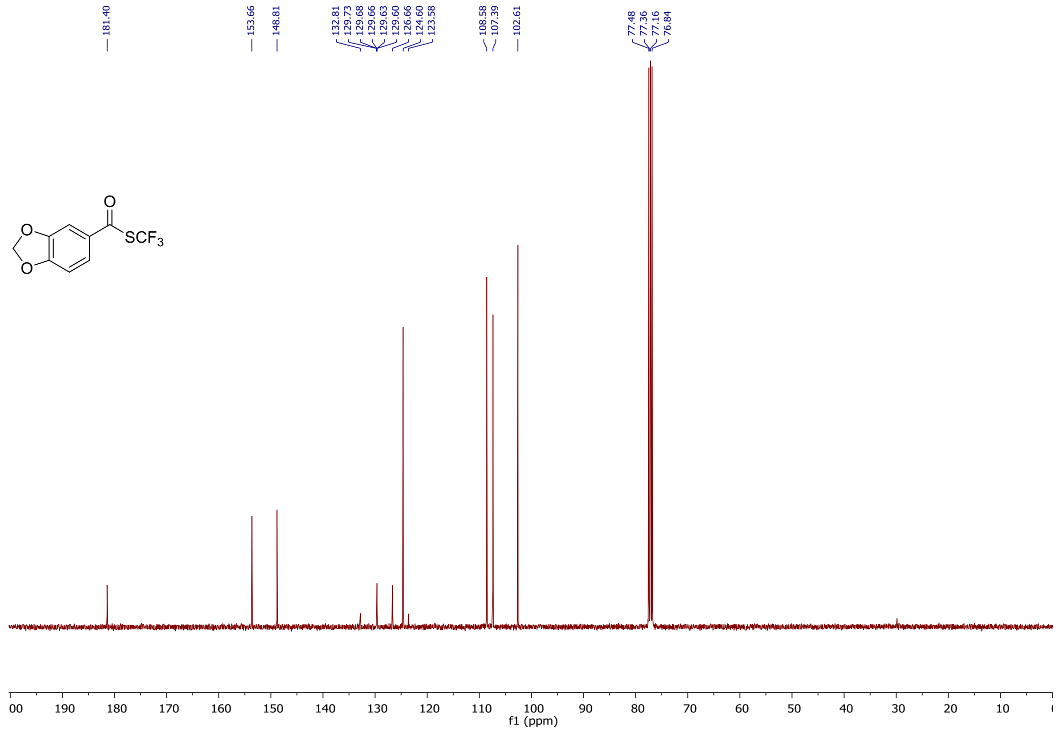

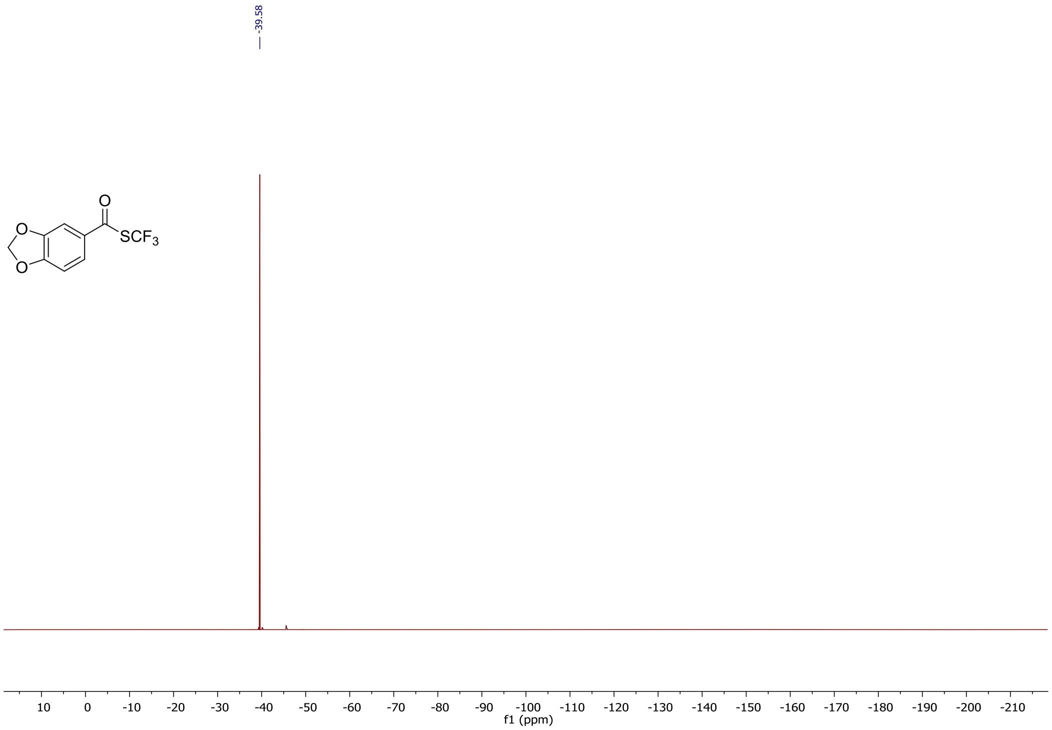
-39.58
SCF3
O
10
0
-10
-20
-30
-40
-50
-60
-70
-80
-90
-100
-110
-120
-130
-140
-150
-160
-170
-180
-190
-200
-210
f1 (ppm)

Figure S22. NMR spectra of 3v

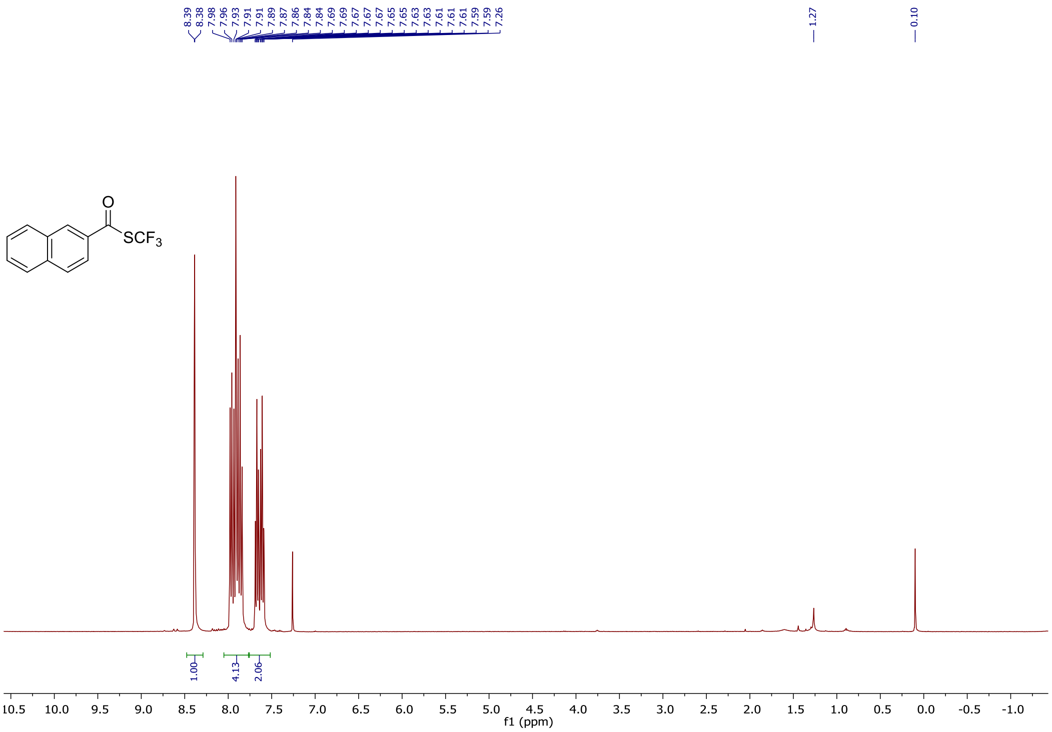

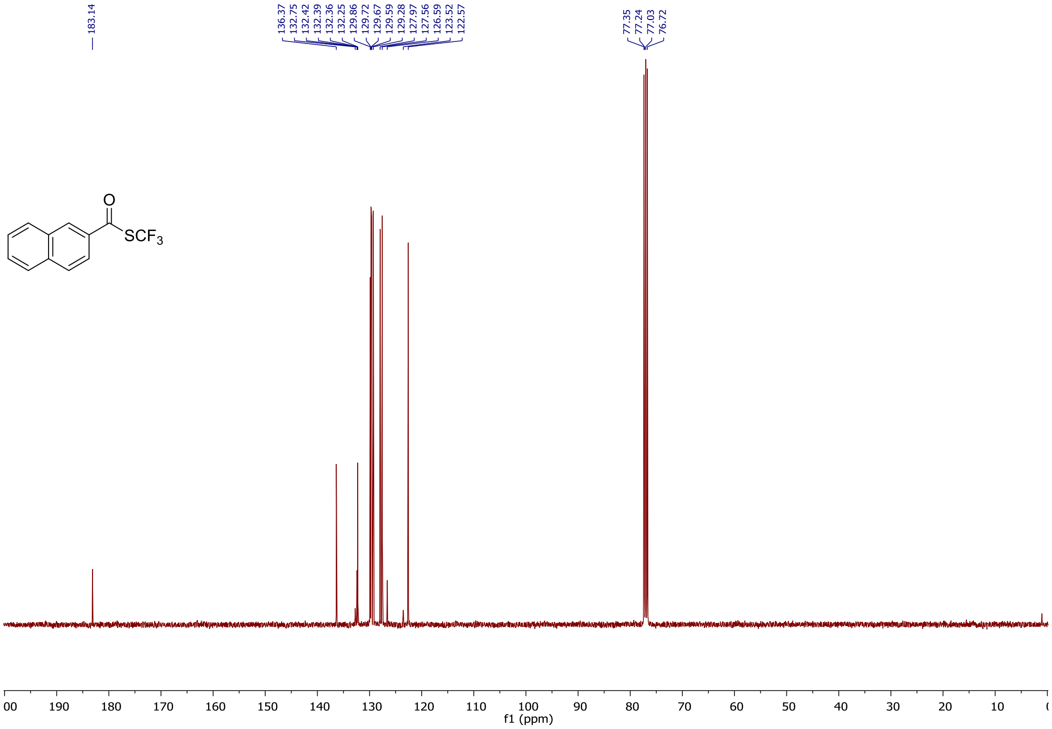

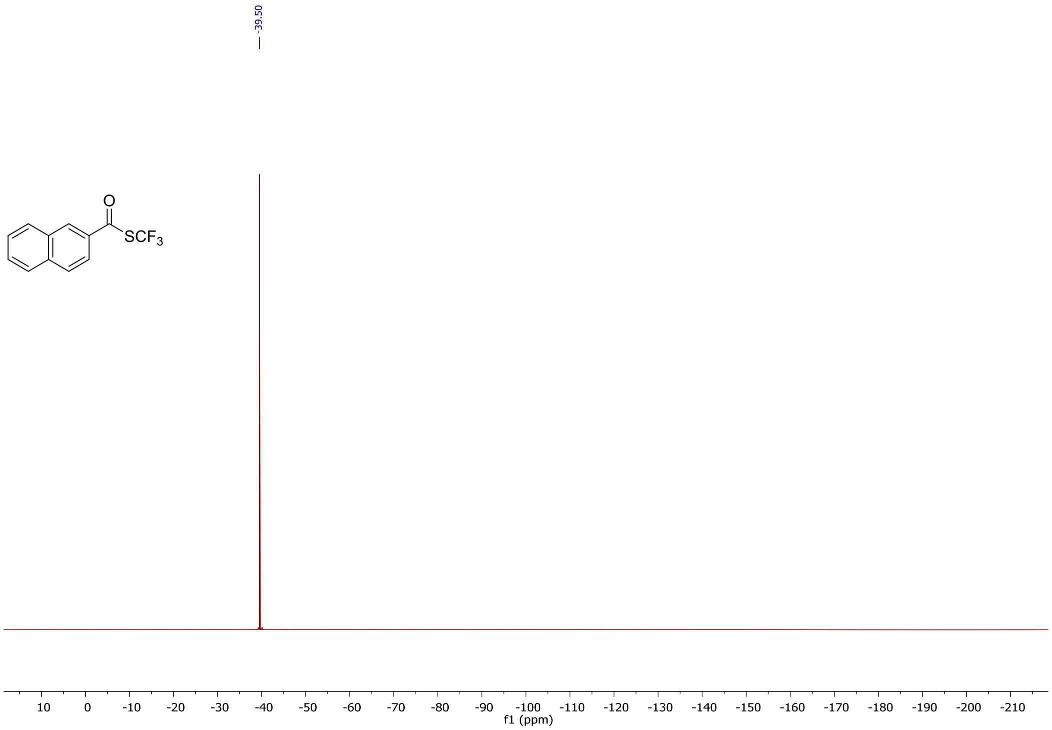

-39.50
SCF3
O
10 0 -10 -20 -30 -40 -50 -60 -70 -80 -90 -100 -110 -120 -130 -140 -150 -160 -170 -180 -190 -200 -210
f1 (ppm)

Figure S23. NMR spectra of 3w

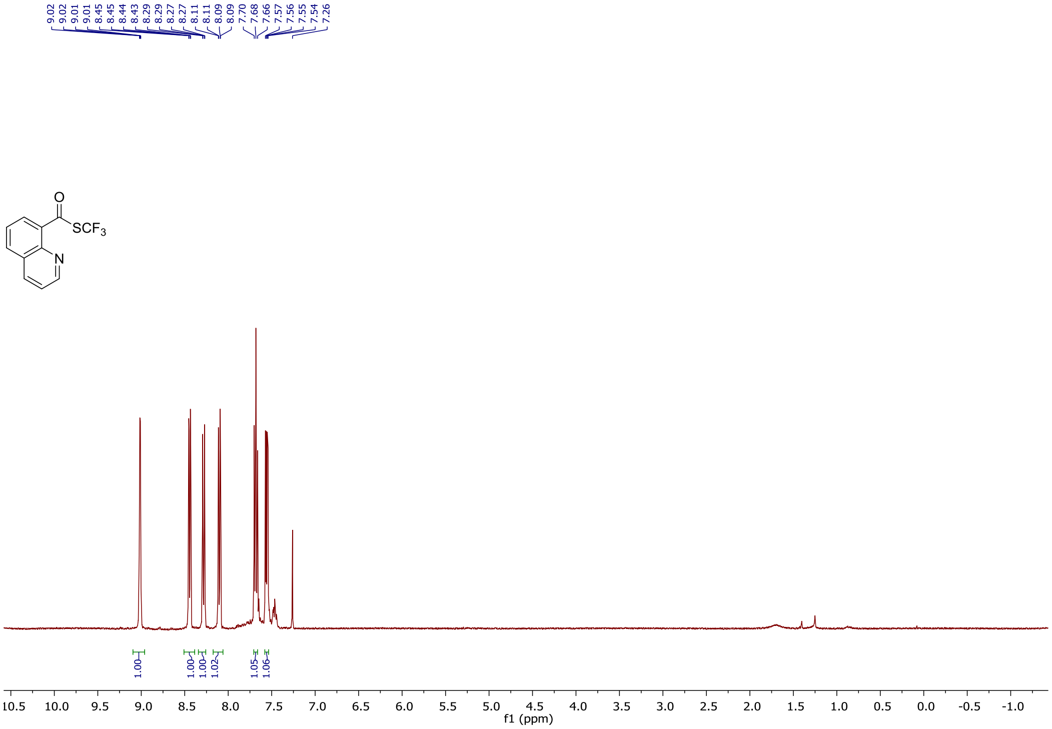

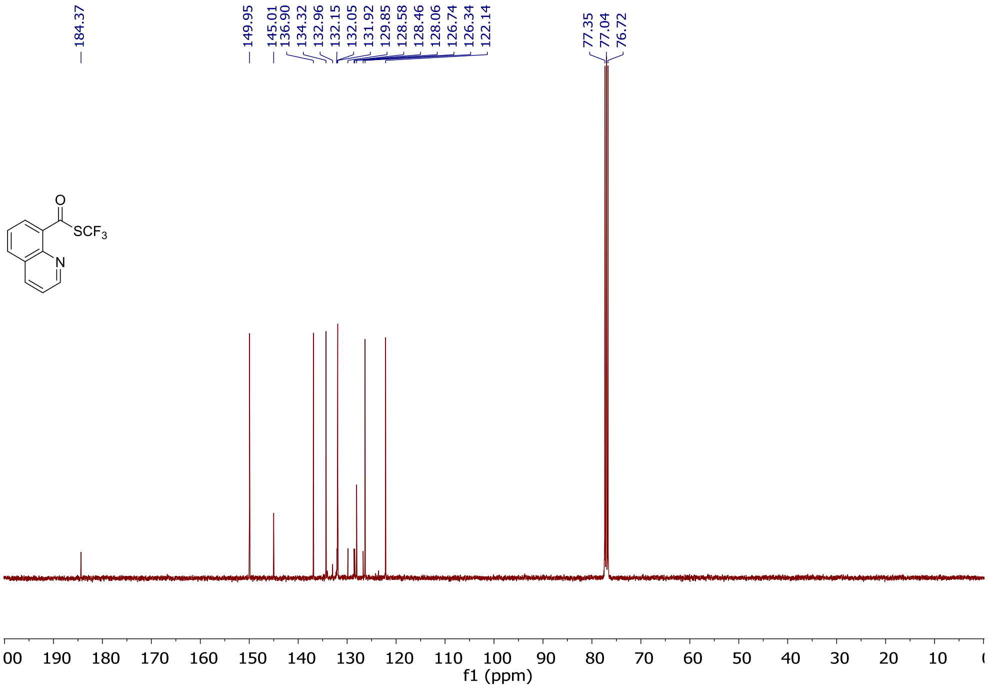

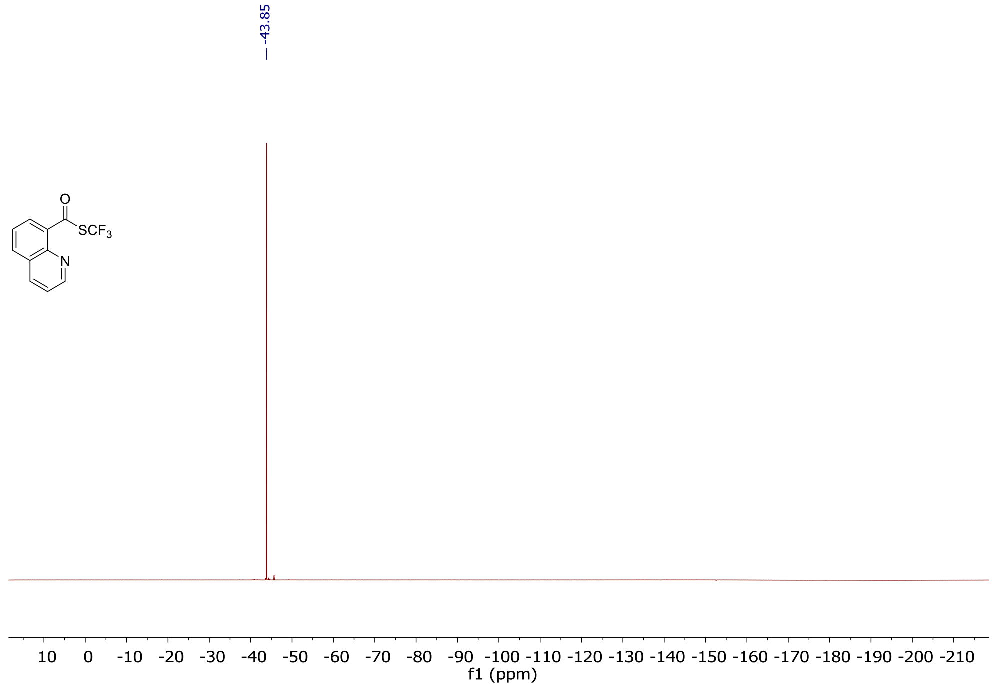

-43.85
10 0 -10 -20 -30 -40 -50 -60 -70 -80 -90 -100 -110 -120 -130 -140 -150 -160 -170 -180 -190 -200 -210
f1 (ppm)

Figure S24. NMR spectra of 3x

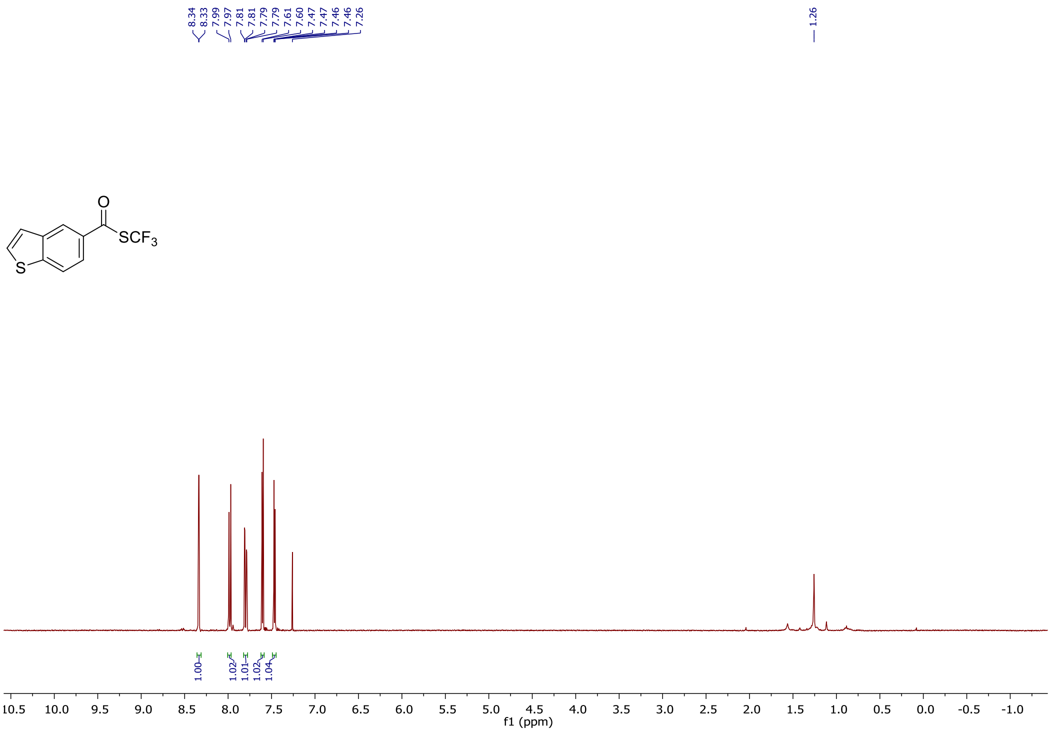

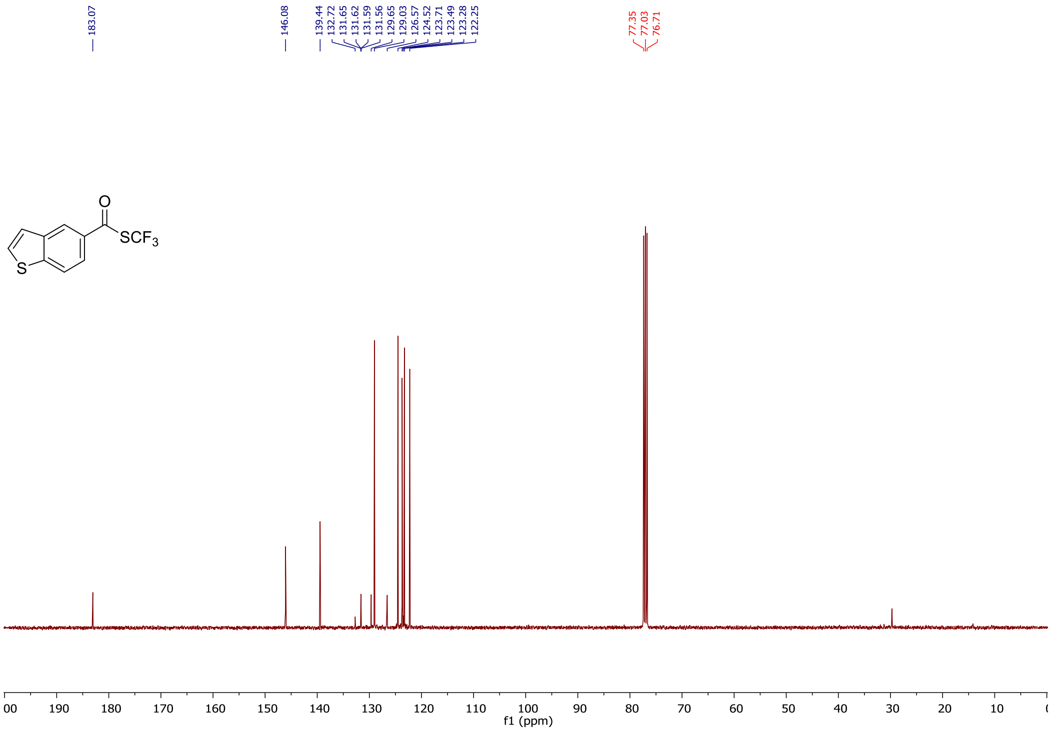

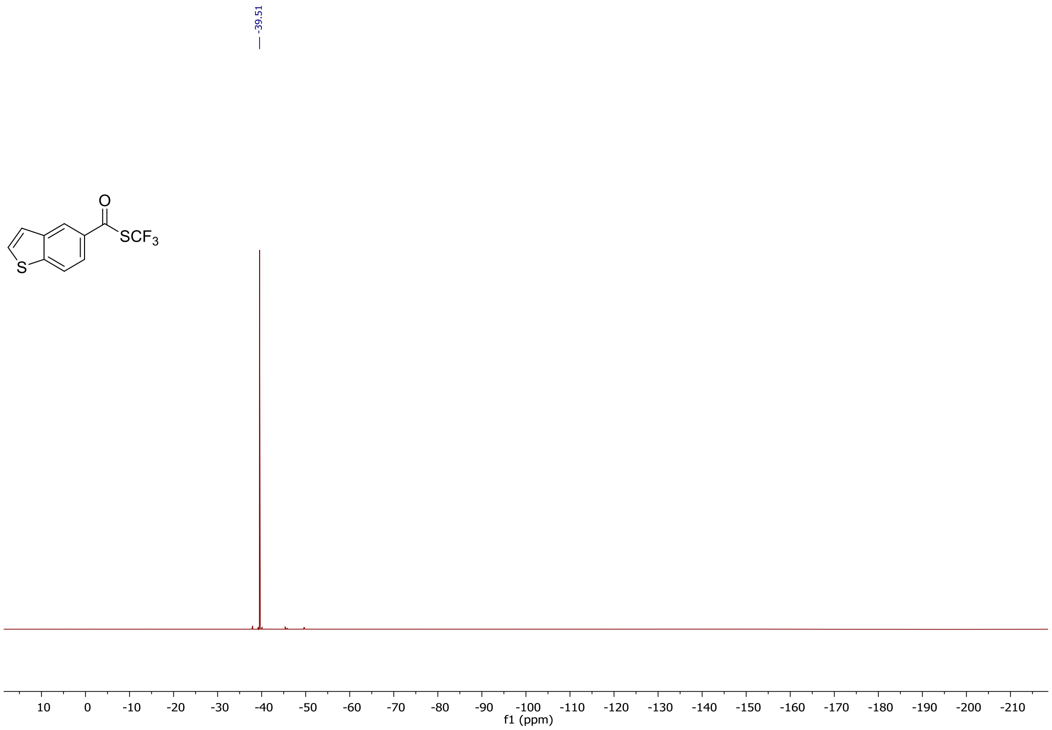

-39.51
SCF3
O
S
10 0 -10 -20 -30 -40 -50 -60 -70 -80 -90 -100 -110 -120 -130 -140 -150 -160 -170 -180 -190 -200 -210
f1 (ppm)

Figure S25. **NMR spectra of 4a**

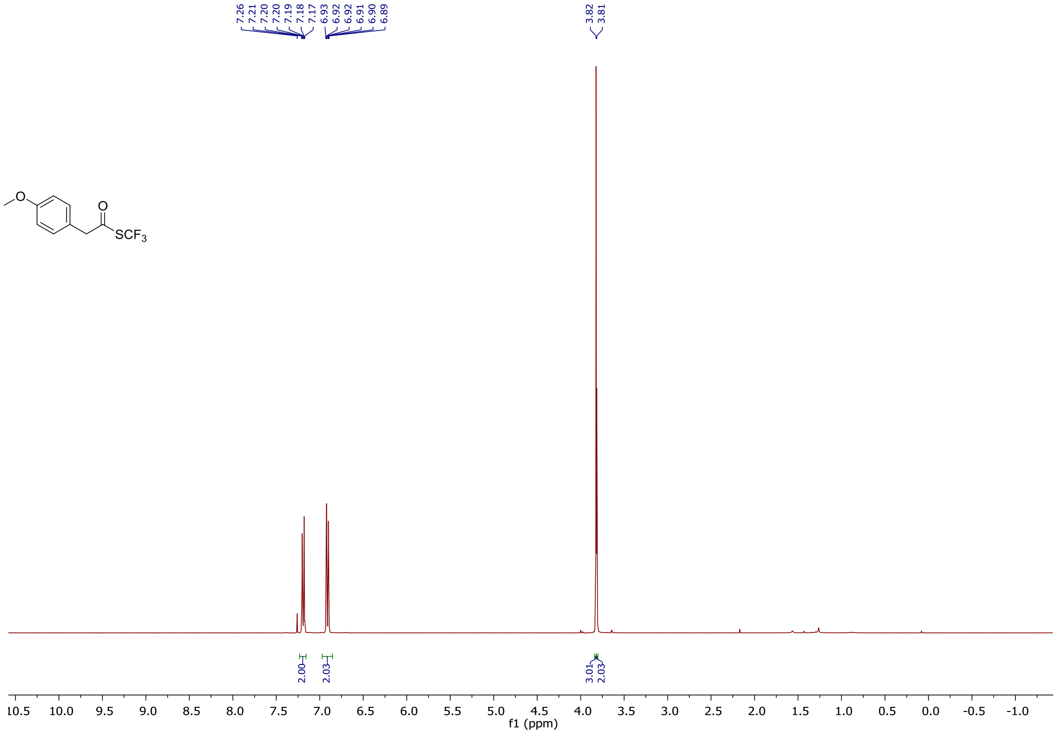

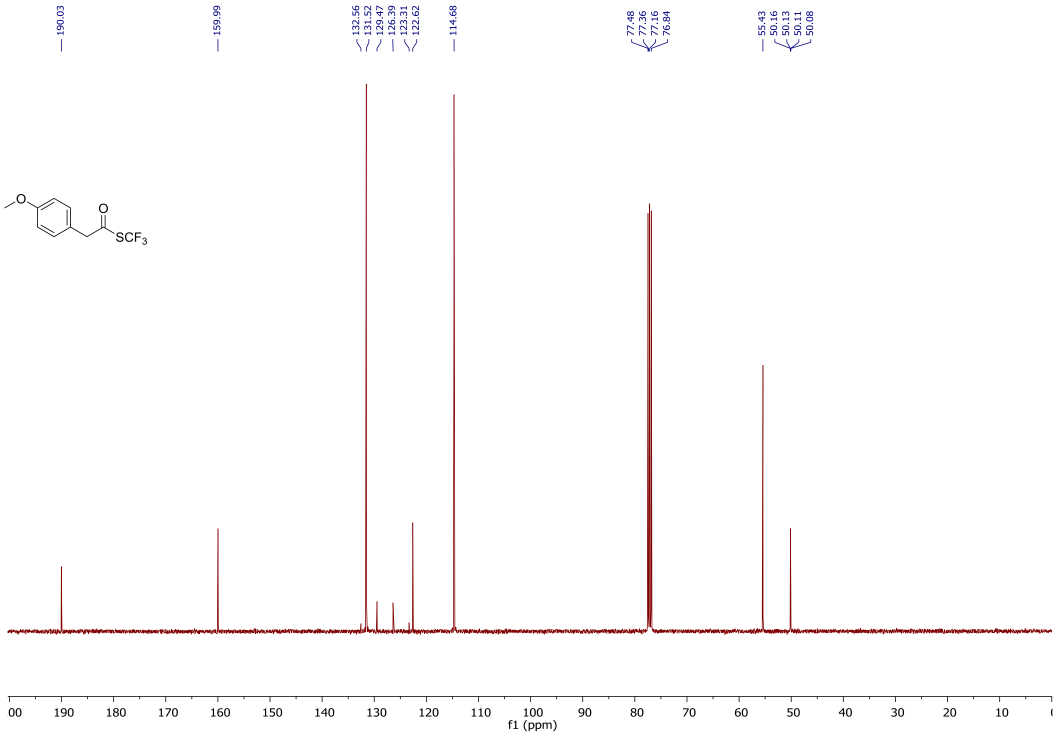

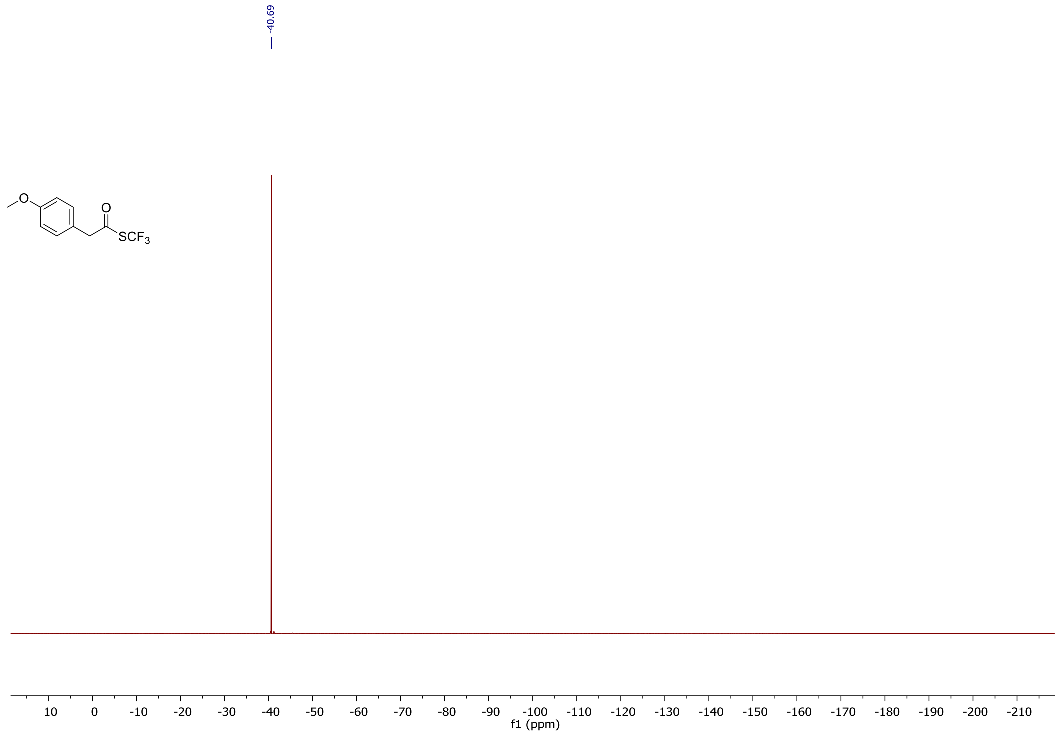
-40.69
SCF3
10 0 -10 -20 -30 -40 -50 -60 -70 -80 -90 -100 -110 -120 -130 -140 -150 -160 -170 -180 -190 -200 -210
f1 (ppm)

Figure S26. NMR spectra of 4b

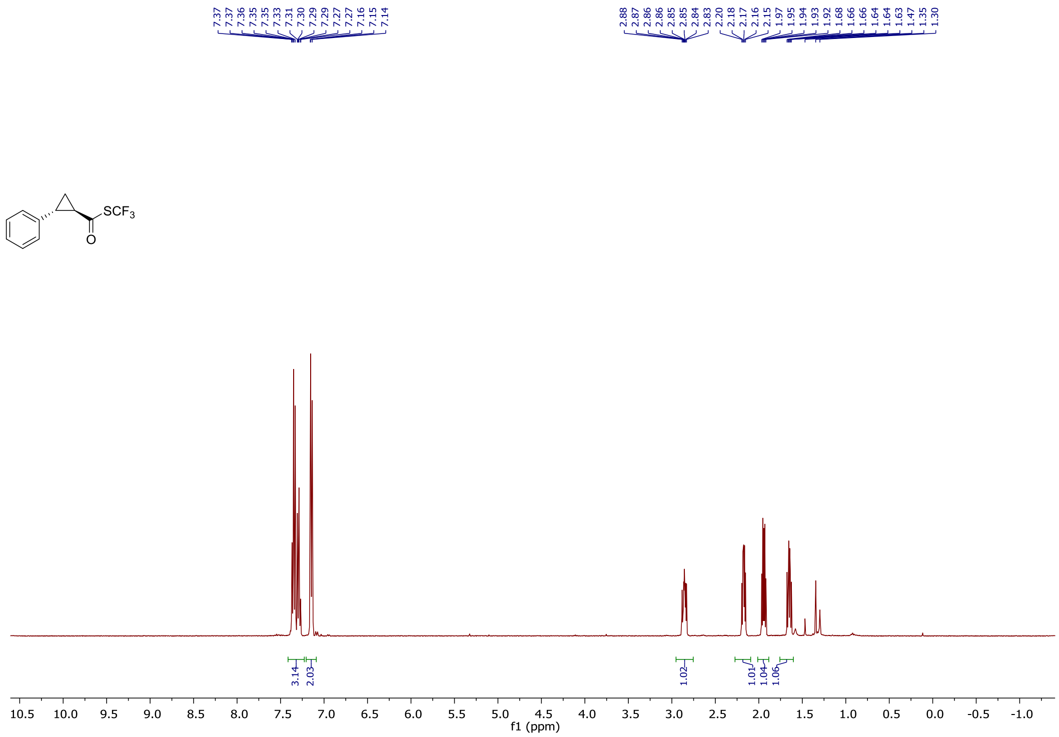

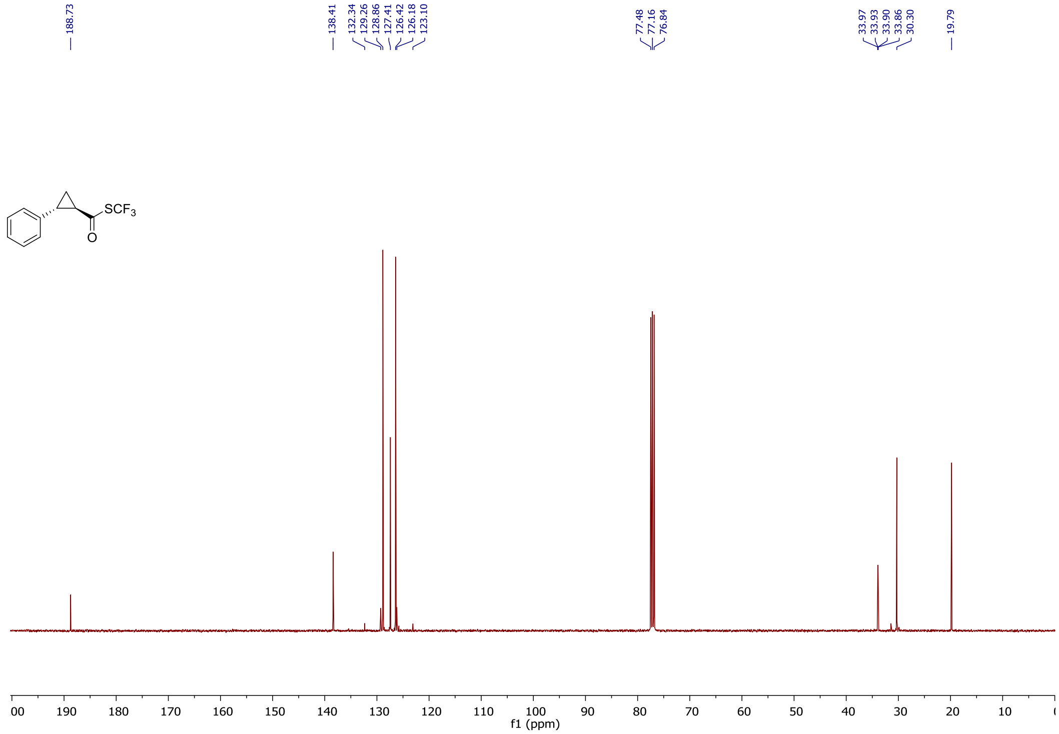

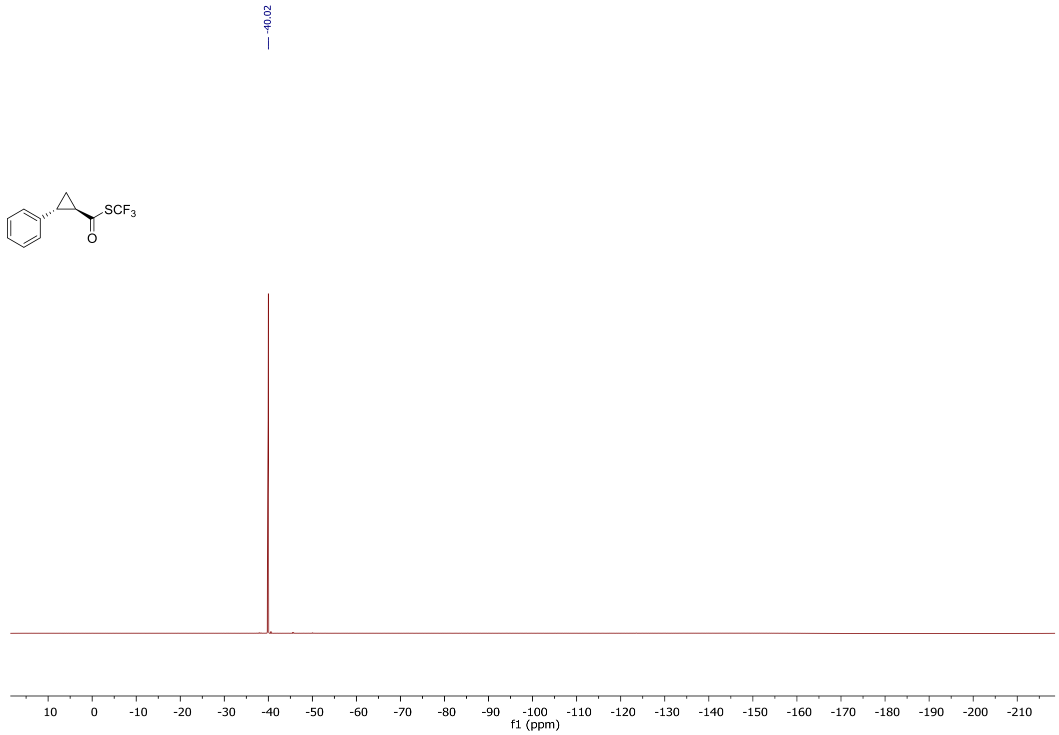

-40.02
SCF3
O
10 0 -10 -20 -30 -40 -50 -60 -70 -80 -90 -100 -110 -120 -130 -140 -150 -160 -170 -180 -190 -200 -210
f1 (ppm)

Figure S27. NMR spectra of 4c

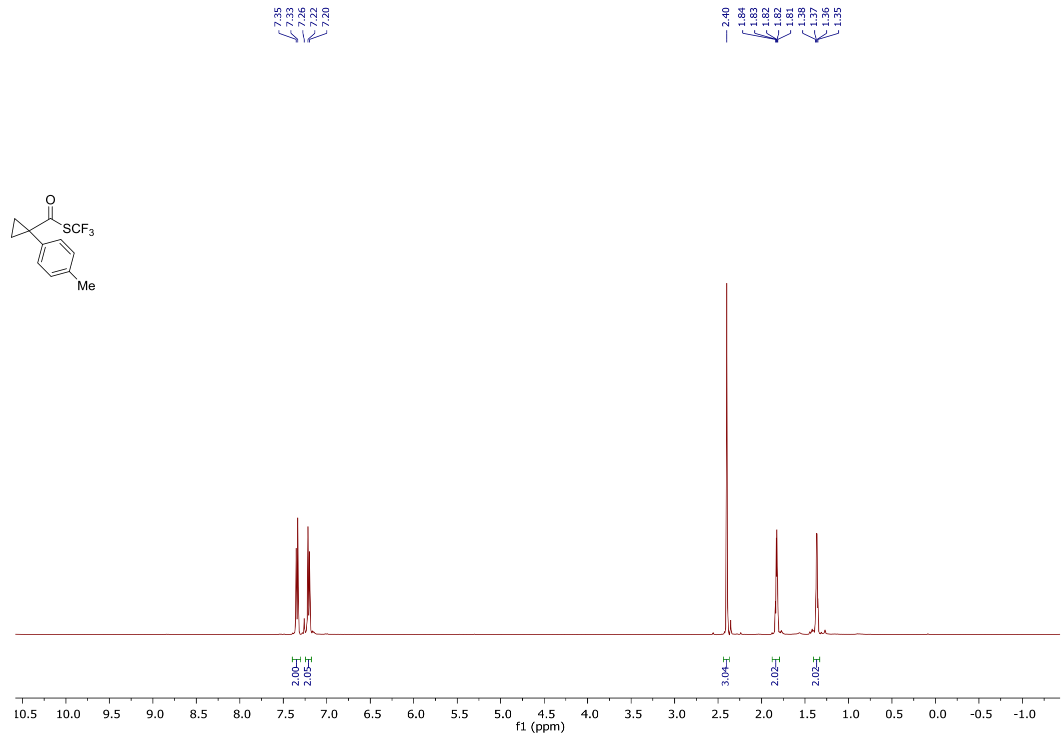

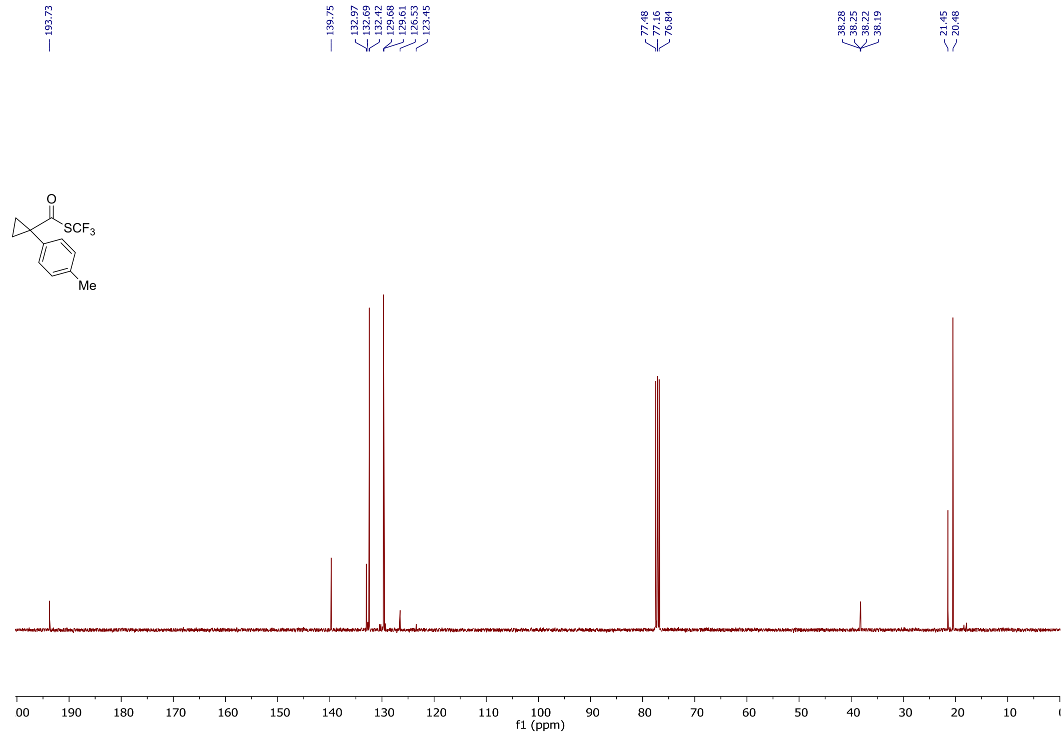

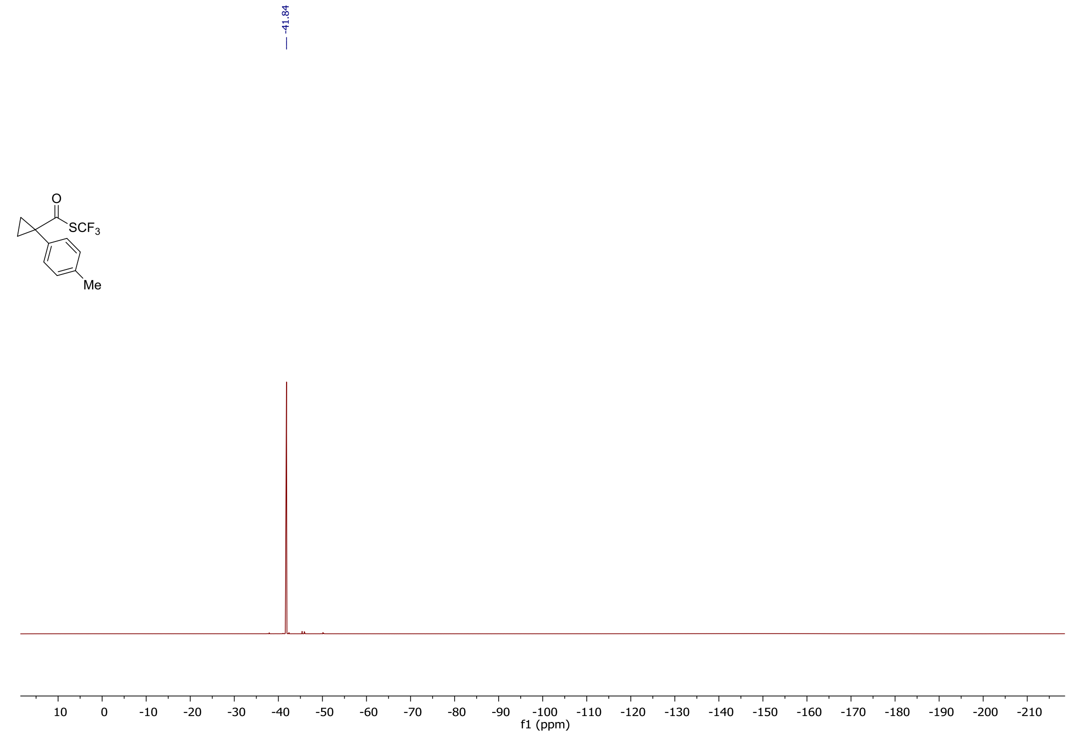

-41.84
O
SCF3
Me
10 0 -10 -20 -30 -40 -50 -60 -70 -80 -90 -100 -110 -120 -130 -140 -150 -160 -170 -180 -190 -200 -210
f1 (ppm)

Figure S28. NMR spectra of 4d

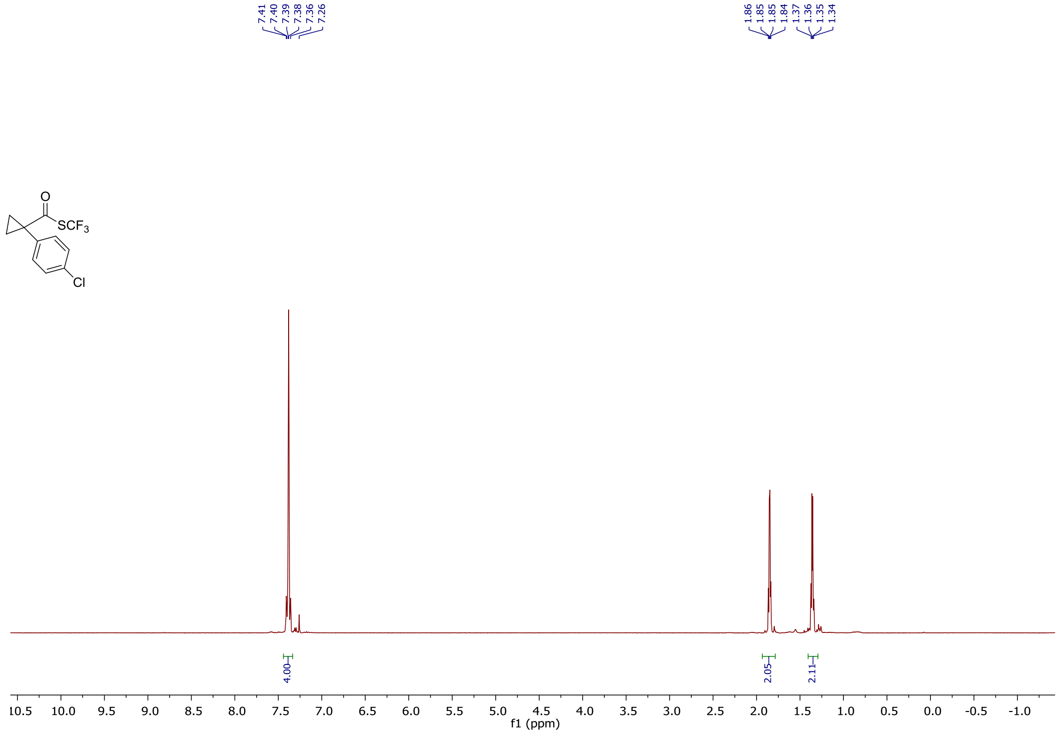

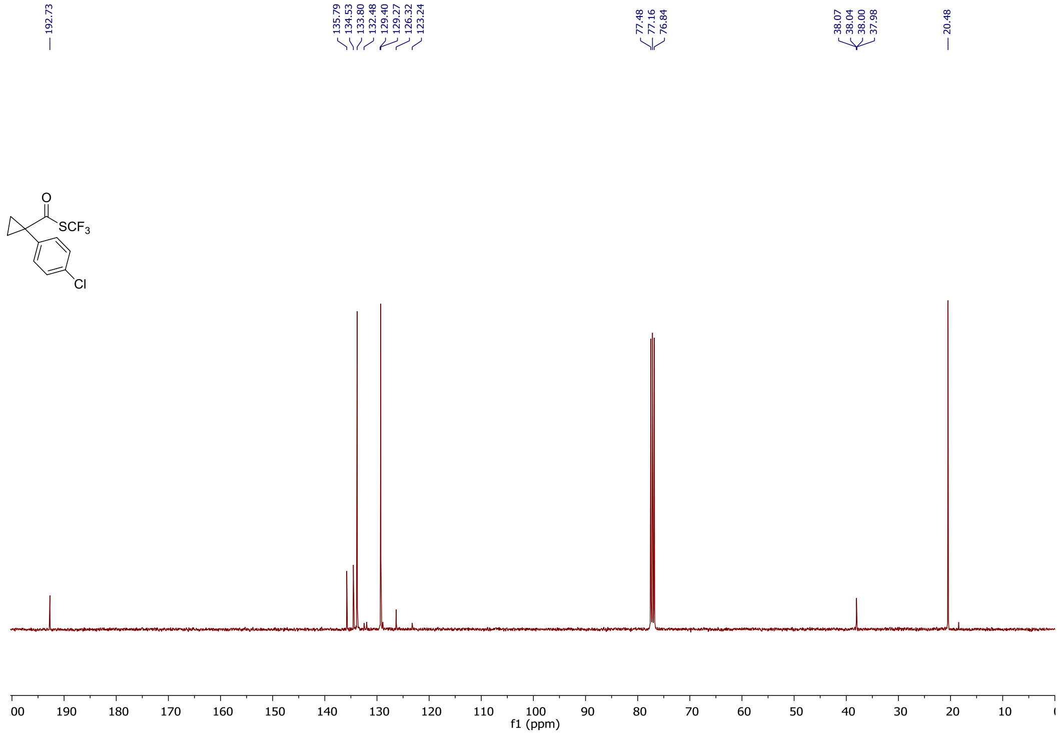

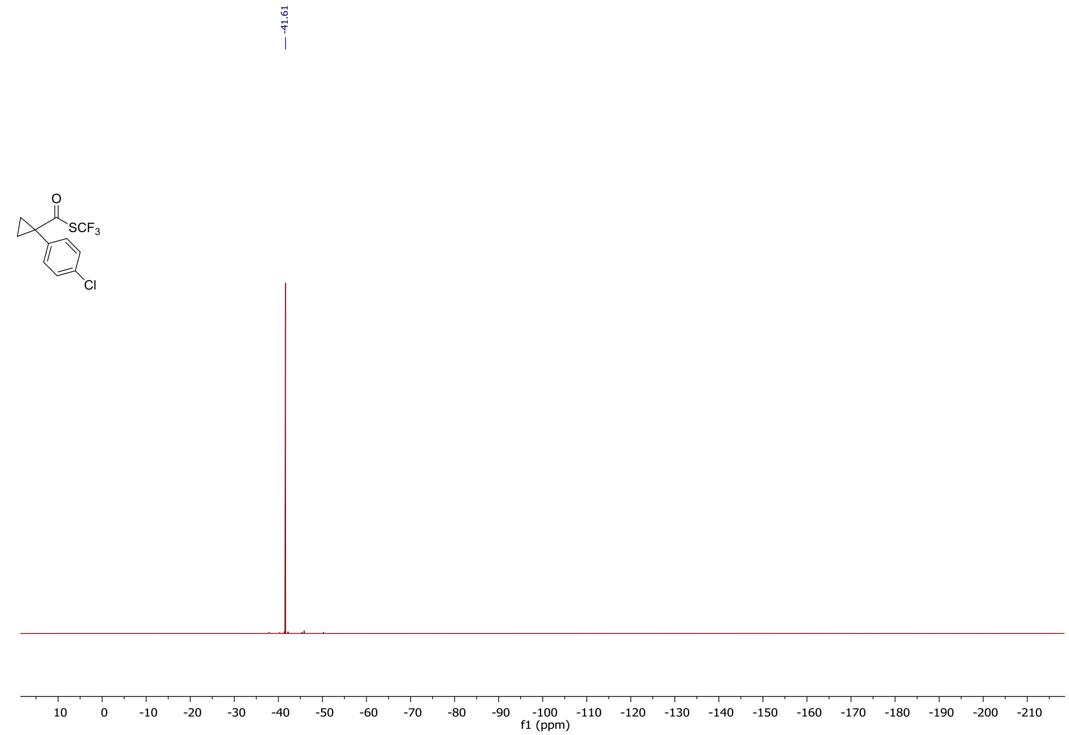
-41.61
O
SCF3
Cl
10 0 -10 -20 -30 -40 -50 -60 -70 -80 -90 -100 -110 -120 -130 -140 -150 -160 -170 -180 -190 -200 -210
f1 (ppm)

Figure S29. NMR spectra of 4e

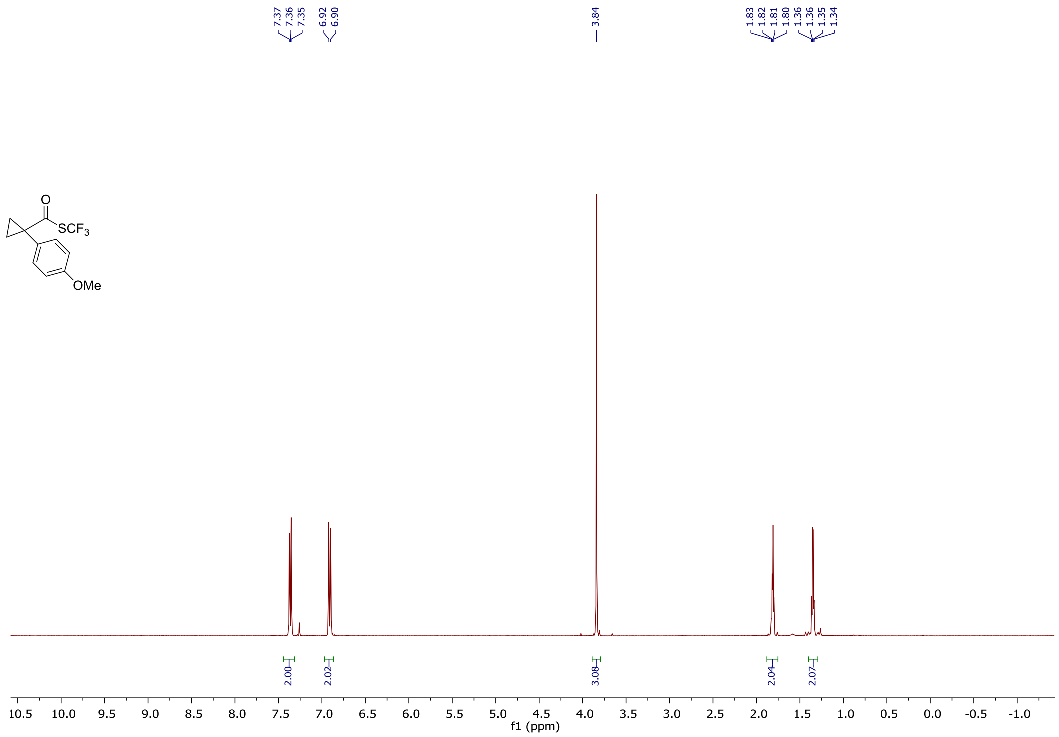

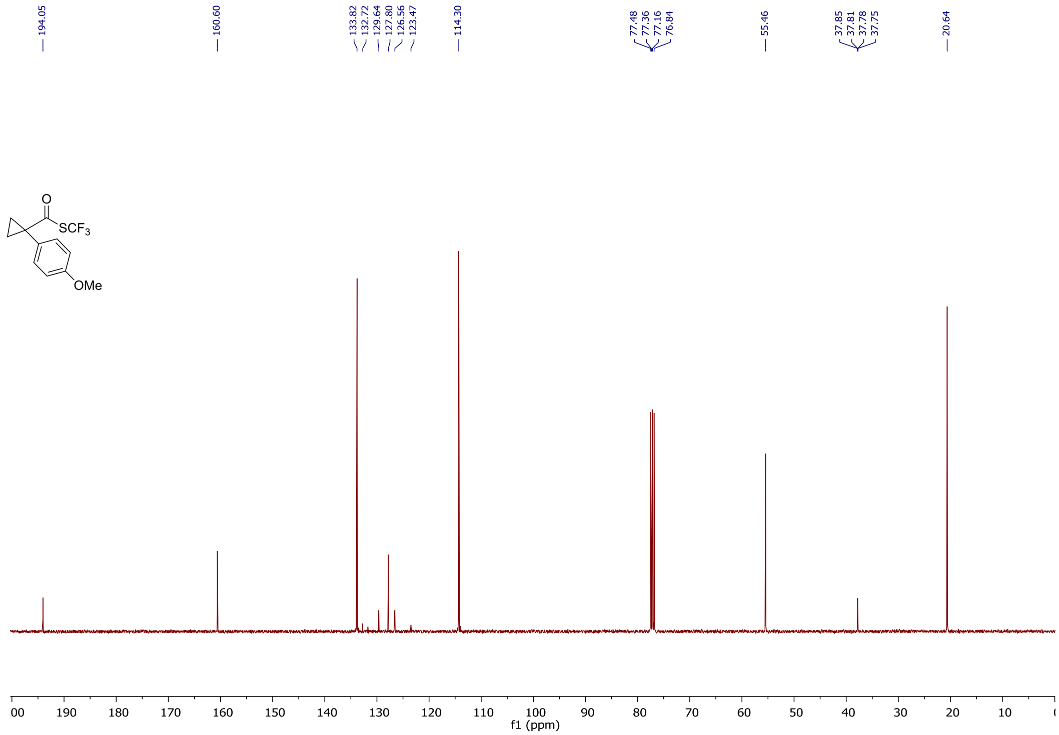

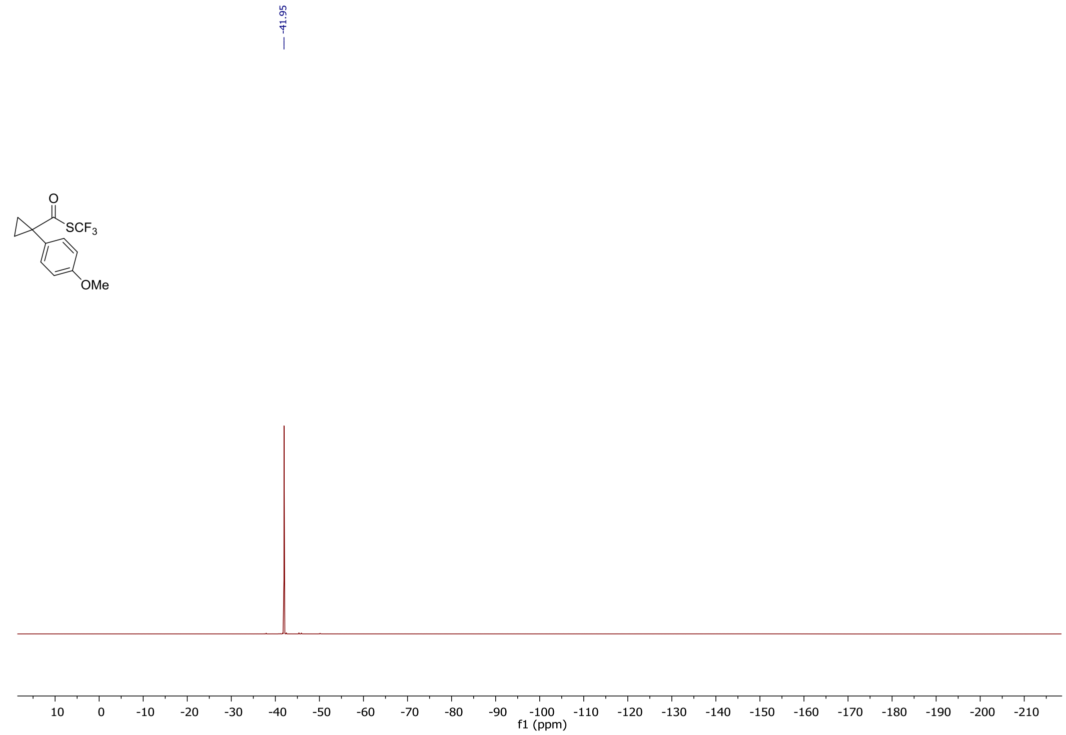
O
SCF3
OMe
-41.95
10
0
-10
-20
-30
-40
-50
-60
-70
-80
-90
-100
-110
-120
-130
-140
-150
-160
-170
-180
-190
-200
-210
f1 (ppm)

Figure S30. NMR spectra of 5a

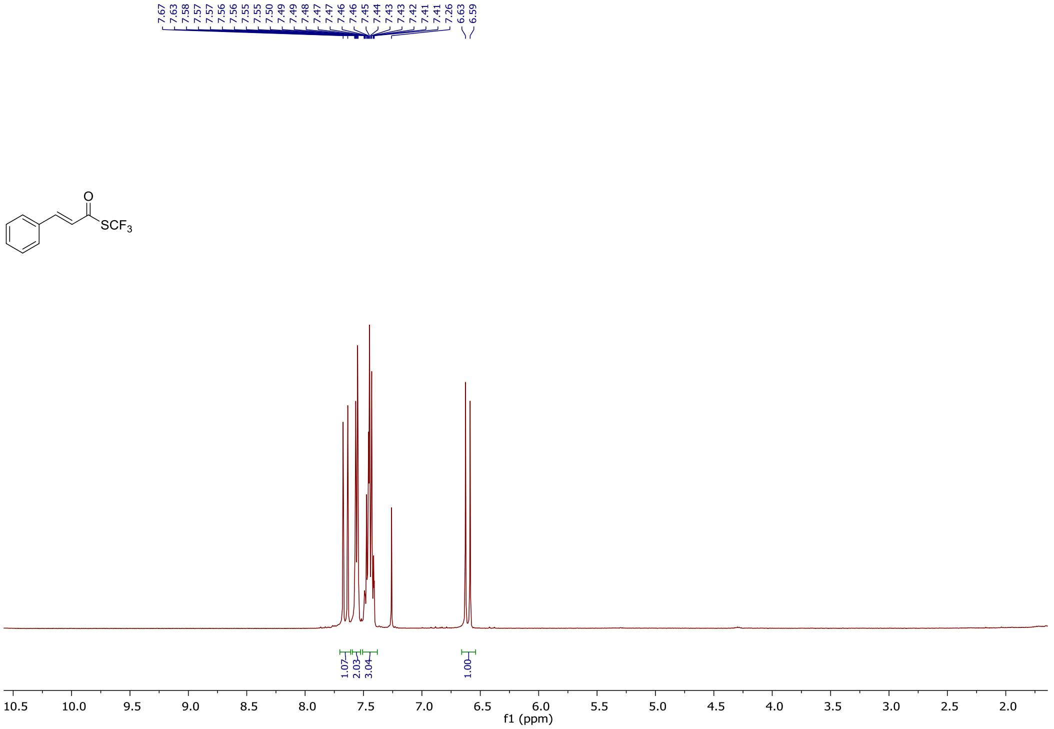

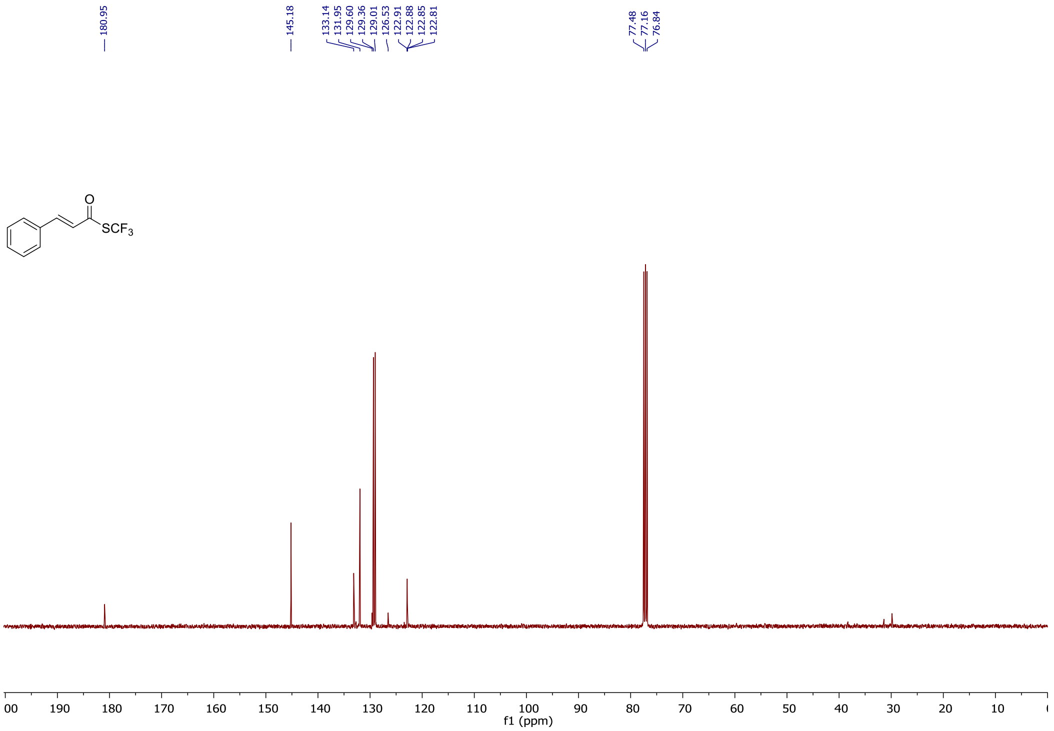

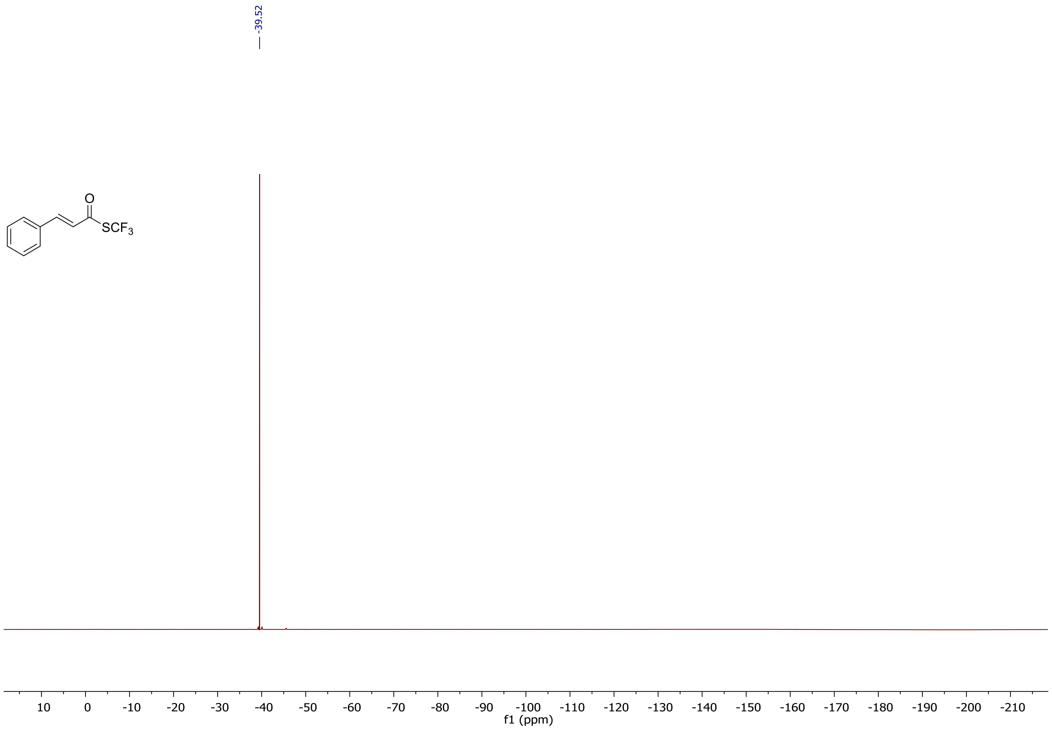

-39.52
O
SCF3
10 0 -10 -20 -30 -40 -50 -60 -70 -80 -90 -100 -110 -120 -130 -140 -150 -160 -170 -180 -190 -200 -210
f1 (ppm)

Figure S31. NMR spectra of 5b

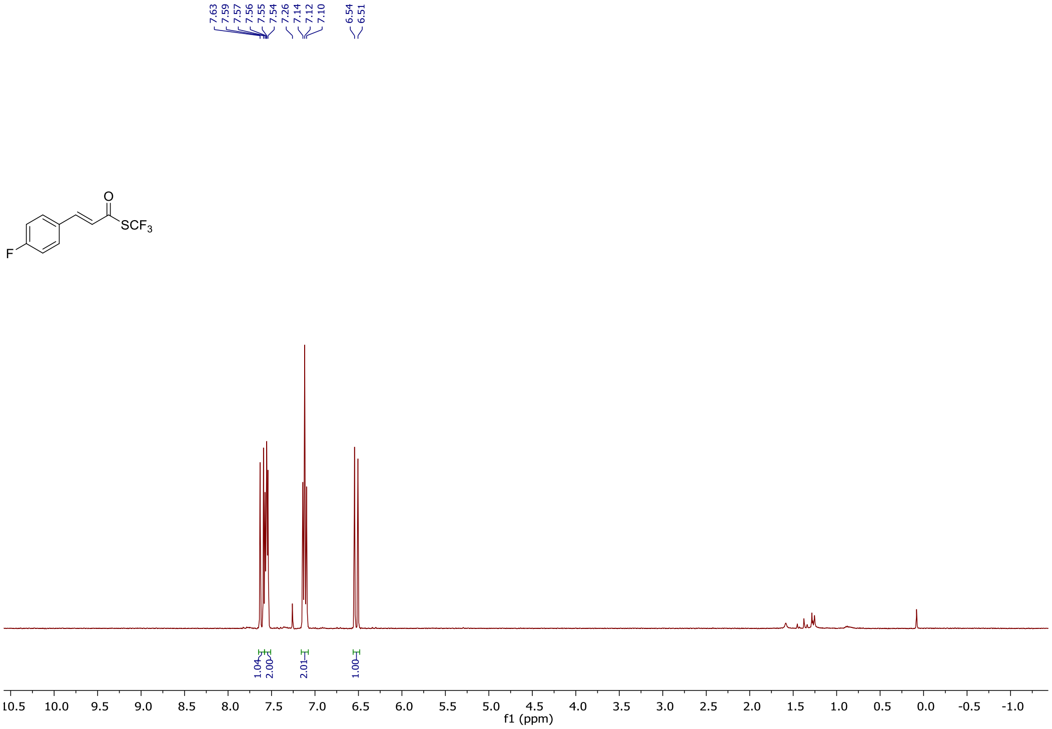

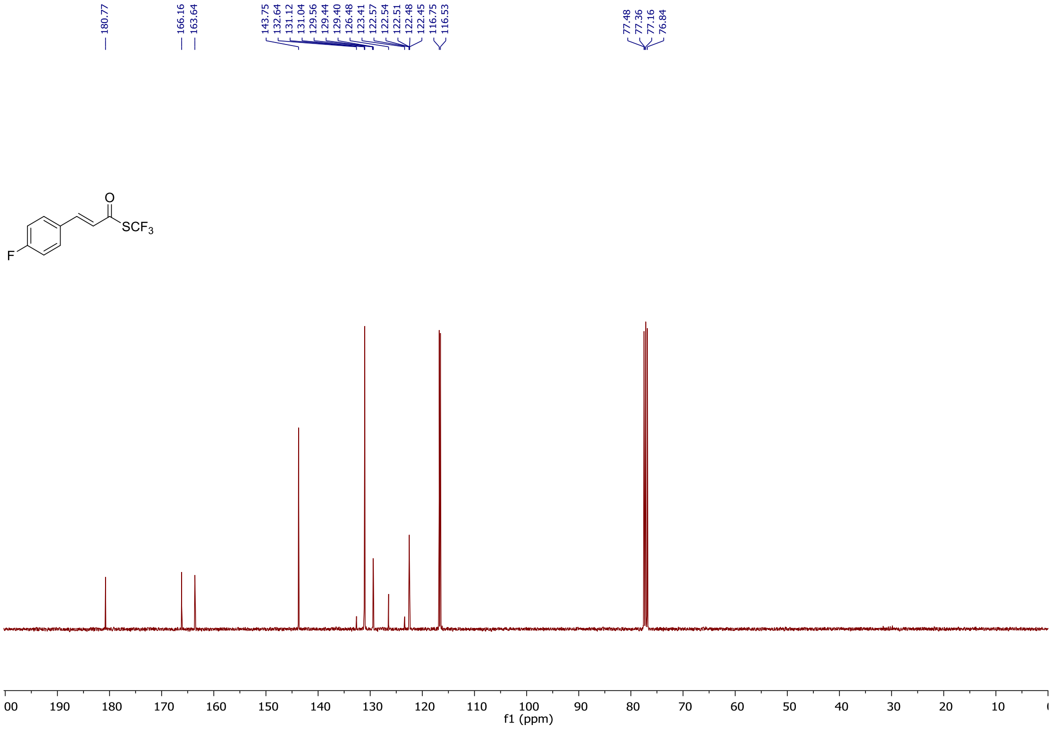

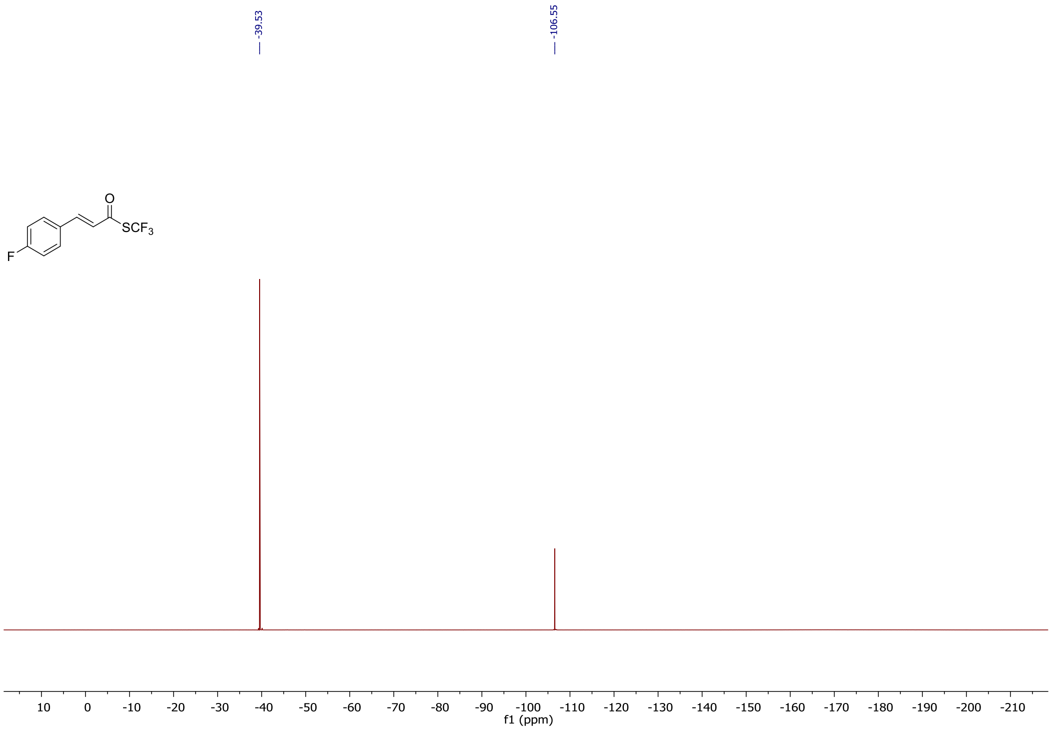

-39.53
-106.55
O
SCF3
F
10 0 -10 -20 -30 -40 -50 -60 -70 -80 -90 -100 -110 -120 -130 -140 -150 -160 -170 -180 -190 -200 -210
f1 (ppm)

Figure S32. NMR spectra of 5c

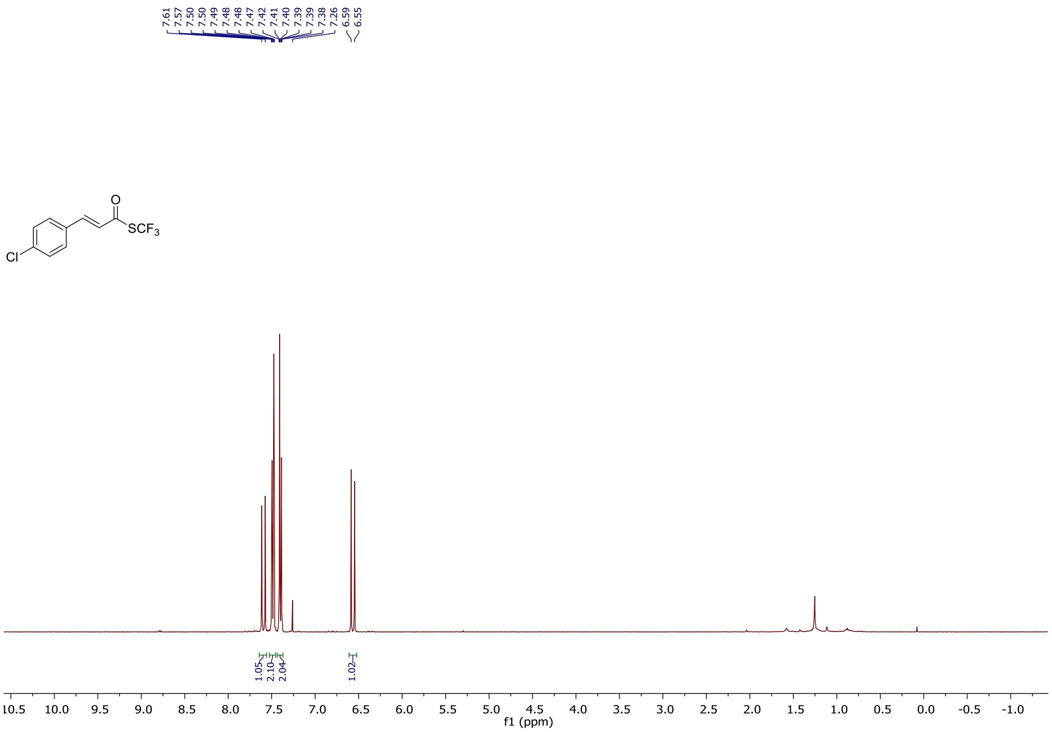

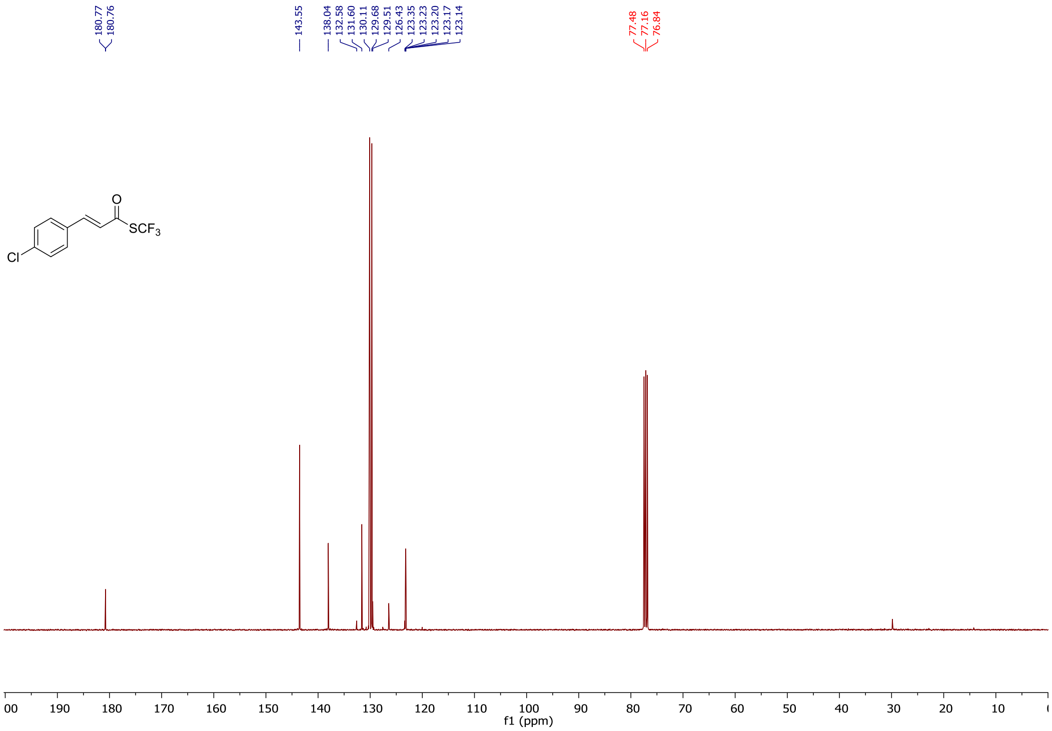

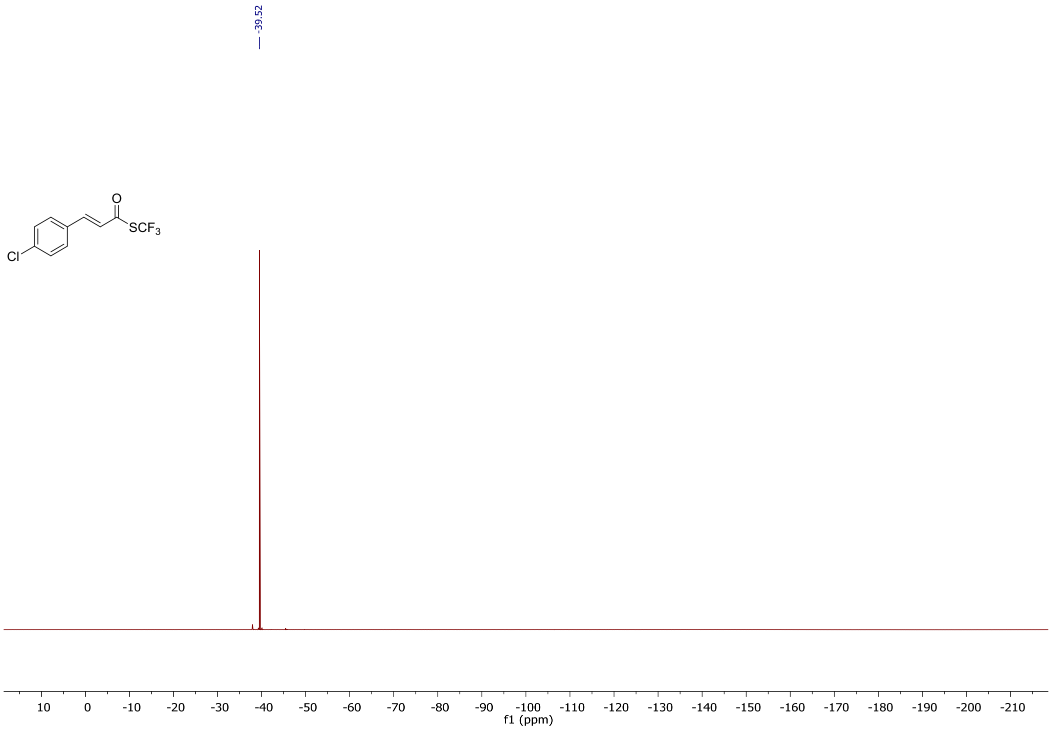

-39.52
SCF3
Cl
10 0 -10 -20 -30 -40 -50 -60 -70 -80 -90 -100 -110 -120 -130 -140 -150 -160 -170 -180 -190 -200 -210
f1 (ppm)

Figure S33. NMR spectra of 5d

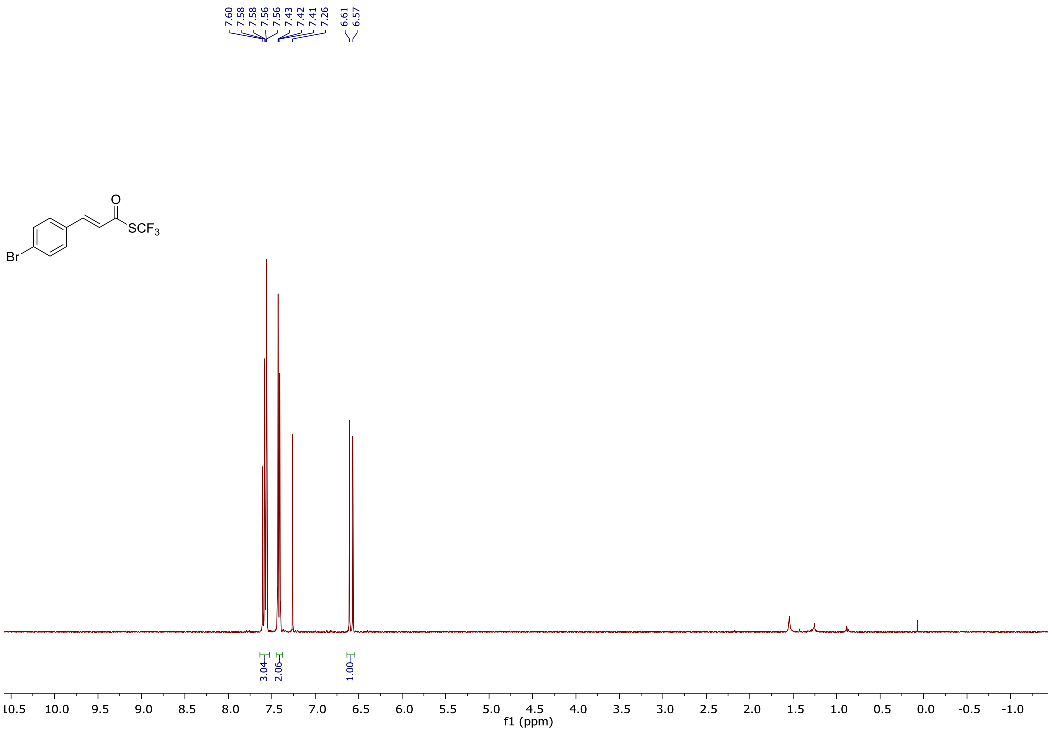

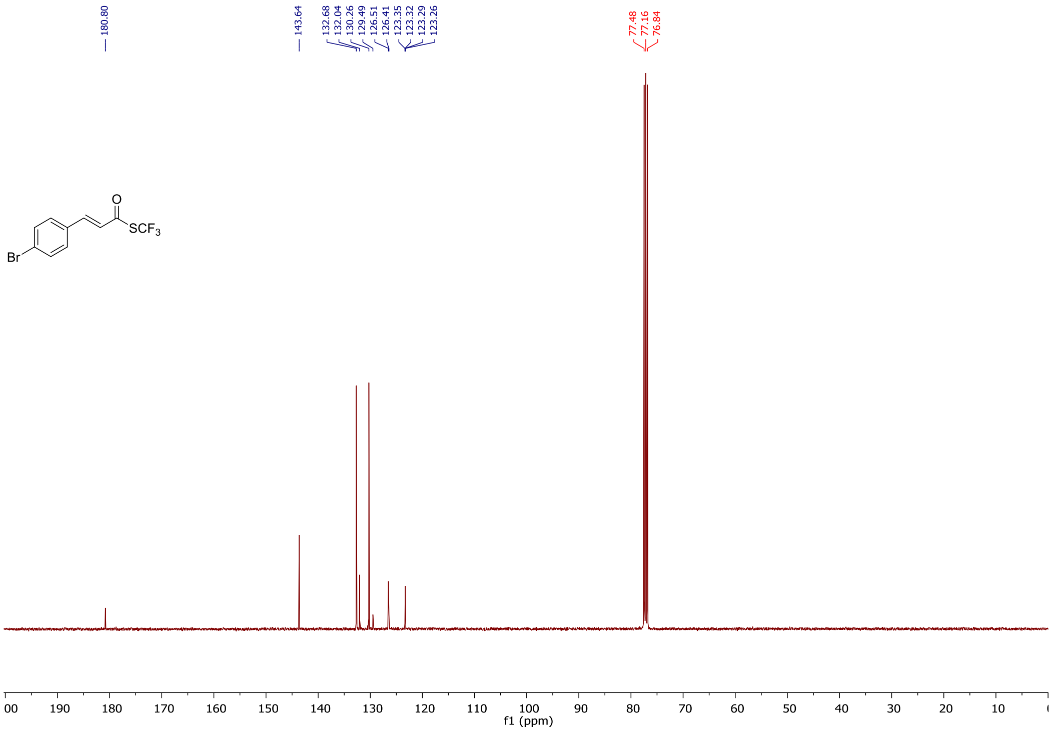

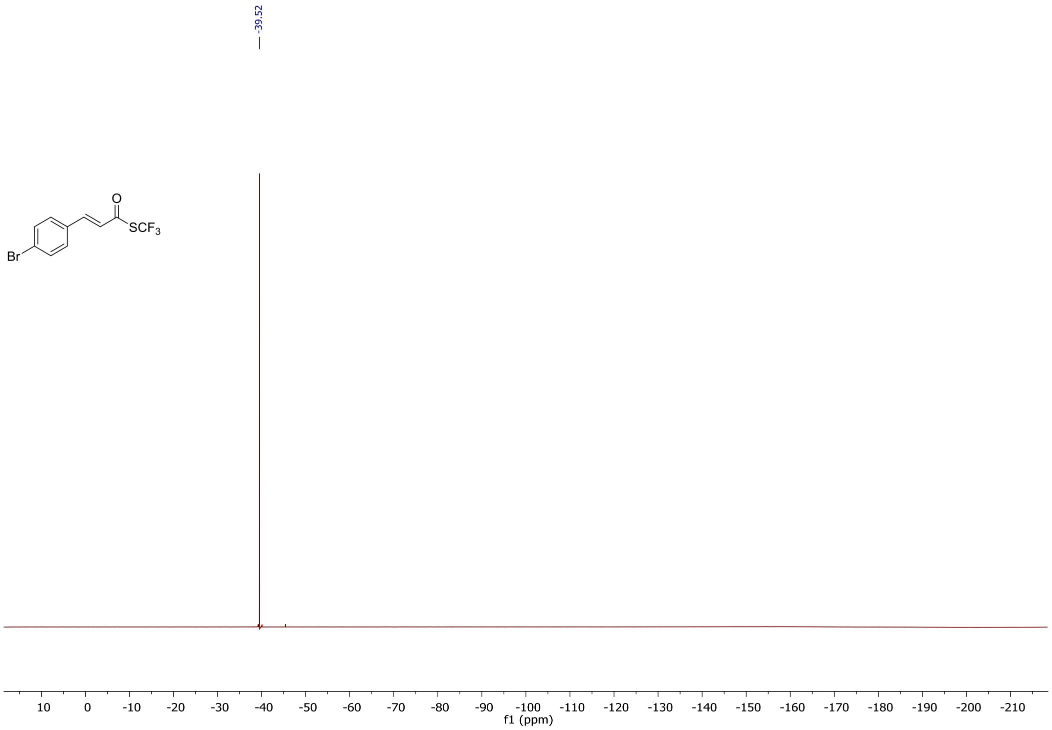
-39.52
Br
O
SCF3
10
0
-10
-20
-30
-40
-50
-60
-70
-80
-90
-100
-110
-120
-130
-140
-150
-160
-170
-180
-190
-200
-210
f1 (ppm)

Figure S34. NMR spectra of 5e

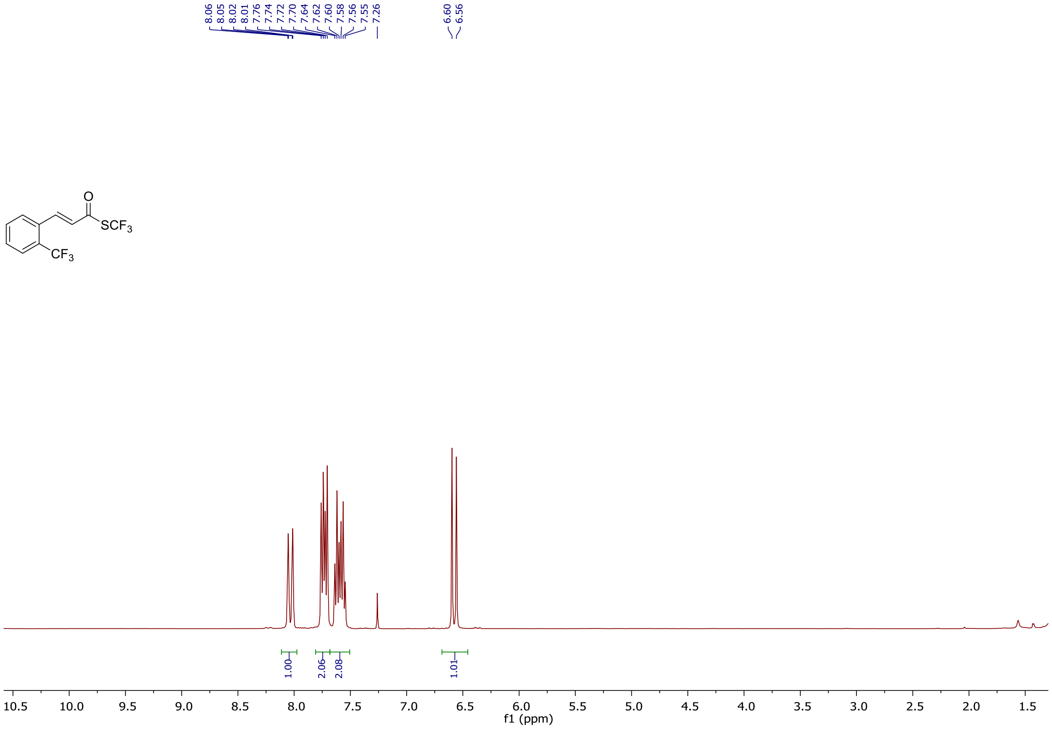

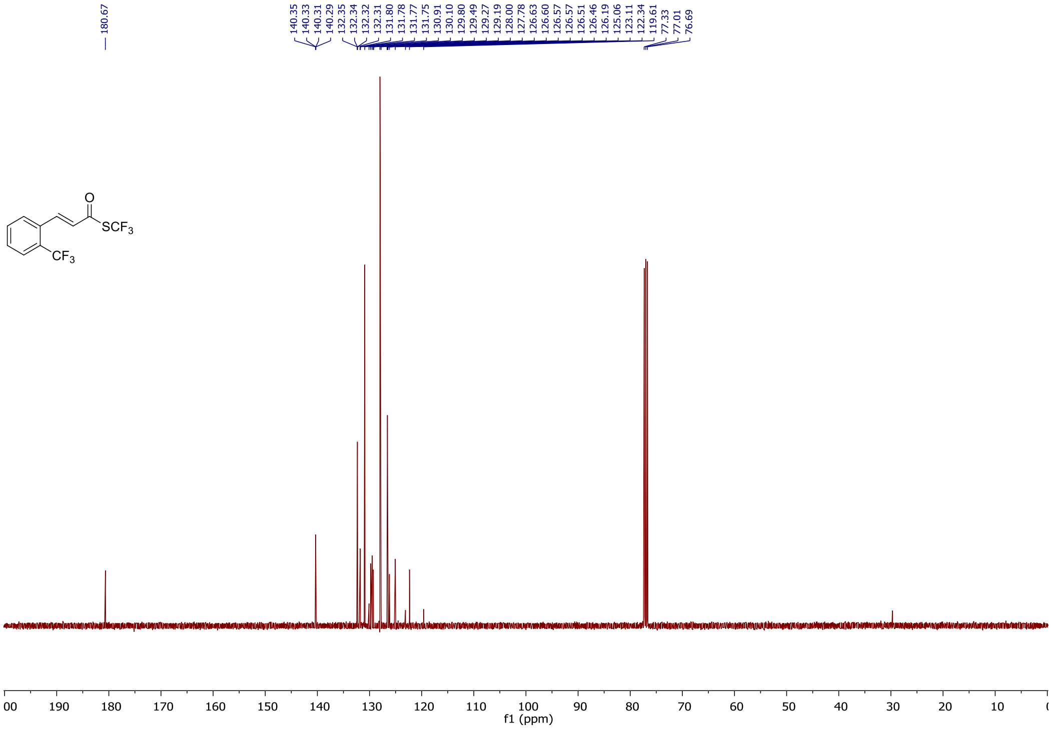

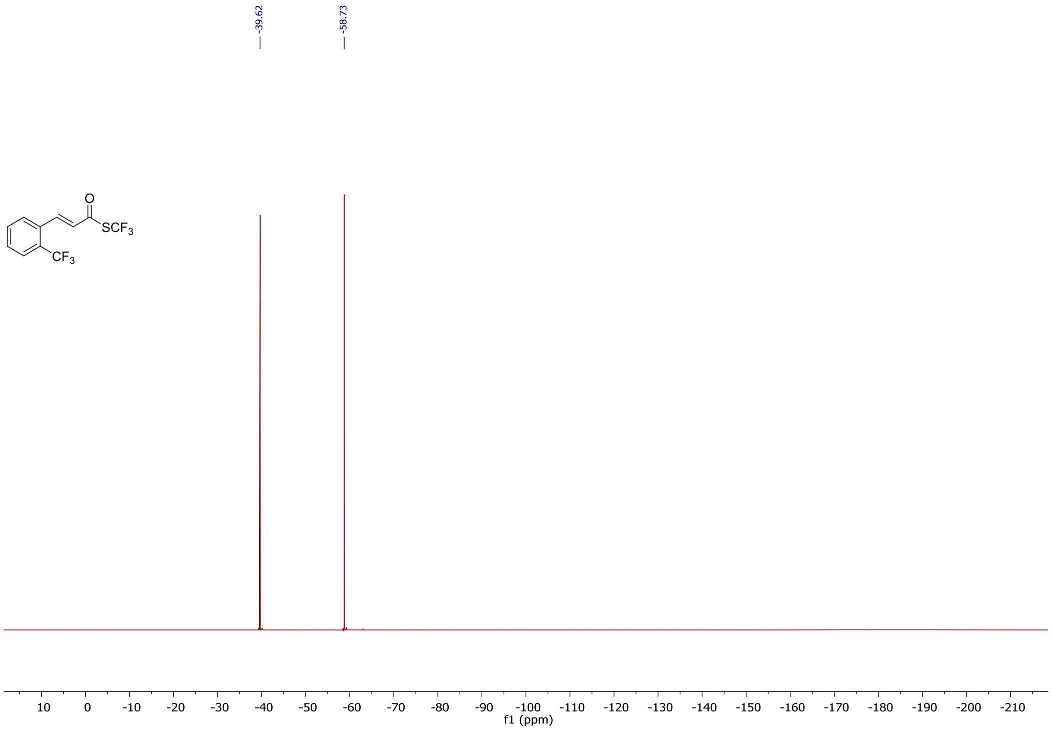
-39.62
-58.73
O
SCF3
CF3
10 0 -10 -20 -30 -40 -50 -60 -70 -80 -90 -100 -110 -120 -130 -140 -150 -160 -170 -180 -190 -200 -210
f1 (ppm)

Figure S35. NMR spectra of 5f

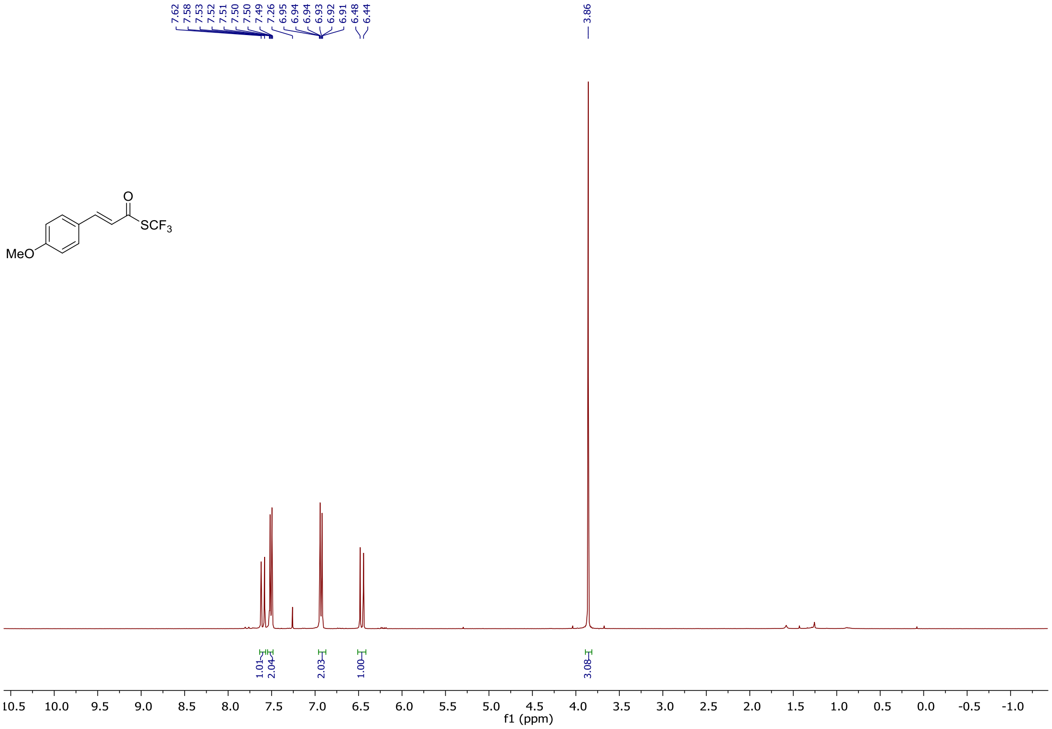

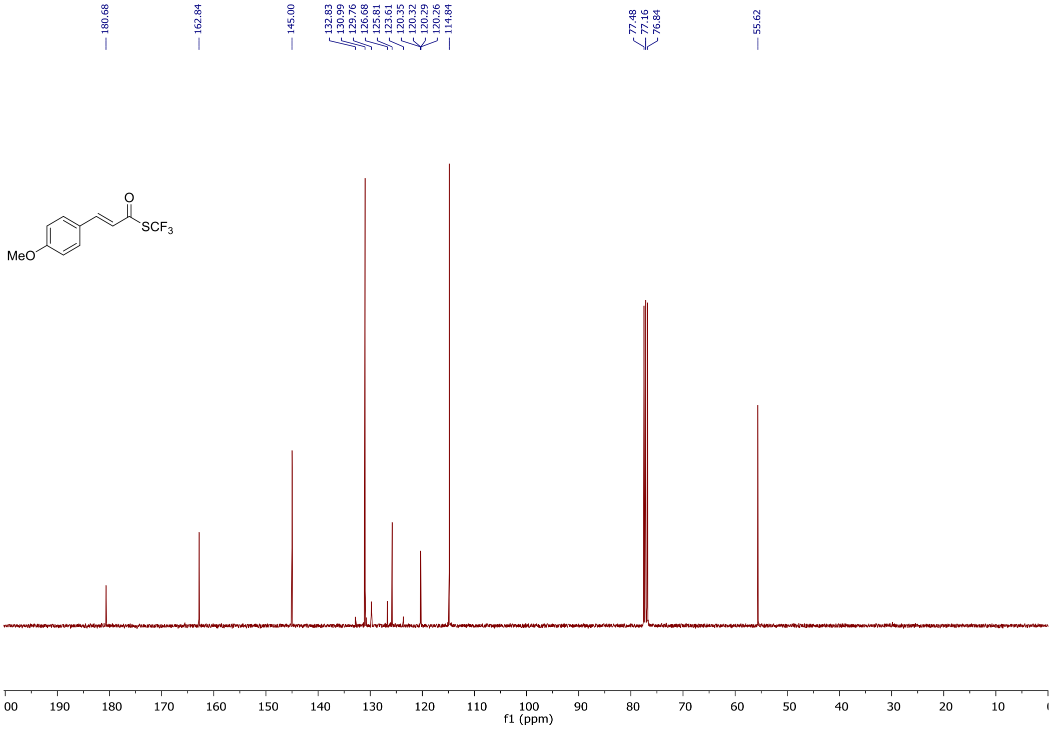

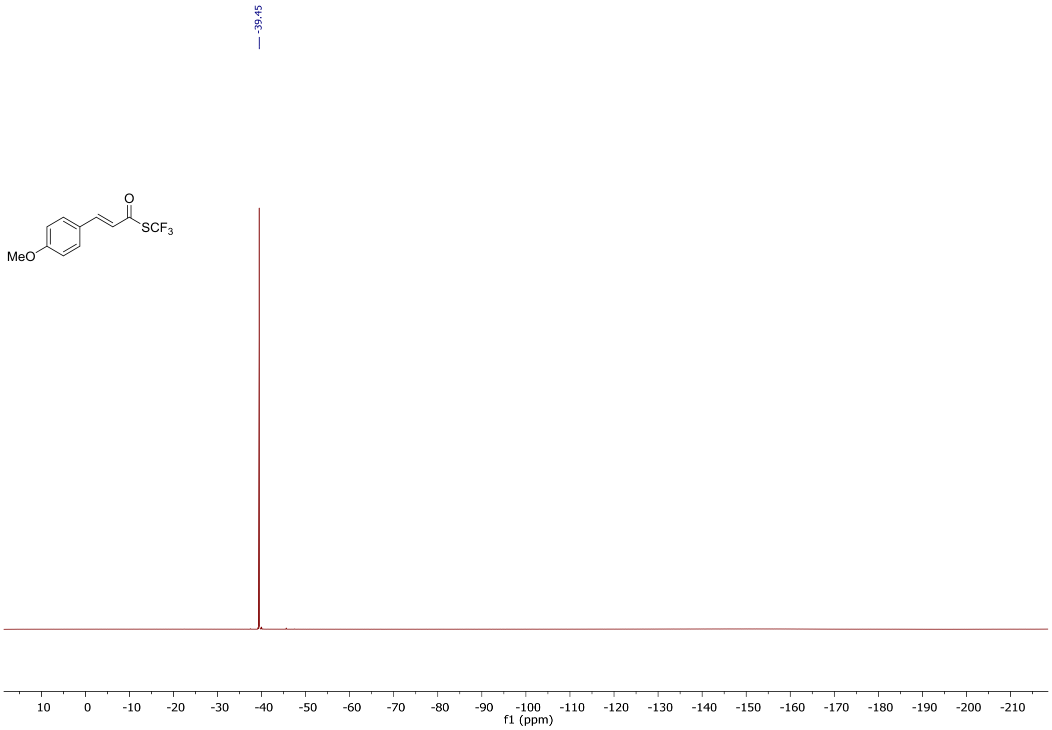

-39.45
MeO
O
SCF3
10 0 -10 -20 -30 -40 -50 -60 -70 -80 -90 -100 -110 -120 -130 -140 -150 -160 -170 -180 -190 -200 -210
f1 (ppm)

Figure S36. NMR spectra of 5g

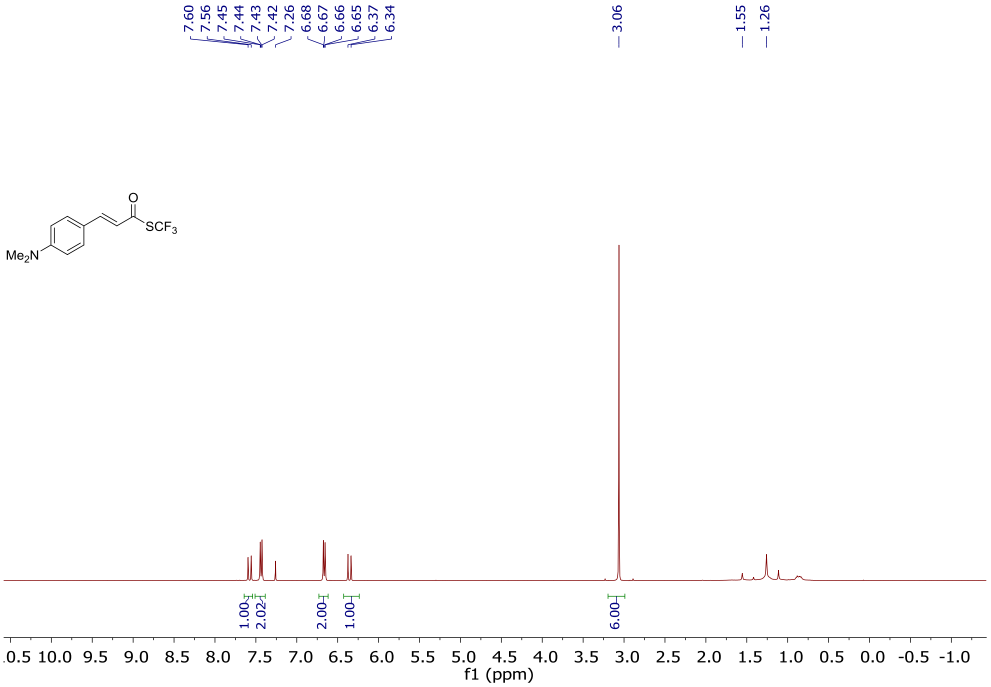

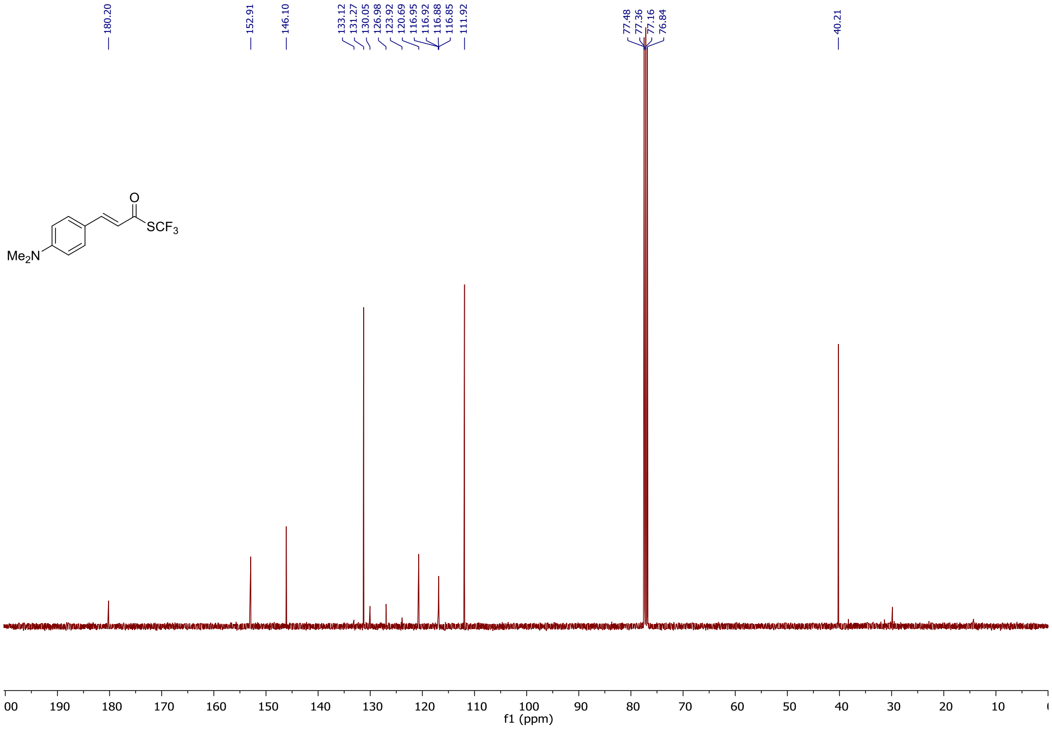

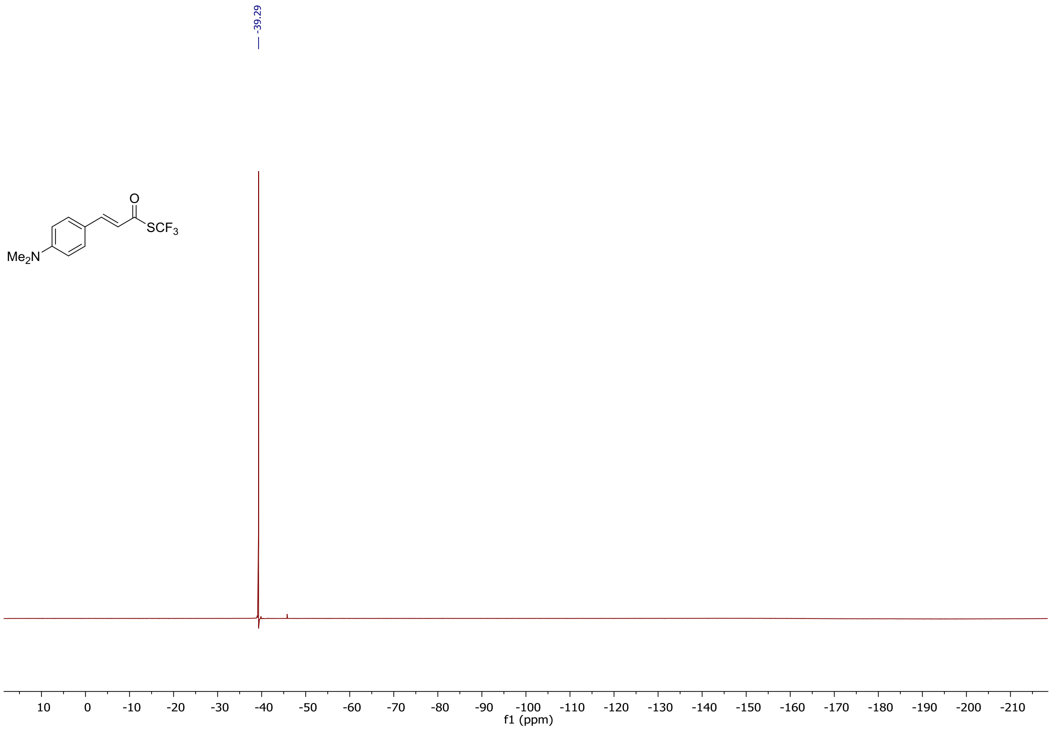
-39.29
Me2N
SCF3
O
f1 (ppm)
10
0
-10
-20
-30
-40
-50
-60
-70
-80
-90
-100
-110
-120
-130
-140
-150
-160
-170
-180
-190
-200
-210

Figure S37. NMR spectra of 5h

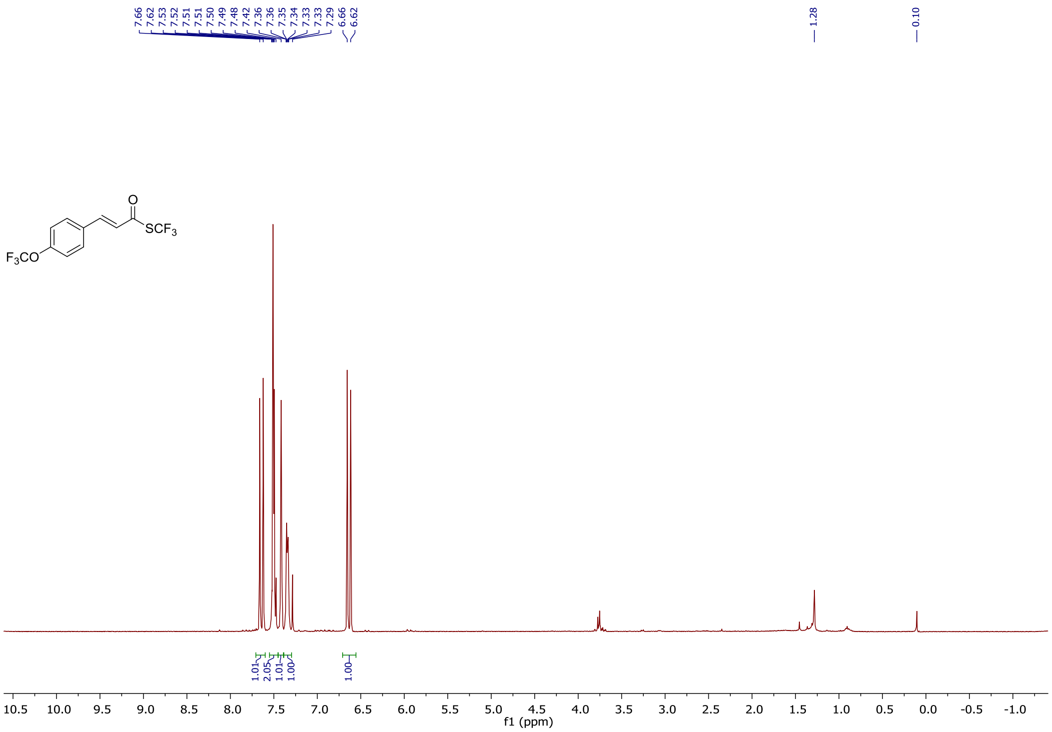

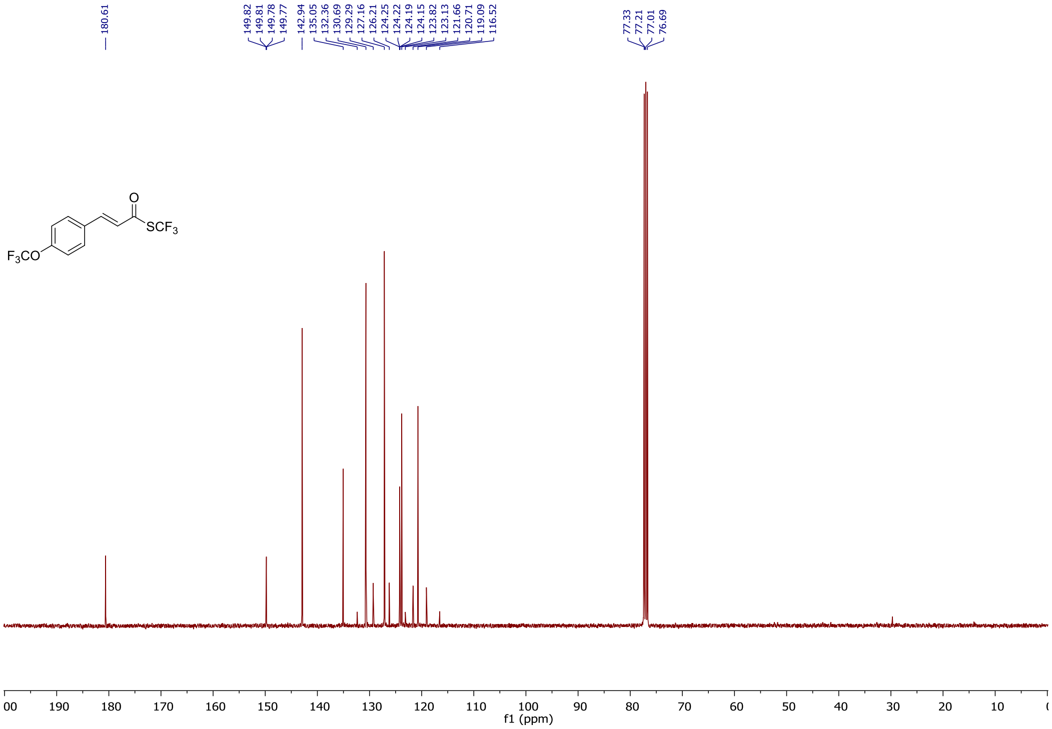

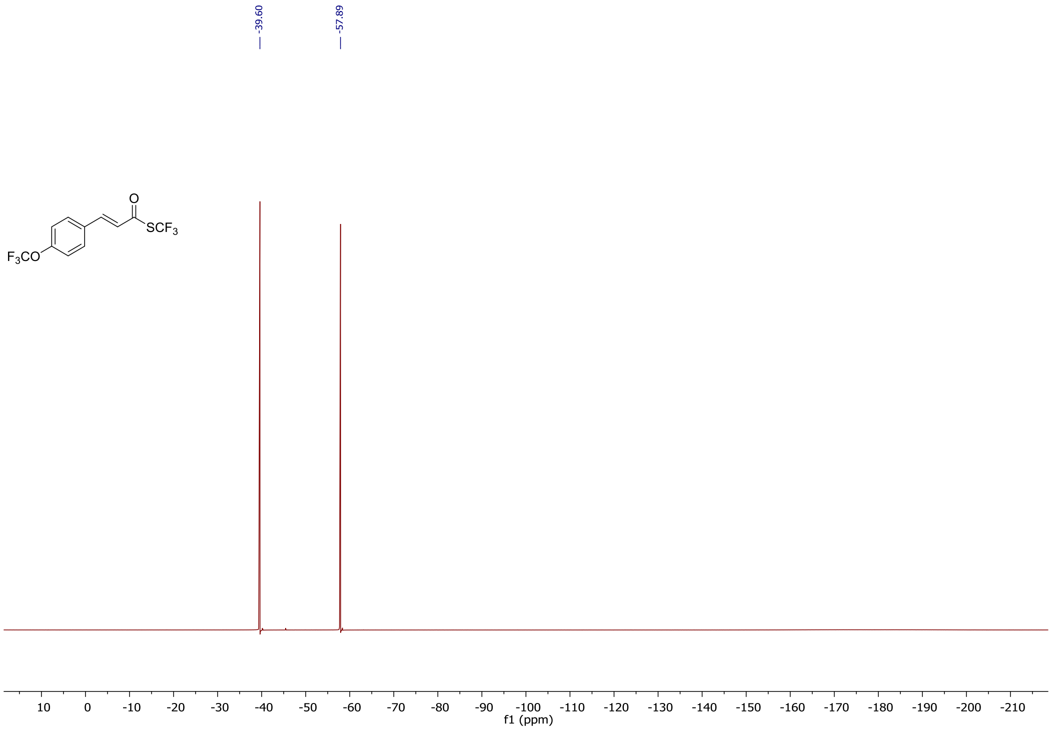

-39.60
-57.89
SCF3
F3CO
10 0 -10 -20 -30 -40 -50 -60 -70 -80 -90 -100 -110 -120 -130 -140 -150 -160 -170 -180 -190 -200 -210
f1 (ppm)

Figure S38. NMR spectra of 6a

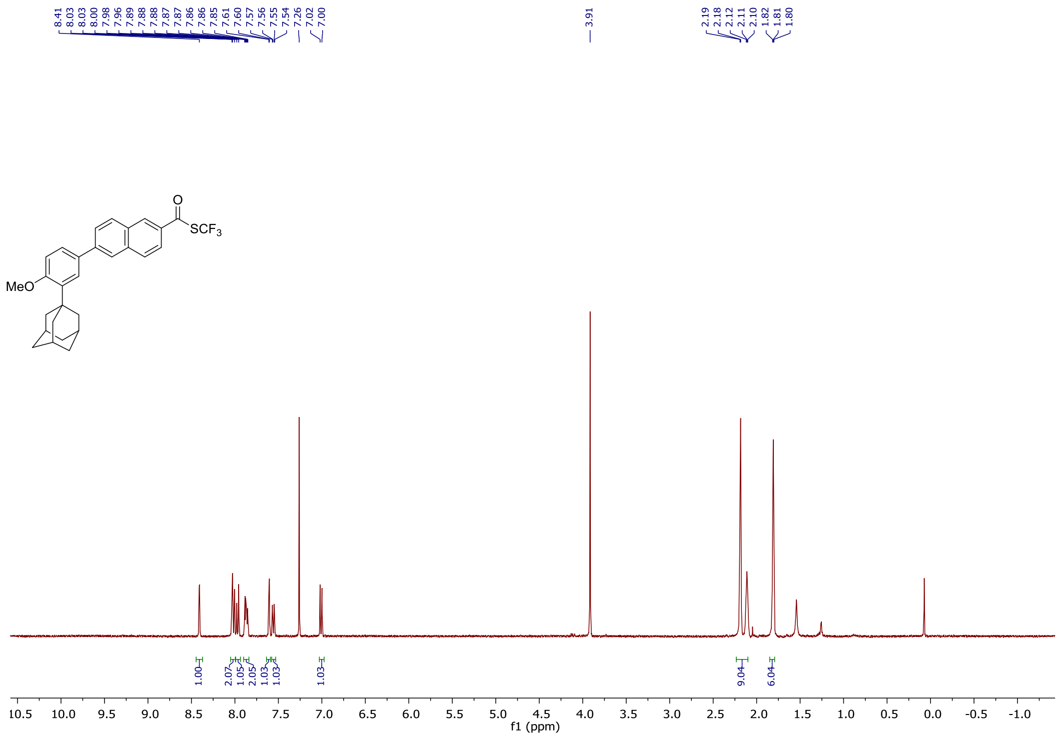

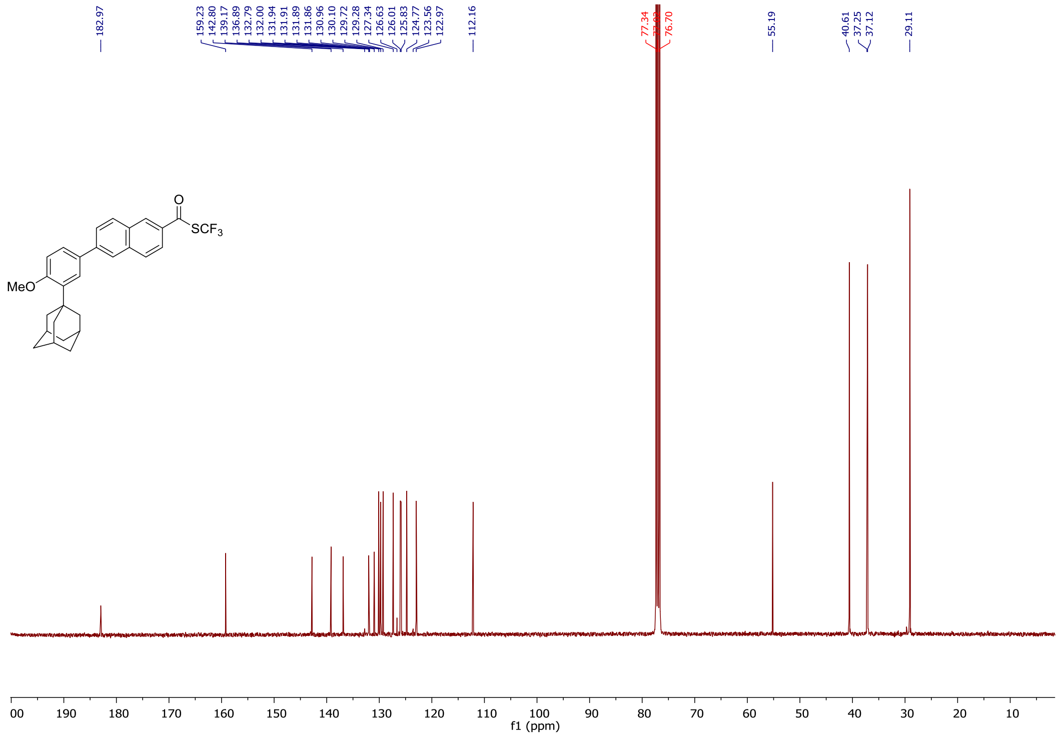

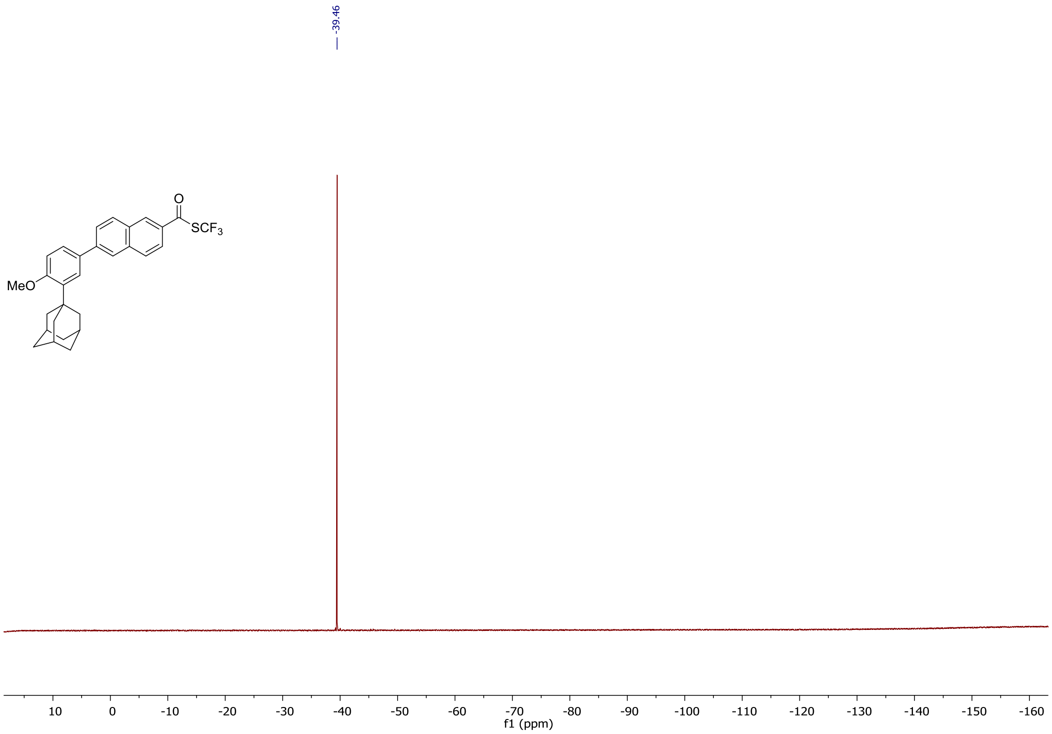
-39.46
O
SCF3
MeO
10 0 -10 -20 -30 -40 -50 -60 -70 -80 -90 -100 -110 -120 -130 -140 -150 -160
f1 (ppm)

Figure S39. NMR spectra of 6b

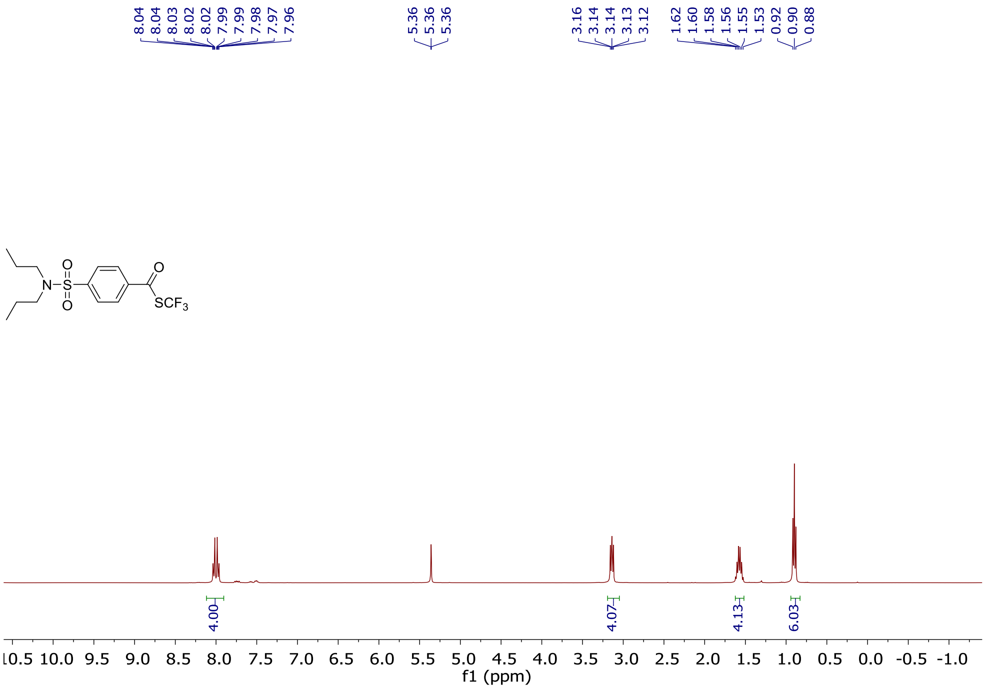

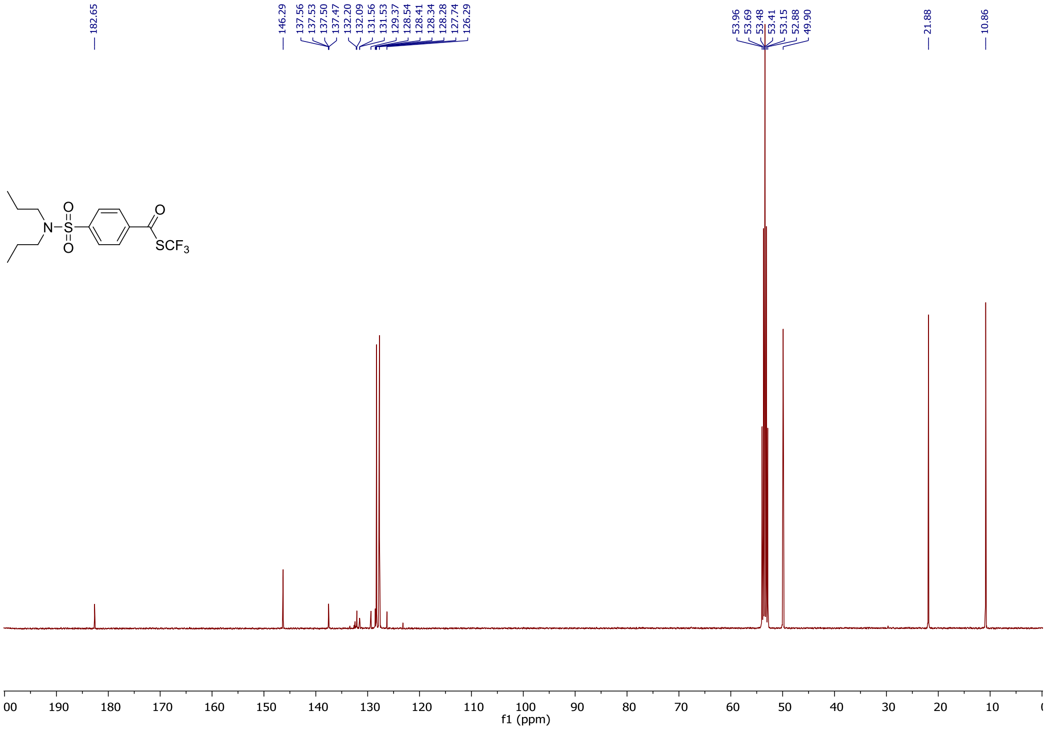

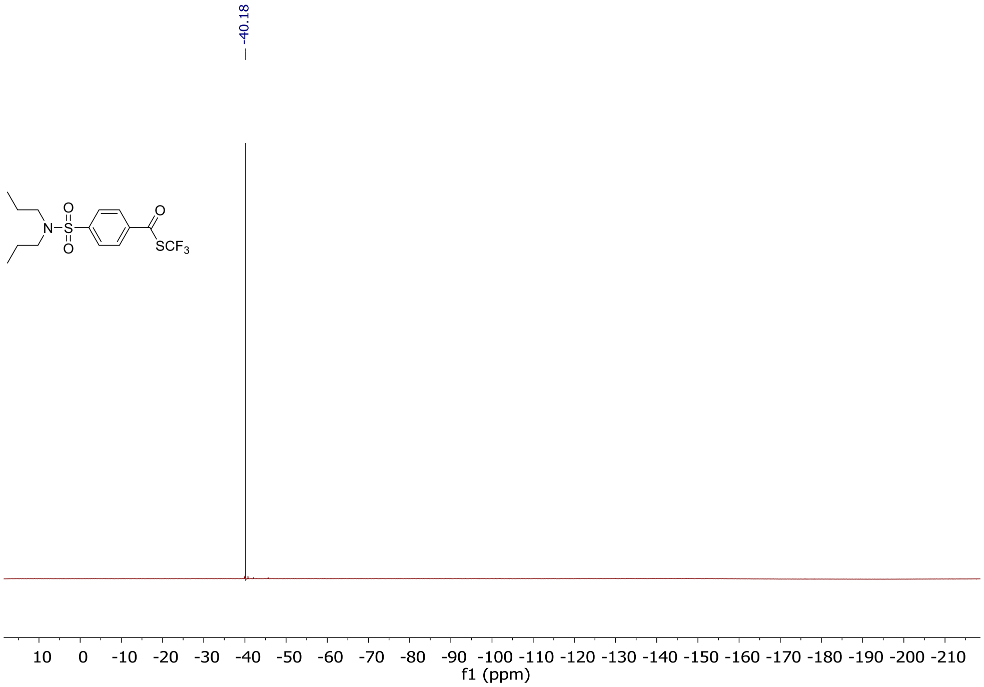

-40.18
O
O
N
S
O
SCF3
10
0
-10
-20
-30
-40
-50
-60
-70
-80
-90
-100
-110
-120
-130
-140
-150
-160
-170
-180
-190
-200
-210
f1 (ppm)

Figure S40. NMR spectra of 6c

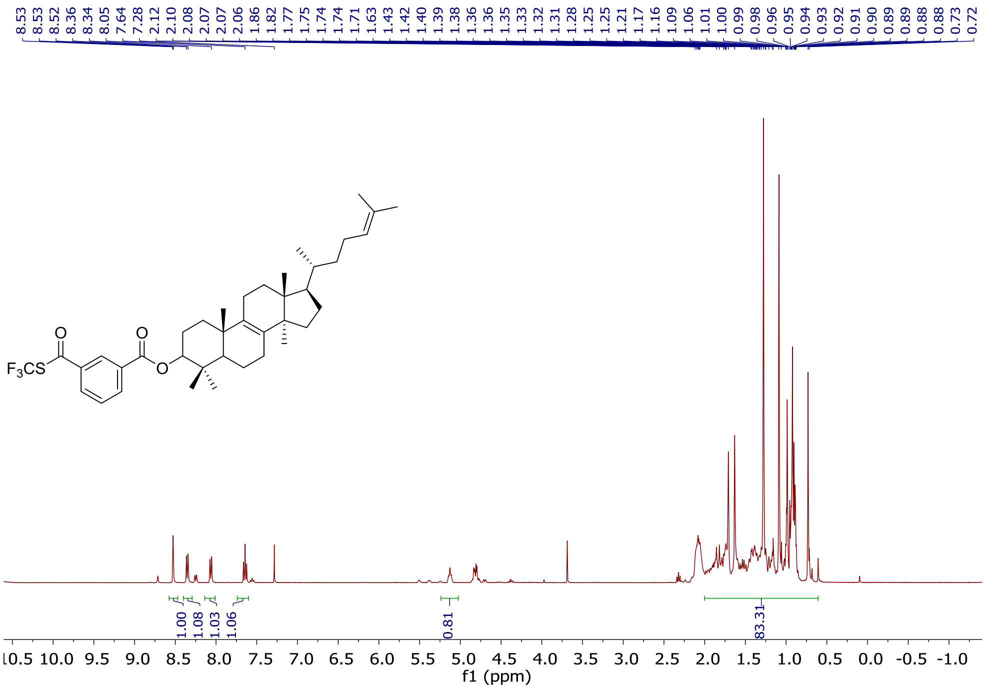

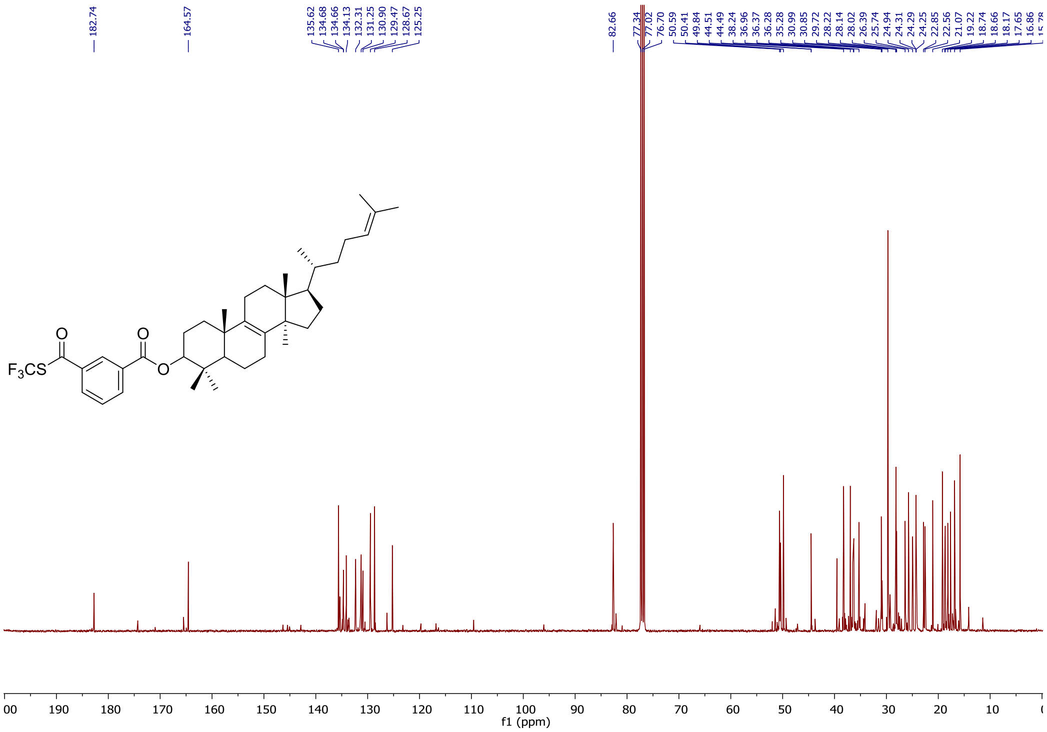

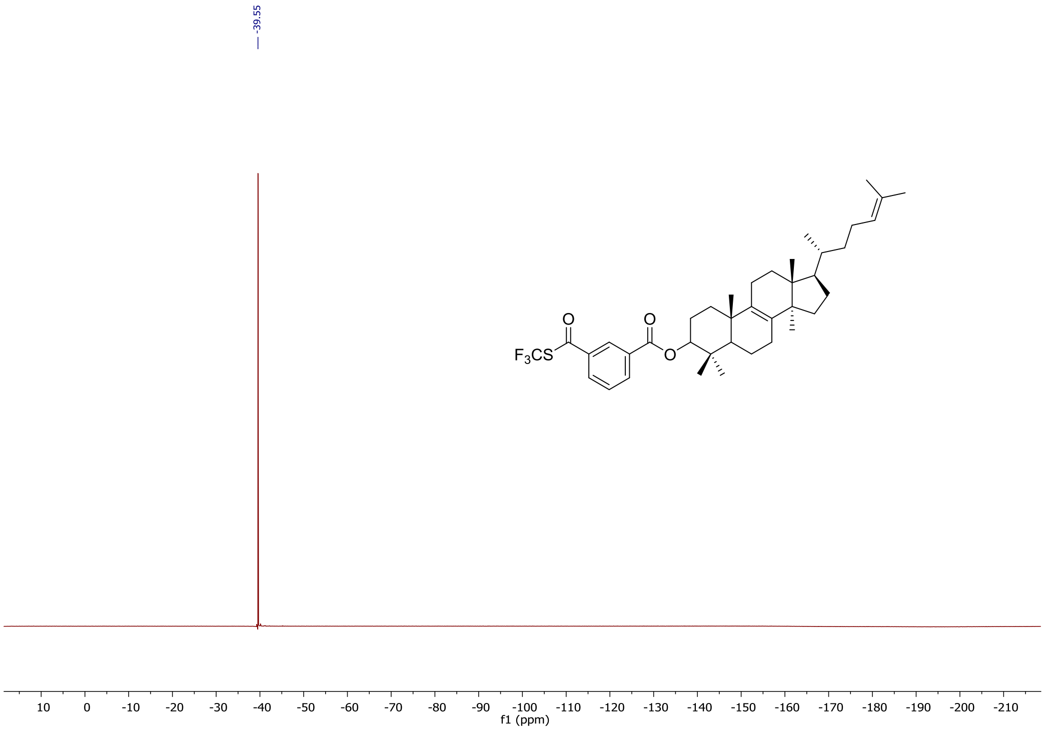
-39.55
F3CS
O
10
0
-10
-20
-30
-40
-50
-60
-70
-80
-90
-100
-110
-120
-130
-140
-150
-160
-170
-180
-190
-200
-210
f1 (ppm)

Figure S41. NMR spectra of 6d

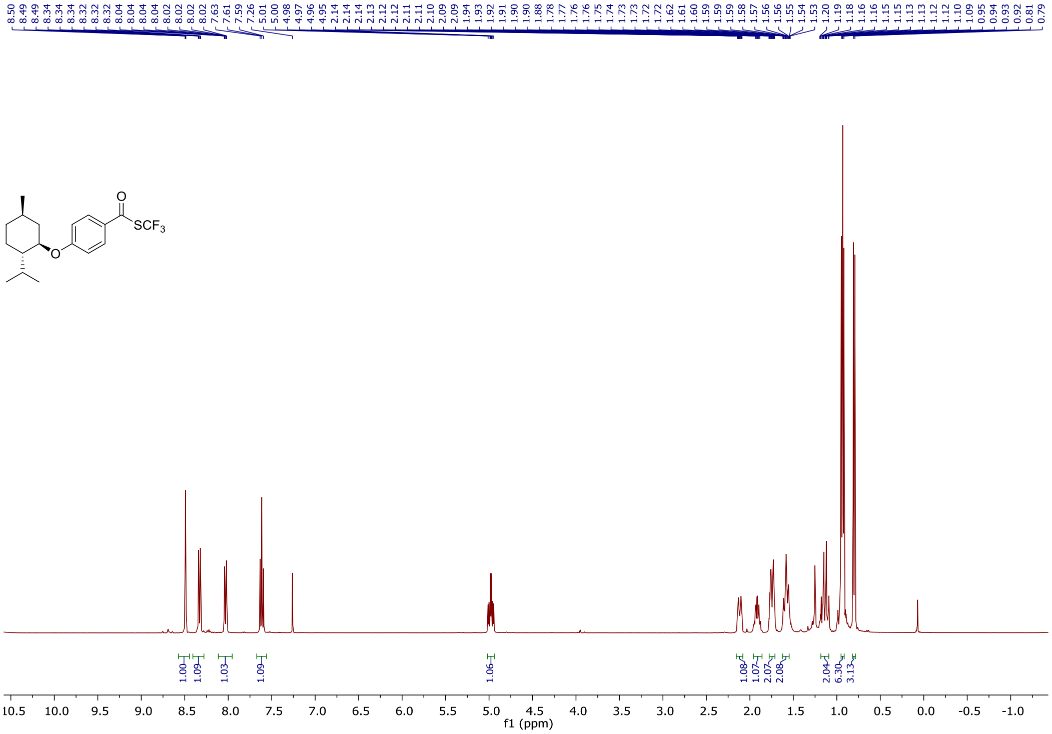

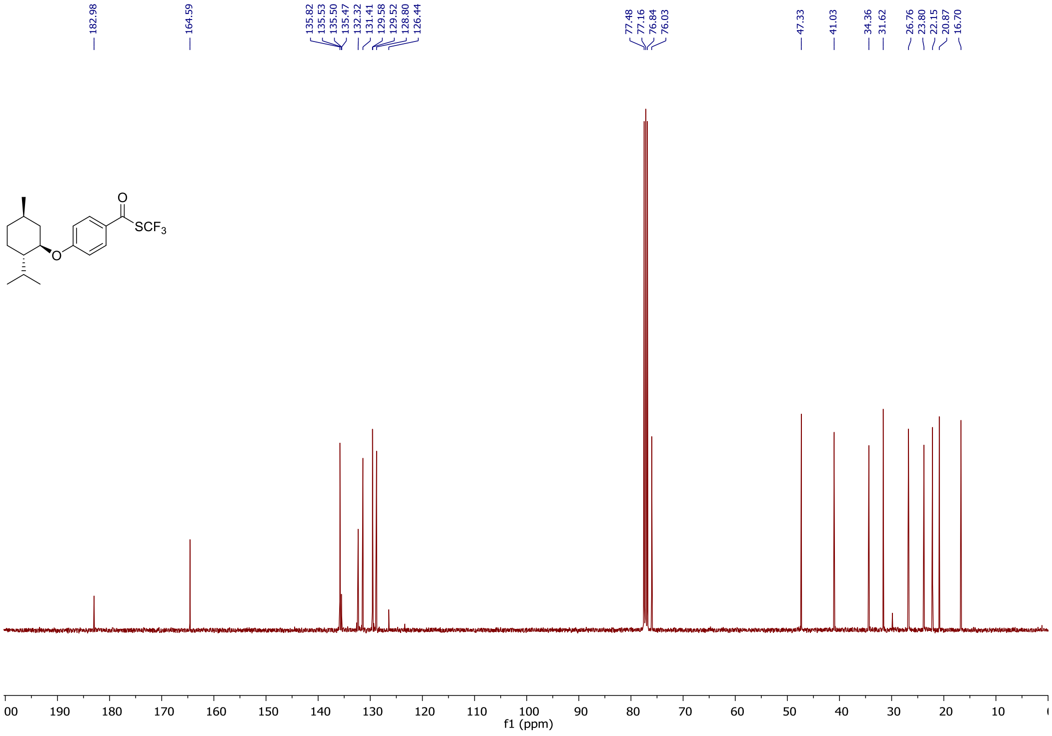

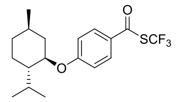
O
SCF3
O

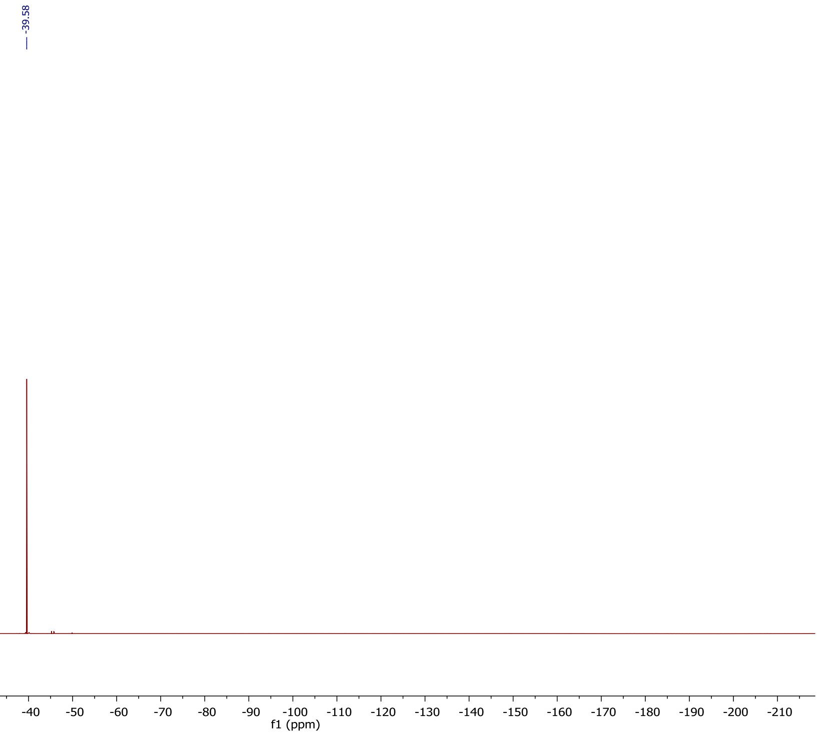
-39.58
10
0
-10
-20
-30
-40
-50
-60
-70
-80
-90
-100
-110
-120
-130
-140
-150
-160
-170
-180
-190
-200
-210
f1 (ppm)

Figure S42. NMR spectra of 6e

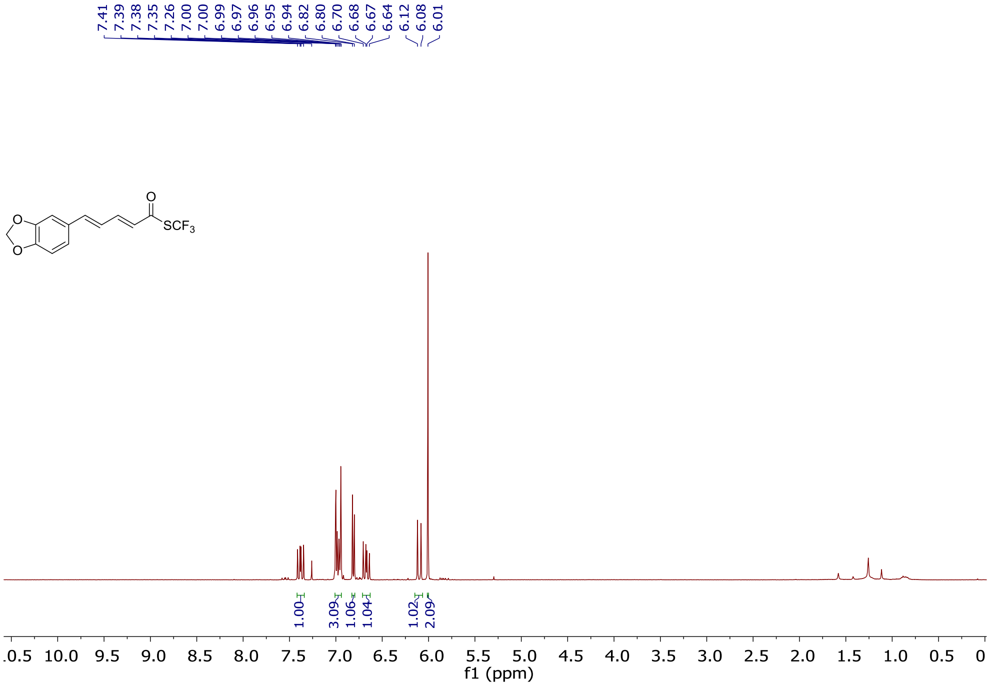

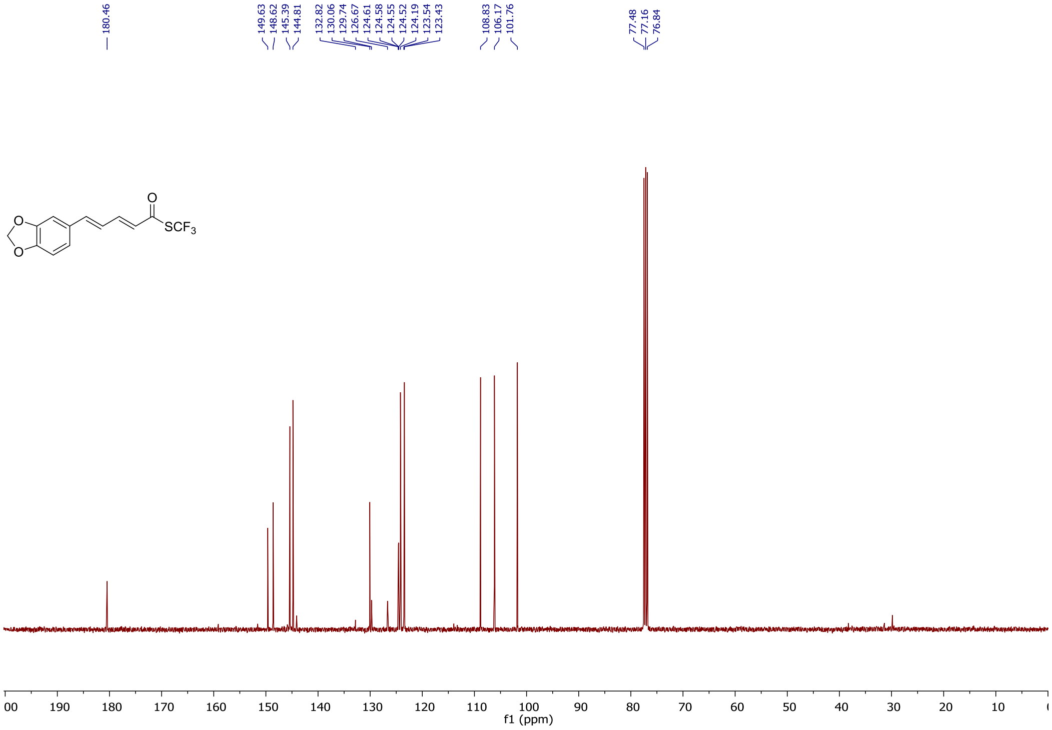

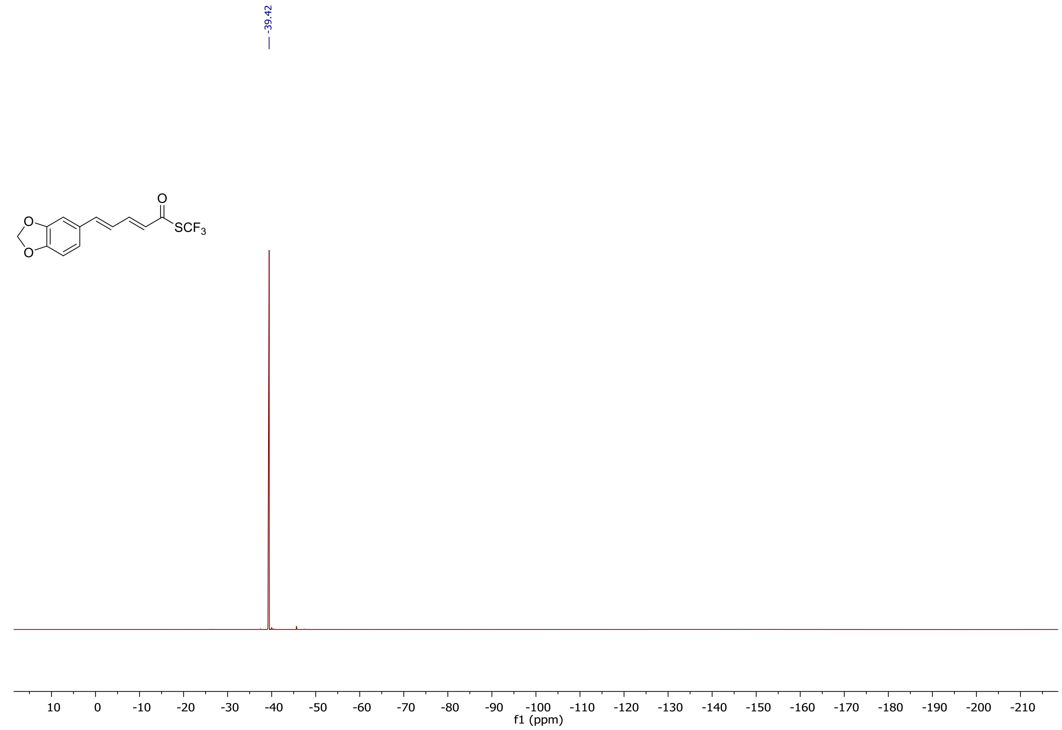
-39.42
SCF3
10
0
-10
-20
-30
-40
-50
-60
-70
-80
-90
-100
-110
-120
-130
-140
-150
-160
-170
-180
-190
-200
-210
f1 (ppm)

Figure S43. NMR spectra of 6f

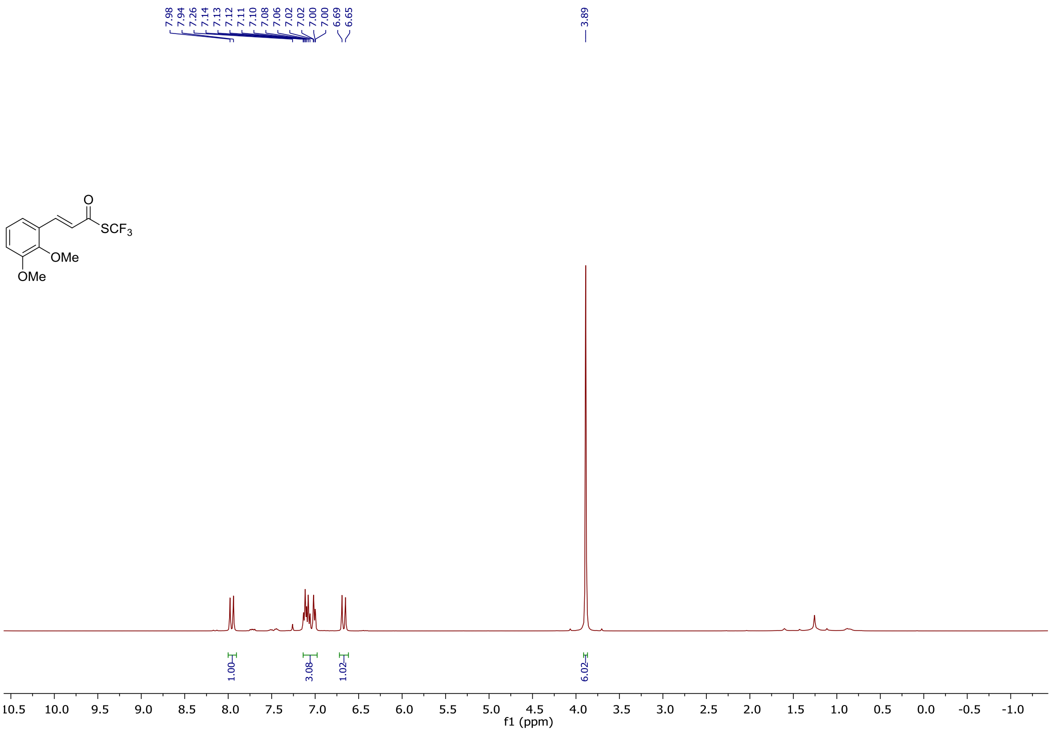

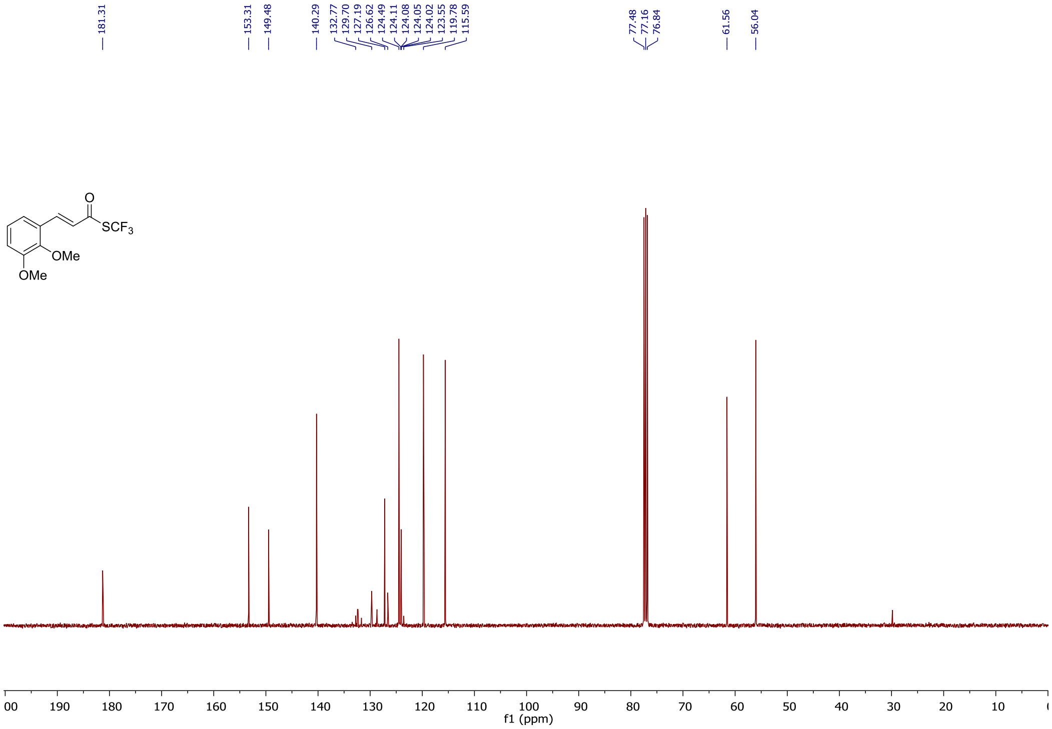

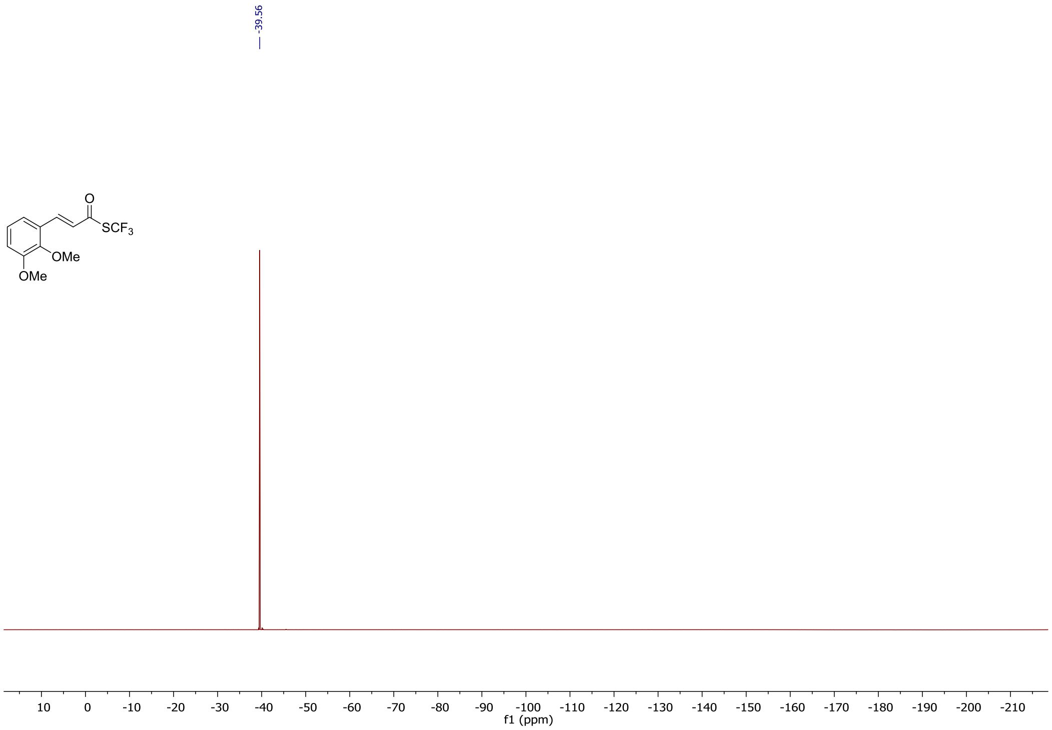
-39.56
SCF3
OMe
OMe
10 0 -10 -20 -30 -40 -50 -60 -70 -80 -90 -100 -110 -120 -130 -140 -150 -160 -170 -180 -190 -200 -210
f1 (ppm)

Figure S44. NMR spectra of 6g

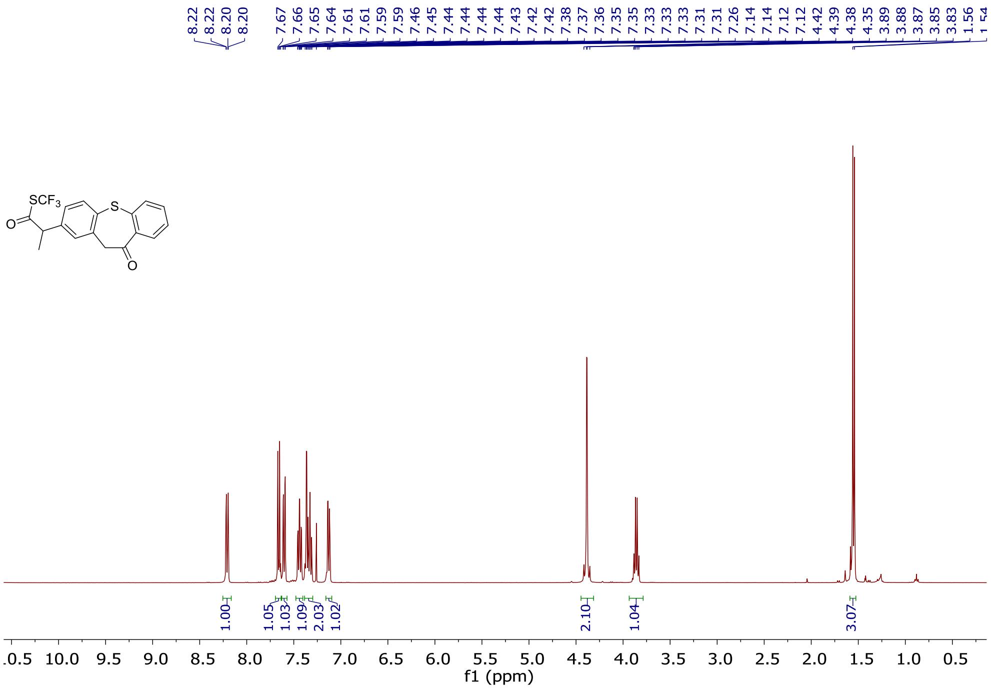

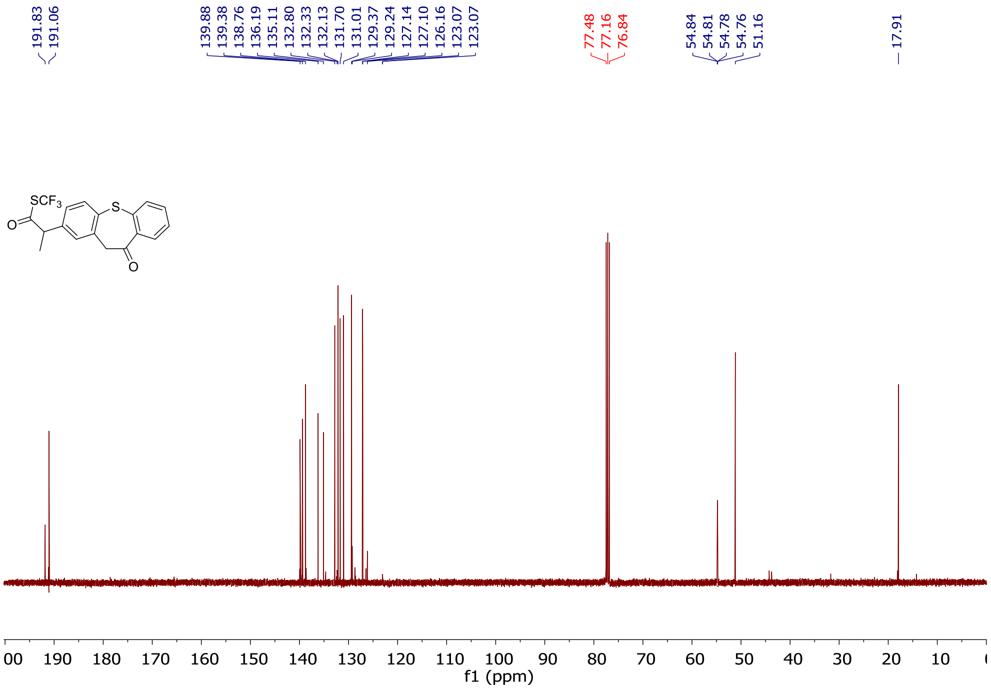

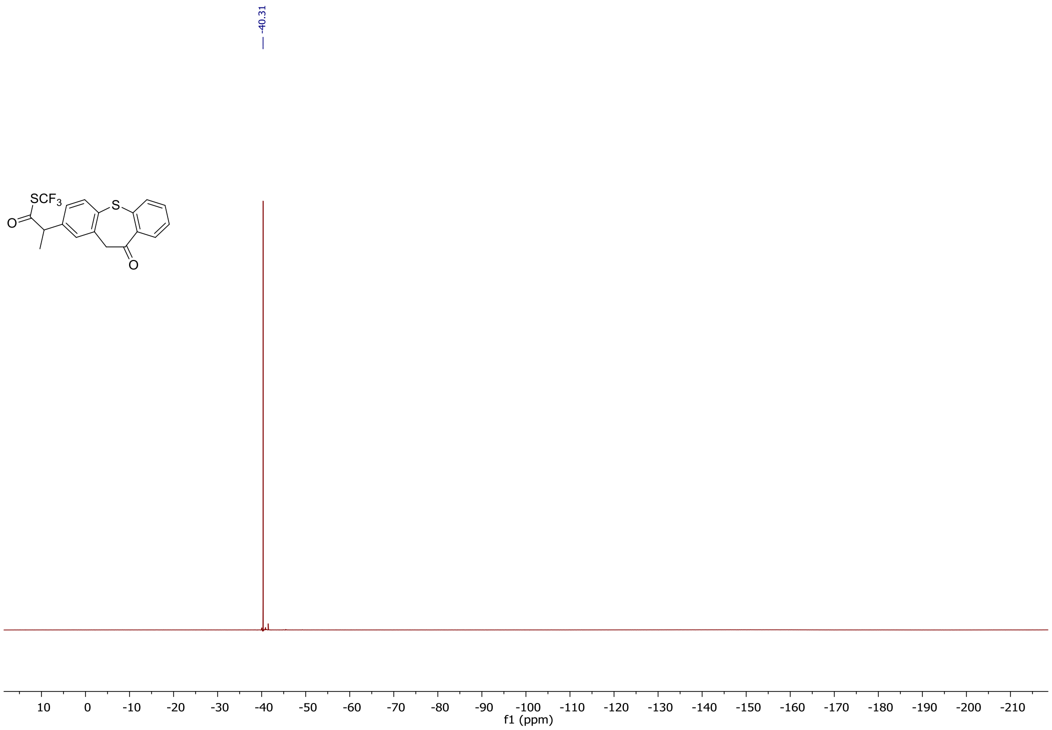

-40.31
SCF3
S
O
O
10
0
-10
-20
-30
-40
-50
-60
-70
-80
-90
-100
-110
-120
-130
-140
-150
-160
-170
-180
-190
-200
-210
f1 (ppm)

Figure S45. NMR spectra of 6h

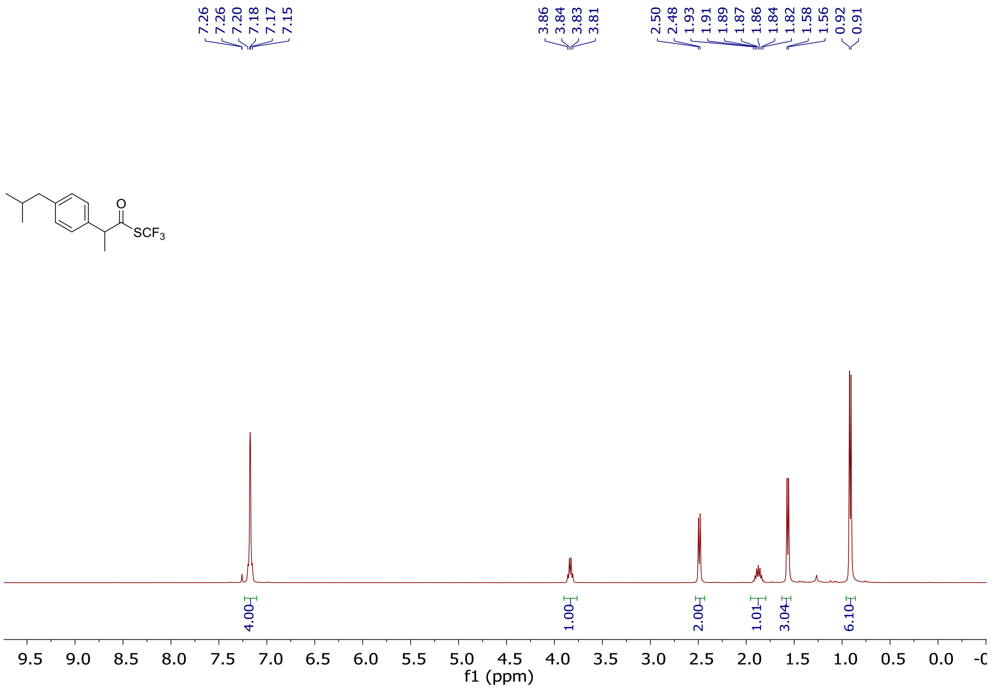

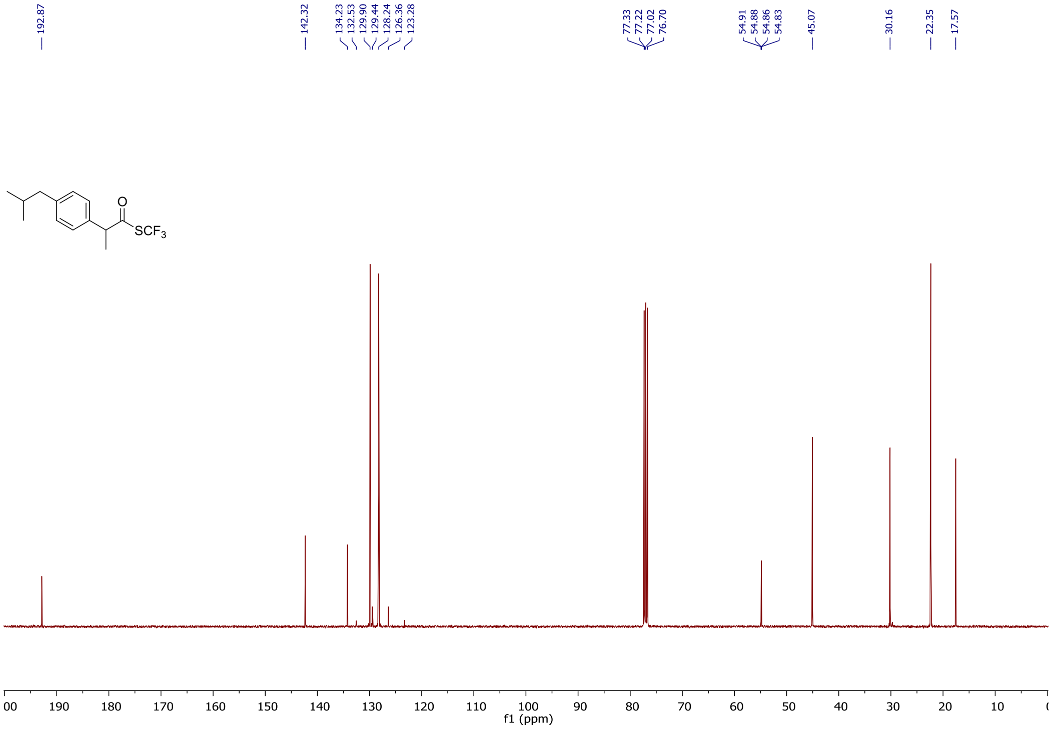

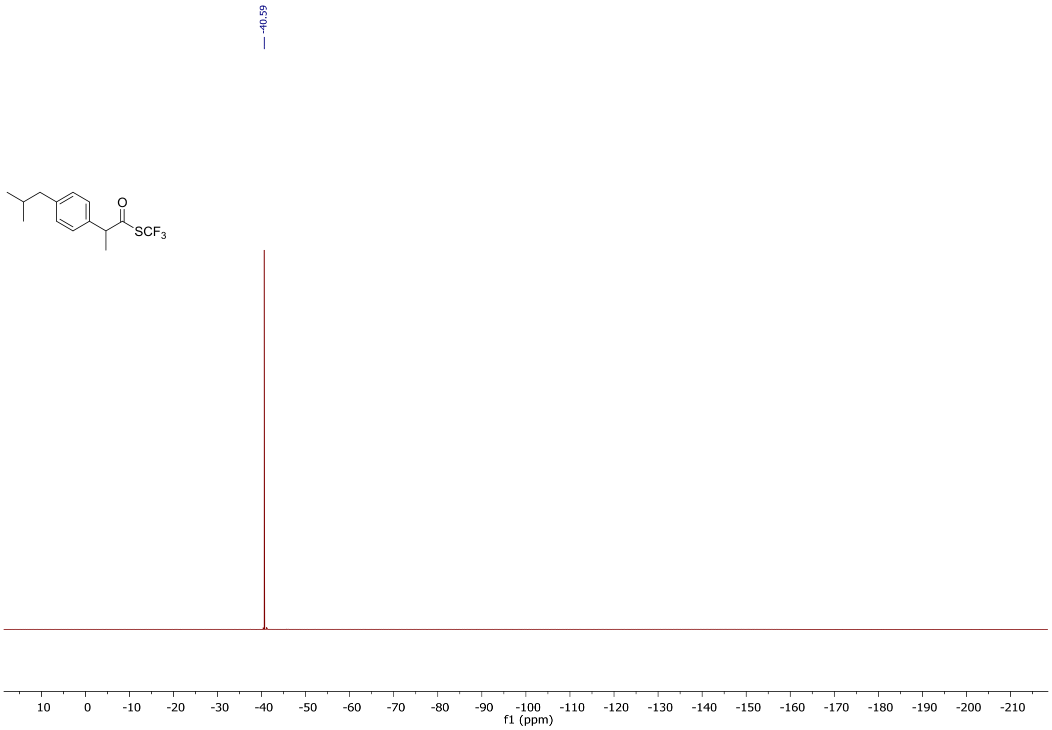
-40.59
SCF3
O
10
0
-10
-20
-30
-40
-50
-60
-70
-80
-90
-100
-110
-120
-130
-140
-150
-160
-170
-180
-190
-200
-210
f1 (ppm)

Figure S46. NMR spectra of 6i

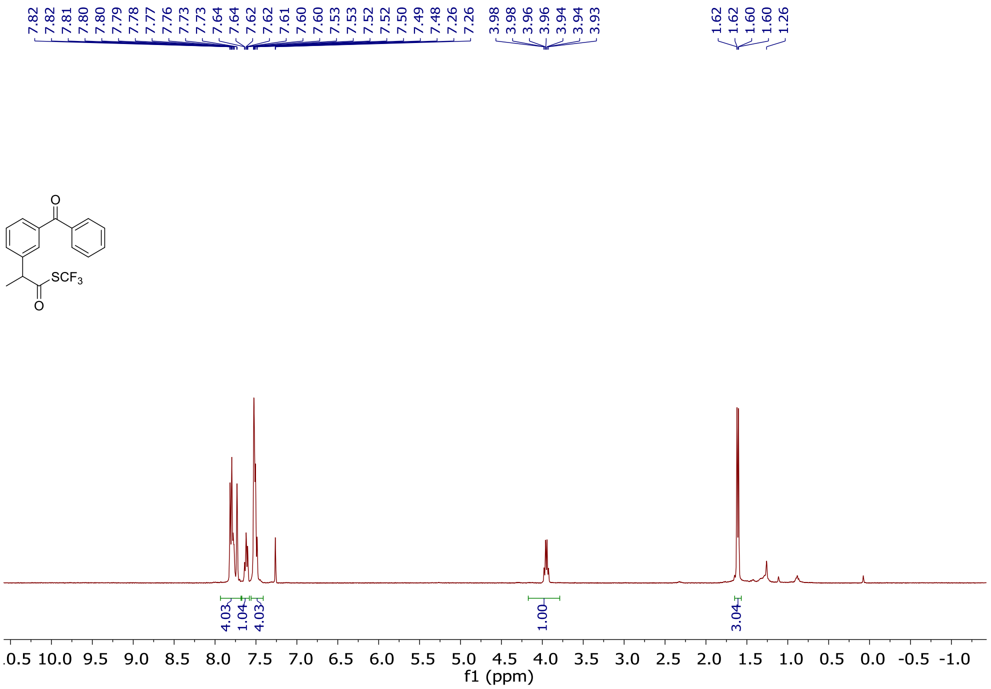

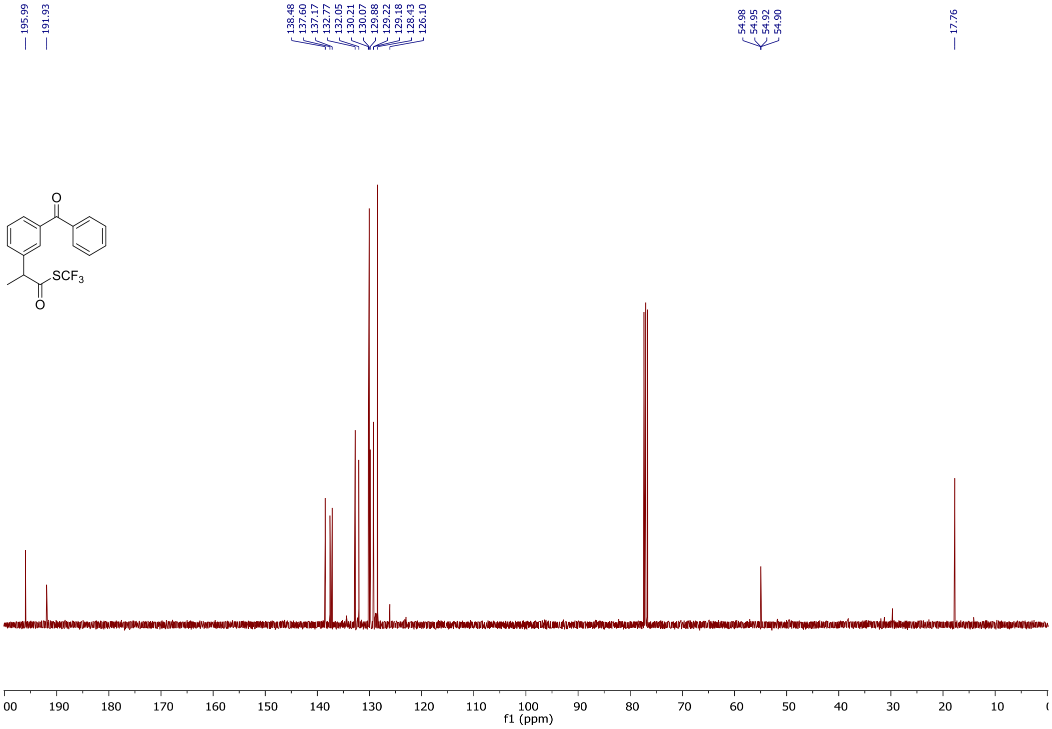

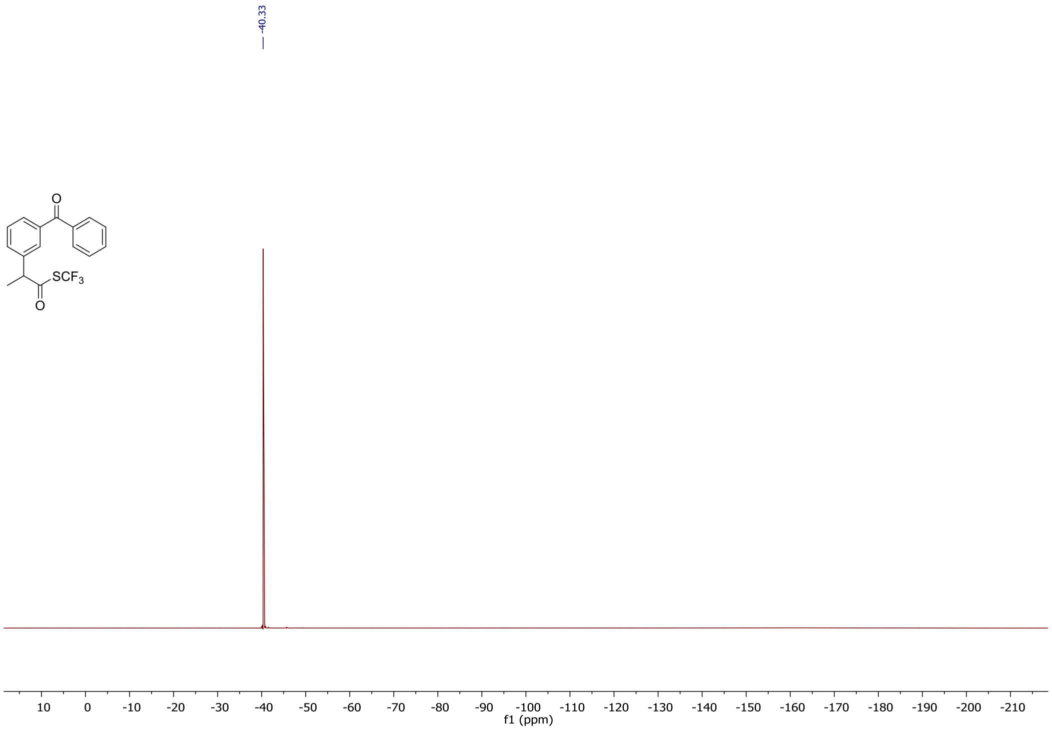

-40.33
SCF3
O
10 0 -10 -20 -30 -40 -50 -60 -70 -80 -90 -100 -110 -120 -130 -140 -150 -160 -170 -180 -190 -200 -210
f1 (ppm)

Figure S47.   NMR spectra of 6j

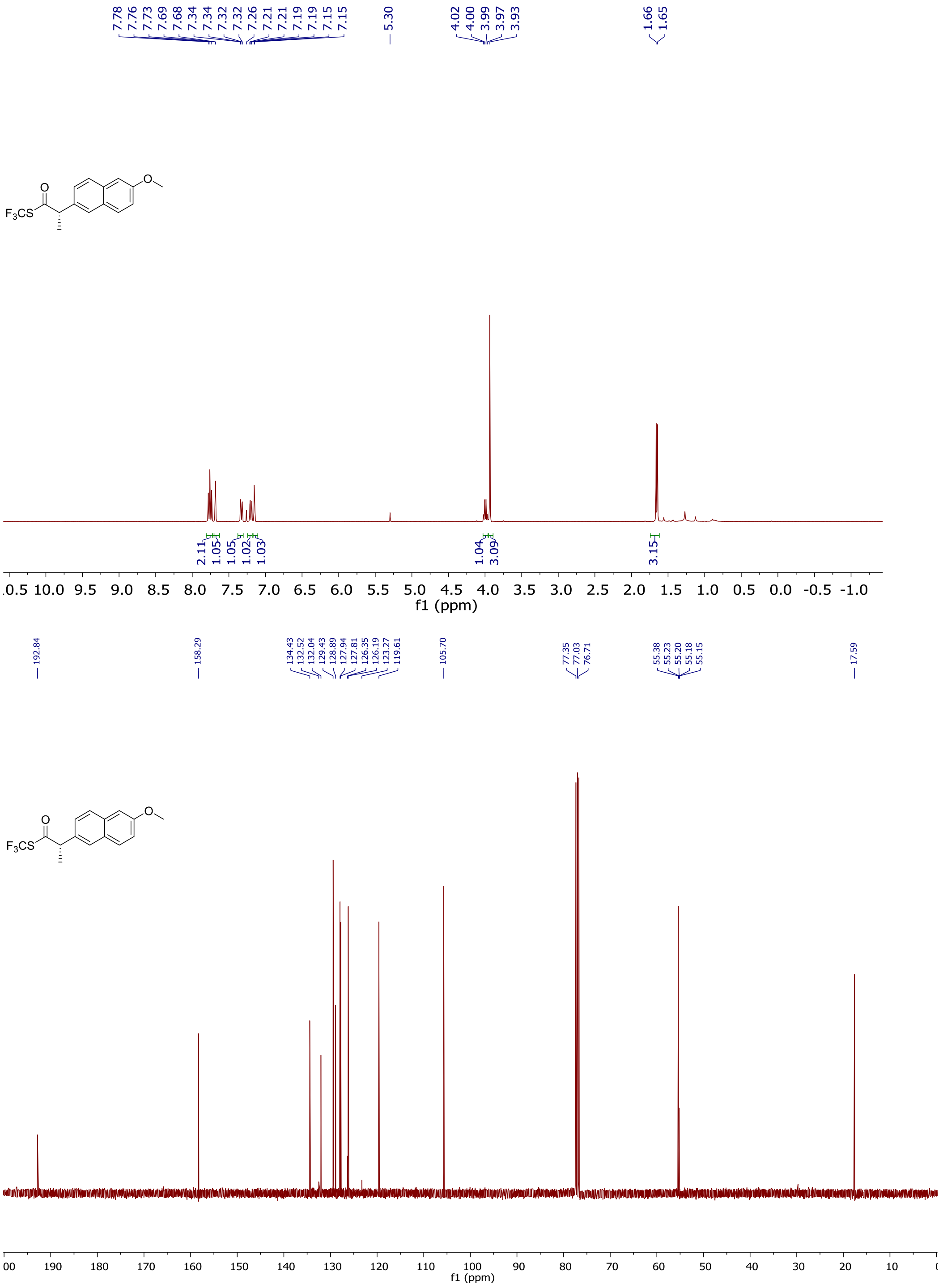

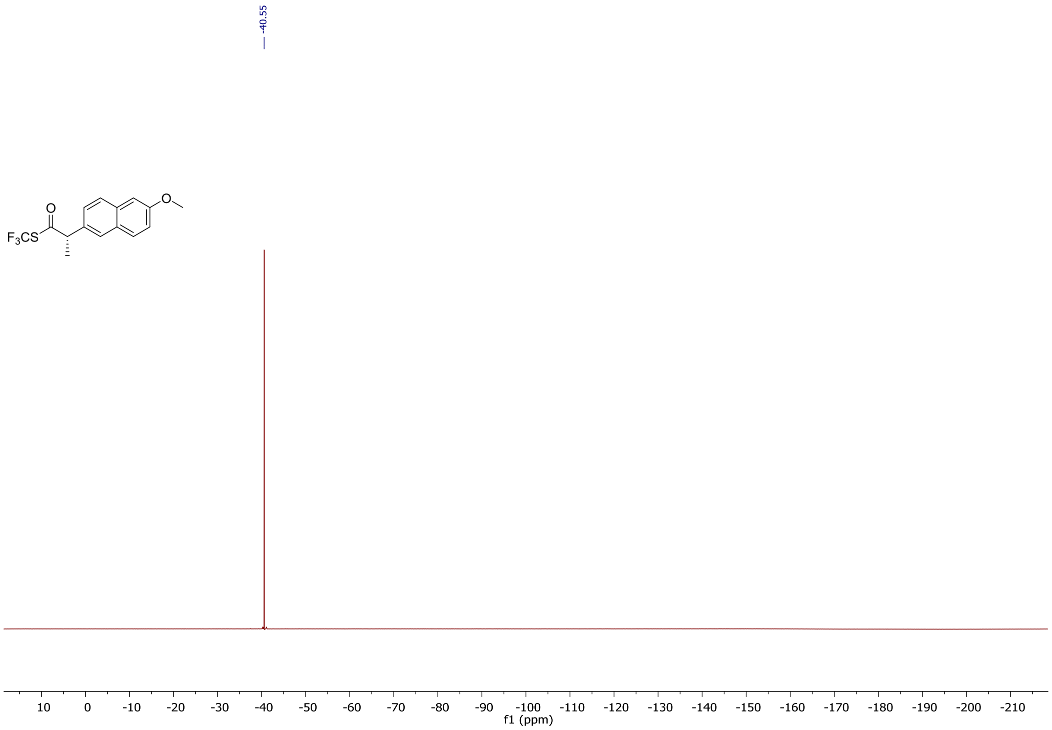

-40.55
F3CS
O
O
10 0 -10 -20 -30 -40 -50 -60 -70 -80 -90 -100 -110 -120 -130 -140 -150 -160 -170 -180 -190 -200 -210
f1 (ppm)

Figure S48. NMR spectra of 6k

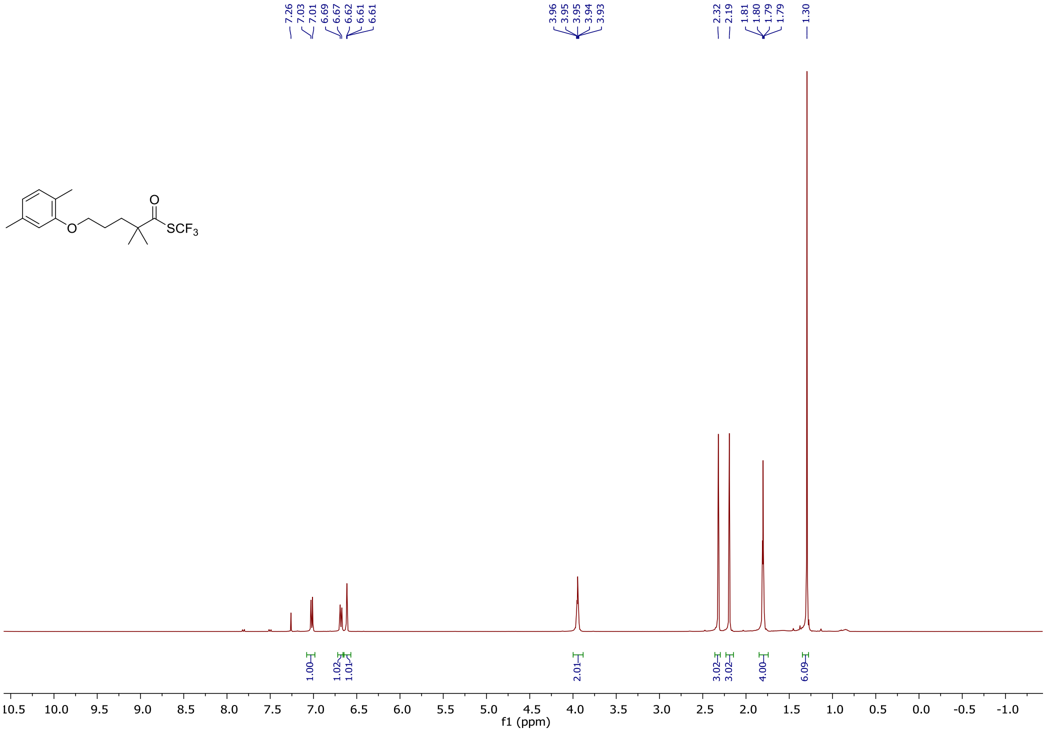

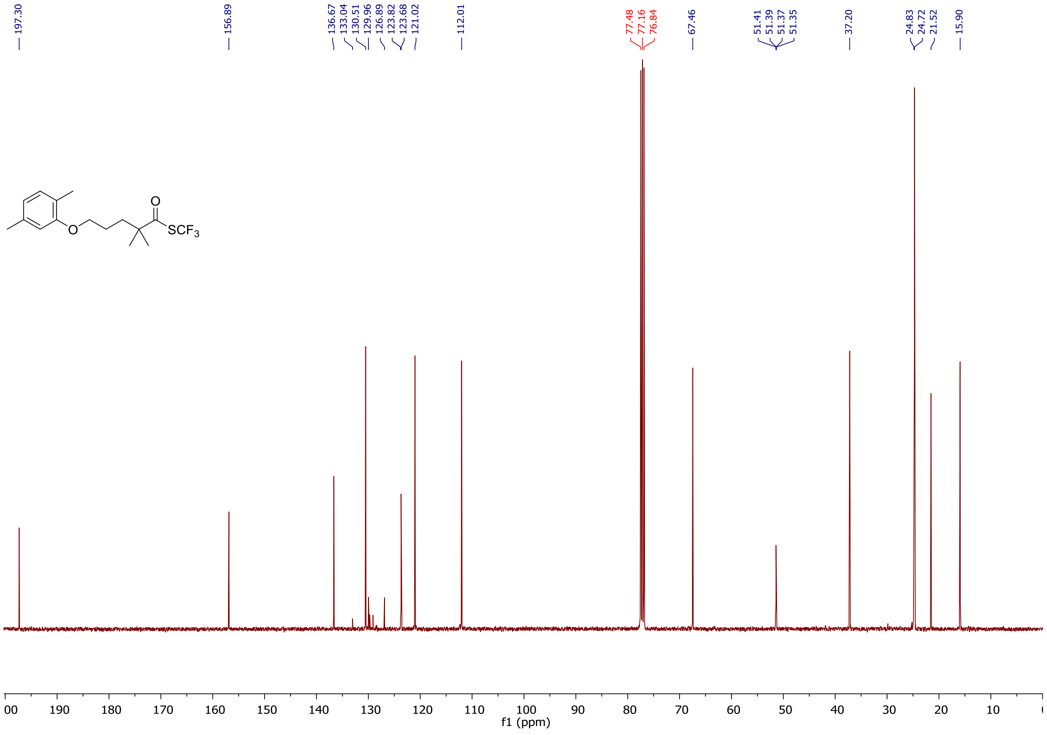

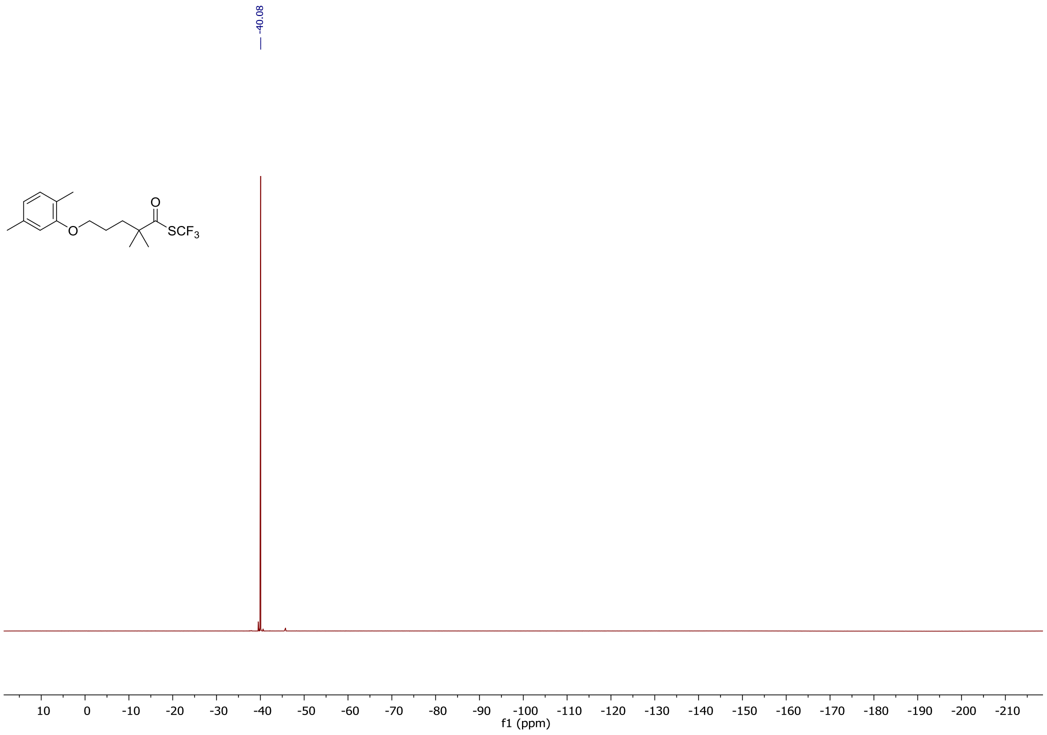
-40.08
O
SCF3
10
0
-10
-20
-30
-40
-50
-60
-70
-80
-90
-100
-110
-120
-130
-140
-150
-160
-170
-180
-190
-200
-210
f1 (ppm)

Figure S49. NMR spectra of 6l

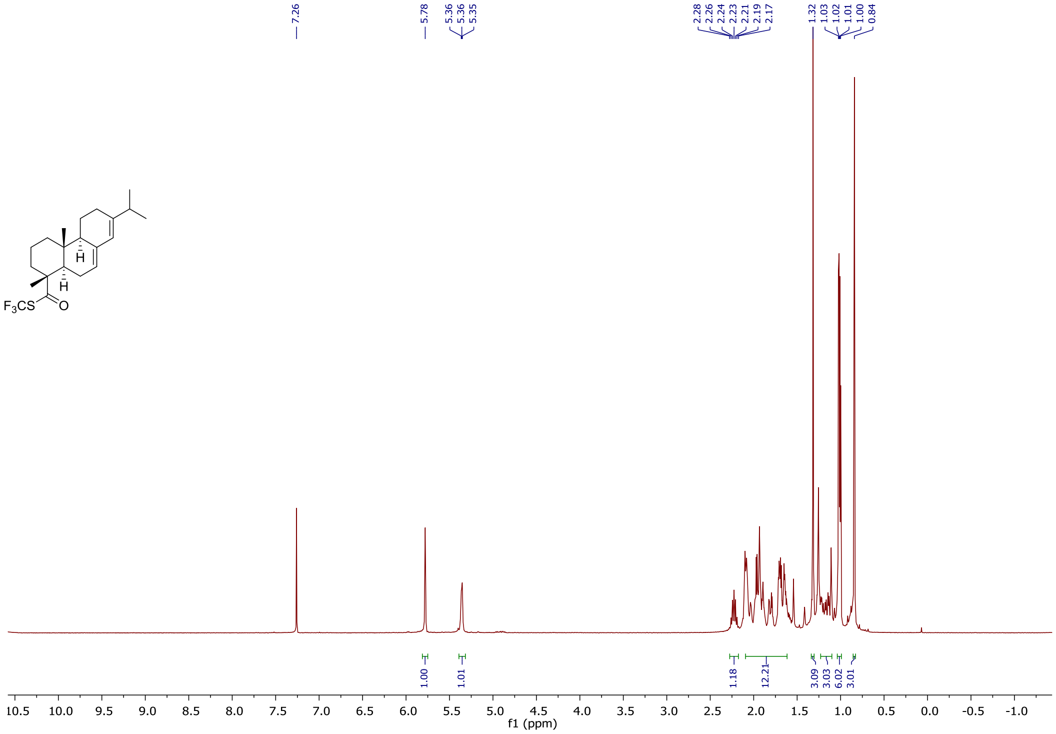

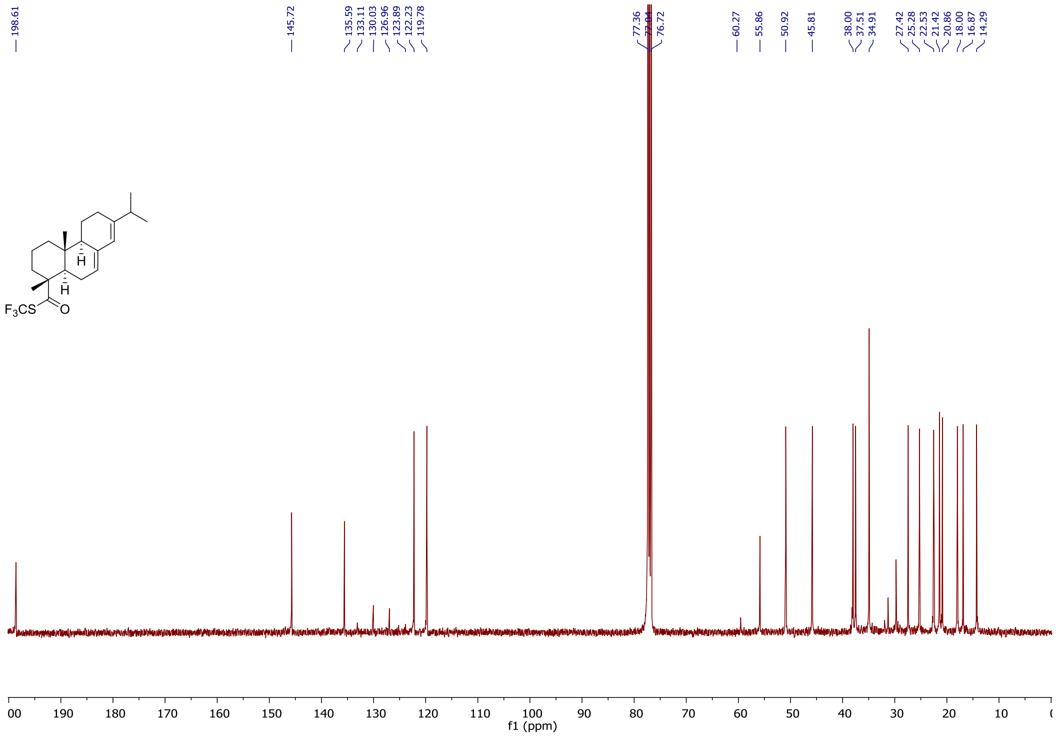

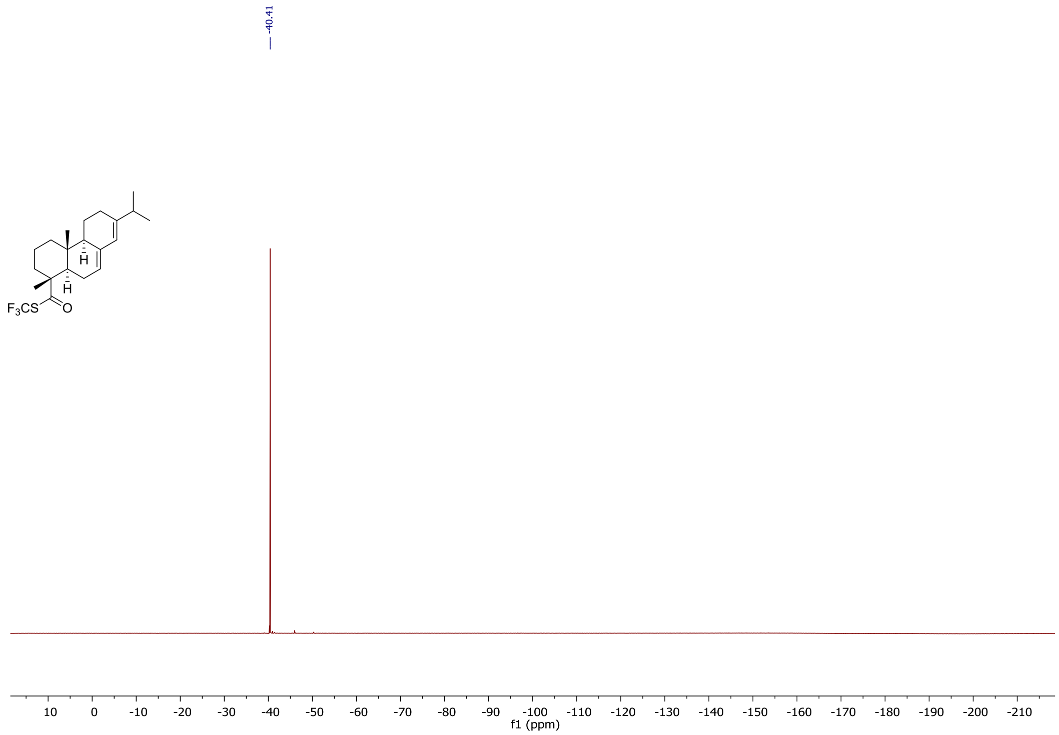
-40.41
F3CS
O
H
H
10 0 -10 -20 -30 -40 -50 -60 -70 -80 -90 -100 -110 -120 -130 -140 -150 -160 -170 -180 -190 -200 -210
f1 (ppm)

Figure S50. NMR spectra of 7a

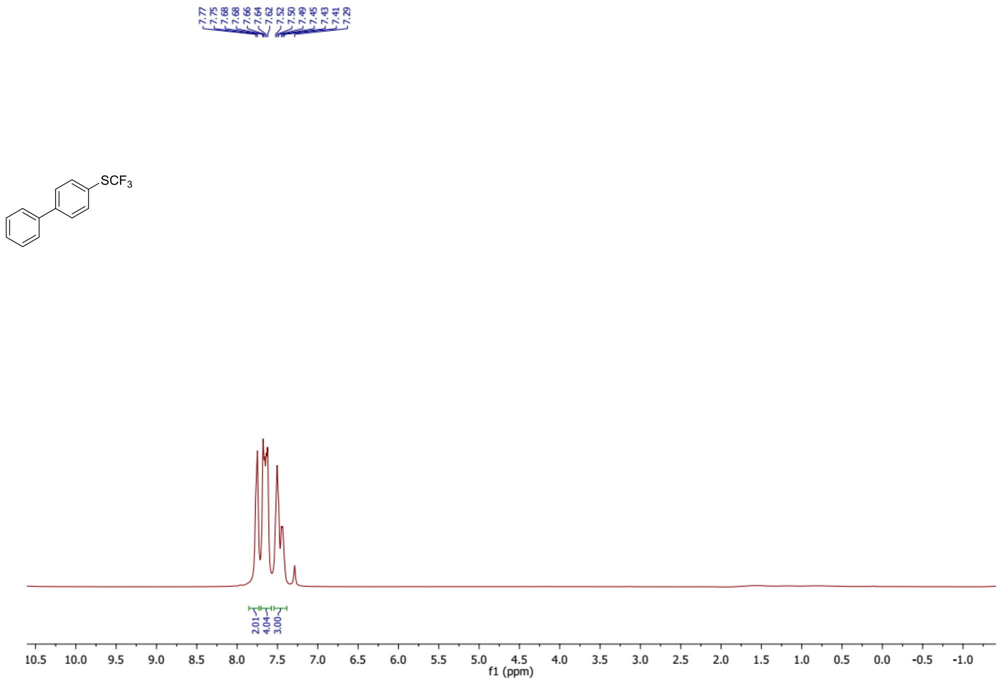

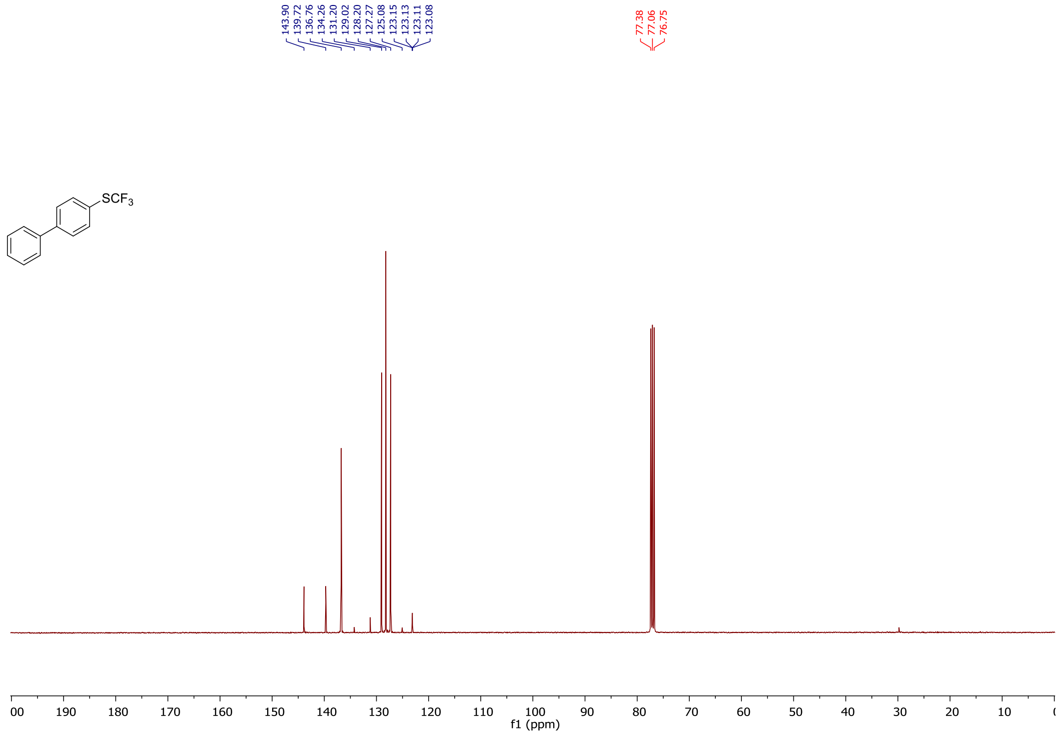

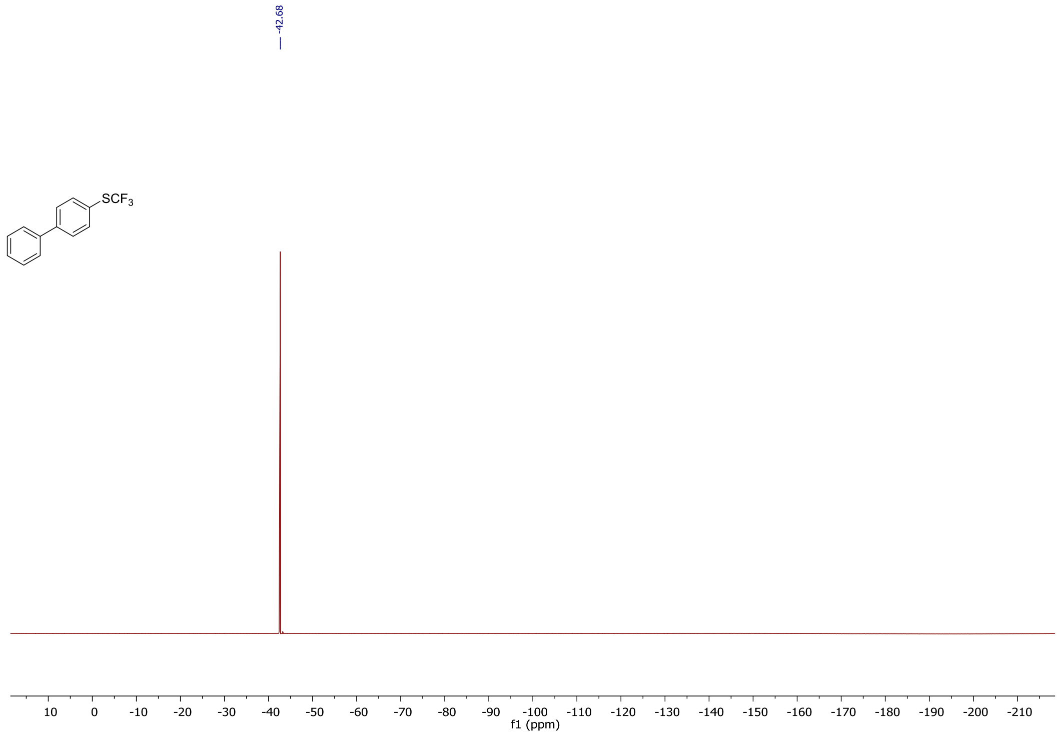
-42.68
SCF3
10 0 -10 -20 -30 -40 -50 -60 -70 -80 -90 -100 -110 -120 -130 -140 -150 -160 -170 -180 -190 -200 -210
f1 (ppm)

Figure S51. ^{31}**P NMR study of reaction**

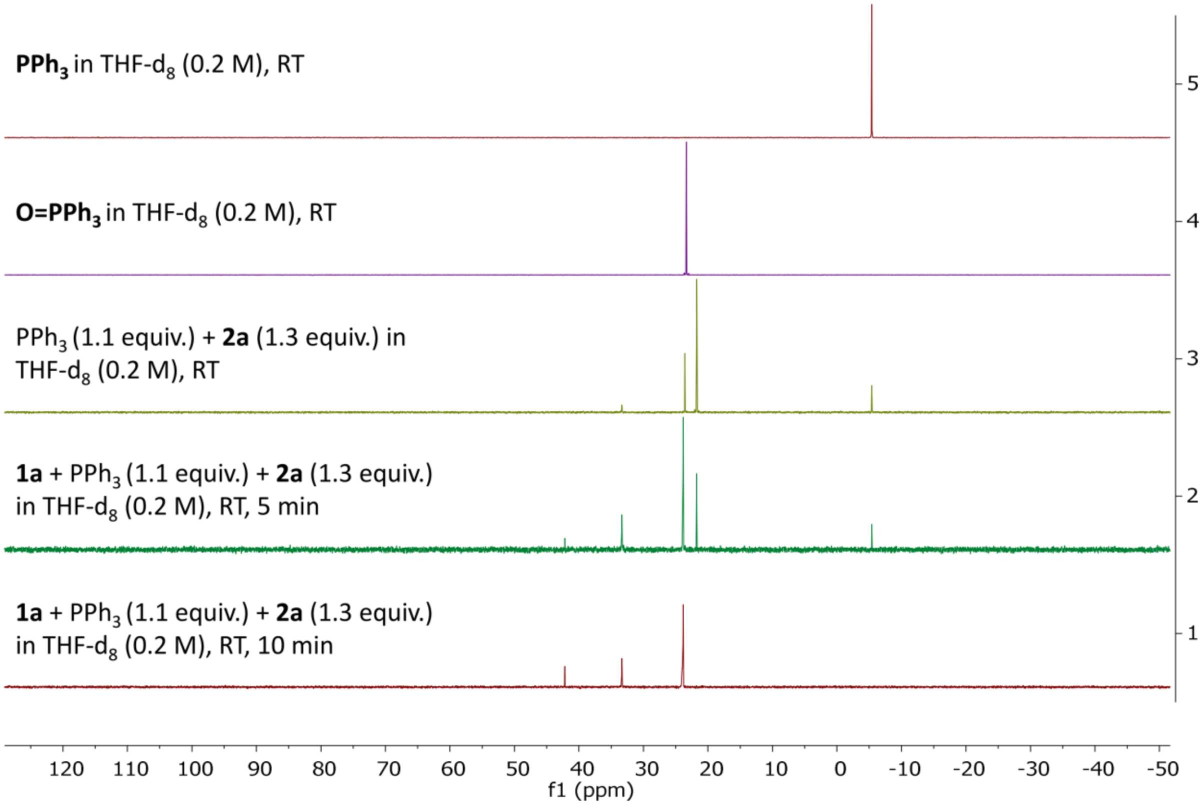

To probe the presence of different P species during the reaction, ^{31}P spectra of various species and the reaction mixture were collected. Addition of *N*-(Trifluoromethylthio)phthalimide (**2a)** to a solution of PPh_3 in THF-d_8 at room temperature led to the formation of a species with a ^{31}P signal at δ = 21.8. This peak was tentatively assigned to trifluoromethylthiophosphonium ion **II**. When **2a** was added to a mixture of PPh_3 and 4-Phenylbenzoic acid (**1a)**, new signals appeared at δ = 33.3, 42.2 and 23.3 (triphenylphosphine oxide) while the ^{31}P signal at δ 21.8 decayed. The peaks at δ = 33.3 and 42.2 are tentatively assigned to the acyloxyphosphonium intermediates including **III**.

References

1. Fulmer, G. R.; Miller, A. J. M.; Sherden, N. H.; Gottlieb, H. E.; Nudelman, A.; Stoltz, B. M.; Bercaw, J. E.; Goldberg, K. I. *Organometallics* **2010,** *29*, 2176.
2. Mukherjee, S.; Patra, T.; Glorius, F. *ACS Catalysis* **2018,** *8*, 5842.
3. Zhang, M.; Chen, J.; Chen, Z.; Weng, Z. *Tetrahedron* **2016,** *72*, 3525.
